# Ancient DNA reveals reproductive barrier despite shared Avar-period culture

**DOI:** 10.1038/s41586-024-08418-5

**Published:** 2025-01-15

**Authors:** Ke Wang, Bendeguz Tobias, Doris Pany-Kucera, Margit Berner, Sabine Eggers, Guido Alberto Gnecchi-Ruscone, Denisa Zlámalová, Joscha Gretzinger, Pavlína Ingrová, Adam B. Rohrlach, Jonathan Tuke, Luca Traverso, Paul Klostermann, Robin Koger, Ronny Friedrich, Karin Wiltschke-Schrotta, Sylvia Kirchengast, Salvatore Liccardo, Sandra Wabnitz, Tivadar Vida, Patrick J. Geary, Falko Daim, Walter Pohl, Johannes Krause, Zuzana Hofmanová

**Affiliations:** 1 State Key Laboratory of Genetic Engineering, Collaborative Innovation Center for Genetics and Development, and Human Phenome Institute, Fudan University, Shanghai, China; 2 Department of Archaeogenetics, Max Planck Institute for Evolutionary Anthropology, Leipzig, Germany; 3 Department of Anthropology and Human Genetics, School of Life Sciences, Fudan University, Shanghai, China; 4 Institute of Medieval Research, Austrian Academy of Sciences, Vienna, Austria; 5 Department of Anthropology, Natural History Museum Vienna, Vienna, Austria; 6 Department of Archaeology and Museology, Faculty of Arts, Masaryk University, Brno, Czech Republic; 7 School of Computer and Mathematical Sciences, The University of Adelaide, Adelaide, Australia; 8 Doctoral School of Ecology and Evolution, University of Vienna, Vienna, Austria; 9 Curt-Engelhorn-Center for Archaeometry, Mannheim, Germany; 10 Department of Evolutionary Anthropology, University of Vienna, Vienna, Austria; 11 HEAS Human Evolution and Archaeological Science Network, University of Vienna, Vienna, Austria; 12 Institute for Austrian Historical Research, University of Vienna, Vienna, Austria; 13 Institute of Archaeological Sciences, ELTE—Eötvös Loránd University, Budapest, Hungary; 14 Institute of Archaeology, Research Centre for the Humanities, HUN-REN—Hungarian Research Network, Budapest, Hungary; 15 Institute for Advanced Study, Princeton, NJ USA

**Keywords:** Comparative genomics, History, Archaeology, Biological anthropology

## Abstract

After a long-distance migration, Avars with Eastern Asian ancestry arrived in Eastern Central Europe in 567 to 568 ce and encountered groups with very different European ancestry^1,2^. We used ancient genome-wide data of 722 individuals and fine-grained interdisciplinary analysis of large seventh- to eighth-century ce neighbouring cemeteries south of Vienna (Austria) to address the centuries-long impact of this encounter^1,2^. We found that even 200 years after immigration, the ancestry at one site (Leobersdorf) remained dominantly East Asian-like, whereas the other site (Mödling) shows local, European-like ancestry. These two nearby sites show little biological relatedness, despite sharing a distinctive late-Avar culture^3,4^. We reconstructed six-generation pedigrees at both sites including up to 450 closely related individuals, allowing per-generation demographic profiling of the communities. Despite different ancestry, these pedigrees together with large networks of distant relatedness show absence of consanguinity, patrilineal pattern with female exogamy, multiple reproductive partnerships (for example, levirate) and direct correlation of biological connectivity with archaeological markers of social status. The generation-long genetic barrier was maintained by systematically choosing partners with similar ancestry from other sites in the Avar realm. Leobersdorf had more biological connections with the Avar heartlands than with Mödling, which is instead linked to another site from the Vienna Basin with European-like ancestry. Mobility between sites was mostly due to female exogamy pointing to different marriage networks as the main driver of the maintenance of the genetic barrier.

## Abstract (web summary)

Ancient genome-wide data of 722 individuals and interdisciplinary analysis of large seventh- to eighth-century ce neighbouring cemeteries near Vienna are used to address the impact of the encounter between Eastern Asian Avars and Europeans.

## Main

In 552 ce, the Turks destroyed the Empire of the Avars (called ‘Rouran’ in Chinese texts) in present-day Mongolia, and numerous Avars and other steppe riders migrated to Europe and settled in the Carpathian Basin (mainly modern Hungary) in 567–568 ce^1,2^. According to written accounts, they dominated an ethnically and culturally heterogeneous population including Bulgars, Gepids, Slavs and Romans, and repeatedly raided East Roman provinces in the Balkan Peninsula, culminating in a failed siege of Constantinople in 626 (refs. ^2,5^). Their realm in Eastern Central Europe continued in a more peaceful fashion until Frankish armies destroyed it in roughly 800 ce^6^.

A rich archaeological record from almost 100,000 graves shows how the cultural diversity of the early Avar period gave way to the homogeneous culture of the eighth century^5,7–9^. Attestations of ethnic multiplicity disappear, and the texts only mention Avars in their realm. Recent archaeogenetic studies have confirmed the Eastern Asian ancestry of the Avar elite in the seventh century ce and traced further population development in the Avar core area, the Danube–Tisza Interfluve (DTI) and Transtisza regions^1,10^. In this study, we address a peripheral region of the Avar Empire, the Vienna Basin in eastern Austria, in the later period of Avar rule (roughly 650–800 ce)^11^. This was a contact zone to the west, sparsely populated before the seventh century when new groups settled there^9,11^.

The texts describe the multiplicity of groups and movements using ethnic designations^12^. Such ethnic labels did (and do) not necessarily correspond to groups of common origin and of shared culture^12,13^, contrary to popular perceptions (for example, in direct-to-consumer ancestry genetic testing) that genetic, archaeological, linguistic and historical evidence would fully align to define ethnic groups^14,15^ (Supplementary Information, sections 1 and 2c). On the basis of a thorough interdisciplinary interpretation of new genetic data, we reconstructed the social structure and behavioural patterns of local communities, and showed long-term yet highly diverse impact of this historically well-attested^7^ major migration on population development and cultural habitus, including a lasting reproductive barrier within the Avar society.

To fully understand the effect of Avar migration and dominion, we sampled two entire cemeteries from the late-Avar period at Leobersdorf (LEO, seventh to early ninth century ce, 155 samples) and Mödling-An der Goldenen Stiege for genomic analysis (MGS, 485 samples, from the same period; Supplementary Table 1). For comparison, we also included two small burial groups from an earlier period at Mödling, Mödling-Lerchengasse (MLS, two samples, second half of the fourth century ce) and Mödling-Leinerinnen (MLE, five samples, mid-sixth century ce). Furthermore, we selected 83 samples from the large cemetery at Wien-Csokorgasse (CSK, 745 graves dated between the seventh and ninth century ce) to test the archaeological hypothesis of a mid-eighth-century immigration^16^ (Supplementary Information, section 3b). Altogether, thanks to the excellent preservation, we newly analysed authentic genome-wide DNA from skeletal samples of 722 individuals from the Vienna Basin obtaining a median around 626,000 of 1,240,000 targeted positions for relatedness estimation with several state-of-the-art approaches (the full dataset) and for investigation of genetic ancestry (677 individuals with minimal contamination 5%) (Methods and Supplementary Table 1).

## Contrast between genetic ancestries

**Fig. 1:**
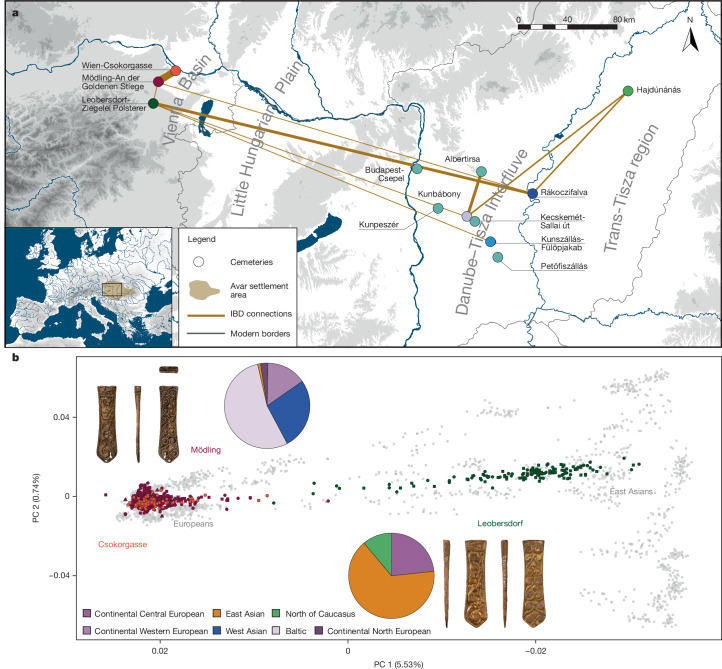
Geographic locations and genetic profiles of ancient individuals in this study. **a**, Map of studied archaeological sites and the average IBD sharing pattern among the studied archaeological sites. The inset shows the location of the Avar settlement area in Europe between the late seventh and eighth century. The IBD connections among the three archaeological sites from the Vienna Basin (1) and the Avar-period sites from the DTI and Transtisza (TT) regions (2)^10^ are highlighted in beige lines, where the width of the line reflects the amount of IBD sharing between sites (Methods). The map is based on Natural Earth Data (projection ED Lambert Europe; https://www.naturalearthdata.com/). **b**, PCA within the context of Eurasian variations. The PC space is calculated from 149 modern-day Eurasian populations, which are shown in grey dots. The newly reported ancient individuals are highlighted with coloured symbols. The PC1 values were inverted on the *x* axis. The pie charts show mean ancestries in the respective populations (based on individual-based results, see Supplementary Information, sections 5 and 7c and Supplementary Table 2). The strap ends with animal combat scenes indicate the cultural similarity between the cemeteries of Leobersdorf and Mödling. Credit: Photograph in **b** by Benedict Seidl.

**Extended Data Fig. 1:**
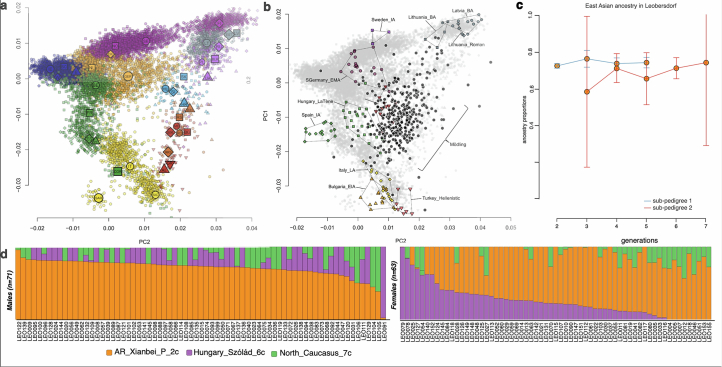
Genetic ancestry analyses. **a)** PCA on a set of present-day 9,472 European individuals based on 426,135 autosomal SNPs. The mean PC1 and PC2 coordinates for each of the 36 present-day European populations are shown in bold symbols. **b)** Projection of published and the newly-generated ancient individuals in this study into the PC space shown in subpanel a). **c)** East Asian ancestry in Leobersdorf pedigree per generation, see pedigree in Fig. 2. **d)** Genetic ancestry proportions of Leobersdorf individuals. Exact ancestry proportions are reported in Supplementary Table 2. Individuals buried at Leobersdorf exhibit a highly connected social structure with relatively equal sex ratio (females: males = 63:71, and 2 individuals with unassigned sex). Overall, more females than males buried in Leobersdorf exhibit ancient central European ancestry (“Hungary_Szólád_6c”). By contrast, all males preserve ancient northeast Asian ancestry (“AR_Xianbei_P_2c”), and over half of them combined with certain ancestry from the Pontic steppes (“North_Caucasus_7c”).

LEO, MGS and CSK all lie in a radius of 20 km, and have been archaeologically classed as typical mid-to-late-Avar-period sites (roughly mid-seventh to early ninth ce)^3,4^. However, we found fundamental genetic differences between them. The main cluster of Leobersdorf individuals differs from individuals from Mödling and Csokorgasse (Fig. 1 and Methods) up to the same level as modern-day populations living in Europe differ from those in East Eurasia as measured by the Euclidean distance on principal components and *F*_ST_ (fixation index) distance (Supplementary Fig. 14). Even at the margins of the Avar realm and until the very end of Avar rule around 800 ce, East Asian ancestry was prevalent in the local community of Leobersdorf with individuals carrying at median 71.5% East Asian ancestry (Fig. 1, Extended Data Fig. 1 and Supplementary Table 2). The genetic ancestry of all Leobersdorf individuals could be traced back to three major ancestry sources (Supplementary Table 2): prevalently, the Iron Age northeast Asian ancestry using ‘AR_Xianbei_2thCE’ from Amur River Basin as the proxy^17^, a steppe ancestry from the Pontic steppes, using ‘North_Caucasus_7thCE’ as the proxy^18^ and to a minor extent also the pre-Avar Carpathian Basin ancestry using ‘Hungary_Szólád_6thCE’ as the proxy^19^. Not unlike previous findings^10^, admixture between East Asian and pre-Avar sources began earlier (142 ± 15 years before LEO), assuming the reference populations as proximal ancestry sources (Supplementary Table 2). Eighty-two Leobersdorf individuals also derive 0.9–61.4% ancestry from the Pontic steppes north of the Caucasus (Extended Data Fig. 1 and Supplementary Table 2) in an admixture process that had started around 300 years before (319 ± 174 years before LEO) (Supplementary Table 2), which suggests that the steppe element in the Avar-period society was in itself heterogeneous. The average proportion of East Asian ancestry at LEO stays around 70% across the roughly 150 years of the cemetery’s use, and no homogenization occurs through time (Extended Data Fig. 1, Supplementary Table 2 and Supplementary Information, sections 4 and 5).

In contrast to Leobersdorf and to contemporary sites in the Avar core region, East Asian ancestry is minimal in Mödling (less than 5% on average, Supplementary Table 2). Instead, the main cluster formed by the Avar-period MGS (seventh to eighth century ce) population is similar to the cluster of modern-day Europeans (Fig. 1), on that resolution, similar to pre-Avar MLE and MLS individuals. In a roughly contemporary high-resolution context, MGS falls between the pre-Avar population of Hungary_Szólád_6thCE and the genetic cline stretching from Hellenistic Anatolia to Iron Age Iberia (Extended Data Fig. 1). In line with this principal component analysis (PCA) placement, qpWave analyses showed no direct local continuity between MLE_6thCE and MGS, and proximal modelling of MGS ancestry suggests that further ancestral components are required (Supplementary Table 2 and Supplementary Information, section 1). On the other hand, individuals from MGS show connections to the two small pre-Avar Mödling cemeteries through shared identical-by-descent (IBD) genomic segments: an indicator of direct biological relatedness (more than 100 individual pairwise connections, Supplementary Table 3). Grave apparel (such as tweezers and combs), burial of dogs^20^ and chronologically overlapping ^14^C ranges of the Mödling sites might indicate some local continuity (Supplementary Information, section 3).

Distal modelling of the MGS site reveals complex admixture roughly datable to the early Roman Imperial Age (roughly the first century bce to the second century ce), in which predating Eastern Mediterranean ancestry (Turkey_Hellenistic/Turkey_IA) mixed with southeastern European ancestry first (Slovenia_EIA/Croatia_IA) and later with north-eastern European ancestry (Lithuanian_BA/Pohansko_9c). The small component of East Asian ancestry was added roughly 200 years before MGS, most plausibly under early Avar rule (Extended Data Fig. 1 and Supplementary Information, section 6). The north-eastern European ancestry had largely been absent from the Carpathian and Vienna Basins in the previous period (fifth to sixth century ce), and can be observed for the first time on a large scale at MGS in the seventh to eighth century ce. Previously reported occurrences in Eastern Central and Southeastern Europe are only attested at later dates, which is not least due to prevalence of cremation burials in seventh to ninth century eastern Europe^21–23^. Similar ancestry is found in the ninth century ce in the Volga-Oka region in Russia and in Pohansko in the Czech Republic^24^ (Supplementary Information, section 6); it is also present in modern-day populations in Eastern Central Europe^23,25^. It is probably connected to what contemporaries since the mid-sixth century ce perceived as an influx of Slavs. However, that does not mean that the people at Mödling need to be regarded as Slavs. They are a result of a long process of admixture, which probably included late-Roman and pre-Avar groups from the Carpathian Basin, but also captives massively transferred by the Avars from Roman provinces in the Balkan Peninsula to Pannonia^2,26,27^. By contrast, individuals of northern European ancestry, prominent in the Carpathian Basin in the fifth to sixth century ce^28^, and groups from the Eurasian steppes had minor roles in the Mödling ancestry (Supplementary Table 2).

The contrasting genetic patterns between two neighbouring Avar-period communities—Mödling and Leobersdorf—suggest limited interactions between them over a period of several generations (Supplementary Table 2), whereas Csokorgasse individuals show very similar ancestry patterns to Avar-period Mödling (Fig. 1 and Supplementary Information, section 6). As in MGS, East Asian ancestry has a marginal role for individuals sampled from CSK (19 of 82 with median 6.6% East Asian ancestry, Supplementary Table 2). Contrary to the initial assumption based on archaeological findings^3^, no eighth-century eastern genetic influx was detected at CSK. In the context of archaeological debates whether objects can migrate without people, this provides a clear example for the spread of cultural objects without migration^29^. The detected admixture of East Asian and European ancestry in Leobersdorf was sex biased with the East Asian component carried more through male individuals. By contrast, the introgression of north-eastern European ancestry into the Mödling population is consistent with transmission by both sexes (Supplementary Information, sections 5c and 6c).

**Extended Data Fig. 2:**
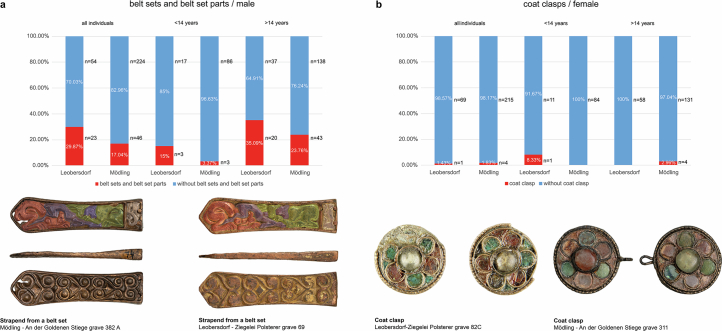
Frequency of individuals buried with status symbols and grave goods in the cemeteries of Leobersdorf and Mödling. **a)** Proportion of male individuals buried with belt sets, together with pictures of belt sets (photo: Benedict Seidl), **b)** Proportion of female individuals buried with coat clasps, together with pictures of coat clasps (left photo: © State Collections of Lower Austria, right photo: Benedict Seidl).

The genetic barrier between neighbouring LEO and MGS does not correspond to strong differences in the archaeological record. Both cemeteries are part of a shared culture of the Avar realm in the eighth century ce^16^, characterized by inhumation in separate burial pits in rows of roughly west–east oriented graves and with at least basic grave goods in most burials. The most conspicuous male markers of status common to both sites are multi-partite belt sets with almost identical cast copper-alloy fittings, often adorned with griffins and ornamental decorations^30–34^ (Fig. 1, Extended Data Fig. 2 and Supplementary Information, section 7). Female graves are often modest, but may contain exceptional prestige items, such as diadems, neck rings, silver bracelets and coat clasps (Extended Data Fig. 2). By contrast, funerary practice was fundamentally different in the Frankish and Longobard kingdoms in the west, where the grave good habit had already ceased^35^; and in surrounding areas of Eastern Central Europe, where Slavs had emerged and cremation of the dead was practised without grave goods or sacrificial objects^36–39^. A good part of the MGS population may have had similar ancestry as Slavic groups, but they shared Avar, not contemporary Slavic cultural habitus^40^.

Genetic outliers that share the cultural habitus of a burial community have been found at other sites^41^, and cultural diversity is documented in the preceding period within the same burial communities^10,19,28^; but to our knowledge, LEO and MGS is a not-yet documented case of fundamental genetic differences between two entire sites with overwhelmingly common cultural traits. The genetic discrepancy cannot be explained by differences in the social status or function of the sites. Leobersdorf was not at a military settlement for the defence of the boundary; just like at MGS, graves contained very few combat weapons and no horse gear, and there is little evidence of trauma or injuries on the skeletons (Supplementary Information, section 7). Many more weapons are found in the Avar core area—for example, at Kunszállás^10^—and a bit more at CSK, which was situated on the old Roman road along the Danube. Neither was LEO an elite settlement of ‘proper’ high-status Avars controlling an area of lower-status westerners at MGS, for indicators of status occur to a similar degree at LEO and MGS. LEO has a slightly higher average of belt sets and belt-fittings (26.4% LEO, 19.7% MGS); at both sites, they were mainly associated with some of the subpedigrees, obviously to display their higher status (Supplementary Information, section 7).

## Reconstruction of reproductive practices

**Fig. 2:**
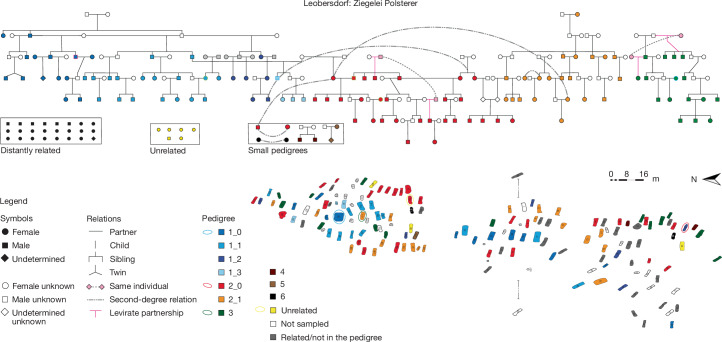
The patrilineal pedigree in Leobersdorf and the cemetery map showing burial locations. The pedigree constructed in Leobersdorf is divided into subpedigree units in specific colours with the subpedigree unit numbers marked in the legend. The cemetery map of all burials in Leobersdorf is filled with colour using the same colour scheme as shown in the pedigree, whereas burials unfit for sampling are unfilled.

**Fig. 3:**
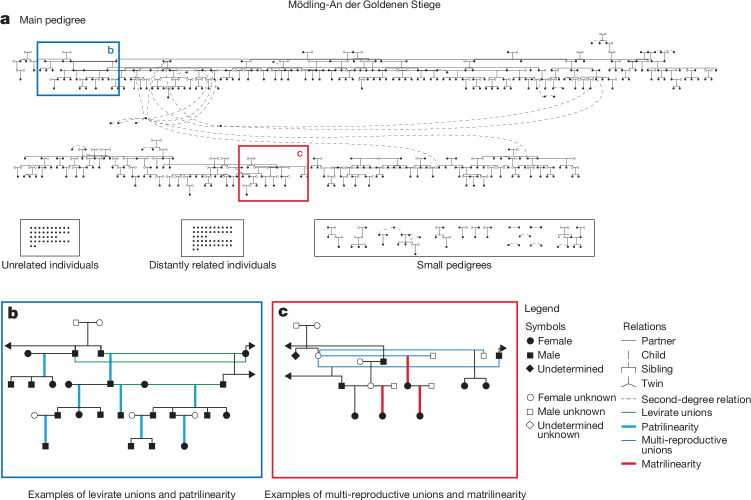
The largest pedigree constructed in Mödling. **a**, Simplified main pedigree in folded view. **b**, A subsection of the pedigree with two levirate scenarios and patrilineal lineages over four generations. **c**, A subsection of the pedigree with matrilineages and multi-reproductive unions.

**Extended Data Fig. 3:**
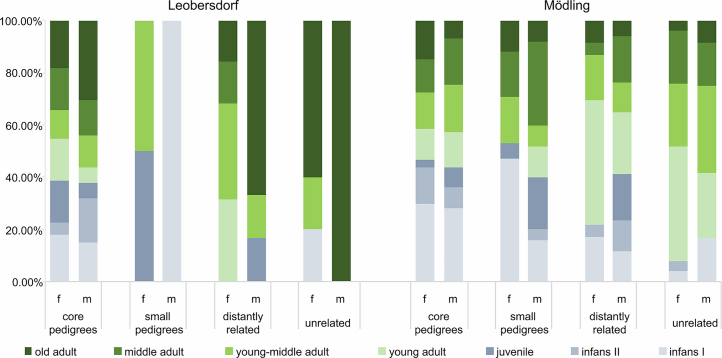
Summary figure on anthropology findings. Distribution of males and females across age-at-death groups in Leobersdorf and Mödling. The high proportion of young and young-middle-aged females in the group of unrelated and distantly related individuals stands out in both cemeteries. Age ranges are: infans I 0–6 years, infans II 7–13 years, juvenile 14–17 years, young adult 18–25 years, young-middle adult 26–35 years, middle adult 36–50 years, old adult 50+ years.

Sampling two large cemeteries as completely as possible enabled us to reconstruct pedigrees for the entire burial community. We built a large pedigree for Mödling based on 356 biologically related individuals including potential twins (Supplementary Table 5), and a six-generation pedigree of 111 individuals for almost the entire Leobersdorf community (Figs. 2 and 3). On average, more than 90% of individuals in Leobersdorf (139 out of 147) and Mödling (450 out of 492) are related through first- to fifth-degree relatedness estimated by KIN (Supplementary Table 4 and Methods). The overall pedigrees are composed of several subpedigrees, often linked by polygamous individuals of both sexes (Supplementary Information, sections 7 and 8). Whereas some more distantly related individuals (24 LEO, 47 MGS) could not be integrated into the overall pedigrees, only few (6 LEO, 39 MGS) were not genetically related to others at all, many of whom were female individuals of young reproductive age (Extended Data Fig. 3 and Supplementary Information, section 7); most likely, these were exogamous partners without children buried in the cemetery.

**Extended Data Fig. 4:**
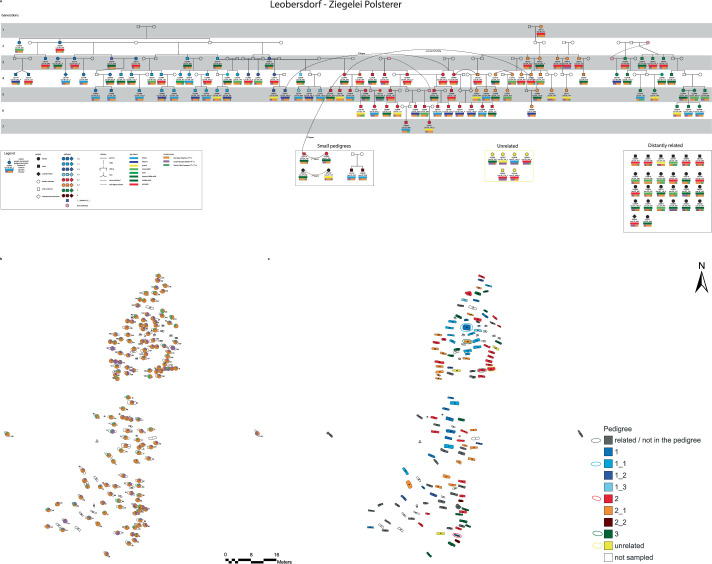
The pedigree in Leobersdorf including meta information for each individual and the cemetery map of the site. **a)** The pedigree of Leobersdorf. The grave ID, anthropologically assigned age group and genetic ancestry composition of each individual are shown underneath the icon in the pedigree. **b)** The ancestry proportions of ancient individuals on the cemetery map. **c)** The burial location of sub-pedigrees in the cemetery map.

**Extended Data Fig. 5:**
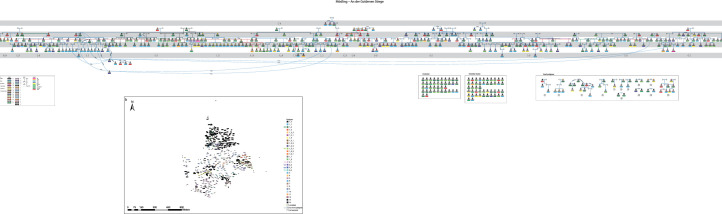
The pedigree constructed for Mödling including meta information for each individual and the cemetery map of the site. **a)** The pedigree of Mödling. The grave ID and anthropologically assigned age group of each individual is shown underneath the icon in the pedigree. **b)** The cemetery map of Mödling and burial location of sub-pedigrees in the cemetery map.

**Extended Data Fig. 6:**
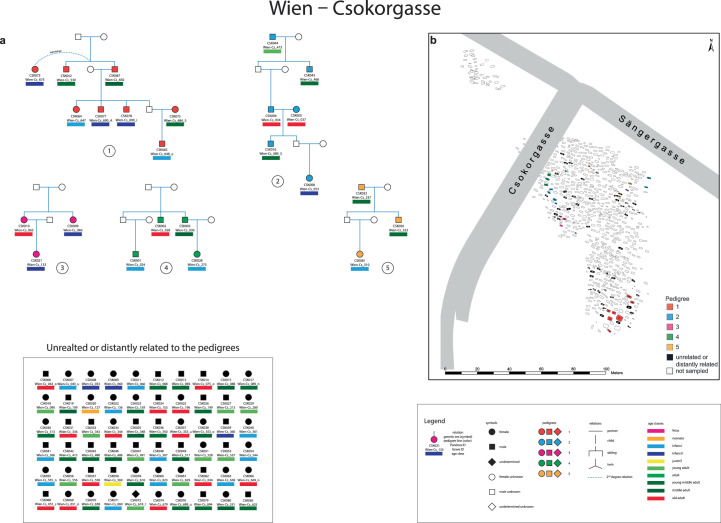
Small pedigrees constructed from related individuals in Csokorgasse and the cemetery map of the site. **a)** The reconstructed small pedigrees in Csokorgasse. **b)** The cemetery map of Csokorgasse. 10% of graves at the site were sampled. The full list of relatedness estimates from sampled individuals is reported in Supplementary Table 4. Background map in **b** adapted from BEV (https://doi.org/10.48677/ce44e0d0-bebc-4ffe-83a2-b2ca2d277be6) under a Creative Commons Licence CC BY 4.0.

Our interdisciplinary approach allowed assessing to what extent biological relatedness corresponded with social kinship. As both communities were mainly formed by biological relatives, and hardly any non-related male individuals were part of them, biological kin was a key factor of social cohesion. Archaeological data confirm the overlap between biological and social relatedness; at both sites, members of subpedigrees form visible small burial clusters, but do not use separate areas (Figs. 2 and 3 and Extended Data Figs. 4–6). Two or more individuals buried together were, in most cases, parents and children, couples or siblings, although there are a few double burials without biological relation (Supplementary Information, section 7).

**Extended Data Fig. 7:**
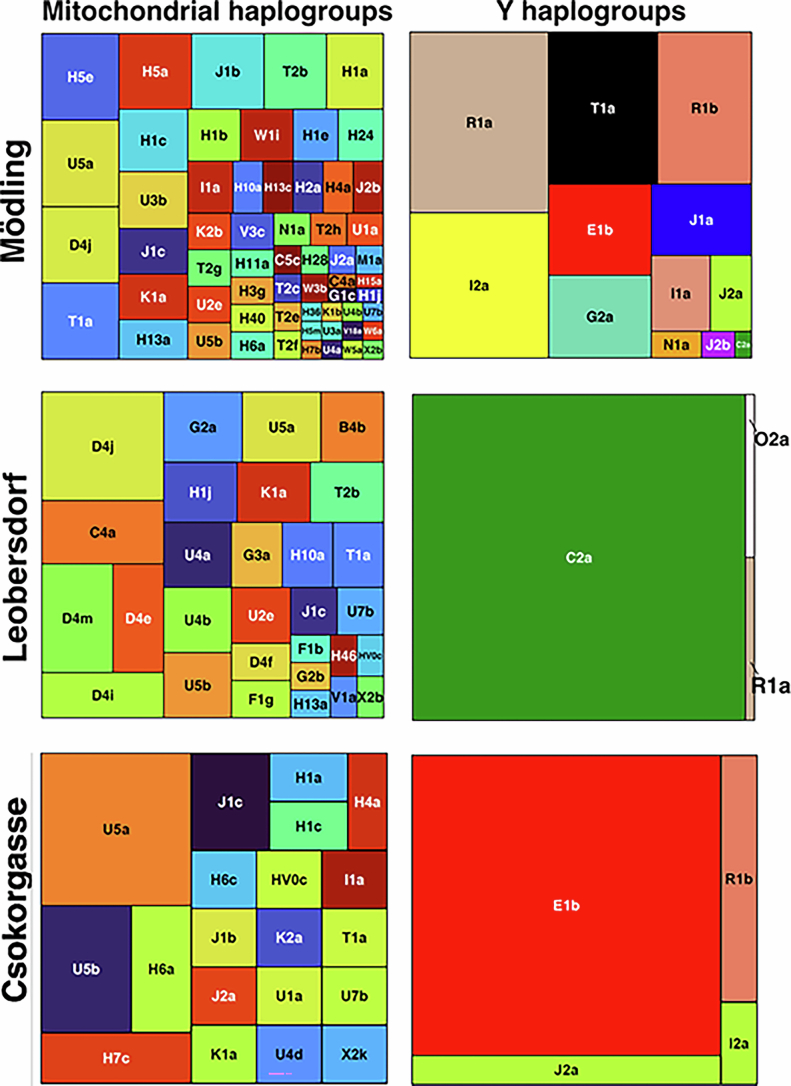
Distribution of uniparental markers in Leobersdorf, Mödling and Csokorgasse. The size of each sub-square is proportional to the frequency of corresponding haplogroup within the site. The contrast between male uniformity and female variation is striking in Leobersdorf and Csokorgasse, while there is considerable Y-Chromosome variety at Mödling. The meta information is reported in Supplementary Table 1.

Overall, the pedigrees show clear reproductive strategies, which are similar between MGS and LEO, but differ in some important respects. Given that none of the newly reported individuals carry high amounts of runs-of-homozygosity genomic regions—the indication of inbreeding—as estimated by hapROH^42^ (Methods and Supplementary Information, section 4), we infer that consanguinity was strictly avoided in both MGS and LEO across six generations (Supplementary Table 6). That was mainly achieved by exogamy: 17 of the 19 (90%) mothers buried in Leobersdorf with identifiable offspring have no ancestors buried on site; in the much larger community of Mödling, they are 46 out of 59 (78%). Many daughters seem to have left to be married elsewhere; between ages 7 and 17 years, the sex ratios of the deceased male to female individuals at LEO and MGS are about 1.5:1 and 1.7:1 respectively, and among adults, hardly any female individuals born by parents on site remain (Supplementary Information, section 7). The uniparentally inherited markers show a similar picture: 62 out of 77 male individuals in LEO derive from Y haplogroup C2b, which is prevalent in East Asia (Extended Data Fig. 7 and Supplementary Table 1). By contrast, female individuals in the Leobersdorf pedigree have diverse mitochondrial DNA (mtDNA) (Extended Data Fig. 7). Mödling is different because male individuals also display several Y haplogroup lineages—for example, frequent European types R1a and R1b (Supplementary Table 1 and Extended Data Fig. 7).

This means that patrilocality and patrilineality were dominant at both sites, if to a different extent (patrilineages account for 88% at LEO and 70% at MGS); the remaining cases did not display consistent matrilineality over more than two generations (Supplementary Information, section 7). At Rákóczifalva in the Avar heartland, patrilineality was calculated at 98% (ref. ^10^). This suggests that common social practices in the Avar realm were followed more thoroughly in its core area, whereas in the periphery more variation was possible.

**Extended Data Fig. 8:**
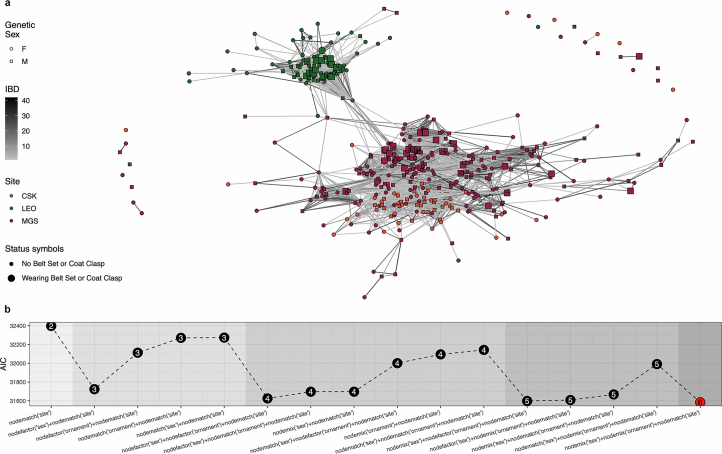
The IBD network diagram with variables statistically significantly correlated with individuals’ connectivity. **a)** The IBD network diagram. The individual-based network with edges weighted by the level of shared IBD segments (in cM). The filled color denotes site, node shape indicates genetic sex, and node size indicates status symbol. **b)** Akaike Information Criterion (AIC) value of tested models. AIC value measures the statistical correlation level between variables in each model listed on the x-axis. ‘Ornament’ on the x-axis refers to ‘Status symbol’ for short. Among all tested correlation models, ‘sex’, ‘ornament’ and ‘site’ are variables that are significantly correlated with connectivity among individuals, and hence highlighted in red color.

Furthermore, KIN and IBD analyses (Methods) showed that in spite of their spatial proximity people from Mödling and Leobersdorf were only marginally mixing with each other in the course of the roughly 150 years in which both cemeteries were contemporaneously used (Supplementary Tables 3 and 4). Only five second-degree related pairs and limited IBD connections between LEO and MGS could be attested, whereas the smaller selection of individuals from Csokorgasse shows 17 pairs of first- and second-degree relatedness (Extended Data Fig. 6) and substantial IBD connections with Mödling (Extended Data Fig. 8). LEO had more genetic connections with some sites in the DTI-Transtisza regions at a distance of almost 400 km (for example, Kunszállás)^10^ than with MGS (Fig. 1 and Supplementary Table 3).

The different genetic ancestry in LEO and MGS can therefore be explained by a similar pattern of partner selection in both communities, mostly in communities where similar ancestry dominated, perhaps at large encounters or by traditional relations between clans, as textual evidence from Central Asia suggests^43,44^. Only three male individuals (two of them brothers) at LEO had children with female individuals of prevalently European ancestry (Extended Data Fig. 4). In this way, in the course of the six generations buried on site, the East Asian ancestry remained dominant. Its per-generation average proportion stayed around 70% over six generations in subpedigree 1 (Extended Data Fig. 1), and it even increased from around 55% in subpedigree 2, reaching 70% in the final generation (Extended Data Fig. 1). Although reproductive partners with similarly high East Asian ancestry were chosen at LEO, at MGS partners with European ancestry were preferred (Extended Data Figs. 4 and 5). An analogous reproductive pattern was thus used to maintain the difference in ancestry from both sides.

**Fig. 4:**
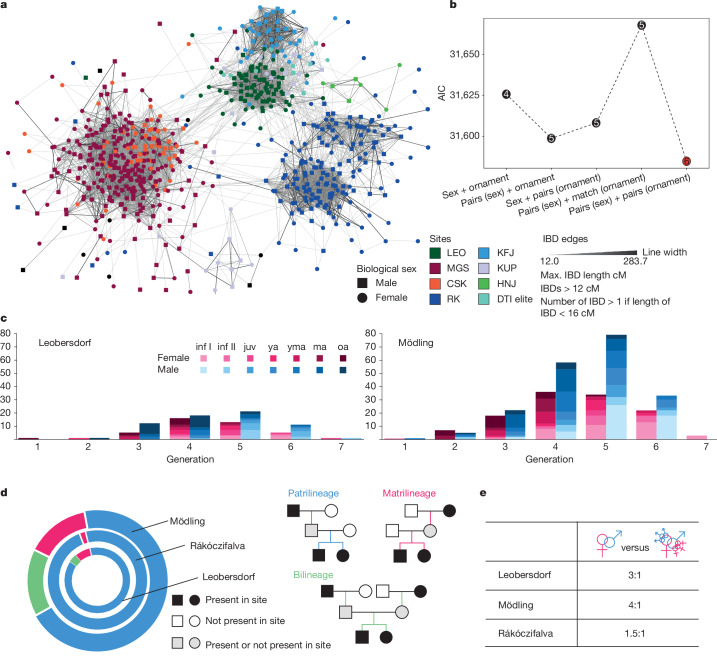
IBD network and reproductive strategy. **a**, Cultural and biological variables correlating with IBD connectivity. We show the IBD network among newly reported three sites (Leobersdorf, Mödling and Csokorgasse), as well as with published Avar-period sites in the Carpathian Basin. **b**, Archaeological and anthropological correlates of IBD connectivity. The calculated Akaike information criterion (AIC) for the connection models of archaeological and anthropological correlates with genetic relatedness are shown here (Supplementary Information, section 8d). **c**, Demographic profile per generation. We report the numbers of individuals for Leobersdorf and Mödling in each generation (age ranges are inf I, infants I 0–6 years; inf II, infants II 7–13 years; juv, juvenile 14–17 years; ya, young adult 18–25 years; yma, young–middle adult 26–35 years; ma, middle adult 36–50 years; oa, older adult 50+ years). **d**, Percentage of patri and/or matrilineages. Proportions of patrilineages, matrilineages and bilineages are calculated for three Avar-period sites (Leobersdorf, Mödling and Rákóczifalva) and examples are given for each. **e**, Single and multiple reproductive union. Ratio of single reproductive unions versus multiple reproductive unions in these three sites are calculated per site. Max., maximum.

A salient feature at both sites were multi-reproductive unions. At Leobersdorf, these were mostly male individuals (10 out of 14) who had children from more than one partner. Polygamy is attested in written sources for Avar khagans^2^ and is the most plausible explanation, although we cannot completely exclude serial monogamy. At Mödling, female individuals also had children with two or more partners almost as often as male individuals (15 out of 31). As most partners of these multi-reproductive female individuals were related to each other (brothers, half-brothers, stepsons), these were probably levirate unions, an arrangement under which a widow marries a male relative of her deceased partner (Supplementary Information, sections 1 and 7). This is a practice frequently attested for the Xiongnu and other Central Asian steppe peoples up to the present; it is intended to maintain the functionality of the clan^10,45,46^. At Leobersdorf, we have found 3 levirates and 14 multi-reproductive unions in 56 reproductive relations, at Mödling 7 levirates and 31 multi-reproductive unions in 161 reproductive relations (Supplementary Table 7 and Supplementary Information, section 7). At Mödling, levirates regard only siblings, whereas at LEO, they can involve two or even three generations: one male had children with a female in the same generation, and with a younger female who (probably later) became the partner of his grandson. The ratios of single versus several reproductive relations differ considerably between sites; they are roughly 3:1 at LEO; 4:1 at MGS and 1.5:1 at Rákóczifalva (Fig. 4, Supplementary Table 8 and Supplementary Information, section 7), so the difference in ratio to a site in the Avar core area exceeds the one between LEO and MGS. Closer to the Avar power centre, steppe traditions may have had a greater role; in this case as in others, we see a lot of local variation on the basis of a shared cultural heritage.

Given the genetic contrast between Leobersdorf and Mödling, we explored whether the ancient populations had distinct phenotypic features; the historical records mention the unusual hairstyle of Avar men^2^, but no distinctive phenotypic traits. The analysis of five phenotypic traits mostly corresponded to expectations. For the dominant skin-pigmentation gene (*SLC24A5*) causing lighter skin colour among Europeans, the allele frequency at MGS is nearly 100%, but it also reaches 50% in Leobersdorf, compared to the zero frequency in present-day East Asians (Supplementary Information, section 6). The *HERC* gene denoting eye colour is at about 50% at LEO and only roughly 15% at MGS. The *EDAR* gene encoding a shovel-shaped incisor typical for modern East Asians is present in higher allele frequency in LEO than in MGS, whereas lactase-persistence (LCT)-related alleles were more frequent at MGS^47^ (Supplementary Information, section 6). The allele of *ADH1B*, associated with facial blushing after drinking alcohol in mainland East Asians^48^, was equally low in both sites. As would be expected, there are differences (Supplementary Fig. 15); but there is no fixed visual cue that could establish a clear distinction between individuals at LEO and MGS.

**Extended Data Fig. 9:**
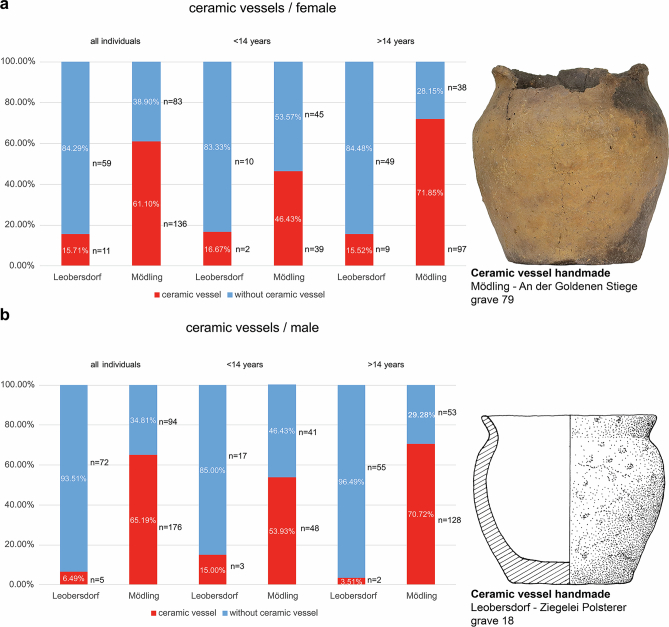
Proportion of individuals buried with ceramic vessels in Leobersdorf and Mödling, together with pictures of ceramic vessels. **a)** Proportion of female individuals buried with ceramic vessels. **b)** Proportion of male individuals buried with ceramic vessels. The two cemeteries show some contrasting patterns in the burial goods (Ceramic vessel from Mödling-An der Goldenen Stiege grave 79, photo: Katharina Richter; ceramic vessel from Leobersdorf-Ziegelei Polsterer grave 18, drawing: plate 14 from ref. ^3^).

Certain archaeological features diverge between LEO and MGS: most significantly, pottery in graves is more frequent at MGS (63.8%) than at LEO (10.9%), and often of higher quality (Supplementary Information, section 7). At MGS, a greater variety of female jewellery and more necklaces with more glass beads were found. The basic female set of earrings, necklace and spindle-whorl is found in 28% of female graves in LEO and in 63% in MGS. Overall, fewer social or gender differences can be detected at MGS. However, these were gradual differences on the basis of a shared set of cultural forms of expression such as the deposition of ceramic vessels in the graves (Extended Data Fig. 9); they do not establish any consistent cultural distinctions (Supplementary Information, section 7c).

The demographic development at LEO and MGS clarified by the pedigrees shows a similar pattern (Fig. 4): both sites were settled by a smaller number of founders with relatively scattered graves in the second half of the seventh century^3^, but not by a large group at once. Only in the third generation did stable communities dominated by large kin-groups with a structured social life and determined funerary practices emerge (Figs. 2 and 3). In the fourth and fifth generations, around the middle of the eighth century ce, both LEO and MGS reached demographic stability and a peak in numbers (Figs. 2 and 3 and Supplementary Information, section 7); 65% of the total burials belonged to generations 4 and 5. In the sixth generation, the number of inhumations declined again and this generation is mostly represented by children’s graves (Supplementary Information, section 7). Those who survived into adulthood either moved away or were buried elsewhere, probably due to the Frankish conquest in the 790s (ref. ^2^).

## IBD networks and long-distance links

We applied IBD network analysis to the large matrix of pairwise IBD connections among individuals from Avar-period sites in the Vienna and Carpathian Basins available with high quality ancient DNA (aDNA) (Fig. 4 and Supplementary Information, section 8). The most evident feature in the network is that individuals from each large site cluster primarily together. That is driven by close genetic relatedness and it is often linked to Y chromosome haplogroups (Extended Data Fig. 8). At LEO, where most male individuals share the same Y haplogroup, individuals form a single cluster; in Rákóczifalva, two successive clusters present two distinct Y haplogroups interpreted as local realignment of power^10^. MGS individuals form a single loosely connected cluster despite their diverse Y haplogroups. The selected samples from CSK, in contrast to all other sites, do not form their own cluster but are firmly set in the MGS cluster, corroborating the similar ancestry observed for CSK and MGS and suggesting intense genetic interaction between the two sites.

Further analysis of the IBD connections shows higher between-sites female relatedness (Fig. 4, Supplementary Information, section 8 and Extended Data Fig. 8), which suggests that mainly female individuals moved between patrilineal communities to be married, as in the Carpathian Basin (DTI and Transtisza regions)^10^. This pattern holds statistically significant for both LEO and MGS/CSK (Supplementary Information section 8). Within sites, therefore, we found more male–male IBD pairs, because male descendants remained there. Applying new formal regression models on the IBD networks, we were able to bring archaeological and genetic variables together in the same statistical framework (Supplementary Information, section 8). With exponential random graph models (ERGMs), we found that within- and between-site female–male pairs were 1.35-fold and male–male pairs 2.54-fold significantly more likely to be related to each other compared to female–female pairs (Supplementary Information, section 8 and Extended Data Fig. 8). When testing archaeological signs of status in such an ERGM, we found that status-related items such as belt sets for male individuals and coat clasps for female individuals were significantly related to IBD connectivity (*P* < 0.0001; Fig. 4, Extended Data Fig. 8 and Supplementary Information section 8): a presence of a prestige object for one from a pair of individuals increased the probability of an IBD link by 1.27-fold; if both carried such an object, by 2.55-fold (Supplementary Information, section 8 and Extended Data Fig. 8). Social status for both sexes can therefore be linked to a higher level of connectivity.

To investigate continuous variables (for example, geographic distance and ancestry), we applied generalized ERGMs (GERGMs) on a site-level network (Supplementary Information, section 8 and Extended Data Fig. 8). Geographical distances were not predictors for connectivity in any of the models, whereas ancestry (East Asian-like or European-like) was statistically correlated with IBD connections (IBD segments > 16 cM) (Supplementary Information, section 8). The communities with high East Asian-related ancestry were more connected to each other, in particular Leobersdorf to DTI and/or Transtisza sites (Fig. 4). That was mostly female connectivity, which helped to maintain LEO’s distinctive ancestry, whereas we found little evidence of male mobility between sites.

## Conclusion

At three archaeological sites from the seventh and eighth centuries ce at the western periphery of the Avar empire in the Vienna Basin, we revealed the strong contrast between very diverse genetic ancestry, a shared cultural habitus and similar social structure. Whereas Leobersdorf displays East Asian ancestry with minor Pontic steppe and European admixture that occurred within the last 200 years, individuals at Mödling-An der Goldenen Stiege showed a mixture of European-like ancestries that were acquired several times in the past, with only minor elements of more recent East Asian admixture. With a whole-cemetery sampling approach, the reconstruction of pedigrees of up to 356 individuals and more precise dating, we showed that the genetic barrier between the two nearby sites lasted several generations. The comprehensive analysis of pedigrees and IBD networks across sites and regions revealed that this genetic barrier was maintained by deliberate reproductive strategies of the communities that despite their different genetic ancestry kept highly similar practices of patrilineality, levirate unions and female exogamy. As IBD relations show, more genetic interactions occurred between Leobersdorf and the core region of the Avar empire than with the neighbouring sites of Mödling and Csokorgasse. The incidence of patrilineality and the number of multiple and levirate reproductive unions at Leobersdorf, well-attested among steppe populations, is lower than in the Tisza region but higher than in Mödling. This shows the range of variation of social practices in the Avar realm, which were, however, basically shared throughout different Avar communities despite their different ancestral background.

The arrival of a group of Central Asian ‘Avars’ in the Vienna Basin and in other parts of East Central Europe in the sixth to seventh century provides a highly suited example to study the impact of a major migration. East Asian ancestry was present in the Vienna Basin until the eighth century, but disappeared later, which requires future study. The admixture process with regional populations was complex: it neither followed a strictly closed model excluding reproductive unions between different groups, nor did it lead to a steady process of admixture. Instead, a variety of local or regional scenarios emerged, in which a shared cultural, political and ethnic frame of reference coexisted with local variation and considerable genetic diversity. Perceptions of people as ‘Avars’ in the texts, the unification of cultural habitus and genetic admixture did not follow analogous rhythms, and these shifts led to rather diverse genetic ancestry in different local communities.

The interaction of communities with vastly different past trajectories, with different ancestries and cultural habitus, has been a key issue in the human past as well as in the present^49^. Simple scenarios of replacement, acculturation or steady admixture are often invoked^15^. Classic population genetic models assume that reproduction was a uniform random process; recent studies have already shown that we have to allow for more regional and local variation^50^. Similar conditions could lead to rather different outcomes. This regarded, not least, kinship and reproduction; human procreation is channelled by a whole set of kinship systems, marriage rules, relations between individual people and the social meanings of birth^51,52^. Our evidence shows that choices about partnership were hardly random. Reproductive practices and patterns of behaviour shared between groups of different ancestry could paradoxically perpetuate their genetic differences.

## Methods

### Sample collection

We obtained all permissions for the work with archaeological and anthropological material in this study. We used a whole-cemetery sampling approach in which we sample every individual if skeletal preservation allows within all identified human skeletons at the studied archaeological sites (Supplementary Information, section 3). For the skeletons of Leobersdorf housed at the Department of Anthropology at the Natural History Museum Vienna, we took 155 samples out of 181 identified individuals by taking petrous bone (*n* = 120), teeth (*n* = 26) and postcranial elements (*n* = 9). For Mödling-An der Goldenen Stiege, we sampled 485 out of 536 identified individuals from the site by taking petrous bone (*n* = 315), teeth (*n* = 114) and postcranial elements (*n* = 49). We also sampled earlier phases at close-by burial groups: five individuals from Mödling-Leinerinnen and two from Mödling-Lerchengasse. For Csokorgasse, we sampled only part of the extensive cemetery of 745 graves, using 83 individuals for aDNA analyses. The bone powder was obtained in the ArcheoGen Brno sampling laboratory (Masaryk University) and Vienna sampling laboratory (University of Vienna, individuals from the site of Csokorgasse) (Supplementary Information, section 3). Considering the individuals with several types of sample (teeth, pars petrosa, bone and so on) we analysed in total 147 individuals from Leobersdorf, 492 individuals from Mödling-An der Goldenen Stiege/-Leinerinnen/-Lerchengasse and 83 individuals from Csokorgasse (Supplementary Information, section 3).

### Ancient DNA data generation

We performed DNA extraction and single-stranded DNA sequencing library preparation at the Max Planck Institute for Evolutionary Anthropology, following standardized protocols designed in Ancient DNA Core facility. We applied a partial treatment of uracil-DNA-glycosylase^53^ and 1,240,000 in-solution capture^54^ for all sampled skeletal parts.

We processed raw sequencing data through the nf-core/eager v.2.3.2 pipeline (https://nf-co.re/eager)^55^, using AdapterRemoval v.2.3.1 (ref. ^56^) for removing adapters and reads shorter than 30 bp, and bwa v.0.7.17 (ref. ^57^) for alignment (with the parameters ‘-n’ and ‘-l’ set to 0.01 and 1,024, respectively). We applied Picard MarkDuplicates v.2.22.9 function (https://github.com/broadinstitute/picard) for PCR duplicates removal, and mapDamage v.2.0 (ref. ^58^) for estimating the proportion of C-to-T taphonomic deamination at the ends of the mapped fragments on a subset of 100,000 sequencing reads passing q30 filter. We trimmed the first and last two bases of each read using trimBam model of bamUtil v.1.0.13 (ref. ^59^) for removing C>T and G>A misincorporations. We made random pseudo-haploid calls on 1,240,000 sites using pileupCaller (https://github.com/stschiff/sequenceTools), based on trimmed BAM files after quality filtering using samtools^60^ with flags -q30 -Q30. We used Schmutzi (v.0.7.12) for estimating contamination level on mitochondria^61^ and ANGSD (v.0.910) for estimating contamination on X chromosomes^62^.

We assigned genetic sex by the ratio of coverage on the X and Y chromosomes versus coverage on autosomes. We expect female individuals to have a roughly even ratio of X chromosome to autosomal coverage (X ratio of 0.8) and a Y ratio of 0, and male individuals to have roughly half the coverage on the X and Y chromosomes as autosomes (0.4). We assigned the mitochondrial haplogroup by HaploGrep2 v.2.51 (ref. ^63^) and assigned the Y chromosome haplogroup by Yhaplo v.11.249 (https://github.com/23andMe/yhaplo), using the ISOGG panel v.15.73 as a reference (https://isogg.org/tree/). We used hapROH v.0.64 (ref. ^42^) to detect genomic segments longer than four centimorgans indicating co-inheritance of identical haplotypes results in stretches of DNA that lack genetic variation.

### Biological kinship estimation

We estimated genetic relatedness among newly reported individuals using KIN^64^ (setting the contam parameter as 0) and ancIBD^65^. For running ancIBD, we imputed our dataset using GLIMPSE^66^. First, we used ATLAS^67^ for computing genotype likelihood of variant sites present in the 1,000 Genomes Phase 3 reference panel^68^, using the MLE caller and the empirical post-mortem damage pattern observed across reads, as described in https://bitbucket.org/wegmannlab/atlas/wiki. Then we ran GLIPMSE in three steps: (1) GLIMPSE_sample_static; (2) GLIMPSE_phase and (3) GLIMPSE_ligate. We ran ancIBD on imputed individuals with over 600,000 single-nucleotide polymorphisms (SNPs) covered on a 1,240,000 panel. For pairs of individuals sharing more than two long IBD segments (greater than 20 cM), we considered this pair of individuals to be related through first to sixth relatedness.

### Pedigree reconstruction

We constructed the pedigree by the estimated relatedness from KIN, using the information from age at death and assigned mitochondrial and Y haplogroups, also with the help from ancIBD for distinguishing between avuncular and grandparent–grandchild relatedness (Supplementary Information, section 4c). First, we identified first-degree related pairs, by connecting siblings first and adding their parents or offspring later. Then, we used estimated second-degree relatedness to confirm the previous core family we drew from the first step, and also to add second-degree related relatives to the parent–child or siblings. There are three types of second-degree relatedness: half-siblings, avuncular and grandparent–grandchild. We followed the principle that siblings should be equally related to half-siblings, grandparents and avuncular. For individuals with uncertainties, we listed many possibilities of pedigree building in the Supplementary Information, section 4 and present the most likely pedigree in Figs. 2 and 3.

### Genetic ancestry analysis

For downstream population genetics analyses within the Eurasia context, we merged our newly generated dataset with previously published data from the Allen Ancient DNA Resource (https://reich.hms.harvard.edu/allen-ancient-dna-resource-aadr-downloadable-genotypes-present-dayand-ancient-dna-data). For investigating the ancestry of Leobersdorf, Mödling and Csokorgasse, we performed PCA analysis using smartpca v.16000 (ref. ^69^) with option lsqproject: YES using a full list of 149 modern-day Eurasian populations for calculating principal components in the context of present-day Eurasian genetic variations. The newly reported uncontaminated ancient individuals with more than 20,000 SNPs covered in the 1,240,000 panel are projected into PCA (Fig. 1).

To examine relative differences in genetic ancestral sources of Leobersdorf individuals (Methods, Supplementary Table 2 and Supplementary Information, section 5), we tested Szólád from Hungary (Hungary_Szólád_6c)^19^ and Sarmatian period from Kazakhstan (Kazakhstan_Sarmatian_IA)^70^ as the possible pre-Avar European ancestry source, following the strategy of using temporally close ancient populations as possible sources. Given the Rouran-origin hypothesis in historical records, we used AR_Xianbei_P_2c (ref. ^17^) as the possible East Asian ancestry source, which is an ancient group preceding the Rouran in and around modern Mongolia. We tested Alan-period individuals (North_Caucasus_7c)^18^ (an Iron Age nomadic population from the North Caucasus) as the proxy for the Pontic steppe source.

To characterize the ancestry of the Mödling individuals (Supplementary Information, section 6) in the context of present-day European genetic diversity, we compiled a modern-day reference dataset including 12,176 modern individuals sampled from 49 European and West Asian populations^25,71–84^. For PCA analyses, we calculated principal components on the basis of 426,135 autosomal SNPs using a list of 36 present-day European populations, and subsequently projected ancient individuals with option lsqproject: YES and shrinkmode: YES.

For genetic ancestry modelling, we used qpAdm v.810 and qpWave v.410 in the admixtools v.5.1 package^69^. For Leobersdorf individuals (Supplementary Information, section 5a), we used a list of 11 populations as outgroup to pull out each distinct ancestry in western and eastern Eurasia (Mbuti.DG, Anatolia_N,Levant_N, Iran_N,Villabrua, Onge.DG, Mixe.DG, DevilsCave_N.SG, MA1, Kolyma_M.SG, YR_MN). For Mödling individuals (Supplementary Information, section 6a), we used a list of 11 populations as the outgroup to pull out each distinct ancestry (Mbuti.DG, Anatolia_N, Levant_N, Iran_N, EHG, Iron_Gates_HG, Onge.DG, Ami.DG, Mixe.DG, DevilsCave_N.SG, Russia_Bolshoy).

### Integrating genetics with archaeological, anthropological and historical data

The interdisciplinary approach used in this study has allowed us to reconstruct biological, cultural and social structures of ancient peoples. Therefore, we (re-)examined anthropological and archaeological features of 639 sampled individuals (Supplementary Information, section 3) and newly generated 94 radiocarbon dates for pedigree building (for LEO, MGS, MLS, MLE, Supplementary Information, section 3). We (re-)estimated age at death, preservation degrees, number and allocation of individuals per each of the four aforementioned sites (Supplementary Information, section 4b). Integrating this information with genetic data, we were able to distinguish parents from their children in cases in which the genealogical position of the individuals was unclear, increasing the number of people represented in the pedigrees. We were also able to resolve unclear contexts in many burials in LEO (that had been anthropologically analysed in 1987, in part with methods outdated today) preventing false information in pedigree construction and thus in biocultural inferences.

A total of 641 graves with 717 burials from the sites of Leobersdorf-Ziegelei Polsterer and Mödling-An der Goldenen Stiege were also re-evaluated regarding the orientation of graves, volume of the grave pits, distribution, frequency and culture-historical meaning of the buried jewellery and funeral offerings. In this way, 15,173 individual items were classified, contextualized and taken into account for the final interpretation. Because the rich archaeological record of the late-Avar period offers a rather elaborate dating grid for a number of objects, their distribution in the graves was related in numerous ways to the genetic and anthropological data (Supplementary Information, section 8). Positions in the pedigree, age at death, ^14^C dates and dating by object typology allowed us to establish a fine-scale chronology and explain the demography of the site. Information on the types of object in the graves or on the size of the grave pit was compared with the ancestry, the position in the pedigree and the number of IBD relations of each individual. To identify variables corresponding with a significant increase in genetic relatedness, we applied ERGMs and GERGMs in the context of an IBD network (Supplementary Information, section 8d). We used the ergm package within the Statnet suite of packages (https://github.com/statnet/ergm). For GERGM analyses, we used the GERGM package^85^. While fitting all additive models, we retained models with minimum Akaike information criterion and evaluated significance by using *Z*-tests of non-zero coefficients integral to the analysis.

Although the written sources do not contain direct information about the sites and say little about the Vienna Basin in the eighth century ce, historical data were integrated into this approach in several ways. There are several texts from the period that contain relevant information about the Carpathian Basin, its neighbouring regions and about early medieval steppe societies in general. The historical disciplines have developed a set of methods of source critique, textual analysis and contextualization that allow assessing the most probable interpretation of this information. Most importantly, historical knowledge about the Avar realm and its temporal and spatial environment has enabled us to formulate pertinent research questions, exclude hypotheses and develop possible historical interpretations that could be tested against the scientific data. Historical information contributed to the creation of an integrated chronology of the sites and of the overarching developments reflected in them. An issue in which historical evidence also proved useful was the reconstruction of structures of relatedness and reproductive practices, for instance, to connect pedigree information about female individuals having children from two or more male individuals closely related to each other with the practice of levirate known from contemporary texts.

The archaeogenomics of historical periods is a relatively new field, and the historical interpretation of the data poses challenges, but also offers many new opportunities. Much effort in the preparation of the present paper went into developing and testing an interdisciplinary workflow and into integrating various disciplinary methods. The results were then embedded within historically plausible time frames. The critical integration of multidisciplinary results renders a plasticity to past lives that neither the archaeological traces of individual remains nor the pedigrees in themselves can disclose.

### Reporting summary

Further information on research design is available in the Nature Portfolio Reporting Summary linked to this article.

## Online content

Any methods, additional references, Nature Portfolio reporting summaries, source data, extended data, supplementary information, acknowledgements, peer review information; details of author contributions and competing interests; and statements of data and code availability are available at 10.1038/s41586-024-08418-5.

## Supplementary information

Supplementary Information Supplementary sections 1–9 (including 53 figures); see Contents for details.

Reporting Summary

Supplementary Tables Supplementary Tables 1–8 and a Table guide.

## Data Availability

The newly produced sequence data is deposited in the European Nucleotide Archive (ENA) with the following accession number: PRJEB76548. The new haploid genotype data is available through the Poseidon framework under: https://github.com/poseidon-framework/community-archive/tree/master/2024_Wang_ViennaBasinAvarPeriod. The previously reported ancient DNA datasets used in this study are available in Allen Ancient DNA Resource v.54.1 (https://reich.hms.harvard.edu/allen-ancient-dna-resource-aadr-downloadable-genotypes-presentday-and-ancient-dna-data). The dataset used for ^14^C radiocarbon date calibration is IntCal20 (http://intcal.org). The reference panel used for the Y haplogroup assignment is ISOGG v.15.73 (https://isogg.org/tree/). The Genome Reference Consortium Human Build 37 (GRCh37) is available through the National Center for Biotechnology Information under accession number PRJNA31257. The revised Cambridge reference sequence is available through the National Center for Biotechnology Information under NCBI Reference Sequence NC_012920.1. Published genotype data for the present-day British sample are available from the Wellcome Trust Case Control Consortium (WTCCC) through the European Genotype Archive (https://www.ebi.ac.uk/ega/) under accession number EGAD00010000634. Published genotype data for the present-day Irish sample are available from the WTCCC through the European Genotype Archive under accession number EGAD00010000124. Published genotype data for the rest of the present-day European samples are available from the WTCCC through the European Genotype Archive under accession number EGAD00000000120. Published genotype data for the Dutch samples are available by the GoNL request process from The Genome of the Netherlands Data Access Committee (DAC) (https://www.nlgenome.nl).

## Code availability

All relevant code and software applied in this work have been acknowledged and corresponding citations are provided in the Methods section: nf-core/eager (v.2.3.2) (https://nf-co.re/eager), AdapterRemoval (v.2.3.1), bwa (v.0.7.17), MarkDuplicates (v.2.22.9) (https://github.com/broadinstitute/picard), mapDamage (v.2.0), bamUtil (v.1.0.13), Schmutzi (v.0.7.12), ANGSD (v.0.910), samtools (v.1.9), HaploGrep2 (v.2.51), hapROH (v.0.64) (https://py.org/project/hapROH/), ancIBD (v.0.5), smartpca (v.16000), EIGENSOFT (v.6.0.1), admixtools (v.5.1), qpWave (v.410), qpAdm (v.810), GLIMPSE (v.1.0.0), ATLAS ((https://bitbucket.org/wegmannlab/atlas/), DATES (v.753), ADMIXTURE (v.1.3.0), KIN (https://github.com/DivyaratanPopli/Kinship_Inference), BREADR (https://github.com/jonotuke/BREADR), pileupCaller (https://github.com/stschiff/sequenceTools), yHaplo (v.11.249) (https://github.com/23andMe/yhaplo), Y-Lineage-Tracker, pathPhynder, SPSS (v.23), OxCal, DataGraph. Scripts for ERGMs and GERGMs in R (v.4.2.0) can be found at GitHub (github.com/BenRohrlach/avarNetworkStudy) and at Zenodo (10.5281/zenodo.13889079)^86^.
