# Supplementary material for: Ancient DNA reveals reproductive barrier despite shared Avar-period culture
Source: Nature. 2025 Jan 15;638(8052):1007–14. doi: 10.1038/s41586-024-08418-5 (PMC11864967; doi:10.1038/s41586-024-08418-5)
Supplement: Supplementary file 1 — Supplementary sections 1–9 (including 53 figures); see Contents for details. [file 41586_2024_8418_MOESM1_ESM.pdf]

nature portfolio

https://doi.org/10.1038/s41586-024-08418-5

Supplementary information

Ancient DNA reveals reproductive barrier despite shared Avar-period culture

In the format provided by the
authors and unedited

Supplementary Information

1. Preliminary remarks, terminology and abbreviations
2. Historical background and synthesis
    a. Population history in and around the Vienna Basin before the Avar period
    b. The Avars and their impact
    c. Ethnicity, ancestry, culture: methodological and terminological approaches
    d. Relatedness, kinship and the status of women – historical observations
    e. Towards an integrated history of Avar-period communities in the Vienna Basin
3. Archaeological overview, sampling and laboratory strategy and chronology
    a. Site selection criteria
    b. Description of the sites: Leobersdorf-Ziegelei Polsterer, Mödling-An der Goldenen Stiege, Mödling-Lerchengasse, Mödling-Leinerinnen, Wien-Csokorgasse
    c. Sampling strategy of the human remains
    d. Sampling process
    e. aDNA laboratory analysis
    f. Chronology by ^{14}C dating
4. Archaeological, anthropological and genetic methods
    a. Archaeological approaches
    b. Anthropological methods
        i. Sex and age at death
        ii. Anthropological evaluation of the pedigrees, distantly and unrelated groups
    c. Methods of pedigree construction
        i. General pedigree construction
            a. Genetically identical individuals and twins
        ii. Pedigree validation
        iii. Exceptional cases
        iv. Division into sub-pedigrees
        v. Small pedigrees in Mödling and Csokorgasse
        vi. Run of Homozygosity
5. East Asian ancestry in the Avar realm and its evolution over time
    a. Ancestry modeling strategy with proximal sources
    b. Distribution of each ancestry component in the Leobersdorf pedigree
    c. Sex bias and admixture date
6. Genetic origins of Mödling population
    a. qpAdm using proximal and distal sources
    b. The estimated admixture date between different ancestries in Mödling
    c. PCA and supervised ADMIXTURE analyses
    d. Contrasting patterns in the Leobersdorf and Mödling cemeteries
        i. Ancestry difference
        ii. Phenotypic marker difference
        iii. Uniparental marker difference
    e. Potential sex bias in the gene flow into Mödling
    f. Genetic similarity between Mödling and Csokorgasse
7. Integrating anthropology, genetics, archaeology and history
    a. Anthropological characteristics of the pedigrees
        i. The configuration of the pedigrees, age at death, sex, sex ratios and generations for Leobersdorf and Mödling

ii. Age at death–-
iii. Sex distribution based on genetic data
iv. Male to female ratios in the Vienna basin pedigrees considering all other groups
v. General features of the generations as depicted by the pedigrees
1. Age at death distribution between the generations
2. Sex distribution among adults and subadults across the generations

b. Reproductive strategies in Leobersdorf and Mödling in comparison to Rákóczifalva
i. Patrilinearity, matrilinearity and bilinearity
ii. Single and multiple reproductive unions
iii. Are more males than females in multi-reproductive unions?
iv. Is the proportion of multiple reproductive unions stable throughout the generations in Leobersdorf and Mödling?
v. Special cases of multiple reproductive unions: levirates and sororates
vi. Are the numbers of children per sex and age group comparable between the Vienna Basin sites?

c. Indicators of regional burial rites, social status and gender
i. Orientations of graves in the cemeteries
ii. Grave volume
iii. Single, double and multiple burials and multiple-use graves
iv. Individuals in multiple burials and multiple-use burials
v. Kinship-oriented cemetery development
vi. Grave goods
vii. Differences in the use of offerings
1. Ceramic vessels
2. Necklaces

viii. Prestige and social status
1. Belts
2. Belt sets
3. Close combat weapons (sword, sabre, seax, axe, lance)
4. Evidences of injuries
5. Coat clasps

ix. Gender
1. Earrings
2. Spindle whorls
3. Combination of earrings, necklaces and spindle whorls
4. Females – Item combinations
5. Males – Item combinations
6. Age related associations grouped by sex

d. Long distance connections
i. Female mobility
1. Pedigrees and linearity - mothers and non-mothers
2. Distantly related - largely young adult females
3. Unrelated - largely females
4. Mobile sisters on site
5. Widows in the Vienna Basin

8. IBD Network analysis
a. General pattern
b. Intra- and interregional pattern of connectivity
c. Male and female connectivity

d. Regression on IBD Networks
i. ERGMs
ii. GERMs
9. References

1. Preliminary remarks, terminology and abbreviations

At the outset, it is important to clarify some terms used in this study. As we, for example, follow genetic usage of terms even where they do not correspond to their uses in the humanities and social sciences, they may create some confusion among the general public. The terms and their definitions used herein are listed alphabetically.

‘Ancestry’ denotes the genetic similarity between a cluster of people under study and similar populations that had lived before them, who may very well not have been actual ancestors, but shared a common genetic background [1].

‘Ancestry decomposition’ refers to the process of finding the most likely source populations as ancestors for the target population employing statistical approaches designed for genomic data. This allows for multiple sources or source populations genetically similar to the target population instead of only the actual ancestral populations.

‘Barbarians’ was a term for non-Greeks or non-Romans used in Antiquity for people who did not share classical civilization. It could be used in a derogatory, but also descriptive sense. Sometimes it was also used by ‘barbarians’ for themselves. For lack of a better word to cover these various populations, scholars in the historical disciplines currently use it as a descriptive term for groups living or originating outside the (former) Roman Empire.

‘Bilinearity’ (from both lines) refers to a reproductive union where both the male and the female are known through genetic analyses to have had 1st to 2nd degree relatives belonging to the previous generations buried in the same cemetery.

‘Boy’ refers to the genetic sex of a male who died younger than 18 years, that is, as infans I, infans II or juvenile.

‘Carpathian Basin’ refers to the lowlands along the Middle Danube surrounded by the Carpathian mountains in the east and north, by the edges of the Alps and the hilly countries of Styria and Croatia in the West, and by the hills of Serbia south of the Sava and Danube rivers. This largely corresponds to the region of late Avar settlement as highlighted in Fig. 1.

‘Consanguinity’ in the genetic sense used here describes reproductive unions between relatively closely related individuals. In Social Anthropology, the term is often used in a much wider sense, denoting relatedness in general.

‘CSK’ refers to the Avar-period cemetery in Wien-Csokorgasse.

‘Main pedigree’ refers to the main pedigrees at Leobersdorf and Mödling constructed mainly on the basis of 1st- or 2nd-degree relations, and thus distinguished from the small pedigrees related to the main pedigree only by 3rd- to 6th-degree relations if at all. The main pedigree is divided into sub-pedigrees and is represented in different colors in Fig. 2-3.

'Distantly related' are individuals who through genetic analyses are known to be only related in the 3rd to the 6th degree to those depicted in the main pedigree.

'DTI' refers to Danube-Tisza Interfluve.

'ERGM' refers to Exponential Random Graph Models.

'Ethnic/ethnicity' Ethnicity is a relational mode of distinguishing between medium-to-large, named social groups, to which an inborn ethnic identity and common origin are ascribed. In the period, such ascriptions are often preserved in texts written by outsiders. If these ascriptions are consistent, they may have corresponded to self-identifications, and the respective ethnic groups could acquire shared agency and stable cohesion. Some scholars still use 'ethnicity' to denote actual common biological origin, and many of them use this definition to argue that ethnicity did not matter in the period under survey in this article. As an a priori assumption, this biological definition of ethnicity can be falsified, not least, in the present study.

'Exogamy/exogamous' A community is exogamous if its members as a rule choose reproductive partners from outside the community.

'Female' refers to individuals of the female genetic sex of all ages.

'Gender' refers to the social construction of characteristics of women, girls, men and boys. Here, for example, grave goods are taken as proxies for gender.

'Genetically identical' is here used for a pair of individuals for which the proportion of non-matching alleles is below the threshold defined in methods for estimating the biological relatedness.

'Generation' refers to the mean time interval between successive generations. We refer here to biological generations, for population genetic analysis we assume that they are not overlapping.

'GERM' refers to Generalised Exponential Random Graph Models.

'Girl' refers to the genetic sex of a female who died younger than 18 years, that is anthropologically characterized, as infans I, infans II or juvenile.

'Heterozygosity' refers to having two different forms of an allele, one inherited from each parent.

'Homozygosity' refers to possessing two identical forms of a particular allele.

'Hungary_Szólád_6c' refers to a group of 20 individuals found in Szólád site of Hungary [2].

'Hungary_Szólád_south' refers to a subset of 8 individuals in the Hungary_Szólád_6c group, who exhibited ancestry closer to southern Europeans from the genetic point of view [2].

'Identity' describes a reciprocal relation between individuals and a community, in which individuals place themselves within or with regard to social groups or models, and social groups define themselves in a process of communication with their acknowledged or prospective members and with outsiders. This process of identification simultaneously creates relations of alterity [3].

'Identity by descent' (IBD) does not conform to humanities usage of the term 'identity', but is used here for shared sections of the genome that were inherited from a common ancestor.

'Infans I' is an individual who died younger than 7 years of age.

'Infans II' is an individual who died between 7 and 13 years of age.

'Inferred individuals' are those individuals known to have lived through the genetic analyses of their offspring present in the pedigree. The inferred individuals are shown in the pedigree through empty squares (for males) and empty circles (for females).

'Juvenile' is an individual who died between 14 and younger than 18 years of age.

'Khagan' is a Central Asian supreme ruler's title, also used by the European Avars.

'Kinship' here exclusively refers to the social and cultural ways in which relatedness is perceived, conceptualized and put into practice. The genetic links between individuals explored in the present study are not described as 'kinship', 'family' or in similar terms, but only as (biological/genetic) relatedness. As "relatedness" is increasingly being used in social anthropology for social relations in order to avoid the term 'kinship' with its unwelcome overtones, it also occurs in its social meaning here. One methodological principle in this study is to distinguish between 'biological' and 'social' relatedness, in order to find out in which cases they overlap.

'Levirate' is a custom according to which a widow should, or in rare cases, must, marry her dead husband's brother. The "brother" may be a biological sibling of the deceased or a person who is socially classified as such. It may also be an uncle or step-father. The levirate can co-occur with the sororate, a practice in which a widower should or must marry his dead wife's sister.

'LEO' is short for the cemetery at Leobersdorf, located at Leobersdorf-Ziegelei Polsterer.

'Leo-ZP' is the abbreviation of the archaeological identifier (plus grave number) at Leobersdorf.

'LEO001', `LEO002' etc. refer to a certain lab ID number associated with the cemetery Leobersdorf - it does not match with the burial number.

'Male' refers to individuals of the male genetic sex of all ages.

'Man' refers to the genetic sex of a male died at age over 18 years old.

'Matrilineality' ('in the mother´s line') refers to a reproductive union where the female is known through genetic analyses to have had 1st to 2nd degree relatives buried in the same cemetery, while the male shows no relatives in this cemetery, see Fig 4.

'Method' is used here in the narrow sense of a specific analytical method applied on the data in the natural sciences, and not in the more general sense of an integrated approach to the evidence used in the humanities.

'Middle adult' describes an individual that died between 36 and 50 years of age.

'MGS' is an abbreviation for individuals from the cemetery called Mödling-An der Goldenen Stiege.

'Mödling' refers to the cemetery Mödling-An der Goldenen Stiege.

'Mödling sites' or Mödling cemeteries refers to Mödling-An der Goldenen Stiege, Mödling-Lerchengasse, and Mödling-Leinerinnen.

'Moe-GST' is the abbreviation of the archaeological identifier (plus grave number) at Mödling-An der Goldenen Stiege.

'MLE_6thCE' or **`MLE'** refers to Mödling-Leinerinnen.

'MLS_4thCE' or **`MLS'** refers to Mödling-Lerchengasse.

'MGS001', `MGS002' etc. refer to a certain lab ID number associated with an individual from the cemetery Mödling-An der Goldenen Stiege and Mödling-Lerchengasse; it does not match with the burial number.

'Multi-reproductive union' refers to a group of individuals including one individual mating with multiple partners. Here in the text, we mention three forms: polygamy, polyandry and polygyny. Polyandry and polygyny are forms of polygamy. We apply polyandry to cases where a female can be shown to have had more than one productive partner, and polygyny to cases where a man has children with multiple partners. Both polyandry and polygyny can also be understood as serial monogamy.

'Neonate' is used to refer to an individual who died around birth.

'**Old adult'** refers to an individual who died older than 50 years of age.

'Pannonia' was a Roman province that stretched along the Danube from the Vienna Woods to the mouth of the Save River; the term was still used after the dissolution of the Western Roman Empire, with more uncertain frontiers.

'Patrilineality' ('in the father´s line') refers to a reproductive lineage where the male is known through genetic analyses to have had 1st to 2nd degree relatives buried in the same cemetery, while the female shows no relatives in this cemetery, see Fig 4.

'Patrilocality' refers to the custom of residing with the local group of the male of the reproductive union.

'PCA' refers to Principal component analysis.

'Pedigree' is the visualization of genetic relatedness and descendancy across several generations used in this study. We have no pedigrees evidenced by people of the region in the period which could give us clues as to how they perceived their kin, and therefore cannot compare the two.

'Pontic steppes' are the steppes north of the Black Sea and the Caucasus Range, in modern Ukraine and Southern Russia.

'Present individuals' are those excavated at the cemetery, analyzed anthropologically and genetically and depicted as full squares (for males) and full circles (for females) in the pedigrees.

'Reproductive age' refers to females of estimated age of 18 to 45 years and to males aged 18 or more years.

'Reproductive union' refers to a female and a male who are known through genetic analyses to have had an offspring. We do not name them "couple" or "parents", since we are not sure what the social connotation of their union was, instead we use 'partners' as they have been sexual partners at some point in their life though that does not imply consent.

'Separate/small pedigrees' are those reproductive unions with offspring that are related to the individuals of the main pedigree in the 3rd to the 6th generation.

'Sex' refers to the sex attributed to an individual through genetic analyses though biological sex via anthropological assessment is also performed.

'Sex ratio' represents the comparison of the numbers of males and females, indicating their relation to each other. Herein, the proportion of male individuals was set to one, while the female proportion is represented by the second number. For example, a sex ratio of 1:0.7 means that for each male there are 0.7 females or 10 males for 7 females.

'Single in multiple reproductive unions' refers to an individual who has offspring only with one other individual, while this partner also has offspring with a second, third or fourth reproductive partner.

'Small pedigrees' are those depicted under the main pedigrees and represent individual reproductive unions and/or reduced networks of siblings, parents and reproductive unions and their offspring, who are related to the main pedigree in 3rd- to 6th- degree relations or not related at all.

'Sororate' refers to a social institution where a male widower marries the sister of his deceased wife, analogous to the levirate entered by a widow.

'Sub-pedigrees' are parts of the main pedigree of a given cemetery and are represented in different colors in Fig 2-3.

'Stable population' is used here for a situation in which a burial community is characterized by a similar number of males and females (roughly a sex ratio of 1:1) and a high infant mortality (individuals who died until the age of 13 - that is, as infans I and II), as shown by the pedigree.

'TT' refers to Transtisza

'Unrelated' designates individuals who are not related within the first six degrees.

'Vienna Basin' is the plain extending southward from Vienna to the east of the Vienna Woods and the Eastern Alps, and to the west of the Leithagebirge, in eastern Lower Austria.

'Women' refers to individuals of the female genetic sex that died at 18 years or older, particularly in reference to the socially constructed female gender.

'Widows' or potential widows are defined for the pedigree as females present in the cemetery and in the pedigree, who had established reproductive unions with present males, had offspring and who seemed to have survived their partners for more than 20 years (to allow for more accurate interpretations).

'Young adult' means an individual that died between 18 and 25 years of age.

'Young middle adult' means an individual that died between 26 and 35 years of age.

2. Historical background and synthesis

The following section does not only provide some historical background, but also seeks to integrate our results in an extended narrative. This integration allows us to overcome differences between Humanities and Natural Sciences in terms of manuscript length and hopefully increases readability for readers from all disciplines.

a. Population history in and around the Vienna Basin before the Avar period

Under Emperor Augustus, around the turn of the era, the regions south/west of the Middle Danube became part of the Roman Empire, and the Danube remained its frontier for more than 400 years. The province of Pannonia covered a wide stretch of land from the Vienna Woods to the river Sava and its mouth near Belgrade. Both the Danube and the road along its southern bank were one of the main east-west connections in the empire, protected by a chain of larger and smaller towns and forts. In the fourth century, the investments into an increasing presence of strong army units along the river alimented an age of prosperity, which has left rich archaeological traces in the Vienna Basin: for instance, in the impressive remains of the city of Carnuntum east of Vienna or the splendid Roman villas in its surroundings. North of the river, the Germanic Quadi and Marcomanni lived, familiar neighbors who only occasionally crossed the river in search of booty.

The increasing opportunities for Germanic warriors in Roman service, the intensification of inner conflict in the Roman Empire, and the arrival of the Huns in the area around 400 soon ended this prosperity, and an age of uncertainty began. Entire warrior groups under their warlords entered Roman territory in order to exploit the situation, whether in Roman service or through blackmail and raiding. Marcomanni moved to the Vienna Basin, and began to be called Suebi with an old name that also covered the Quadi in what is now western Slovakia, and the Alamanni who lived further west along the Middle and Upper Rhine. When the Huns built their power center in the Carpathian Basin, between Danube and Tisza, in the first half of the 5th century, Roman control of Pannonia crumbled. In the 430s/440s, King Attila became the master not only of his Huns, but also of many other peoples in the region, Goths, Gepids, Heruls, Sarmatians, Suebi and others. In spite of his great exploits and of military expeditions ranging between Constantinople, Gaul and Italy, Hunnic rule remained an episode; after Attila's death in 454, the Hun core group disintegrated and mostly withdrew from the Middle Danube region. Rome never recovered the bulk of Pannonia, which became a node on the way to glory for several 'barbarian' warrior groups. The Ostrogoths ruled there until 473, when they left for more ambitious goals under their young king Theodoric, who would later become 'the Great' as king of an Ostrogothic realm in Italy. Heruls and Suebi seem to have controlled parts of Pannonia after the Goths had left, but we know little about the fates of the Vienna Basin in the period. West of the Vienna Woods, a decreasing number of Roman forts held out by arrangement with the Rugi north of the river until 487/88, when the last Roman army ever to operate in this region evacuated them to Italy on the orders of King Odoacer, who had overthrown the last Western Roman emperor in 476. The archaeological traces can hardly be attributed to any of the peoples attested in the area at the time. Some groups of the provincial population may have stayed on but have left little trace; Roman life in the forts along the Danube had mostly ceased.

At the end of the 5th century, the Longobards appeared west and soon also east of the Vienna Woods and gradually expanded across most of Pannonia. Cemeteries from the first and second third of the 6th century attest to the presence of well-equipped warriors and their families in many parts of Pannonia and also north-west of it; these traces are, however, less frequent in the Vienna Basin than west of the Vienna Woods and further east. The Longobards built a kingdom that eventually came into conflict with the Heruls, whom

they expelled, and with the Gepids in the eastern half of the Carpathian Basin. They also established contacts with the East Romans, who continued to rule the province of Illyricum to the south of Pannonia, and to the Franks in the west, who gained control of the Eastern Alps. In the long and destructive Gothic War (535-554), East Rome regained control of Italy, but also overstretched its forces. In 567, the Longobards destroyed the Gepid kingdom; in 568, they left for Italy, where they built their kingdom, although they never managed to conquer the entire peninsula. With them, as one report has it, numerous groups deserted the Carpathian Basin: Gepids, Suebi, Sarmatians, Bulgars and Pannonians. From the written sources, we would conclude that most parts of the – already thin – population of the Vienna Basin and its neighboring regions had disappeared after the Longobards had gone.

b. The Avars and their impact

On the basis of the numerous results of this study and the two previous ones[4,5], it has become possible to fill many gaps in our knowledge about migrations, demographic changes and conditions of life in the Carpathian Basin. Further studies will bring yet more information, but we can already sketch the most probable scenario of the population history of the late 6th to 8th centuries in the Avar empire and the Vienna Basin. In particular, we have already learnt much about the migration of a large group from the Central Asian steppes whom the texts from the period call Avars, and about the long-term changes that their arrival triggered among themselves and the remaining population of the region.

As we know from the texts, these Avars had arrived north of the Caucasus in 557, subdued other groups of the Pontic steppe, and then moved on to the Carpathian Basin in 567/68. On the basis of the textual record, historians have long assumed that these Avars came from the territory of the Rouran Empire, which had been defeated by the Turks in 552 and had collapsed in the following years[6]. It also seems likely that the Avar ruling group led by a khagan (a Central Asian supreme ruler's title) Baian came from the dynasty of the Rouran khagans. In 2022, Gnecchi-Ruscone et al.[4] have shown that the Avar elite group had actually come from Eastern Central Asia, with an ancestry pointing even farther northeast. This considerable genetic difference provides an extraordinary opportunity to trace processes of admixture and the impact of migrations, supported by a rich historical and archaeological record.

We can already confirm that the Avar move to Europe was not simply a march of a male military force, but a considerable number of women must have come along, although their number may well have been inferior to that of the men. From the time range of past admixture calculated from the individuals buried in Rákóczifalva[5] and Leobersdorf (see Supplementary Fig. 1), we can deduce that reproductive unions of Eastern Asians with individuals from the Pontic steppes (prevalent at Rákóczifalva) and inhabitants of the Carpathian Basin began upon the arrival of the Avar core group. In some cases, groups from the Central Asian steppes that had arrived in Eastern Europe before the Avars and mainly settled in the Pontic steppes also seem to have been involved in admixture with inhabitants. This may have regarded the Huns who arrived in 375/400, Bulgars who are mentioned at the Lower Danube since before 500, and Turkic peoples (Ogurs, Onogurs, Kutrigurs, Utigurs, Sabirs) whose arrival in the Pontic/Caucasian steppes is reported for the year 463.

The population that the Avars encountered in the Carpathian Basin was rather varied, as we can deduct from the historical sources, the archaeological evidence, but also from a growing number of genetic studies[2,7]. In Pannonia, west of the Danube, the descendants of the population of the Roman province persisted, albeit at a reduced scale. The late Roman population of the 4th century had already been mixed in itself, including

groups that had arrived from Mediterranean countries. In the present study, this part of the population may explain the part of the Mödling ancestry defined as 'western Asian' or 'Anatolian' (Extended Data Fig.1), which is also one of the main proxies we have for groups living in South Eastern Europe in the Roman period, who probably were of eastern Mediterranean origin. Many of the inhabitants of Roman Pannonia had left the region in the fifth and sixth centuries, but others stayed behind, especially in southern Pannonia and around Lake Balaton, while most seem to have deserted the Vienna Basin and the adjacent regions to the west. Suebi, Heruls, Longobards and others had all left the region after a few decades. Some groups of this mixed migration-age population remained (or even arrived) after 568 in the southern parts of Pannonia. Still, the descendants of the late-Roman provincials played some part in the ancestry of the inhabitants of the Mödling site, while the late/post-Roman 'barbarians' had less of a persistent impact (Extended Data Fig.1). Most of them probably had come to Mödling from the central parts of the Avar realm in the 7th century; but the IBD connection between the small 5th- and 6th-century cemeteries in Mödling and the Avar-period population at Mödling-An der Goldenen Stiege indicates some loose local continuity.

A further element of the Avar-period population of the Carpathian Basin were Slavs. They are mentioned by several contemporary authors, in the 6th century along the Lower Danube, in the Balkan provinces, in the Carpathian Basin, and in Avar armies, and from c. 600 also in the Eastern Alps and the Northwest Balkans[6]. Yet they have left little specific archaeological evidence because they mostly cremated their dead, deposed no grave goods, used simple pottery and lived in sunken huts that hardly serve as a sure indicator of the presence of 'Slavs'. Cremation also means that genetic evidence is hardly available for the groups that practiced it in regions where Slavs are mentioned before 800. Cremation burials, simple pottery and sunken huts are defined by archaeologists as Prague-Korchak culture and connected with the Slavs; in many cases, that is very likely, but it remains too unspecific as a sure marker of ethnic identity. What we know from the written sources is that 'Slavs', first mentioned by Roman authors in the middle of the 6th century, soon spread across increasingly wide areas of Eastern Europe. They had settled in many parts of the Balkan Peninsula, in Bohemia and the lands to the north of it in the 7th century, and inhabited large parts of Central and Eastern Europe in the 9th century: from the Baltic to the Aegean Sea and from the Eastern Alps to the Dnjepr and beyond. On the basis of the patchy evidence, historians and archaeologists have debated whether that was due to the expansion of a clearly-delineated ethnic group from an 'original homeland' (which would hardly explain such a wide diffusion), to the gradual Slavicization of a variety of previous populations ('becoming Slavs'), or to an umbrella term 'Slavs' used by foreign authors for disparate groups with a similar lifestyle, which only gradually adopted this name for self-identification[6,8–10]. None of these hypotheses can explain the entire process. The presence of northeastern ancestry in Mödling, and later also in other places, seems to indicate extensive migration from north of the Carpathians to Central and Southeastern Europe.

In the course of the over 250 years of Avar rule in the Carpathian Basin, the ethnic composition of the population, as perceived in the written sources, seems to have changed. In the reports by Byzantine, but also by some Western sources in the 6th and 7th centuries, the groups mentioned under Avar rule included Cutrigurs, Utigurs, Bulgars, Slavs, Gepids, Longobards and Romans, the latter mentioned as captives settled in the Avar core area. A unique written testimony of processes of admixture and shifting identities in 7th-century Pannonia has been preserved in the contemporary account of the Miracles of St Demetrius of Thessalonica (chapter 2,5). In the Avar and Slavic raids of the Balkan provinces around 600, as the text explains, the khagan deported the population to Pannonia and settled them there as his subjects. From then on, 'they mixed with Bulgars, Avars and other peoples, having children with each other, and became a very numerous people; and each child received from the father the traditions of his homeland. When sixty years and more had passed since the barbarians had captured their fathers, a group of new people had appeared in these regions, and over time most of them had acquired their freedom.' Then the khagan gave them a

leader, a Bulgar named Kuver. The desire of those raised in their Roman tradition to return to Roman territories, and of the Bulgars to escape from the rule of the Avars then led to a successful flight to the region of Thessalonica still under East Roman control. The narrative may be topical in several ways, but relies on information from fugitives arriving in Thessalonica, whose account of their fates sounds plausible.

As a result of these changes in population and its identity, the Latin evidence of the 8th and early 9th century only speaks of Avars in the Carpathian Basin (sometimes identified with the Huns), who were surrounded by Slavic groups on all sides: in the Eastern Alps and north of the Danube, north and east of the Carpathians, on the Lower Danube and in the north-western Balkans. The population of the Avar realm was not differentiated along ethnic lines anymore, they were seen as 'Avars'. One of the rare names of an Avar dignitary mentioned in the texts, explicitly presented as 'of Avar origin', had a Germanic name, Unguimeri[6]. ,

The processes of admixture, changing cultural habitus and shifting ethnic identities, however, did not proceed at the same pace in the 7th and 8th centuries. The cultural and ethnic plurality in the early Avar Empire gradually gave way to integration in the course of the seventh century, which is clearly visible in the archaeological record. The cultural remains from the Late Avar period do not allow delineating clear subgroups, and the forms of self-representation (especially the belt sets, see Section 8) are surprisingly similar across the Avar settlement area, which stretched roughly from the Vienna Woods to the valleys of Transylvania. This seems to have corresponded to the outside perception of this population as 'Avars', although we can only hypothesize whether that corresponded to a strong self-identification. As we see from the examples of Leobersdorf and Mödling, this ethnic and cultural unification was not the result of genetic admixture, or of the prevalence of either the 'Asian' or the 'European' ancestry. Genetic differences remained, and were also maintained by the deliberate choice of reproductive partners. It was rather a matter of a population of varied origin 'becoming Avars', of demonstrating by outward signs (for instance, belt sets) that one belonged, but of maintaining internal differences. Within the common frame of a shared Avar identity with a political basis, various strategies of distinction were employed that could set one group or community apart from others – for instance, by consistently marrying wives of Asian ancestry, as it happened in Leobersdorf.

c. Ethnicity, ancestry, culture: methodological and terminological approaches

An important result of the present study, then, is that the ethnic names used to describe certain groups in the written sources did not simply reflect units set apart by more or less co-extensive political, cultural and genetic boundaries. We have to distinguish between these three (and further) forms of defining groups of population. Such distinctions raise problems of terminology. What do all these ethnic names in the texts mean, and how do we synchronize them with genetic and archaeological interpretations? Ethnicity and identity are among the categories that have been much-challenged in the humanities and social sciences because they are not easy to define, but easy to misuse for identity politics and nationalist ideologies. But that should not lead us to abandon them – on the contrary, it shows that they describe issues that really matter in many societies, past and present. The present paper relies on the following definitions: ''Ethnic' is a relational mode of social organization among a number of distinctive groups, which are perceived as being constituted by an ingrained common nature. 'Ethnic identity' denotes a reciprocal relationship between a person and a group that is reproduced through verbal or symbolic statements and acts of identification and complemented by ascriptions of alterity. These identifications, and the symbolic markers used in them, are regarded as expressions of an inner self (individually) and of a natural community (collectively). It is this belief in the ingrained nature of ethnic identity in a person that is at the core of the concept[3]. This means that 'ethnicity' does not necessarily refer to an actual, more or less homogeneous biological group with shared descent and ancestry, but to the belief that it was.

The historical evidence about the period under study here, the so-called 'Migration Period'[11], clearly shows that groups acting under one ethnic name could split, and merge with groups of rather different origin, who would at some point adopt the ethnic identity of its leaders[12]. Migrations could accelerate this process. This did not require actual admixture, but just identification with the group one felt one belonged to, and its acceptance by that group. It could, of course, build on or lead to shared ancestry, but we cannot take that for granted. Although this was a process influenced by and also expressed in cultural choices, it finds no direct correspondence in the cultural remains recovered by archaeology either, although many archaeologists have long tended to identify 'archaeological cultures' with ethnic labels[13].

Therefore, we have to keep the three aspects apart in the research process, which can be exemplified by the area under study: First, the genetic and bio-informatic results, according to which even in the three culturally very similar Vienna-Basin sites analyzed in the present article, everything between almost exclusively Central Asian or European ancestry was possible, as were different shades of admixture. Second, the archaeological record that shows a process of cultural blending from a considerable variety of forms in the late sixth century to a surprisingly homogeneous style of representation in the eighth, as represented by the belt-sets with bronze fittings – although some less conspicuous differences between sites remained, for instance in the use of pottery in graves. And third, the groups and identities in the Avar Empire defined by ethnonyms, as summed up above. Our evidence does not allow us to reach any conclusions as to a fourth element, language. We know that many peoples of Germanic origin spoke Gothic or another Germanic language, and that Slavic was used in most regions of Eastern Central Europe in the 9th century. We do not know which language Huns and Avars spoke, it may have been a Turkic or Mongolic language, or even an unknown one[6]. At the court of Attila the Hun, multilinguality (Hunnic, Gothic, Latin, Greek) is attested, which is also plausible for at least the Early Avar Period.

Thus, we should be careful not to use any of these historical, archaeological and genetic (and linguistic) groupings as a proxy for any of the others. However, they were hardly completely independent of each other. This leads to a problem with the use of ethnic names for cultural and genetic groups, which is still rather current in both disciplines. As a principle, we should avoid ethnic labels both in genetics and in archaeology, at least where we do not have sufficient evidence that the individuals buried in a site were consistently identified by that name. Even then, we can never be sure whether each of them would have identified him/herself, or would have been regarded by others as members of the ethnic group in question. Affiliations to ethnic groups were usually well-defined in a core group and fuzzy at the margins, although notional boundaries between 'us' and 'them' may have been rather well-determined in some contexts, for instance, when privileges were at stake. Affiliations could also be graded in concentric circles, in which a core group regarded itself as (for instance) Avars in the full sense. Over longer periods, the range of accepted belonging to a ruling group in a given population could gradually become wider, and include formerly excluded groups, often of lower status. This is, for instance, how the Germanic Franks and their Romance-speaking subjects eventually became the French.

We can assume a similar process in the case of the Avars. As the dominating group in the Rouran khaganate seems to have called themselves 'Avars', initially only the descendants of this group may have regarded themselves as Avars in the full sense. On the march, other warrior groups from the Rouran realm and beyond joined, and steppe origins and shared hardships and successes may soon have given them opportunities to be accepted as 'Avars'. Taken together, these were the 'Avars' that appeared north of the Caucasus in 557, and whom we call 'Avar core group' here. At this point, they largely must have corresponded to the group with Eastern Central Asian ancestry pinpointed in the Gnecchi-Ruscone study[4], although not all of these 'Asians' necessarily identified as Avars. These newcomers from Central Asia, however, cannot be distinguished easily in the archaeological record from the earliest phase after their

arrival in the Carpathian Basin in 567/68. They also increasingly attracted new groups of steppe riders from the Pontic steppes, with whom some degree of admixture must have happened from the beginning. These groups continued to be distinguished under their ethnonyms as Cutrigurs or Utigurs in the late sixth, or more broadly as Bulgars until the end of the 7th century[6]. The Avar elite of mainly Asian origin largely maintained their status, and also seems to have sought to marry women of similar origin. Still, the large overlap between the leading 'core group' and the eastern central Asian ancestry became fuzzy in some places. What the evidence from Leobersdorf crucially shows is that entire groups of mostly Asian ancestry did not display elite or warrior status any more in the 8th century, and had moved out of the core area of the Avar realm.

The sex bias in the gene flow analyzed for Leobersdorf and for four sites east and west of the Tisza river (see Section 5-6) clearly indicates that the main driver for admixture into the Central Asian ancestry were unions of Asian men with women from the Pontic steppes or from Central Europe. Yet this was far from being a steady process, which would have led to a progressive assimilation of the Asian immigrants. The example of Leobersdorf indicates that the social practice of preferring reproductive unions with women with a high level of Asian ancestry led to its preservation over a period of 200 years and more. The low rate of IBD sharing between Leobersdorf and the neighboring 'European' population of Mödling shows that such unions were rather avoided, while IBD connections between Leobersdorf and distant Rákóczifalva and other sites of that area were more intense. These could be due to the immigration of the Leobersdorf population from the Avar core area or result from more recent connections between the regions. As in the study of the center of the khaganate[5], the IBD analysis done for the present study points to higher connectivity of females between the sites. The pedigrees also disclose patrilinear and exogamous communities, in which young females who were to become mothers regularly came from outside and had no ancestors on the sites. Consequently, we find very few daughters born by local parents buried on site as adults; they had obviously gone elsewhere to be married. Evidence from physical anthropology (especially age-at-death) plays an important part in these reconstructions.

In the early-Avar Carpathian Basin, 'Avars' were distinguished from resident Gepids, Slavs, 'Romans' and Bulgars. In 599, Byzantine observers reporting about a Byzantine army that attacked Avar territory and made numerous prisoners in a battle at the Tisza River differentiate clearly between Avar, Bulgar, Gepid and Slavic contingents among their captives[6]. Bulgars and Slavs had to carry out the brunt of the attack on the walls of Constantinople at the siege of 626, while Avar horsemen stood by. A story about the miraculous protection by Saint Demetrius against an attack on Thessalonica in the late 7th century describes how the descendants of Roman captives had gained their freedom in Avar Pannonia and mixed with Bulgars and Avars, before the resulting 'new people' defeated the Avar army, left the realm and marched on Thessalonica[6]. Bulgars appear rather often in the 7th-century Avar realm, but they cease to be mentioned around the time when Bulgars from the Pontic steppes founded their khanate around the lowest stretch of the Danube. On the whole, we can assume that the number of people identified as Avars must have grown, because the other ethnonyms now disappear from the texts. The genetic and cultural flows also increased, but not at a steady pace and hardly in conjunction with ethnic or cultural integration. One of the important results of the present study is that perceptions of people as Avars in the texts, the unification of material culture and genetic admixture did not follow analogous rhythms, and that these shifts led to rather diverse genetic results in different local communities.

d. Relatedness, kinship and the status of women – historical observations

The basic facts of biological relatedness may be immutable, but they are surrounded by a wide area of kinship practices: fertility rates; incest taboos; marriage bans; polygyny/polyandry; regulations for abortion; uneven conditions for the survival of mothers and children; differing social roles of mothers/fathers and of

female/male children; relations between mother/father and child; patrilinearity/matrilinearity; the extent of adoption and ritual brotherhood; chastity requirements; inheritance rules; and the entire structuring of biological relations[14]. All of these factors result from cultural codes and social practices, and reflect back on the structure of biological pedigrees, which we can now reconstruct on the basis of genomic data. Only some of these phenomena can be clarified by genetic methods; for some, we can find traces in archaeological and anthropological evidence; some we will never know; and for the larger context, we can rely on written sources.

We have relatively scarce written evidence about the roles of women and of kinship in the Avar period[6]. Not unexpectedly, we almost exclusively hear about rulers and their families. We know that both Attila king of the Huns and the early Avar khagans had several wives and therefore numerous sons. At least some wives and sons accompanied Baian's son and successor on campaign into Byzantine Thrace in the 580s, where the women enjoyed themselves in the Roman baths, whereas several sons died of the plague. We also hear that the Avar chief priest once had an affair with one of the khagan's wives, upon which he had to flee to the Romans for fear of a death penalty. Only at the end of Avar rule do we get a precious piece of information about the role of the khagan's main consort, the *katun*. When after the Frankish victory in 796, the khagan came to the Frankish camp to submit, he was accompanied by the *katun*, and they were told that their reigns (in the plural) had ended[15]. This corresponds to the evidence from 8th-century inscriptions of the second Turkish khaganate in Mongolia, which also attest a shared rule given by the heavenly god to both the khagan and the *katun*. In the earlier Byzantine sources, a main consort with an important position is not attested, and it may only have been adopted by the Avars in the late 7th or 8th century, together with a number of titles of rank current in the eastern steppes[6].

The interdisciplinary approach taken in this study requires methodological caution in several respects. First, assessing structures of relatedness requires, as far as possible, an analysis of large and entire burial communities. In our research, the conclusions from the pedigrees of the almost 500 individuals buried at Mödling were much more fine-grained and in some aspects differed from the provisional results reached after an analysis of the first 150 sequenced skeletons. As becomes clear in many sections below, quantitative and statistical analyses that connect anthropological and archaeological data with positions in the pedigrees have great potential, and require full coverage of the material.

Second, and similar to the reconstructions of the role of ethnicity, we have to distinguish between biological relatedness and social kinship, and cannot take it for granted that the two coincided. Only when we keep them apart in the interpretation of our data can we then proceed to assess to what extent they coincided. Clues in our evidence may come from multiple burials, for instance of adults and children – were they biologically related or not? Were genetic relatives buried close to each other, and did they have similar objects in their graves? In both Leobersdorf and Mödling, such traces occur relatively frequently (see Section 7), and allow to conclude that biological relatedness was an important factor of social cohesion – as emerges already from the fact that at both sites, the vast majority of individuals were somehow related with one another.

Third, however, we should not take traditional Eurocentric models of kinship, long used in social anthropological and historical studies, for granted. In fact, in both disciplines the concept of kinship itself has recently been criticized, mainly because of the great cultural variety of roles that different forms of kin relations play in human communities[16,17]. Concepts of kinship pervade all societies, but they differ, and often include several types of non-biological relationship, such as adoption, foster parenthood, or ritual brother/sisterhood. Bonds to non-relatives could be much more effective than those to parents or siblings[18,19]. We have little opportunity to make such observations in our material. Basically, we have more to

gain from ethnographic reports about Central Asian steppe peoples of the past and from current social anthropological studies in this region[5]. Yet we should also be cautious in following traditional models which often put the role of the clan in the forefront. Social models for the Mongols which assume a coherent structure of clans and tribes have recently been criticized[20]. Even though the evidence of Leobersdorf and Mödling suggests an important role of patrilineages, we do not know how these communities would have conceived of their kindred – was this one big clan, and if not, where would contemporaries have drawn the boundaries between clans? The sub-pedigrees that we have constructed, also taking archaeological and anthropological clues into account, are just one possibility to structure the pedigree, and may not correspond to contemporary perceptions.

Still, we have much to gain from an interdisciplinary venture such as ours. We can hope to find new clues about reproductive unions and strategies; the status and positions of women; conditions for admixture; changes or maintenance of ancestries; social roles and status differences; the social organization of burial communities; the impact of diseases; the chronology, the beginning and the end of cemeteries; the demography of the sites; relations between contemporaneous or successive sites; the impact of migrations and mobility and other aspects. As the experience in the present study shows, involving all concerned disciplines can take us far beyond what each discipline can achieve on its own.

e. Towards an integrated history of Avar-period communities in the Vienna Basin

The core area of Avar rule and settlement, roughly in the territory of modern Hungary, had its crises and frequent population shifts in the 5th to 7th centuries, but there is continuous archaeological evidence of small-to-medium communities living in the region, mainly around Lake Balaton and along the Danube and Tisza rivers[7]. Population size and prosperity seem to have grown in the early Avar period (c. 570-630/50), and some of the richest graves are known from the middle of the 7th century. At the same time, the Vienna Basin seems to have experienced a period of low population density or at least scarcity of archaeological remains, in particular, after the Longobards had left for Italy in 568. It may be that some Slavic groups settled there, but the rare evidence is inconclusive. Although the region surely stood under Avar rule, early-Avar period traces are largely lacking. Only in the middle of the 7th century, larger groups of population from the Avar core area, perhaps also from other directions, began to expand there. The cemeteries of Leobersdorf, Mödling and Wien-Csokorgasse now emerged, as did several others in the region, until a relatively dense population lived here in the 8th century.

The initial phases of Leobersdorf and Mödling, although not exactly contemporaneous, suggest a rather gradual and experimental beginning of these cemeteries, and probably also of the respective settlements, which have not been found. In contrast to the density of cemeteries, we have as yet hardly any evidence of settlements from the period between the 7th and 9th centuries in the Vienna Basin south of the Danube[21]. The few existing traces of settlements are sunken buildings or workshop areas. Therefore, settlements of this period are not easy to find, all the more if the initial settlers in the Vienna Basin may have led a nomadic rather than sedentary life. In any case, in the beginning, around the mid-7th century, only scattered graves were dug, only few of which have been found in the area of the later cemetery. The pedigrees suggest a rather small group of founders, part of whom we can only reconstruct through their offspring. The most plausible explanation would be that the group that began to bury their dead at Leobersdorf still engaged in animal husbandry with short-range mobility, lived in yurts or seasonal huts and buried their dead near the place where they happened to settle. Possibly, the zone of scattered graves stretched far beyond the cemetery that was excavated, and the population was in fact more extensive than we can reconstruct.

However, that does not reflect in the Leobersdorf pedigree, where the later population is descended from a small founding group. In the third generation, the population grew, and inhumations became more structured. Gradually, graves began to be organized in clusters of closely-related individuals. In the fourth generation, the number of people buried there reached a peak, and inhumations became very dense, although they rarely overlapped, so previous graves must have been visible. In the fifth generation, population decreased again, and in the sixth generation, mostly children were buried, which indicates that those who lived to grow older left the place or died without a proper funeral.

In Mödling, the development of the cemetery, more than three times the size of Leobersdorf, was roughly similar. The deceased are of mostly European ancestry, with only a few individuals with minor Eastern Asian elements (see Section 6). In this respect, their genetic background is more homogeneous than in cemeteries with a higher percentage of Asian ancestry. Still, within this basically similar European ancestry, they are far from homogeneous (see Section 6). It needs to be stressed that roughly contemporary reference samples still are missing from many regions, so we have to work with proxies that do not allow more precise localisation. About half of MGS ancestry points to the region roughly between the Baltic Sea and the Carpathian Arc. This most likely is a trace of an earlier migratory movement by what the written sources usually call Slavs. Smaller components can be linked with Western Asia (probably descendants of a population of Eastern Mediterranean origin that had lived in late-antique South-Eastern Europe), and with the Western European continent (that is, the Frankish kingdoms). Both of the latter groups may go back to earlier populations in the wider area, but hardly in the Vienna Basin itself, given that we have only faint indications of population continuity there. These populations had mixed for some time, so the migrants from the northeast had already been in contact with them by the 6th century. It is striking that in this case, we do not have a sex bias in admixture (see Section 6). This means that about as many of the 'northeastern' men married women from other parts of Europe as vice versa. This was not an admixture between high-status men and lower-status women, but among groups of equal status. Most likely, their ancestors had all been subaltern groups in the early Avar Empire, whose offspring acquired full liberty and perhaps also Avar identity, and proudly displayed the same markers of status as the ones used by the community of mainly Eastern Asian origin in Leobersdorf.

Most of the 8th-century cemeteries in the Vienna Basin terminated around 800, when the Franks began to control the region, and a remaining Avar tributary principality with its center west of Lake Neusiedl did not succeed in achieving lasting stability. Slavs more or less under Frankish suzerainty began to take the political initiative – some of them may have been the descendants of groups using Avar forms of representation like the one in Mödling. An Avar prince is last mentioned in 822, and then the Avars as a people, their polity and its remarkably homogeneous material culture vanish.

In many respects, the reconstruction of biological relatedness and its contextualisation in matching archaeological, anthropological and historical evidence is generating a novel view of Avar-period society and ways of life. The interdisciplinary approach used in this study has created a new integrated toolbox for the chronology of mid-to large-size cemeteries: the succession of generations and the genetic inference of relative birth dates complement the archaeological methods of object typology, stratigraphy and succession of inhumation (and, where available, coin dating); it can be related to anthropological aging as a means to infer dates of death; and it can be used as an additional proxy to arrive at more precise modeling of ^{14}C ranges. These results can then be accommodated within historically plausible time frames. The critical integration of multidisciplinary results renders a plasticity to past lives that neither the archaeological traces of individual remains nor the naked pedigrees can disclose. Furthermore, pedigrees and IBD-relations allow putting reconstructions of ancestry in perspective. Estimating ancestry is a valuable tool to trace migratory events and the shifting composition of ethnic, territorial and political groupings. However, as the present

and further examples in the analysis of Avar-period cemeteries can show, ancestry provides no sufficient basis to decide 'who we really are'. Local communities and the pedigrees at their core are potentially more stable than biological ancestries that can be modified within a few generations depending on reproductive strategies. One lesson to be drawn from the present study is that ethnic or regional patterns of identification may evolve quite independently from biological ancestry, as has resulted from much previous historical research[3,22]. Biological patterns and the identities of local communities may both be closely related to practices of reproduction, as in the case of Leobersdorf. In a nutshell: biology did not determine social identity; both were ultimately the result of social practices and cultural patterns enacted by local communities.

3. Archaeological overview, sampling strategy and chronology

a. Site selection criteria

The Vienna Basin was quite densely populated in the Late Avar period (8th c. CE), and a number of sites have already been fully or partly excavated (e.g. Sommerein, Zillingtal, Vösendorf). The sites of Leobersdorf and Mödling have been selected for sampling because they are fairly representative, well-documented, and just the right size to be analyzed in their entirety. Mödling, in fact, is the largest archaeological site of any period fully sequenced by the current date to our knowledge. The two sites, at a distance of under 20 km from each other, are also well-suited for comparison between each other and to other Avar-period sites. The third Avar-period site, that of Wien-Csokorgasse, was too big for complete sequencing, but has been partially sampled for specific comparative questions. Individuals from all occupation phases of the Wien-Csokorgasse cemetery were selected based on the well dated archaeological finds. The grave furnishings of many of these individuals show influences from the east. The initial research question was whether these influences could also be attributed to migratory movements from within the Avar realm or from the Pontic steppes. Two smaller cemeteries of Mödling-Lerchengasse and Mödling-Leinerinnen dated to the late 4th and 6th century were selected to examine possible connections to the previous centuries.

b. Description of the sites: Leobersdorf-Ziegelei Polsterer, Mödling-An der Goldenen Stiege, Mödling-Lerchengasse, Mödling-Leinerinnen, Wien-Csokorgasse

All five genetically-investigated cemeteries are located in the present-day Vienna Basin, south of the Danube. The southernmost cemetery, located in **Leobersdorf** on the western edge of the Vienna Basin at the exit of the Triesting valley, was discovered accidentally on the site of the Polsterer brickyard, when in 1953 a single grave came to light about 330 m away from the other burials. Between 1977 and 1983, a total of 153 graves and 181 burials of the 7th to early 9th century were excavated. No evidence for the use of the burial ground before the early Middle Ages could be found[23]. About 18 km north of Leobersdorf, the cemetery of **Mödling-An der Goldenen Stiege** is situated on a terrace on the eastern slope of the Vienna Woods. A total of 488 graves with 536 burials of the 7th to 9th centuries were excavated between 1967 and 1973[24–29]. The burial site was built on the remains of a Neolithic settlement and a Hallstatt period cremation burial ground and was surrounded by an undated rampart[30]. About 900 m away and 20 m deeper in the flatland of the Vienna Basin, evidence of earlier burials from the end of the 4th (**Mödling-Lerchengasse**) and the 6th centuries (**Mödling-Leinerinnen**) was found[31,32]. The cemetery of **Wien (Vienna)-Csokorgasse** is located in the east of today's Vienna city area on the edge of a former river terrace of the Danube, about 16 km from Mödling and 32 km from Leobersdorf. It is not only the largest of the three investigated burial sites but, with a total of 745 graves, also one of the largest in the 7th-9th century period of today's eastern Austria. Between 1977 and 1978 a total of 705 graves with 771 burials were discovered[33–36]. Twenty years later, a small burial group with 40 graves was excavated immediately northwest of it[37–40]. Similar to the burial ground of Mödling-An der Goldenen Stiege, there was already a prehistoric settlement of the late Bronze Age on the

burial ground. All the selected cemeteries were discovered by chance during construction work. It can be assumed that most of the graves were excavated. However, some graves were certainly destroyed in the course of the work. Of course, it cannot be ruled out that there are still graves in the vicinity that have not yet been excavated.

c. Sampling strategy of the human remains

The aim of the project was to take samples of all individuals buried in the cemeteries selected, and we almost reached that goal. However, this was not entirely possible due to preservation issues; hence we employed the following sampling strategies.

The skeletons of Leobersdorf are housed at the Department of Anthropology at the Natural History Museum Vienna, except for one individual (Leo-ZP_071), who is exhibited in the Leum Museum Leobersdorf (Lower Austria). They were anthropologically first analyzed in 1987[41], where basic data, pathological conditions and measurements were recorded. For this project, the skeletons and data were revised and adapted.

For Leobersdorf, 33 or 18.2% of the 181 identified individuals could not be sampled (morphologically assessed they were 5 males, 3 females, while the others were either subadults, or no remains could be recovered from the grave at all). From the 155 samples of 147 individuals, most were from the petrous part of the temporal bone (n=120; 77.4 %), whereas teeth (n=26; 16.8 %) or postcranial elements (n=9; 5.8) were second and third choice. In terms of anthropological sex, the proportion of male to female bones in the sample is as follows: petrous bone 33.6 % males, 40.3 % females, 26.1 % undetermined; teeth: 40.9 % males, 31.8 % females, 27.3 % undetermined; other skeletal elements: 42.9 % males, 42.9 % females, 14.3 % undetermined. The proportions of the sampled bones of adults to subadults is: petrous bones 68,1 % : 31.9 %, teeth 59.1 : 40.9 %, and of other skeletal elements 85.7 % : 14.3 %. In 162 individuals, age at death estimation was possible. The result was that 33.3% subadults had died younger than 18 years old, and 66.7% died as adults. The morphological and genetic sex estimation of adults was concordant in Leobersdorf, except for one case where the skeleton was badly preserved.

The skeletons from the site Mödling-An der Goldenen Stiege are stored and curated at the Department of Anthropology at the Natural History Museum in Vienna, except for one individual (Moe-GST_144), who is exhibited in the Mödling Museum (lower Austria). They are inventoried under the numbers OSTE-27691 to 28205. K. Wiltschke-Schrotta collected the basic data on this skeleton series.

In Mödling, 536 individuals from the cemetery could be identified, of whom 47 (8.8 %) could not be sampled, as there were either no (n=27) or too few (n=20) skeletal remains present (morphologically assessed among the non sampled individuals were 0 males, 2 females, 13 indifferent adults and 8 subadults). Among all 489 individuals, the mainly sampled elements were the petrous parts of the temporal bone (n=317; 68.8 %), followed by teeth (n=116; 23.7 %), and lastly by postcranial elements or other cranial elements (n=56; 11.5 %).

In terms of anthropological sex, the proportion of male to female bones in the sample is as follows: petrous bone 33.8 % males, 28.1 % females, 38.2 % undetermined; teeth: 18.9 % males, 30.5 % females, 50.5 % undetermined; other skeletal elements: 32.1 % males, 17.9 % females, 50.0 % undetermined. The

proportions of the sampled bones of adults to subadults is: petrous bones 59.6 % adult : 40.4 % subadult, teeth 62.1 % adult : 37.9 % subadult, and of other skeletal elements 64.3 % adult : 35.7 % subadult. In 523 individuals, an estimation of age at death was possible whereas in 30 of them it could only be determined that they died as adults. Subadults made up 40.3% and adults 59.7% of the individuals buried in Mödling. The morphological and genetic sex estimation of adults was largely concordant in Mödling - in only 11 cases (or less than 5%) there was a discrepancy, because of preservation issues.

One of the earlier sites compared to Leobersdorf and Mödling in the analysis is called Mödling-Leinerinnen, consisting of seven graves[31]. The individuals include two subadults (infans I) and four adults, one of the latter was badly preserved, and from one grave no skeletal remains could be recovered. All except the last two mentioned could be sampled (petrous part of the temporal bones). A part of the skeletons was originally stored in the Mödling museum, but have been merged with those already in the Natural History Museum Vienna in the course of the analysis.

From the other earlier site, Mödling-Lerchengasse, two individuals are present and included in the analysis. The young adult male and the female child (infans II) were both sampled for the project (auditory ossicle and petrous part of the temporal bone). Both are exhibited in the Mödling museum.

In Wien-Csokorgasse, a total of 755 skeletons were identified (228 adult males, 223 adult Females and 204 of unidentified sex including subadults[42,43]. They are housed at the Department of Evolutionary Anthropology at the University of Vienna, and 83 individuals (11.1 %) were selected and sampled for aDNA analysis (see above), 20 temporal bones and 63 teeth. According to the anthropological assessment they consisted of 30 subadults (31.3 %) and 53 adult individuals (68.7 %). These are divided among the age groups as follows: 18 infans I, 6 infans II, 2 juveniles, 11 young adult, 22 young- middle adult, 14 middle adult, 9 old adult and 1 adult (not closer definable).

d. Sampling process

To reduce external contamination, all bone and teeth samples were transported to a dedicated ancient DNA cleanroom/laboratory. First, samples were photo-documented and UV-irradiated (30 minutes for bones and 15 minutes for teeth on each side). The further processing was conducted under controlled conditions by lab technicians wearing protective clothing (overall, sleeves, hairnet, face shield, respirators, gloves) with regular changing of latex gloves and continuous sterilization of the workplace (DNA-Exitus Plus, 1:10 bleach dilution). Samples from sites MGS, LEO, MLE, MLS were sampled in the ArcheoGen lab at the Department of Archaeology and Museology, Faculty of Arts, Masaryk University, Brno, Czech Republic. Samples from site CSK were sampled in the lab of the Department of Evolutionary Biology at the Vienna University, Vienna, Austria.

Bone powder of disarticulated petrous bones was produced following a previously published sampling protocol by Orfanou et al.[44] for minimally-invasive drilling of the dense parts in the inner ear (*auris interna*) around the cochlear area. Well-preserved intact skulls were sampled using a modified method by Sirak et al.[45]. in order to drill the targeted spot in the petrous bone from the cranial base and to minimize the damage to the skull. Teeth samples were processed as described in Neumann et al.[46] by drilling the inner pulp chamber of a tooth. For the sampling of other cranial or postcranial bones, steps from protocol by Orfanou et al.[44] were implemented. Bone powder of other types of bones was sampled by targeting the densest bone material in each sample.

e. aDNA laboratory analysis

25-50 mg bone powder gained by drilling was transferred to MPI-EVA, Leipzig, Germany. DNA extraction and subsequent laboratory steps were performed in the Ancient DNA Core Unit of the MPI-EVA. DNA was extracted from between 11 and 65 mg of powdered sample material using a silica-based method optimized for the recovery of short DNA fragments[47]. Briefly, lysates were prepared by adding 1 ml of extraction buffer (0.45 M EDTA, pH 8.0, 0.25 mg/ml proteinase K, 0.05% Tween-20) to the sample material in 2.0-ml Eppendorf LoBind tubes and rotating the tubes at 37 °C for approximately 16 hours[47,48]. Using an automated liquid handling system (Bravo NGS Workstation B, Agilent Technologies) DNA was purified from 150 µl lysate using silica-coated magnetic beads and binding buffer D as described in Rohland et al.[48]. Elution volume was 30 µl. Extraction blanks without sample material were carried alongside the samples during DNA extraction.

DNA libraries were prepared from 30 µl extract using an automated version of single-stranded DNA library preparation[49]. E.coli Uracil-DNA-glycosylase and E. coli endonuclease VIII were added during library preparation to remove uracils in the interior of molecules. Libraries were prepared from both the sample DNA extracts and the extraction blanks, and additional negative controls (library blanks) were added. Library yields and efficiency of library preparation were determined using two quantitative PCR assays38. Libraries were tagged with pairs of sample-specific indices via PCR extension using AccuPrime Pfx DNA polymerase[50]. Indexed libraries were amplified and purified using SPRI technology[50,51].

Sample and control libraries were enriched in-solution for 1,237,207 informative single nucleotide polymorphisms (SNPs) (a method commonly used in the field and known as the "1240K capture"[52]) targeting 394,577 SNPs[53] (390k panel) and 842,630 SNPs[54] (840k panel). Two consecutive rounds of 1240k capture were performed using the Bravo NGS workstation B. Libraries were pooled and sequenced single-read or pair-read on a HiSeq4000 sequencing platform (Illumina Technology). In total, 754 1240K enriched libraries were sequenced and median coverage of 1.42 on the autosomes, corresponding to a median of around 626,000 1240K SNPs covered at least once were obtained (Supplementary Table 1).

f. Chronology by ^{14}C dating

In order to establish an absolute chronology for the sites, ^{14}C dating was performed on 65 individuals from cemeteries in Mödling (abbreviated as "MOE" in the ^{14}C plots) and on 29 individuals from the cemetery at Leobersdorf-Ziegelei Polsterer (abbreviated with "Leo-ZP" in archaeological identifiers). Samples from MOE come from individuals from three different cemeteries - MOE-Lerchengasse, MOE-Leinerinnen and MOE-Goldene Stiege (abbreviated as "Moe-GST" in archaeological identifiers). The bone samples of Mödling and Leobersdorf were pre-treated and analyzed at two different laboratories - the radiocarbon laboratory of the University of Vienna (laboratory identifier "VERA"), Austria and the laboratory at Curt-Engelhorn-Center Archaeometry (CEZA) Mannheim, Germany (laboratory identifier "MAMS").

Bone pre-treatment at MAMS follows the modified Longin extraction method[55] that includes a series of Acid-Base-Acid washes. First samples are fully demineralized with 0.5M HCl at 4 °C, followed by a 0.1M-NaOH wash to remove humic acids overnight at 4°C and a final 0.4M-HCl wash to release CO_2 previously trapped in the NaOH solution. The bone collagen is then extracted in a pH3 solution at 70°C for 24h, insoluble material removed by filtration (Ezee filter), ultra-filtered to remove molecule chains < 30 kD and freeze dried. An elemental analyzer (microCube, Elementar Germany) was then used to combust the remaining collagen to CO_2, which is then reduced to graphite in either a custom build or commercially available (AGE3, IonPlus Switzerland) graphitization unit. An accelerator mass-spectrometer (AMS) of the

type MICADAS (IonPlus, Switzerland) was then used for determining the ^{14}C content and the conventional ^{14}C ages. Samples are analyzed together with blanks, normalization standards (Oxalic acid II) and quality control standards. The conventional ^{14}C ages are fractionation corrected and normalized to δ13C = -25‰. All bone material dated at MAMS shows good preservation with C/N-ratios (carbon to nitrogen ratios) of 3.2 to 3.3, which is well in the range of values (2.9 - 3.6) that are considered to indicate well preserved bone collagen[56]. The pre-treatment procedure at VERA follows a very similar procedure with a sequence of acid base acid washes. Samples are, in contrast to MAMS, not further treated by ultrafiltration. The determination of ^{14}C employs a 3-MV Pelletron tandem accelerator (National Electrostatics Corporation (NEC), USA).

The measured conventional ^{14}C ages were calibrated to absolut calendar dates using the software OxCal[57] and the dataset IntCal20[58]. Here, the discussed calibrated dates are generally given as the 2-sigma uncertainty intervals.

Supplementary Fig. 1-2 show the calibrated ^{14}C dates results of the sampled skeletons from Leobersdorf and Mödling, respectively. The sum probability distribution combining all individual distributions for each cemetery are shown in Supplementary Fig. 3, as well as the overall coverage of the ^{14}C ages for each site. Please note that the sum distributions are merely a visual tool that summarizes all probability distributions into one and does not include any statistical treatment or any other chronological consideration of the data.

Generally, the individual calibrated ^{14}C dates cover rather large periods, often spanning up to 150 years as either a single wide range or a bimodal distribution with two possible date ranges. Even though the conventional ^{14}C dates are measured very precisely (uncertainties of 20-38 years), the shape of the calibration curve with large wiggles during that particular period of time prevents more precise calibrated dates when relying on ^{14}C alone. Note that the VERA dates generally have larger uncertainties of around 24-40 years compared to the more recent measured data from MAMS with 17 - 22 years uncertainties. Thus, the calibrated date ranges are also wider for those individuals with higher ^{14}C uncertainties.

The earliest sample (Leo-ZP-11-VERA273) at Leobersdorf cemetery dates back to 595 - 665 calAD (calibrated 2-sigma range) and the latest sample (Leo_ZP-109-MAMS57297) to 774 - 886 calAD. Only two sampled skeletons from Moe-Lerchengasse were dated and point to an early usage covering the period 251 - 531 calAD (minimum and maximum boundaries of both calibrated ranges). The two sampled skeletons from the cemetery MOE-Leinerinnen cover a period of 414 - 600 calAD dating between MOE-Lerchengasse and MOE-Goldene Stiege. The large number of sampled individuals from Moe-Goldene Stiege provide a better picture of the usage time compared to the other two Mödling cemeteries. Its dates cover a very similar time period as seen in Leobersdorf with the earliest sample dating to 430 - 544 (Moe-GST 197_CO-MAMS63654) and the latest date at 679 - 890 calAD (Moe-GST-VERA54). The sum distributions of the dates of all cemeteries in Supplementary Fig. 3 visualize that a large number of sampled individuals are from very similar periods of time, with Moe-Lerchengasse and Moe-Leinerinnen reaching back to earlier dates compared to the cemeteries LEO and Moe-Goldene Stiege.

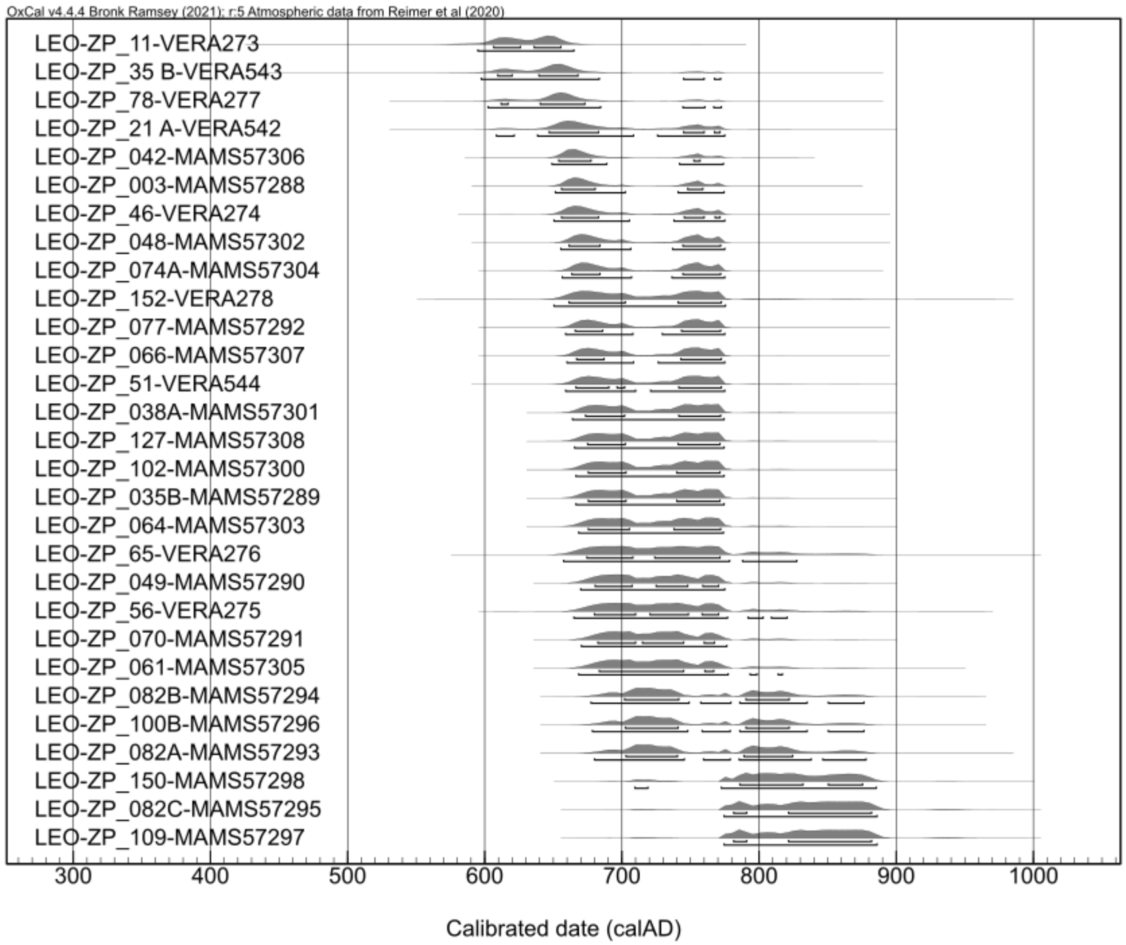

Supplementary Fig. 1: Overview of all calibrated ^{14}C dates obtained from the Leobersdorf (LEO) cemetery.

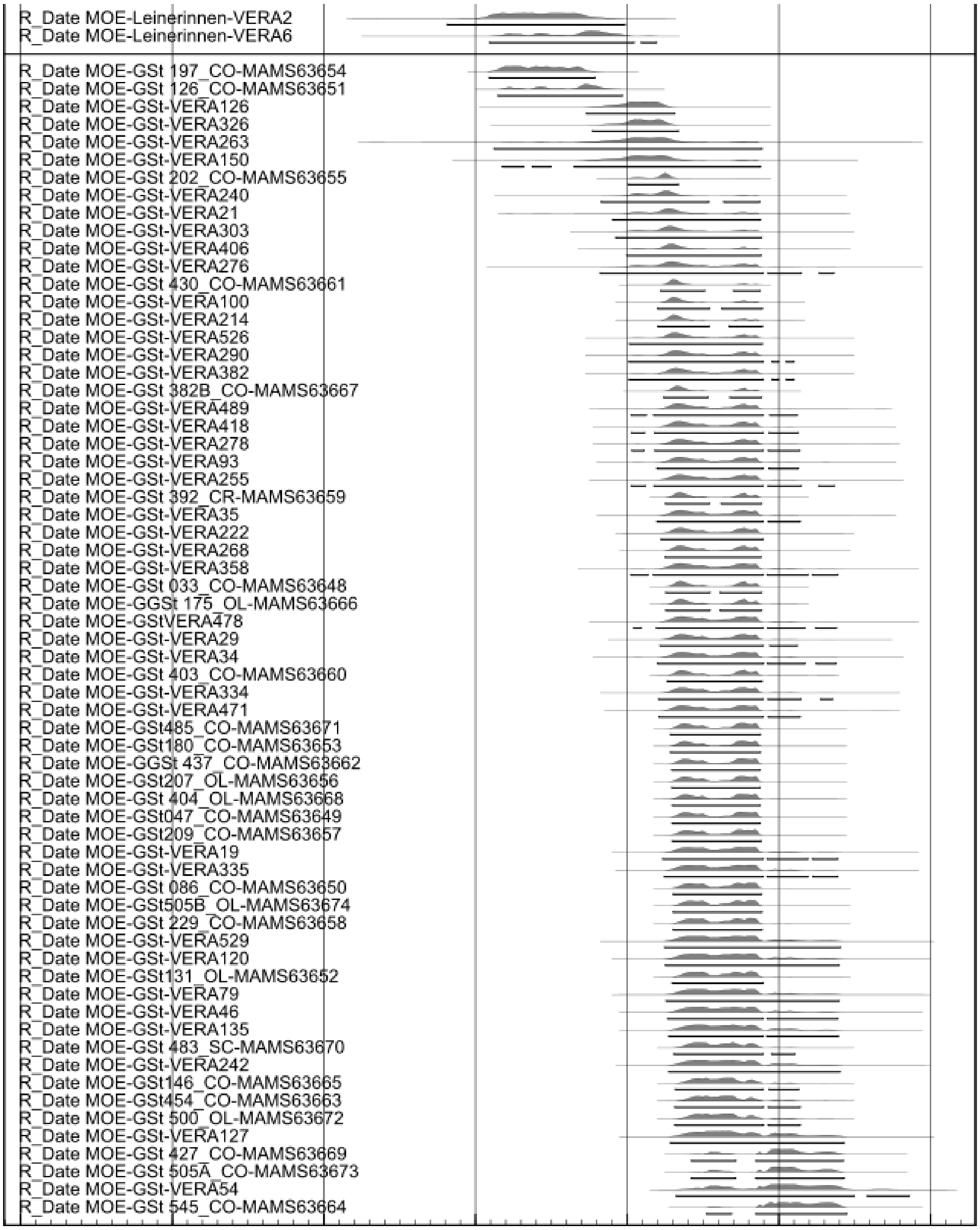

Supplementary Fig. 2: Overview of all calibrated ^{14}C dates obtained from the three cemeteries of Mödling (MOE): MOE-Lerchengasse, MOE-Leinerinnen and MOE-Goldene Stiege (MOE-GSt).

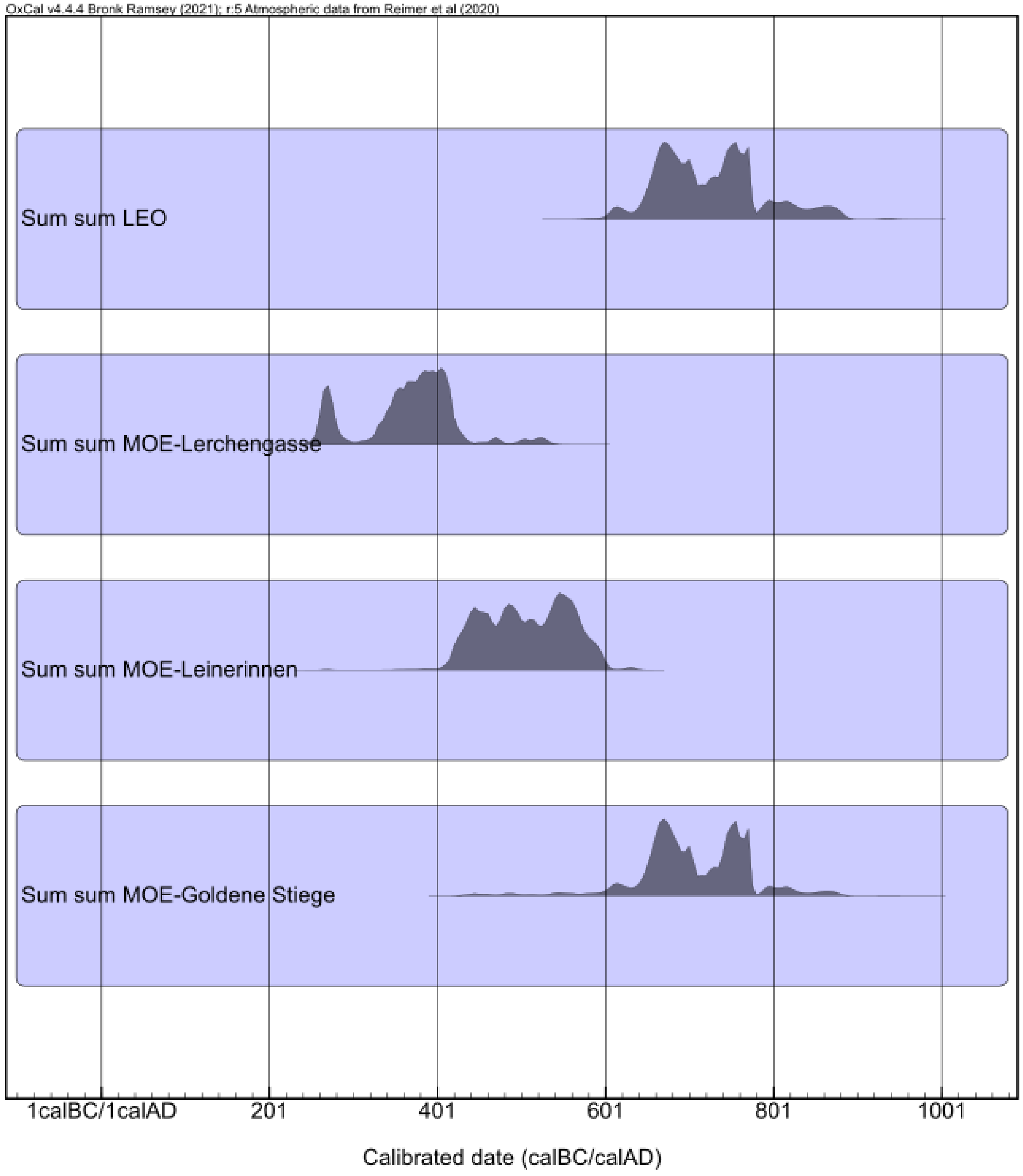

Supplementary Fig. 3: Sum distributions of the calibrated ^{14}C dates of the Leobersdorf (LEO) and the three cemeteries of Mödling (MOE): MOE-Lerchengasse, MOE-Leinerinnen and MOE-Goldene Stiege (MOE-GSt). Note that the data of MOE-Lerchengasse and MOE-Leinerinnen are composed of only two samples each.

4. Archaeological, anthropological and genetic methods

a. Archaeological approaches

The cemeteries under consideration had already undergone extensive archaeological study before the present project. A comprehensive monograph on the Leobersdorf cemetery has been published by F. Daim, co-author of this article[23]. Results of Mödling-An der Goldenen Stiege and of Wien-Csokorgasse are only partially accessible in published form[29,31,33], but there is extensive documentation. This material has allowed us to devise the research questions and prepare the sampling process. In parallel to the sequencing and the bioinformatic process, archaeological in-depth analysis has been conducted, increasingly focusing on the issues raised by the genetic results. The archaeological evidence made it possible to group the material according to various criteria such as period of inhumation, construction of the graves, sex, age, types of grave goods and markers of status. Due to the wealth of archaeological material from the Avar period, a rather precise chronology had already been established, based on object typologies and other dating criteria. The material from Leobersdorf, Mödling and Wien-Csokorgasse falls into the Middle Avar period (c. 650 to 710) and the Late Avar Period (c. 710 to post c. 800)[23], which can be divided into three sub-periods. A good number of publications on other Avar-period sites enable contextualisation and comparisons of the sites under study in this article.

Although archaeology has also developed numerous methods of qualitative interpretation, for the purposes of the present study, quantitative approaches have been prioritized, which allow addressing the pertinent research questions. The data on the archaeological finds were classified hierarchically and recorded in table formats. The analysis of the finds, including anthropological and genetic data, was carried out using the BI software Tableau. Furthermore, all relevant archaeological and anthropological data plus some relevant genetic and isotope data were entered into the project database.

b. Anthropological methods

i. **Sex and age at death**

In the following subchapters on anthropology we focus on age at death, sex, their interrelations with pedigree features, analyses per generations and finally present results on special questions linked to social structure. For this study, a new systematic anthropological analysis of Leobersdorf was performed, on the one hand in order to update and expand the already collected anthropological data by Grefen-Peters 1987[41], and on the other hand to achieve a basis of comparison with Mödling.

First anthropological data on Mödling-An der Goldenen Stiege had already been collected by Marhold 1977[59]. One of the co-authors, K. Wiltschke-Schrotta, evaluated all skeletons systematically in the 1990s. They are currently supplemented and partly revised according to a new anthropology protocol (methods and variables), which was agreed upon in a series of meetings between anthropologists from several countries involved in the project at its outset. It will also facilitate cooperation between anthropologists beyond the range of this study.

Methods of sexing include a macroscopic assessment of a set of a total of 30 features on the cranium, the mandible, the pelvis and the long bones. Characteristic features were graded between +2 (definitely male) and –2 (definitely female) and weighted according to their relevance. Pelvic features were given more weight than cranial features[60–64]. Subadults were not sexed morphologically. For calculations, individuals were classified into age groups based on mean age as following: fetus/neonate (around birth), infans I (0-6), II

(7-13), juvenile (14-17), young adult (18-25), young-middle adult (26-35), middle adult (36-50), old adult 50+, adult (18+, wide age spans of 30 years or larger (based on Buikstra & Ubelaker 1994[65]).

The methods used to assess age at death, sex, health and traumatic aspects through the morphology of the human remains followed standard protocols. Bone length measurements, tooth eruption patterns and epiphyseal fusion are taken into account for children[66–69] Methods of aging adults are based on dental abrasion, changes at the pubic symphysis, fusion of endo- and ectocranial sutures, changes in the sternal joint surface of the clavicle and epiphyseal fusion[60,65,70–73]. Data on age at death and sex for Wien-Csokorgasse were taken from Großschmidt (1990)[42].

For the individuals of Leobersdorf, additionally the Transition Analysis for estimation of age at death was applied. It combines information from different skeletal components of various traits, and uses statistical and machine learning methods to produce maximum likelihood age estimates. It is particularly suitable to refine age in older individuals[74,75]. This method is currently also applied to the Mödling skeleton series.

ii. **Anthropological evaluation of the pedigrees, distantly and unrelated groups**

The main aim of the detailed anthropological analysis is to reconstruct paleodemographic and temporal patterns in order to trace social structure and certain aspects of daily life. The analysis of the pedigrees is restricted to Leobersdorf and Mödling, and, where applicable, to Rákóczifalva. All the definitions of the termini used here can be found in the glossary.

Following genetic sex determination (see below) and the construction of the genetic relations leading to the pedigrees, we count the numbers of present and inferred males and females individually. Among the present individuals, the anthropologically estimated age at death group is crucial to determining details on generation sequence in the pedigrees (see below). However, in some cases individuals with wide age ranges were not included in some analyses. According to these results, it is possible to establish the number and sex distribution of the reproductive union's offspring, regardless of the fact that both, one or none of the parents are present in the pedigree.

The counted information is transformed into variables for statistical calculations in Excel and SPSS 23. In some cases, such as the comparison of the frequencies of matri-, patri- and bilinearity, the statistical analysis is restricted to chi-square (χ^2), and for small numbers, Fisher's exact test. In all cases, a p-value smaller than 0.05 was utilized.

In terms of the proportions of males and females in the sites, we established sex ratios for each of the pedigrees, distantly related, unrelated as well as inferred individuals associated to the cemeteries. This allows us to compare and statistically test further observations on patri-, matri- and bilinearity. The classification of the type of lineage is performed on the pedigrees attributing a higher weight to present or inferred parents of the individuals compared to siblings in the site. Among siblings, a higher number of siblings weighed more than only a single sibling.

To stipulate temporal, demographic and social patterns, we followed the generation sequence determined by genetics, archaeology and age at death (see below). Certain anthropological analyses required excluding the so-called first generation composed solely of inferred individuals. We used this approach for example to shed light on the demographic development, including the founding and abandonment of the sites.

According to the pedigrees it is possible to determine, count and compare different kinds of reproductive unions: single-, single in multiple-, and multiple reproductive unions as well as levirates. The number of children of the present individuals refers only to those who were exhumed and sampled. The genetically inferred individuals increase this number, however the total number of offspring is still an underestimation, as they could have been buried elsewhere and can therefore not be recorded here.

c. Methods of pedigree construction

i. General Pedigree Construction

We determined genetic sex for every newly sequenced individual in the dataset by the ratio of coverage on X and Y chromosomes versus coverage on autosomes. We expect females to have an approximately even ratio of X to autosomal coverage (X-ratio of 0.8) and a Y-ratio of 0, and males to have approximately half the coverage on X and Y as autosomes (0.4). For individuals with the ratio of Y to autosomal coverage falls between 0.1 and 0.4, we do not assign genetic sex. Hence the sex information of these are marked as 'U' in Supplementary Table 1. For 11 individuals of undetermined genetic sex, we have adopted the anthropologically assigned sex information (Supplementary Table 1).

The genetic relatedness was estimated from KIN[76], primarily using 1st- and 2nd-degree relations for pedigree construction. These data were complemented by genetic sex, mitochondrial haplogroups and Y haplogroups, and by the anthropologically-assigned age at death. Reproductive age, ^{14}C dates and archaeological chronology were also used for determining the directionality of the genealogy in rare cases.

The state-of-art software KIN[76], which we used for estimating genetic relatedness, applies a Hidden Markovian Model (HMM) to infer the relatedness between a pair of individuals through shared IBD segments. KIN can identify first-degree, second-degree and third-degree relatedness, while also distinguishing within-first-degree relationships, parent-child and full-sibling relations. This approach also allows classification of three types of second-degree relatedness (half-siblings, grandparent-grandchild, avuncular relatedness) to a certain extent, namely distinguishing grandparent-grandchild from avuncular relatedness. For each pair, KIN runs through one HMM for every possible relatedness model (including parent-child, siblings, grandparent-grandchild, avuncular, half-siblings, 3rd degree, 4th degree, 5th degree and unrelated), and classifies each pair to the relatedness model with the highest likelihood. Here, the relatedness assignments with log-likelihood greater than 3 are considered as statistically reliable (as per authors recommendation[76]), since both low coverage of data and cross-contaminations could lead to false classifications on the assignment.

For the list of analyzed pairs from three archaeological sites in the Vienna Basin (Leobersdorf, Mödling and Wien-Csokorgasse), we employed KIN as the primary approach for building the basic structure of pedigree with the aid of genetic sex and anthropologically assigned age information (see Section 4b). In the beginning, we run KIN on all individuals within the archaeological site, and then take the 1st degree related pairs to start pedigree-building. There are the following steps in the pedigree-building:

First, we identify all pairs of siblings related within an archaeological site and then we combine them to build multiple-siblings relations with the mutual sibling (we did not observe discrepancies in the ability to estimate mutual sibling relationships).

Second, we identify all pairs of parent-child relatedness, and assign the role of parent or child based on their age at death and sibling relations if any exists. When two or multiple siblings have the parent-child relatedness to the same target individual, we assign this target individual as a parent for these siblings. When two or multiple siblings have parent-child relatedness to two individuals of opposite sex who are unrelated to each other, we assign them as parents of these siblings and as a couple to each other. When the target individual has a unique parent-child relation that is not shared with other mutual siblings, we assign the role of parent to this target individual. In the cases of parent-child relations without mutual relatives involved, we use anthropologically estimated age at death and haplogroups information (mitochondria, Y-chromosome) as the primary information for assigning the role of parent and the role of child in the target pair. When one of the individuals is under the reproductive age (see Section 4), this individual could only be in the role of a child. As the age information only implies the age at death of the target individuals, in cases when the age information is not informative for distinguishing parent from child, we examine if the parent-child related pair of opposite sex share the same mitochondrial haplogroups (a sign of maternal inheritance), hence indicating mother-child relation. For the cases of undetermined directionality in parent-child and grandparent-grandchild relations, we examined the second relatedness derived from mutual relatives and the consistency of haplogroups. For instance, for a pair of parent-child who both share second-degree relatedness to the same individual, we could infer the progenitor and the descendent by the type of second-degree relatedness, namely the avuncular relatedness and the grandparent-grandchild relatedness distinguishes individuals from the progenitor generation and the descent generation.

Based on the genealogy structure built from the first-degree relatedness, we add more related individuals to the basic pedigree structure via second-degree and third-degree relatedness. The main challenge of assigning second-degree related pairs to the pedigree is that KIN allows distinguishing avuncular from grandparent-grandchild cases, while it does not exclude possibility of them being half-siblings when the estimate is avuncular or grandparent-grandchild. Namely, the half-sibling relations could not be distinguished from avuncular or grandparent-grandchild relations. In these cases, we attempted to resolve the puzzle by employing information from mutual relatives together with age information and uniparental marker haplogroup. In exceptional cases, chronological information from archaeological evidence could then be used (see Section 3,4). For example, when multiple siblings share a mutual second-degree-related relative, it allows three possibilities, including 1) the mutual relative from the same generation as half-sibling, 2) the mutual relative from the generation lower being nephew or niece, 3) the mutual relative from the generation higher, being the aunt/uncle, or being the grandparents. Using their age-at-death, we exclude option 3 if the age of the mutual relative is under reproductive age. By examining their mitochondrial and Y haplogroup, we relate the mutual relative to the father's or mother's side of the existing genealogy when they share the same haplogroup with either the father's or mother's side. By examining their archaeological chronology and radiocarbon date information, we classify individuals to different generation levels, under the rationale that the individuals from earlier generations could only be excavated from deeper layers than individuals from later generations and correspondingly the related radiocarbon dates should be rather consistent across individuals in the same generation (see Section 7).

Overall, for individuals with a relative within an archaeological site, we classify them into the following three categories that are further used in the text (Fig 2-3, Extended Data Fig 4, 5, 6):

i) individuals in the main pedigree;

ii) individuals who are distantly related to the main pedigree via third to sixth degree relatedness, herein always referred to by “distantly related”;
iii) individuals who are unrelated to anyone else sampled in the site, referred to herein as “unrelated”.

d. Genetically identical individuals and twins

In total, we found 9 genetically identical pairs of individuals in Leobersdorf and 12 in Mödling, which could be the result of either real twin relatedness (Leobersdorf: n=2; Mödling: n=9) or double sampling of the same individual. To distinguish between the two possibilities, we re-checked the sampling process, examined the anthropologically estimated age at death and the genetic sex of the skeletal remains and the burial location. When the two pieces of the skeletal remains are of different age, or distinct burial locality, or same part of skeleton (i.e. left petrous bone versus left petrous bone), we assigned twins for these genetically identical pairs (see Section 7) though misidentification prior to sequencing could not be excluded. The list of possible twins and all genetically identical pairs are reported in Supplementary Table 5.

In Leobersdorf, genetic analyses allowed the assignment of commingled skeletal parts of a potential double burial (Leo-ZP_16A to Leo-ZP_10) and of four individuals in multiple use burials, located in close vicinity in the cemetery (Leo-ZP_79b/B to 147, Leo-ZP_134A to 79b/C, Leo-ZP_134B to 134C, Leo-ZP_134B to 79b/D) to certain already genetically identified individuals. Moreover, two single graves showed commingling, and were allocated, Leo-ZP_061 and 013, as well as Leo-ZP_153 and 53b. In Mödling, genetic analyses allowed the assignment of commingled skeletal parts to certain already genetically identified individuals in three cases (in one case, commingled bones of two grave numbers and three individuals could be merged: the individuals sampled as Moe-GST_46 I (right temporal bone, petrous part), Moe-GST_46 II (left temporal bone, petrous part), could later be assigned to individual Moe-GST_47 due to size and age (bones fit to skull parts), and skull fragments Moe-GST_of 85 II (left temporal bone sample) could be assigned as missing parts of individual Moe-GST_102 (tooth 63 sampled) as well as skull fragments of Moe-GST_190_II could be assigned as the missing parts of individual Moe-GST_90.

ii. Pedigree validation

When constructing the pedigree, we used Y haplogroup and mitochondrial haplogroup information (Supplementary Table 1) for helping in determining the directionality of parent-child and the type of second-degree relatedness. After completion of pedigree-building, we performed sanity check of the pedigree by examining the mitochondrial and Y haplogroup for individuals in the pedigree to make sure that mitochondrial haplogroups along maternal lines and Y haplogroups along paternal lines are consistent. We encountered inconsistent uniparental markers only when the coverage on mtDNA and Y chromosome was too low and resulted in less reliable calls on mitochondrial and Y haplogroups (Supplementary Table 1).

At the same time, we performed IBD analyses using ancIBD[77] for validating the relatedness estimates from KIN and for the unclassified second-degree related pairs in the pedigree. As shown in Supplementary Fig. 4, we estimated the IBD sharing patterns for the individuals having more than 450 000 SNPs genotyped on a 1240k SNP panel, and marked the relatedness estimates from KIN in different colors. The amount of shared long IBD segments are informative signals of biological relatedness. Multiple sharing of long IBD segments is the direct evidence of very recent biological interactions, and hence it is possible to infer the genetic relatedness based on the distribution of IBD sharing pattern estimated with ancIBD[77]. For instance, the cloud of orange dots shown in Supplementary Fig. 4 are a group of parent-child pairs with shared particularly long

IBD segments. With the biggest ancient pedigree to date, which we constructed on the basis of the data from Mödling and which includes 337 individuals, we were able to compare the measured IBD sharing pattern to the estimated biological relatedness from KIN among the individuals in Mödling, Leobersdorf and Csokorgasse. The IBD sharing distribution fits well with biological relatedness. In the second degree relatedness estimates when the inferred relatedness from KIN was 'half-sibling', we have further investigated IBD sharing patterns to establish if grandparent/grandchild or avuncular relationship was possible (those two are mutually distinguishable by IBD sharing patterns, see Supplementary Table 3 and Ringbauer et al.[77], but both cannot be distinguished from half-sibling relationships). We generally examined the IBD sharing pattern for the pairs estimated with half-siblings, avuncular, grandparent-grandchild relatedness from KIN to differentiate avuncular and grandparent-grandchild cases as much as possible (in particular when it was possible to estimate IBD given the quality of aDNA of the individuals). This test provided independent confirmation for the relatedness results obtained through KIN. We also performed BREADR as another independent examination for estimating biological relatedness among individuals and reported the estimated relatedness consistency between BREADR, ancIBD and KIN. (Supplementary Fig. 5).

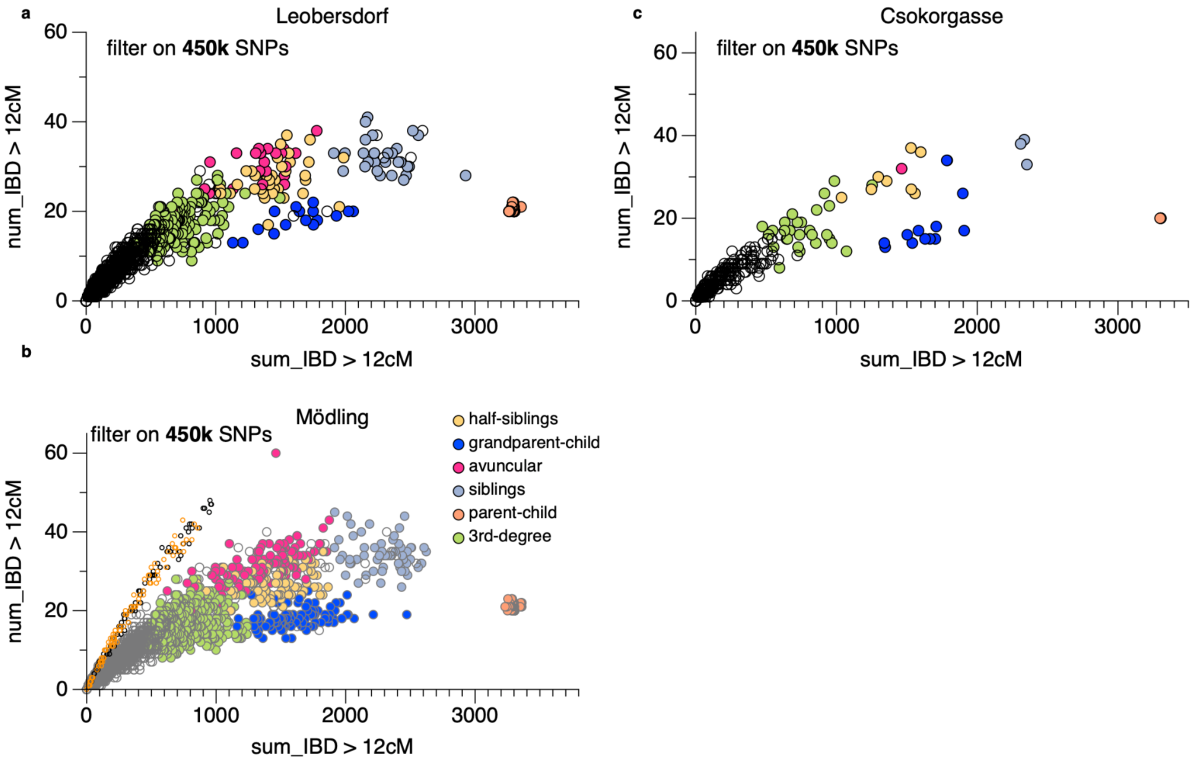

Supplementary Fig. 4. Estimated IBD-sharing among individuals within each site. a) IBD sharing pattern within Leobersdorf; b) IBD sharing pattern within Mödling; c) IBD sharing pattern within Csokorgasse. The plots visualize both the number count (y-axis) and the summed length (x-axis) of all IBD segments longer than 12 centimorgans, namely "num_IBD>12cM" and "sum_IBD>12cM" as labeled. We show that the relatedness estimated by KIn is in line with results based on IBD as reported in reference[77].

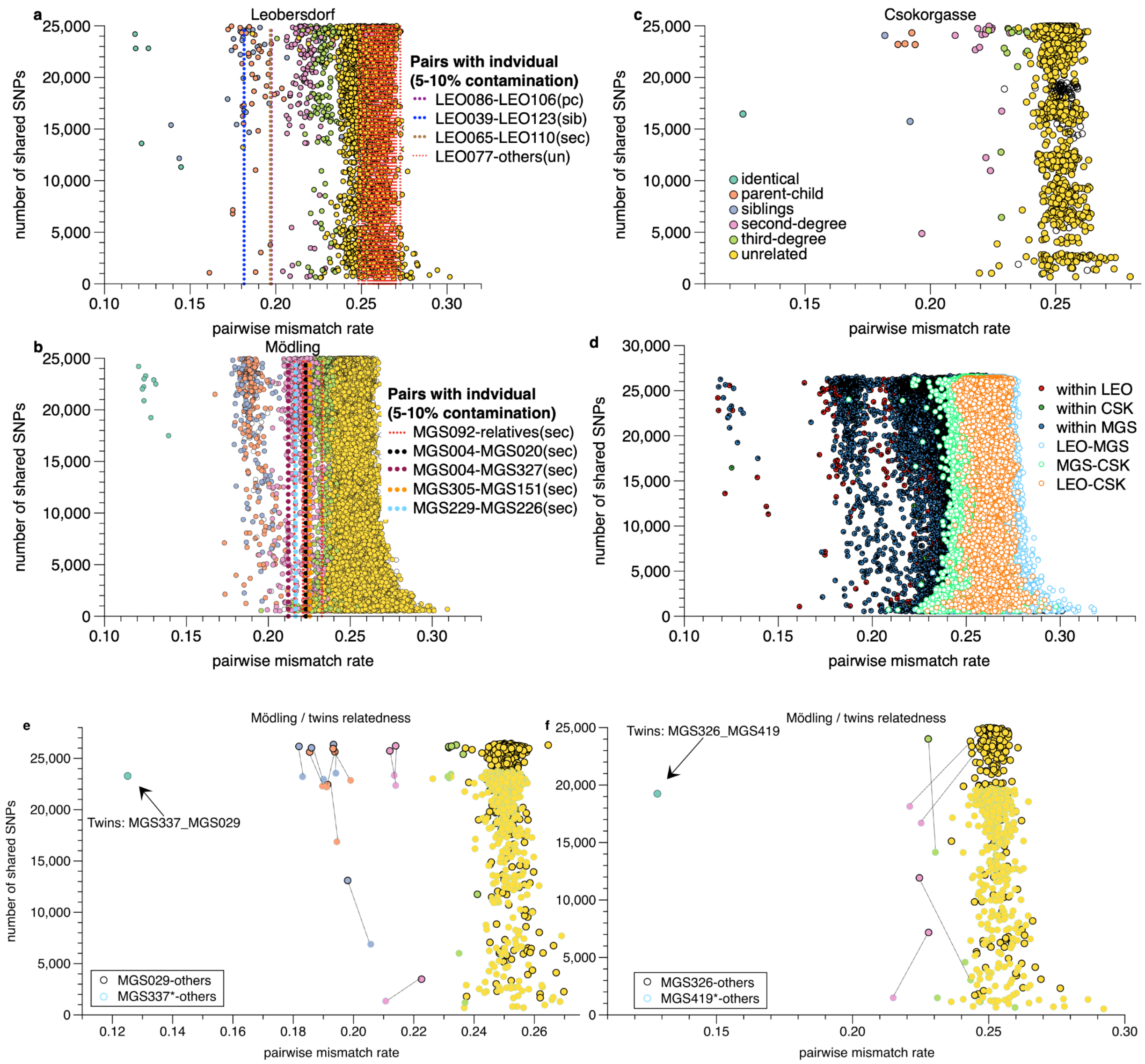

Supplementary Fig. 5. Estimated pairwise mismatch rates among individuals within each site and between sites using BREADR. Here, we highlight pairwise mismatch estimates for pairs involving relatively contaminated individuals in dashed vertical lines who are anchored in the pedigree via the paired individual (the estimated relatedness from KIN shown in brackets. a) Pairwise mismatch rate distribution within Leobersdorf; b) pairwise mismatch rate distribution pattern within Mödling; c) pairwise mismatch rate distribution pattern within Csokorgasse; d) pairwise mismatch rate distribution among the newly studied three archaeological sites. The dots in a) b) c) are filled with colors indicating the relatedness estimated from KIN, and the color scheme shown in panel b) is shared across sub-panel a) b) c). e) The estimated relatedness for the pair of twins MGS029/MGS337 with MGS337 marked with asterisk due to contamination. f) The estimated relatedness for the pair of twins MGS326/MGS419 with MGS419 marked with asterisk due to contamination. For pairs in e) and f), we marked the shift between pairs involving contaminated twin and clean twin in dashed lines for comparing the relatedness miscellaneous caused by contamination.

iii. Exceptional cases

We encountered exceptional cases while building the pedigree, which do not have consistent relatedness among mutual relatives, and could not be explained by the low data quality or poorly estimated likelihood. There are multiple scenarios that the individuals fall between the first degree and the second degree relatedness, namely 1.5 degree relatedness. This can be the case when a pair of individuals who are identified as genetic siblings only have one common parent while the other parent shares close biological relatedness, such as first-degree or second-degree related. This happens when a female has children with two brothers or other closely related males. In many cases, this was probably due to levirate unions (see Section 1), a practice of re-marrying widows within the clan known from written sources about steppe peoples, which was first genetically identified in Avar communities[5].

In Leobersdorf, we outline three potential cases of levirate patterns that involve three absent females partnered with a pair of brothers, a pair of grandfather-grandson, and a pair of father-son (namely the stepson of the involved female) (see also Section 7). In Mödling, we outline five potential cases of levirate where females partnered with a pair of brothers, and two cases of more specifical sororate where males partnered with a pair of sisters (see also Section 7). For individuals involved in potential levirate cases and exceptional cases, we examined the biological relatedness from KIN, BREADR and IBD sharing patterns, and summarized corresponding results in Supplementary Table 3-4.

We also report exceptional cases in Leobersdorf for levirate units and unusual pairs described below.

- Levirate unit 1:
    - LEO024 - LEO141: siblings in KIN
    - LEO024 - LEO049: siblings in KIN
    - LEO050 - LEO049: parent-child in KIN
    - LEO050 - LEO141: parent-child in KIN
    - LEO050 - LEO024: half-sibling in KIN, contradicts the sibling relatedness between LEO024-LEO049/141.
    - LEO024 - LEO144: siblings in KIN
    - LEO141 - LEO144: siblings in KIN
    - LEO049 - LEO144: grandparent-child in KIN, contradicts the sibling relatedness between LEO141-LEO144
    - LEO050 - LEO144: half-siblings in KIN, contradicts the parent-child relatedness between LEO050-LEO141 and the the sibling relatedness between LEO141-LEO144

Considering the direct contradiction to the first-degree related pairs, we proposed a levirate mating strategy for resolving the contradictions while constructing the pedigree. In such a way, the full sibling-ness estimate between LEO024/144 and LEO049/141 actually results from a shared mother mating with a brother, namely, they have 1.5-degree relatedness, instead of 1st- or 2nd-degree relatedness.

- Levirate unit 2:
    - LEO040 - LEO056: grandparent-child in KIN
    - LEO036/056/138/146: siblings in KIN
    - LEO045 - LEO040: avuncular in KIN, could be half-siblings
    - LEO045 - LEO056: avuncular in KIN, contradicts the second-degree relatedness between LEO040/056, and LEO040/45
    - LEO045 - LEO036: half-sibling in KIN, contradicts the sibling relatedness between LEO036/056 as LEO045 is in avuncular relatedness with LEO056

- LEO045 - LEO138: half-sibling in KIN, contradicts the sibling relatedness between LEO056/138/146 as LEO045 is in avuncular relatedness with LEO056
- LEO045 - LEO146: grandparent-child in KIN, contradicts the sibling relatedness between LEO056/146 as LEO045 is in avuncular relatedness with LEO056

To resolve the contradictions from estimated relatedness, we propose a levirate mating strategy that LEO045 shares the same father with LEO036/056/138/146, but has a mother mated with the son of LEO056 (offspring LEO040) and the father of LEO056 (offspring LEO045). So that LEO045 would fit as half-sibling to LEO036/056/138/146, and meanwhile as half-sibling to LEO040 - the grandson of LEO056.

- Levirate unit 3:
  - LEO068 - LEO038/083/092: half-sibling in KIN
  - LEO068 - LEO126: parent-child in KIN
  - LEO038 - LEO126: sibling in KIN, contradicts the half-sibling-ness between LEO068/038
  - LEO083 - LEO126: sibling in KIN, contradicts the half-sibling-ness between LEO068/083
  - LEO092 - LEO126: avuncular in KIN, contradicts the half-sibling-ness between LEO068/092

We propose a levirate mating strategy where the mother of LEO126 mated with the unsampled father of LEO038/083/092 produced LEO038/083/092, as well as with LEO68 (son of the unsampled father with the other female partner) produced LEO126. In such case, LEO126 is in 1.5-degree relatedness with LEO038/083/092, as half-siblings resulted from the same mother mating with two males in father-son relationship.

- Exceptional pairs:
  - LEO133 - LEO023/094/072: avuncular in KIN. There are two possibilities in this avuncular relationship - either LEO133 being nephew or LEO133 being uncle to LEO023/094/072. Considering the radiocarbon dates of LEO133 (7th-8th CE) and LEO023 (6th-7th CE), we assign LEO133 as a nephew from a later period.
  - Unit of LEO057 - LEO043/109/137: avuncular in KIN, and LEO043-LEO109/147 being related in 4th/5th degree. This allows three possibilities of relatedness units, due to undetermined generation directionality among avuncular-related individuals. Individual LEO043 is dated to 6th-7th CE, hence we assigned this individual to an earlier generation than LEO109/137. Given that LEO109/137 (G. 145), LEO057 (G.81) and LEO100 (G. 133) have the same type of belt buckle which may even have been made by the same model and LEO109/137 (G. 145) being children under age 10 in terms of anthropology, we assigned LEO057 as an uncle to LEO109/137. As LEO144 (the father of LEO057) is unrelated to LEO043, we assign avuncular/half-sibling relatedness between LEO057 and LEO043 via the side of LEO057's mother, in which case LEO043 is the child from LEO057's mother with another partner or is the sibling of LEO057's mother. Given the later option would push the chronology of sub-pedigree 2 one generation higher than pedigree 1 againsting the archaeological chronology, we opt for the half-sibling relatedness between LEO043-LEO057.
  - Uncertain couple LEO005-LEO133. Given the grandparent-child relatedness of LEO005-LEO074/075/080/114, LEO133-LEO074/075/080/114, the relatedness between LEO005 (female) and LEO133(male) is uncertain. Based on the radiocarbon dates, the dates of LEO005 and LEO133 have one year overlap (774 CE, Supplementary Section X), which is less likely to be evidence of partnership but more likely to point to two grandparents from mother's and father's side separately.
  - LEO034 - LEO038/083/092: avuncular/half-siblings in KIN. This poses three possibilities of LEO034's position in the pedigree: i) being nephew of LEO038/083/092; ii) being uncle to

LEO038/083/092; iii) being half-sibling to LEO038/083/092. As the archaeological chronology implies LEO034 (G. 51) is from a generation lower than LEO038 (G. 56) , we opt for option i) in which case LEO034 is assigned as the nephew of LEO038/083/092.

For Mödling, we report the following exceptional cases as below, and summarize the inconsistent relatedness estimates from KIn, BREADR, and IBD estimates in Supplementary Table 3-4.

We report cases with discrepancies between KIN and BREADR estimates or between mitochondrial (mt) or Y haplogroups as following:

- MGS094 - MGS362: parent-child in KIN while sec in BREADR
- MGS475 - MGS367 (2 years old): parent-child in MGS094
- MGS451 - MGS335: mother - son, draw MGS451 as sec to MGS330, deg3 to MGS096
- MGS297 - MGS206/251/252: siblings, MGS297 has D4 mt haplogroup, different from her siblings have I1a. The mt data quality of MGS297 is low (0.81x), with contamination at 5%.

We find the following pairs have rather different mt or Y haplogroups despite of first-degree relatedness estimated and confirmed by both KIN and BREADR (in which case the related pair should at least share the same mt or Y haplogroups, or both haplogroups):

- MGS154 - MGS490: different Y haplogroups and the Y chromosome data quality is low (0.17x, 0.5x).
- MGS082 - MGS315: different mt haplogroups and the mt data quality of MGS082 is low (0.79x).
- MGS082 - MGS326: different mt haplogroups and the mt data quality of MGS082 is low (0.79x).
- MGS016 - MGS314: different mt haplogroups and the mt data quality of MGS134 is low (0.76x).
- MGS293 - MGS398: different mt haplogroups and the mt data quality of MGS293 is low (0.5x).
- MGS293 - MGS401: different mt haplogroups and the mt data quality of MGS293 is low (0.5x).
- MGS293 - MGS472: different mt haplogroups and the mt data quality of MGS293 is low (0.5x).
- MGS171 - MGS306: different mt haplogroup and the mt data quality of MGS171 is low (0.76x).
- MGS171 - MGS328: different mt haplogroup and the mt data quality of MGS171 is low (0.76x).
- MGS288 - MGS415: different mt haplogroup and the mt data quality of MGS288 is low (0.62x).
- MGS144 - MGS300: different mt haplogroup (H1a and H5), which both derive from H.
- MGS330 - MGS420: different Y haplogroup and the Y chromosome data of MGS330 is low (0.021x).
- MGS096 - MGS330: different mt haplogroup and the mt data of MGS330 is low.

In the main pedigree of Mödling, there are two small family units plotted twice as their matri- and patri-lineal lines appeared in different parts of the pedigree. Noticeably, our reconstructed pedigree here preserves both their patrilineal and matrilineal ancestral lines. As shown below, for the pair of father-son (MGS357-MGS086), we have successfully reconstructed the patrilineal line in sub-pedigree 1_9, and the matrilineal line in sub-pedigree 2_4. For the parents-daughter unit (MGS292-MGS061-MGS432), we have successfully reconstructed the patrilineal line in sub-pedigree 2, and the matrilineal line in sub-pedigree 2_3.

We have obtained relatedness estimates from various methods, including KIN and BREADR (based on pairwise mismatch rate) and we plot the estimated pairwise mismatch rate of pairs containing also the relatively low contaminated individuals in our dataset (the pairs involving these individuals that were used for pedigree construction, are presented below in detail) in Supplementary Fig. 5. As shown, the estimated pairwise mismatch rate of these pairs falls into the range of pairwise mismatch rates across the whole dataset.

There were 4 contaminated individuals in Leobersdorf:

1) LEO086 (female) is the exogamous mother of LEO106 sharing the same mitochondrial haplogroup (K1) with him and this first-degree relatedness is not affected by the contamination rate of LEO086 (7%, lower than 10%, see Supplementary Fig. 5). The son (LEO106) is connected to the main pedigree through the nephew-uncle relations between LEO106 and three sampled uncles (unaffected by the contamination status of his mother), placing hence also LEO086 in this pedigree.
2) LEO123 (child) is a brother of LEO039, sharing the same mitochondrial haplogroup (D4m2a). This first-degree relatedness is not affected by the contamination rate of LEO123 (6%, lower than 10%, see Supplementary Fig. 5).
3) LEO077 (female) is not related to anyone else in the sample dataset and given the 9% contamination rate (Supplementary Fig. 5), it is unlikely that the individual has a first-degree relationship among the analysed samples.
4) LEO065 (female) is loosely attached to the main pedigree via second degree relatedness to LEO110 (male). We caution about this second degree relatedness between LEO065 (7% contaminated) and LEO110, and now mark the individual with an asterisk in the pedigree (Extended Data Fig 4).

Among the contaminated individuals in Moedling (41), 29 individuals are included in the main pedigree based on the presence of first-degree relatedness (25) and the fact that the contamination rate is lower than 10% (see Supplementary Fig. 5). Among these, there is the case of two twins MGS337/MGS029 (clean) and MGS419/MGS326 (clean) where one is contaminated (8%) and still has consistent relationships to the other individuals in the pedigree as the other twin (Supplementary Fig. 5) shows how the estimates are unaffected by the slightly elevated contamination rate.

4 contaminated samples are linked to the pedigree via second-degree related relatives (4):

1) We identify MGS092 as part of the main pedigree based on the estimated second-degree related to four separate sampled uncles (two from mothers' side, the other two from father's side), which is unlikely to be a false positive signal from a cross contamination.
2) MGS004 has the second-degree relatedness to an uncontaminated individual in the pedigree, and logical third-degree relatedness to others in the pedigree. To be secure, we mark that individual in the pedigree as contaminated (Extended Data Fig 5)
3) We are less certain about the MGS229, MGS305, as they have a single second-degree relatedness to the pedigree and it could be that they are in fact only third degree related with a strong cross contamination. We marked these individuals in the pedigree with an asterisk snew (Extended Data Fig 5).

The rest of contaminated individuals (9) had no relatedness to other individuals. The individuals are marked in Table S1 and we can be confident about the absence of close relatedness to the rest of analysed individuals as their contamination rate is below 10% (see Supplementary Fig5).

The contaminated individual in Csokorgasse (1, CKS083) is in second degree relatedness to CSK031, CSK063, which is now marked with an asterisk in the pedigree (Extended Data Fig4).

iv. Division into sub-pedigrees

In Mödling-An der Goldenen Stiege, we have reconstructed a main pedigree with 356 individuals involved, while as we have 447 related individuals in total here based on KIn estimates (including identical individuals), among which 90 individuals remain disattached from the main pedigree (n=44 in small pedigrees, n=47 in distant relatedness).

A main consideration in subdividing the main pedigree was to better understand the complex biological relationships. For this reason, the following subdivision criteria were applied: It was a primary concern to group closely-related individuals together and at the same time to consider the location of the deceased within the burial ground. First of all, the main pedigree can be divided into two large sub-pedigrees (1 and 2), which are connected by the individuals MGS357 (Moe-GST_151) via the male line and the missing female partner via the female line. Sub pedigree 1 can be further divided into a total of 11 sub pedigrees. The starting point for pedigree 1_2 is female MGS353 (Moe-GST_129). This pedigree subsequently includes her child and also all biological kinship connections via her partner. All individuals of sub pedigree **1_2** can be located in the north of the cemetery. The sister of MGS353 (Moe-GST_129) MGS471 (Moe-GST_436) is the starting point of pedigree **1_4**. Here it is her partner and all descendants resulting from this partnership who belong to this sub-pedigree. This group can be located primarily in the west of the cemetery. Sub-pedigree 3 can be further divided into three subgroups. The starting point for the sub pedigree **1_3_0** is the female MGS440 (Moe-GST_213) and subsequently her offspring with the corresponding partners. Closely related to 1_3_0 is the sub-pedigree **1_3_2** via the female MGS312 (Moe-GST_317_A). Her parents and the relationship through another partnership of her mother form this pedigree. Finally, the sub pedigree **1_3_1** is connected via a missing partner in generation 4. Starting point are the siblings MGS283 (Moe-GST_485) and MGS412 (Moe-GST_488) in generation 1. The sub pedigrees 1_3_0 and 1_3_2 can be located in the north of the burial ground, while the deceased of 1_3_ 1 are located in the west. The starting point for the pedigrees 1_5 is the male MGS253 (Moe-GST_440) and his two half brothers MGS154 (Moe-GST_152) and MGS349 (Moe-GST_119_I). MGS253 occupies a special social position within the cemetery with three different partnerships and ten children in total (Table S6). The sub-pedigree **1_5_1** is formed by the partnership with MGS107 (Moe-GST_195) and the resulting descendants. The sub-pedigree **1_5_2** is formed with the partnership to MGS082 (Moe-GST_146) and her resulting descendants. Subsequently two of his female partners MGS237 (Moe-GST_413) and MGS082 (Moe-GST_146) founded the two sub-pedigrees **1_5_3** and **1_5_4** with his two half brothers as their partners (Moe-GST_152 and Moe-GST_119_I). All four sub-pedigrees can be located in the east of the cemetery. Likewise in the east of the cemetery is sub-pedigree 1_6, which can be divided into two subgroups. The starting point of sub pedigree **1_6_1** is the female MGS037 (Moe-GST_074) and her daughter MGS067 (Moe-GST_120). The other children of MGS037 all form independent sub-pedigrees (1_7, 1_2 and 1_4). The two partners (brothers) of MGS067 belong to the sub pedigree **1_6_0**; furthermore, another missing brother of the brother pair. In additional, there are the partnerships of two brothers with the resulting offspring. The starting point of sub-pedigree 1_6_1 is the absent partner of the absent brother in the cemetery. This branch includes twin brothers and a granddaughter of one of her brothers. The starting point of pedigree **1_7** is the male MGS036 (Moe-GST_071). His partner MGS016 (Moe-GST_034) and the descendants up to the seventh generation belong to it. The members of this pedigree are more scattered from the northern to the central part of the cemetery. The starting point of pedigree **1_1** are the siblings MGS425 (Moe-GS_019) and MGS225 (Moe-GST_390). The descendants from the partnership of MGS425 with an unknown partner belong to this sub-pedigree. The pedigree can be localised mainly in the north of the cemetery, with some individuals also lying in the central area. The starting point of pedigree **1_0** are the siblings MGS044 (Moe-GST_082) and MGS442 (Moe-GST_222). The offspring from the partnership of MGS442 with an unknown partner belong to this sub-pedigree. The starting point for the sub pedigree **1_8** is the female MGS365 (Moe-GST_175). Her

two sons and one grandson belong to the pedigree. All burials of this pedigree are found in the middle area of the cemetery.

The pedigree 2 can be divided into a total of four sub-pedigrees. The starting point for sub-pedigree **2_1** is the female MGS099 (Moe-GST_181). All her descendants belong to it, including the next of kin of her granddaughter's partner MGS324 (Moe-GST_505_B). Most of the relatives of this sub pedigree were buried in the south-western part of the cemetery, but some are scattered in the north and east of the cemetery. Three females MGS369 (Moe-GST_186), MGS011 (Moe-GST_028 and MGS366 (Moe-GST_177) with missing partners are the starting points for pedigree **2_4**. Her offspring belong to this pedigree. Connections to the pedigree 2_2 and 2_0 exist via two partnerships of a central missing daughter of MGS369 (Moe-GST_186). The graves of the relatives are located in the south and west area of the cemetery. The starting point for pedigree **2_3** is the male MGS451 (Moe-GST_280). The individuals belonging to pedigree 2_3 are scattered over the central and western part of the cemetery. Sub pedigree **2_2** forms the connection between pedigree 2_3 and 2_4. Therefore, there are also spatial overlaps of these pedigrees in the cemetery, with sub pedigree 2_2 being concentrated in the central and also western part of the cemetery.

The main pedigree of Leobersdorf can be divided into three sub-pedigrees, the third of which is not connected to the others by first-degree relationships. The sub-pedigree 1 can be divided into four further sub-pedigrees. Starting point for the pedigree **1_0** are the siblings LEO029 (Leo-ZP_042) and LEO050 (Leo-ZP_070). Since LEO029 had no descendants in the burial ground, only the descendants from both partnerships of LEO050 belong to the pedigree. In the cemetery, the members of this pedigree lie along the middle of the north-south axis (Extended Data Fig. 4).

The starting point for sub-pedigree **1_1** are the brothers LEO024 (Leo-ZP_036) and LEO144 (Leo-ZP_071). It includes the descendants of LEO024 and those of a non-existent brother. The descendants of the brother LEO144 form the pedigree 1_3. While the members of pedigree 1_1 are also positioned along the central axis of the cemetery and form a group in the northern part, those of pedigree **1_3** lie exclusively in the northern area of the cemetery. The starting point for pedigree **1_2** is the female LEO017 (Leo-ZP_026). Her descendants and those of her brother who are not present in Leobersdorf belong to it. The relatives are scattered all over the cemetery. Sub-pedigree 2 can be divided into three subgroups. The starting points for the sub-pedigree **2_0** are four brothers LEO036 (Leo-ZP_054), LEO138 (Leo-ZP_002), LEO146 (Leo-ZP_057) and LEO056 (Leo-ZP_079_b_D). It includes the descendants and partners of LEO036 and LEO056; also the sister and descendants of LEO055 (Leo-ZP_079_b_C). The graves of sub pedigree 2_0 lie to the west and especially to the east of those of pedigree 1 at the edges of the cemetery. The starting point for sub pedigree **2_1** is the female LEO117 (Leo-ZP_012). The descendants of the partnership of her granddaughter LEO022 (Leo-ZP_035_A) and the descendants of the brothers of her partner belong to this sub-pedigree. As with sub pedigree 2_0, the relatives of this pedigree are located along the western and eastern edges of the cemetery. The descendants of a double partnership of which all three persons are missing in Leobersdorf form pedigree **3**. The graves of this pedigree are located at the south-eastern edge or the northern area of the cemetery (Extended Data Fig. 2).

v. Small pedigrees in Mödling and Csokorgasse

After excluding low quality individuals (n=32) and contaminated ones (n=13) (Supplementary Table S1), for 44 related individuals in Mödling who do not fit into the main pedigree, we built 15 small pedigrees, with each kinship union including from two to ten members (Supplementary Table S4, Extended Data Fig. 6). Among these small pedigrees, there are two pairs of individuals in parent-child relatedness, with

undetermined directionality of parent and child. There are three pairs of adult-child individuals who are in second-degree relatedness, with undetermined type of second-degree relatedness.

For individuals in Csokorgasse, we identified 1 pair of genetically identical individuals, 8 pairs of parent-child relatedness, 5 pairs of siblings, 38 pairs of second-degree-related individuals and 44 pairs of third-degree-related individuals (Supplementary Table S4), based on relatedness estimates from KIN. We finally reconstructed 5 small pedigrees including 3 to 8 members (Extended Data Fig. 4), with 2 pairs of parent-child related individuals with undetermined directionality and 19 pairs of second-degree-related individuals in addition (Supplementary Table S4).

vi. Runs of Homozygosity

We examined genetic consanguinity using hapROH[78]. We employed hapROH to detect genomic segments longer than four centimorgans indicating co-inheritance of identical haplotypes results in stretches of DNA that lack genetic variation. While the majority of sampled individuals in Leobersdorf (139 out of 147), Mödling (450 out of 492, and Csokorgasse (59 out of 83) have biologically related relatives in the site, we do not find individuals with substantial amount (>20cM) of ROH indicating any sign of inbreeding, namely no partnerships of close relatives in the recent ancestral past of these individuals. This is in contrast to the fact that the buried individuals have large amounts of relatedness within and across the sites, suggesting that these individuals have intentionally avoided mating with their related ones as part of their social practice. Noticeably, we found that the amount of ROH among individuals within the Csokorgasse site is nearly absent, whereas in Leobersdorf and Mödling, the detected ROH on average is just numerically low (Supplementary Fig. 6).

There is one exception, an individual MGS143, who has a relatively long ROH segment of 24 cM long on chromosome 1 (colored in red in the legend of Supplementary Fig. 6). However, this is neither observed in his brother MGS142, nor in his grandmother MGS035 (Extended Data Fig. 5). Hence, the long ROH in MGS143 less likely reflects the inter-marriage between closely related relatives, but more likely the relatively low effective population size in Mödling.

ROH is an indication of an offspring from a pair of closely related relatives and also of a society with relatively small population size. Here, we noticed that the ROH distribution in the three studied archaeological sites presents rather different patterns. For comparing the ROH distributions, we carried out two sample Kolmogorov-Sminov test (k-s test), and found out that the ROH distribution between Leobersdorf and Mödling is significantly different, as well as between Csokorgasse and Leobersdorf (Supplementary Table 6), suggesting that sampled individuals at these sites did not belong to the same population. The ROH distributions of Csokorgasse and Mödling, who both have western Eurasian ancestry, are not statistically different (Supplementary Table 6). This potentially reflects differences in population size or even in more distant demographic history in the three cemeteries.

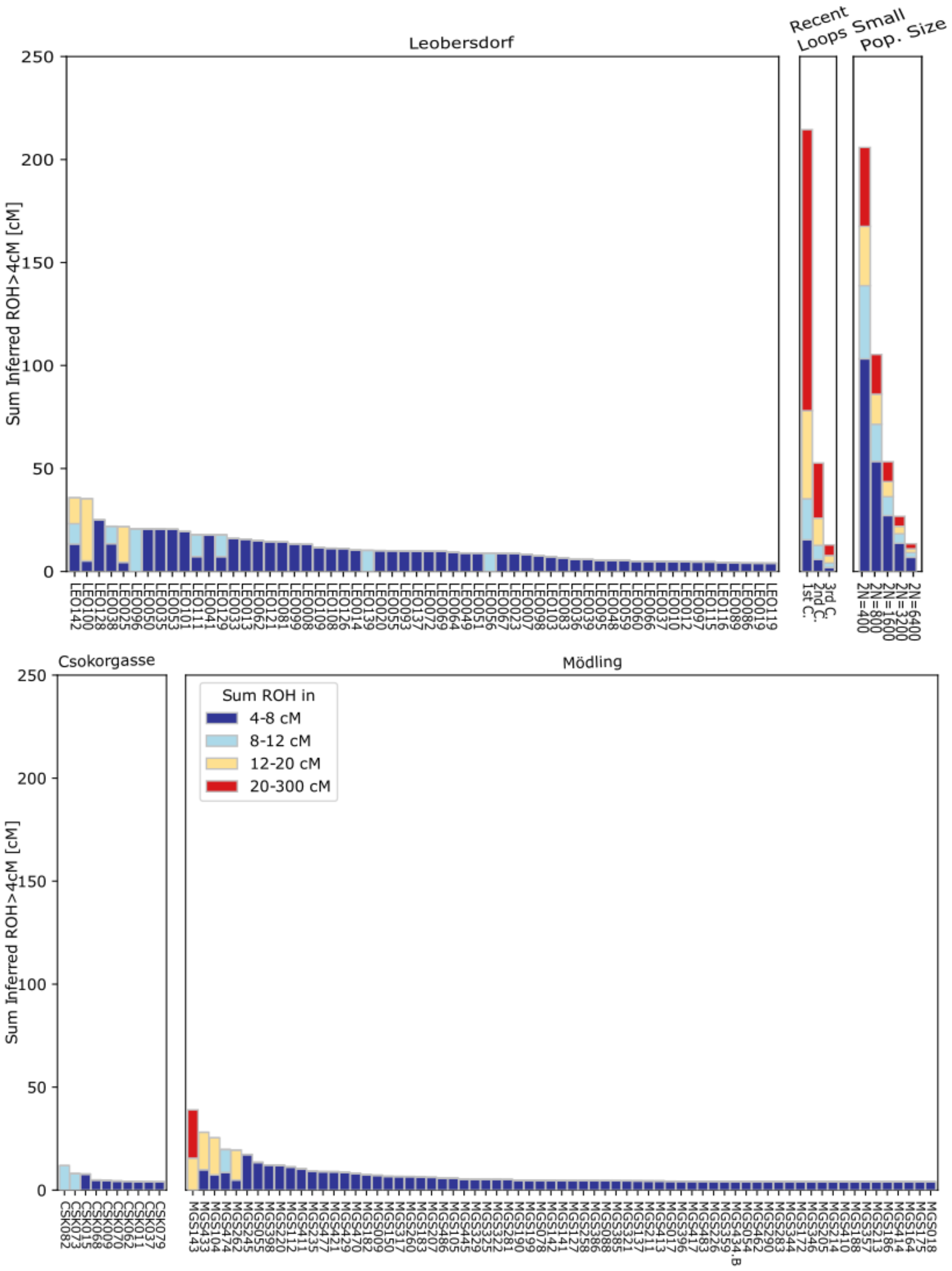

Supplementary Fig. 6. ROH distributions in Leobersdorf, Mödling and Csokorgasse using hapROH (Methods).

5. East Asian ancestry in the Avar realm and its evolution over time

a. Ancestry modeling strategy with proximal sources

We find the genetic ancestry of all sampled Leobersdorf individuals could be traced back to three ancestry sources, namely the ancient northeast Asian ancestry using group “AR_Xianbei_P_2c” from the 1st-3rd CE in Amur River Basin as the proxy[79], the 6th-century central European ancestry using “Hungary_Szólád_6c” from 6th century Hungary as the proxy[2], and 7th-century ancestry from the North Caucasus using “North_Caucasus_7c” as the proxy[80]. Notably, we identified that the Leobersdorf population still preserves a predominant northeastern Asian genetic ancestry component, and to a lesser degree also central European ancestry and gene influx from the North Caucasus.

The genetic makeup of males and females in Leobersdorf cemetery shows differences. We find that the ancient northeast Asian ancestry is ubiquitous in all males of Leobersdorf, while females present higher levels of central European ancestry (D=0.207 and p-value=0.08 in Kolmogorov-Smirnov test, see the proportions of “Hungary_Szólád_6c”) (Extended Data Fig. 1). Meanwhile, the females appear to be less related to each other, compared to males, when zooming into the average relatedness of each individual (see Section 7d). In the sampled 147 individuals from Leobersdorf, only five individuals are unrelated to anyone else in the dataset. Four of them are females (Extended Data Fig. 4) of western Eurasian genetic ancestry with no relatives buried near them or within Leobersdorf cemetery (Extended Data Fig. 4).

b. Distribution of each ancestry component in the Leobersdorf pedigree

As shown in Extended Data Figure 1, we found that the gene pool of Leobersdorf is composed of three ancestry components. By averaging the proportion of each ancestral component, we obtained an average ancestry proportion among individuals in each generation of sub-pedigree 1 and sub-pedigree 2 in Leobersdorf. We found that overall the proportion of East Asian ancestry in sub-pedigree 1 is rather stable over generations, at the level around 70%, while in the sub-pedigree 2, the East-Asian-related ancestry increases from generation 2 to generation 6 (except for the drop between generation 2 and generation 3), accompanied by the drop of Szólád-related ancestry. At the end of generation 6, the East-Asian-related ancestry in sub-pedigree 2 reaches the same level as in sub-pedigree 1. The North Caucasus-related ancestry remains minor over generation in both sub-pedigree 1 and sub-pedigree 2, with no more than 20% on average (Supplementary Fig. 7). Such genetic shit over generations in each sub-pedigree is clearly evident on the Eurasia PCA (Supplementary Fig. 8).

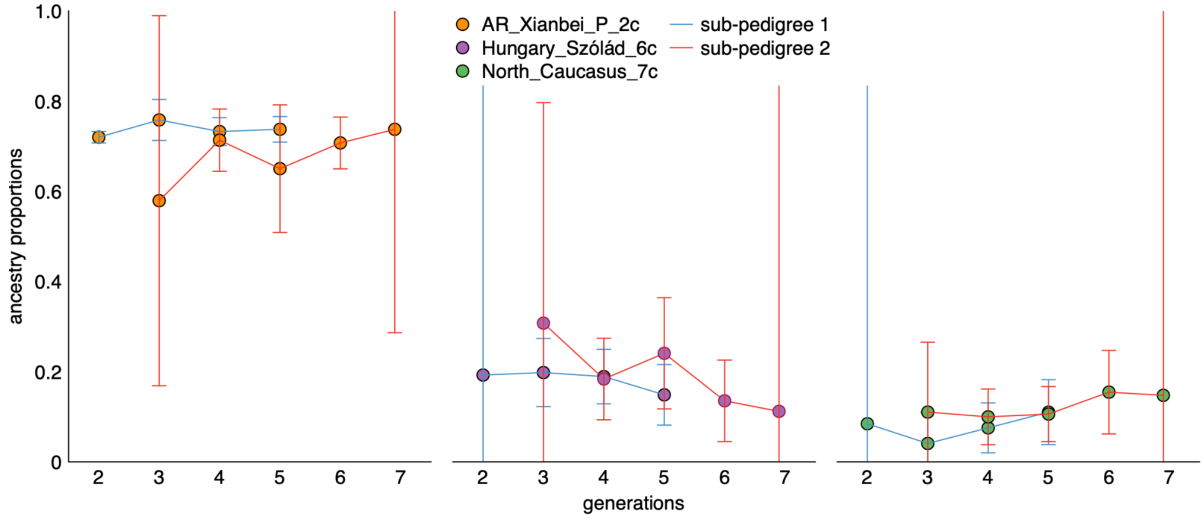

Supplementary Fig. 7. Three genetic ancestry components change over generations in Leobersdorf pedigree. We calculate the average ancestry proportions among individuals in each generation of sub-pedigree 1 and sub-pedigree 2. As sub-pedigree 2 has only a single individual in generation 0 and zero individuals in generation 1, we plot the average ancestry proportions from generation 2 in sub-pedigree 2. The 95% confidence interval estimated from R package Rimsc is plotted for visualising ancestry variations within the generation.

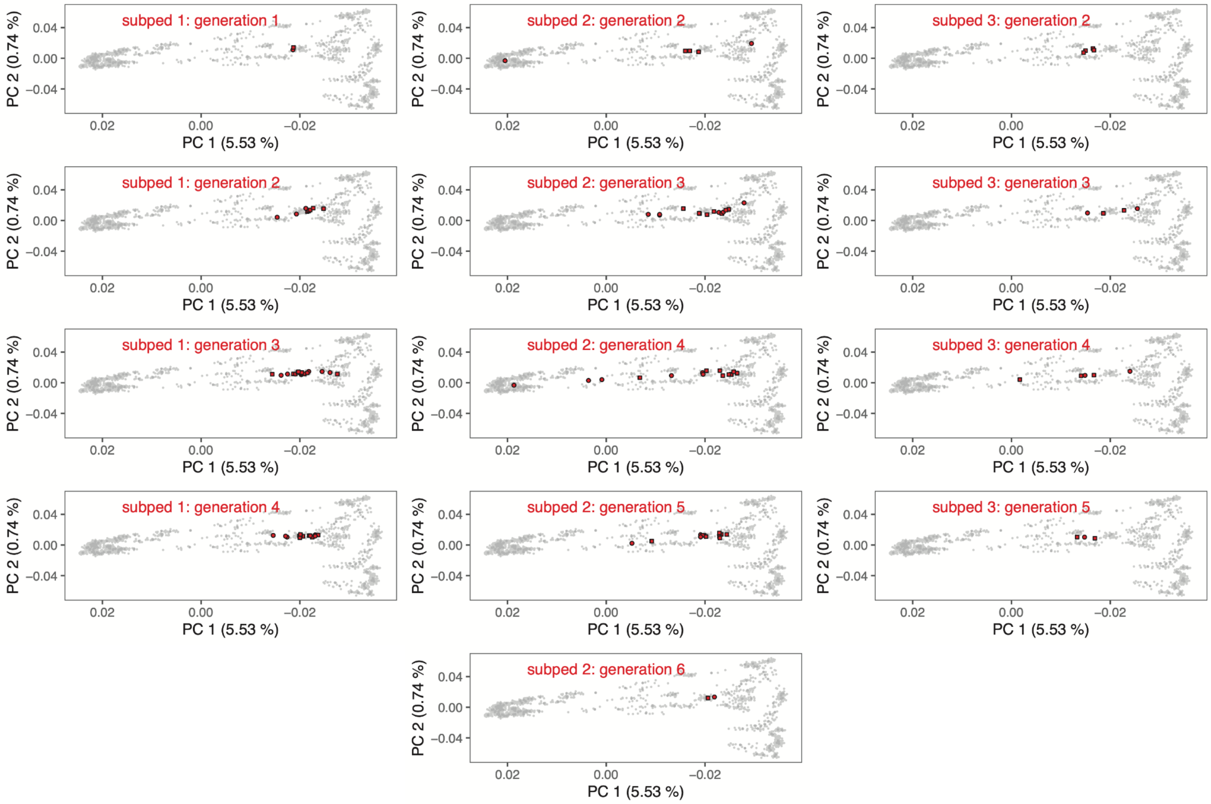

Supplementary Fig. 8. The ancestry shifted over generations in Leobersdorf . We plot the individuals in three sub-pedigrees separately on PCA. We mark individuals in each generation as plotted in the pedigree shown in Fig. 2 and Extended Data Fig. 4. The unrelated ones are marked in unfilled circles and squares.

c. Sex bias and admixture date

A question of sex bias during the admixture process is a contentious issue[81,82]. In the case of Avars, the issue is to what extent the Avar core group that arrived in the Carpathian Basin in 567/68 comprised females who had come along all the way from Eastern Central Asia (see Section 2)[6]. The study that unveiled the Eastern Asian ancestry of the elites of the Avar Empire[4] had mainly analyzed male members of the Avar core group in the Danube-Tisza Interfluve. If only male steppe riders had made their way across the 7000km or more from the Mongolian steppes, the newly-arrived warriors would have had to take wives from the European population. There are in fact historical, albeit later and legendary, records of Avars having children with local females (see Section 2). Yet the fact that the Eastern Asian ancestry had been preserved in Leobersdorf even two centuries after the arrival of the Avar core group from Central Asia can hardly be explained without at least a good number of females with Asian ancestry coming along. To obtain a more differentiated picture, we have investigated the presence of sex bias in the studied communities. Within the site of Leobersdorf, we explored sex bias phenomenon via f_4 statistic in the format of f_4 (Mbuti, per Leobersdorf individual; Ulaanzuukh_SlabGrave, Szólád) on autosomes and X chromosomes separately. As explored earlier, the main

ancestry of Leobersdorf individuals could be traced back to Ancient Northeast Asian-related and Szólád-related ancestry. In the neutrality case, namely no sex bias in terms of gene influx, the f_4 statistics on X-chromosome is expected to be at the same numerical level as the f_4 statistics on the autosomes. In Supplementary Fig. 9, we outlined the expected neutrality line (y=x) in yellow. As the majority of genetic ancestry in Leobersdorf could be traced back to East-Asian-related ancestry (on average at 66%, Supplementary Table S2), we expect the numerical level of f_4(Mbuti, per Leobersdorf individual; Ulaanzuukh_SlabGrave, Szólád) to be negative, given that they share more genetic affinity with East Asians than with Europeans. We found the f_4 statistics on the autosomes are numerically negative as expected, while the f_4 statistics on the X-chromosome appear to be ranging from negative to positive, implying that in multiple individuals the genetic ancestry of X-chromosome share more genetic affinity with Europeans than with East Asians. As the X-chromosome is inherited along the maternal line, the extra shared genetic affinity with Europeans on X-chromosome serves as tentative evidence that the gene flow from Szólád-related into Leobersdorf was likely female-biased. This is consistent with the distribution of mitochondrial haplogroups within the Leobersdorf site (see Section 6), which encompasses much more diversity and at the same time mitochondrial haplogroups typical of Europe (such as haplogroup U4a, U4b, U5a, U5b, T1a, T2b, J1c, K1a, H13a, H46, H10a).

Similarly, we performed such tests for individuals from Avar-related archaeological sites in Danube-Tisza Interfluve (DTI) regions (RK, Rákóczifalva; KFJ, Kunszállás; KUP, Kunpeszér; HNJ, Hajdúnánás[5]. As shown in Supplementary Fig. 9, the Avar-period individuals from DTI regions show substantial deviation from the neutrality line, resembling the sex bias pattern as observed in Leobersdorf of ViennaBasin. Therefore, we conclude that the European-like ancestry in DTI regions and Vienna Basin are both female-driven, implying that mostly men with prevalent East Asian ancestry mixed with females with dominant European ancestry in the Avar communities in the Carpathian Basin and the Vienna Basin.

We estimated the possible admixture date between Szólád-related ancestry and East Asian-related ancestry in Leobersdorf, and DTI sites. As shown in Supplementary Fig. 10, on average the admixture date between East-Asian-related ancestry and European-related ancestry is estimated to be ranging from 4th-7th c. CE for the individuals in the Leobersdorf main pedigree (assuming the settlement of Leobersdorf started from 7th century CE), which on average is 142.1 years (4.9±0.5 generations, assuming 29 years per generation[83], Methods) before the start of Leobersdorf occupation. As for the Alan-related ancestry, we estimated this admixture with East Asian-related ancestry occurred around 300 years before the cemetery age (11±6 generations, Methods).

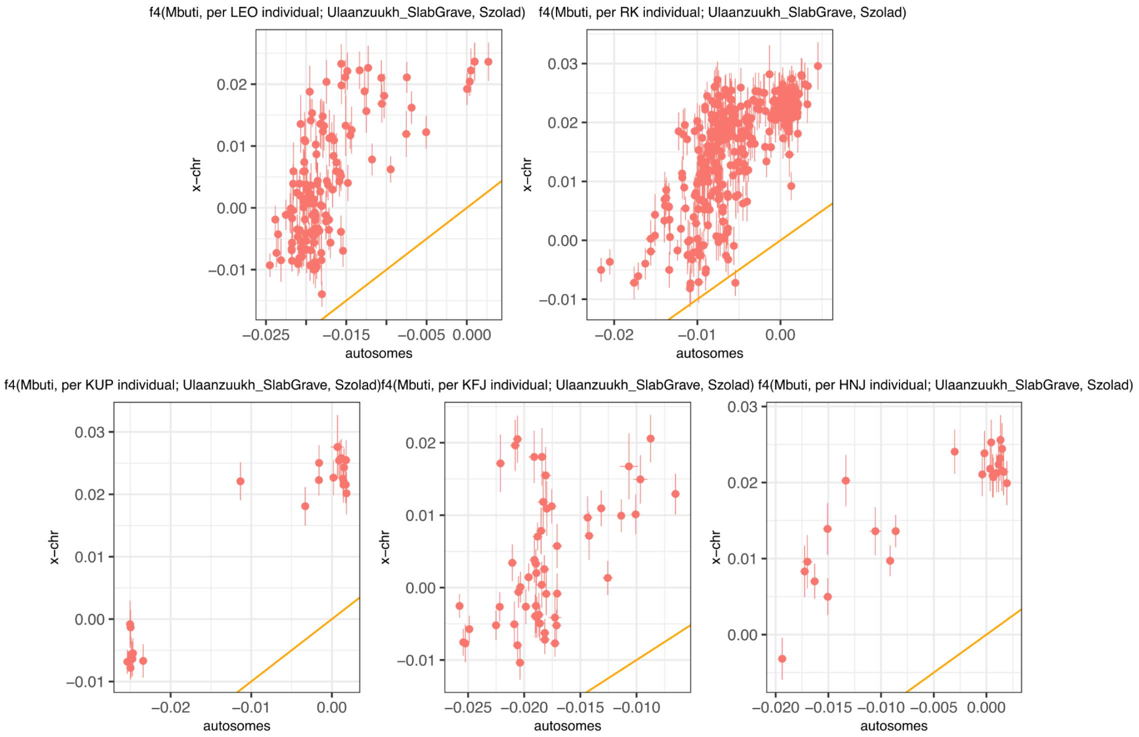

Supplementary Fig. 9. f_4 statistics on autosomes and X-chromosome in format of f_4(Mbuti, per Site individual; Ulaanzuukh_SlabGrave, Szólád). Here, we tested individuals from Leobersdorf (LEO), as well as four Avar-related archaeological site from DTI region, Carpathian Basin (RK, Rákóczifalva; KFJ, Kunszállás; KUP, Kunpeszér; HNJ, Hajdúnánás). We plotted the f_4 statistics for the pairs including more than a total number of 1000 SNPs used for statistics calculation. Bars represent 95% confidence intervals.

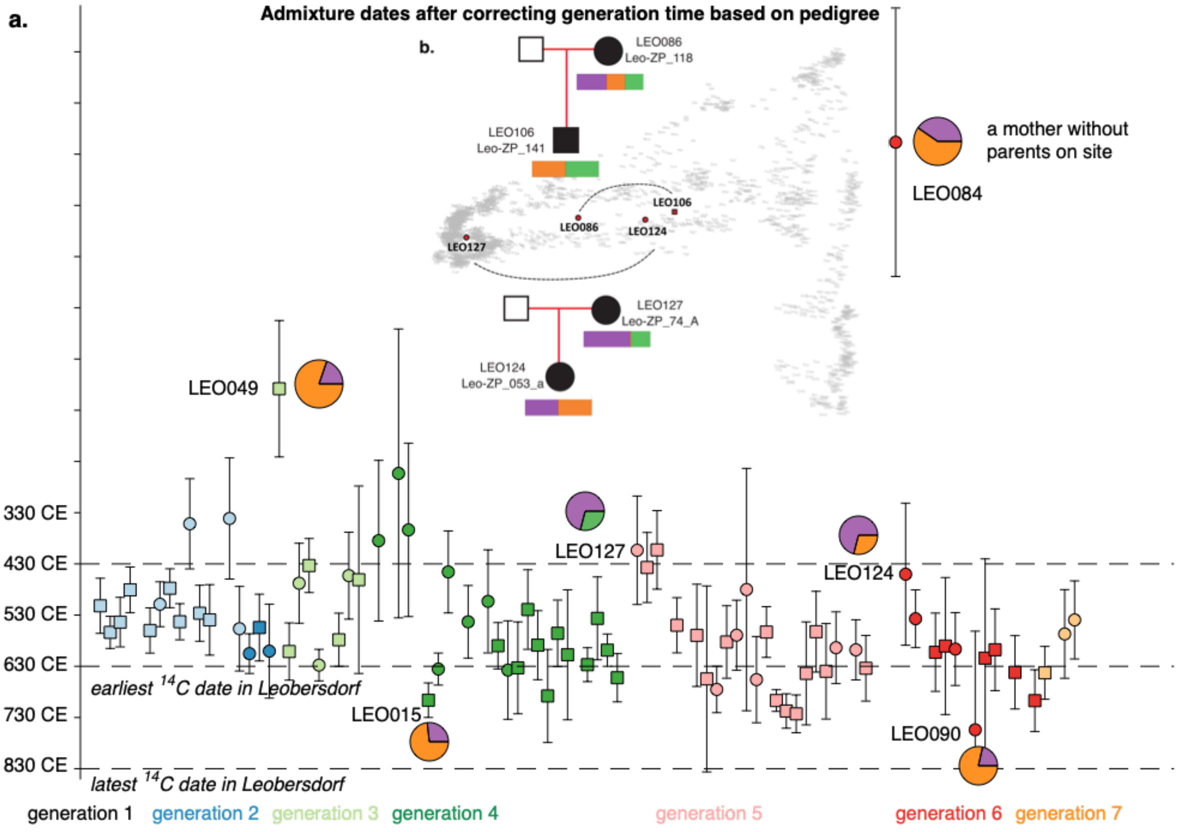

Supplementary Fig. 10. Estimated admixture date for Leobersdorf individuals with non-zero Hungary_Szólád_6c and East Asian (AR_Xianbei_P_2c) ancestry. a) estimated admixture time in Leobersdorf by using Hungary_Szólád_6c as the western source and Ulannzuukh_SlabGrave as the eastern source from software DATES[84]. Squares are males, while circles are females. We use color codes for marking individuals in various generations of Leobersdorf pedigree. Individuals who are not included in the main pedigree or not related at all are marked as undetermined. Vertical standard error (SE) bars are one SE estimated from DATES by the leave-one-chromosome-out jackknifing method. b) example of first-generation admixture for two pairs of parent-child of distinct ancestries. The x-axis shows the Euclidean distance on PC1 among individuals and bar charts illustrate the ancestry decomposition of each individual. LEO086 is an external mother without relatives buried on site, who married into her partner's family though her partner is not buried on site.

6. Genetic origins of Mödling population

a. qpAdm using proximal and distal sources

For the modeling with proximal source (i.e. proximal modeling), we tested following sources: 1) an earlier-phase ancestry in Mödling-Lerchengasse (MLS_4thCE) and in Mödling-Leinerinnen (MLE_6thCE); 2) a northern-European-related ancestry represented by a local source (MLE_6thCE_north); 3) a subset of individuals from Szólád site, central Hungary ("Hungary_Szólád_south", see Supplementary Table 2); 4) ancient northeast Asian ancestry (AR_Xianbei_P_2c, same as the ancestral source for Leobersdorf). We used a list of 11 populations as the outgroup to pull out each distinct ancestry: Mbuti.DG, Anatolia_N, Levant_N, Iran_N, EHG, Iron_Gates_HG, Onge.DG, Ami.DG, Mixe.DG, DevilsCave_N.SG, Russia_Bolshoy. For the modeling with distal sources (i.e. distal modeling), we tested following proxies: 1) a southeastern European

ancestry from Iron Age (Croatia_IA, Slovenia_IA); 2) an early northeastern European ancestry (Lithuanian_BA), which resembles a 9th CE Moravian ancestry (Pohansko[85]); 3) an early western Asian/near eastern ancestry (Anatolia_IA, Anatolia_Hellenistic); 4) ancient northeast Asian ancestry (AR_Xianbei_P_2c). We modeled the ancestry of Csokorgasse with the same set of distal ancestry sources, with very similar results (Supplementary Table 2).

In MGS, the ancestry of nearly half of the population (n=242) is consistent with being proximally decomposed into Hungary_Szólád_south and the few individuals from the local population of "MLE_6thCE" (Supplementary Table 2, Supplementary Information Section 1). The ancestry of others requires additional admixture from the Leobersdorf or the eastern Asian element as found at LEO (n=63). Alternatively there are individuals who do not require any Hungary_Szólád_south-related ancestry but are well modeled as a mixture of MLE_6thCE and an East Asian element (n=54), or simply resemble subgroups in MLE_6thCE (Supplementary Table 2). Though the East Asian element is likely due to the Eastern European ancestry increase seen in MGS sampled individuals and missing in MLE sampled skeletons (see Extended Data Fig. 1), 27 MGS individuals could be well fitted by Szolad_south and Pohansko mixture, and 96 MGS individuals better be modeled with distal sources than proximal sources (Supplementary Table 2).

On the one hand, qpWave analyses of MLS_4thCE and MLE_6thCE showed no direct continuity between either of the sites and MGS, and proximal modeling required additional admixture (Supplementary Table 2, Extended Data Fig. 1). On the other hand, individuals from MGS also show connections to two small earlier pre-Avar Mödling cemeteries via shared Identical-by-descent (IBD) genomic segments - an indicator of some level of direct biological relatedness (more than 100 individual pairwise connections; Supplementary Table 3). Two graves at MGS which were disturbed by later ones (unusual at this well-ordered cemetery) have ^{14}C dates in the 5th/6th c. (see section 3d), and point to earlier settlers at the site. Burial rites common in the 5th/6th c. CE and also found at MGS, such as the deposition of dogs, combs or a glass vessel may also support a thread of local continuity, for which evidence has been rare so far[86].

b. The estimated admixture date between different ancestries in Mödling

For estimating the admixture date in Mödling, we used distal ancestry sources and grouped the Mödling individuals into three sub-groups based on their ancestry decomposition from distal modeling results to maximize the resolution from DATES[84]. For individuals with non-zero Lithuanian_BA ancestry, we grouped these (n=217) into "MGS_Lithuanian_BA", and applied similar grouping to "MGS_Xianbei"(n=162) and "MGS_Pohansko" (n=162). For these three target genetic groups, we estimated the admixture date between Slovenia_IA and the Lithuanian_BA/Xianbei_IA/Pohansko ancestry, as shown in blue dots in Supplementary Fig. 11. The estimated admixture between Anatolia_IA and the Lithuanian_BA/Xianbei_IA/Pohansko ancestry are shown in red dots in Supplementary Fig. 11. Overall, we observed that the appearance of ancient northeastern Asian ancestry in Mödling is within 200 years before the age of Mödling, which is recent and consistent with the estimated admixture date in Leobersdorf. While the admixture date between Anatolia_IA and Lithuanian_BA/Pohansko is estimated to be older though it differed depending on the exact model applied (note that the source populations are unlikely to be fully representative of a full source

affecting these dates and their confidence intervals; Supplementary Fig. 11).

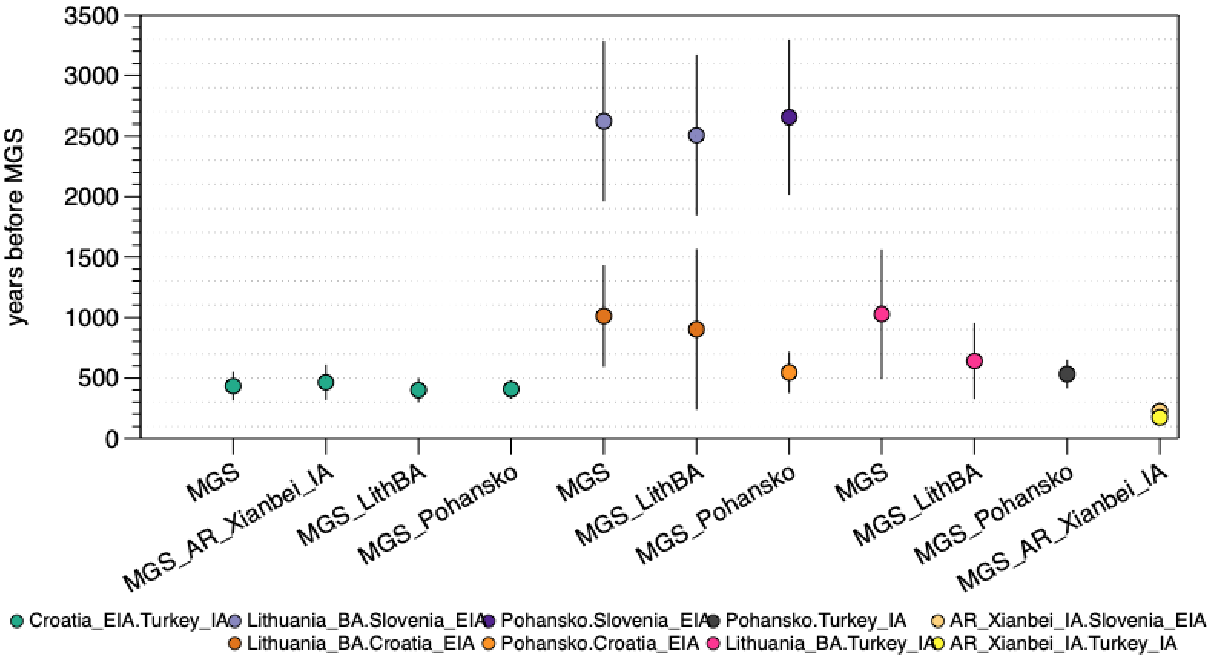

Supplementary Fig. 11. Admixture date for each genetic group in the Mödling sites. The colored dots represent the two-way admixture we tested in the model. We use Croatia_EI/Slovenia_IA as SE European ancestry proxy, Lithuanian_BA/Pohansko as NE European ancestry proxy, and AR_Xianbei_P_2c as E Asian ancestry proxy. We plot one standard error bar here estimated from DATES.

c. PCA and supervised ADMIXTURE analyses

We compiled a high-resolution autosomal reference dataset of microarray SNP data for the purpose of analyzing the fine-scale ancestry of the Mödling individuals in the context of present-day European genetic diversity as described in Gretzinger et al. 2022[87]. Our final reference dataset contained genotypes for 426,135 SNPs (the intersection of several different Affymetrix and Illumina chip types) from 12,176 contemporary individuals sampled from 49 (mostly European and West Asian) populations from previously published datasets[88–102] for following analyses. We carried out principal component analysis using the smartpca software v16000 from the EIGENSOFT package (v6.0.1)[103]. We computed principal components on 426,135 autosomal SNPs and a set of present-day 9,472 individuals from 36 European populations (Extended Data Fig. 1) and subsequently projected ancient individuals using lsqproject: YES and shrinkmode: YES.

The 496 Mödling individuals plot distinctively in between the genetic diversity of present-day Italians and diverse populations from the Balkans and eastern Europe, occupying space along PC1 and PC2 which is not covered by any European group in the dataset (Extended Data Fig. 1). Yet, several outlier individuals cluster closely together with present-day populations from north-eastern Europe and the Baltics, Central Europe, Scandinavia, the British Isles as well as the Iberian and Italian peninsula, indicating a population composition that is to some extent heterogeneous.

To better assess the genetic make-up of the Mödling population, we proceeded to decompose the ancestry of each individual separately by applying supervised model-based clustering implemented in ADMIXTURE[104]. For that, we followed the approach described in Gretzinger et al. 2022[87]. We initially used an unsupervised

approach to identify clusters in 10,216 present-day Europeans, West and East Asians, as well as sub-Saharan Africans on the same SNP set used for the Principal Component Analysis described above. We then selected the present-day populations in which a component was maximized to represent this cluster as a source within the supervised ADMIXTURE setup. Those 13 components are: $FIN_{Finnish}$ (maximized in Finns; n = 606); $CNE_{Continental}$ North European (maximized in Danes, northern Germans, and Dutch; n = 905); $IT_{Italian}$ (maximized in Italians; n = 884); NOR_{Norse} (maximized in Swedes and Norwegians; n = 1910); $WBI_{Western}$ British Irish (maximized in Irish, northern Irish, Scottish, and Welsh; n = 667); $CWE_{Continental}$ Western European (maximized in Spanish and French; n = 812); BAL_{Baltic} (maximized in Russians, Belarussians, Latvians, Lithuanians, and Polish; n = 167).

We further added the following non-European sources in order to also detect Asian and African genetic variation: $WAS_{West\ Asian}$ (approximated by Adygei, Druze, Cypriots and Assyrians; n = 67); $MEA_{Middle\ Eastern}$ (represented by Bedouins, Yemeni, Syrians, Lebanese, Jordanians and Saudi; n = 193); $AFR_{African}$ (represented by Esan, Mende, and Yoruba; n = 200); $SAS_{South\ Asian}$ (represented by Punjabi, Gujaratis, Brahui, Burusho and Balochi; n = 286); $EAS_{East\ Asian}$ (represented by Han Chinese and Japanese; n = 207); $NAS_{North\ Asian}$ (represented by Yakut and Hezhen; n = 28). Subsequently, we modeled each Mödling individual as admixed between these groups at $K = 13$.

We find that the inferred admixture components are highly consistent with the PCA position of the respective individual (Supplementary Fig. 13). Especially, the percentages of CWE (Pearson's product-moment correlation; $t = -10.958$, $df = 454$, p-value < 2.2e-16, $r^2 = -0.4573365$), WAS (Pearson's product-moment correlation; $t = -21.925$, $df = 454$, p-value < 2.2e-16, $r^2 = -0.7171415$), and BAL (Pearson's product-moment correlation; $t = 27.029$, $df = 454$, p-value < 2.2e-16, $r^2 = 0.7853237$) are strongly correlated with PC1 position, and, in the case of BAL ancestry, PC2 position (Pearson's product-moment correlation; $t = 16.311$, $df = 454$, p-value < 2.2e-16, $r^2 = 0.6078595$) (Supplementary Fig. 13).

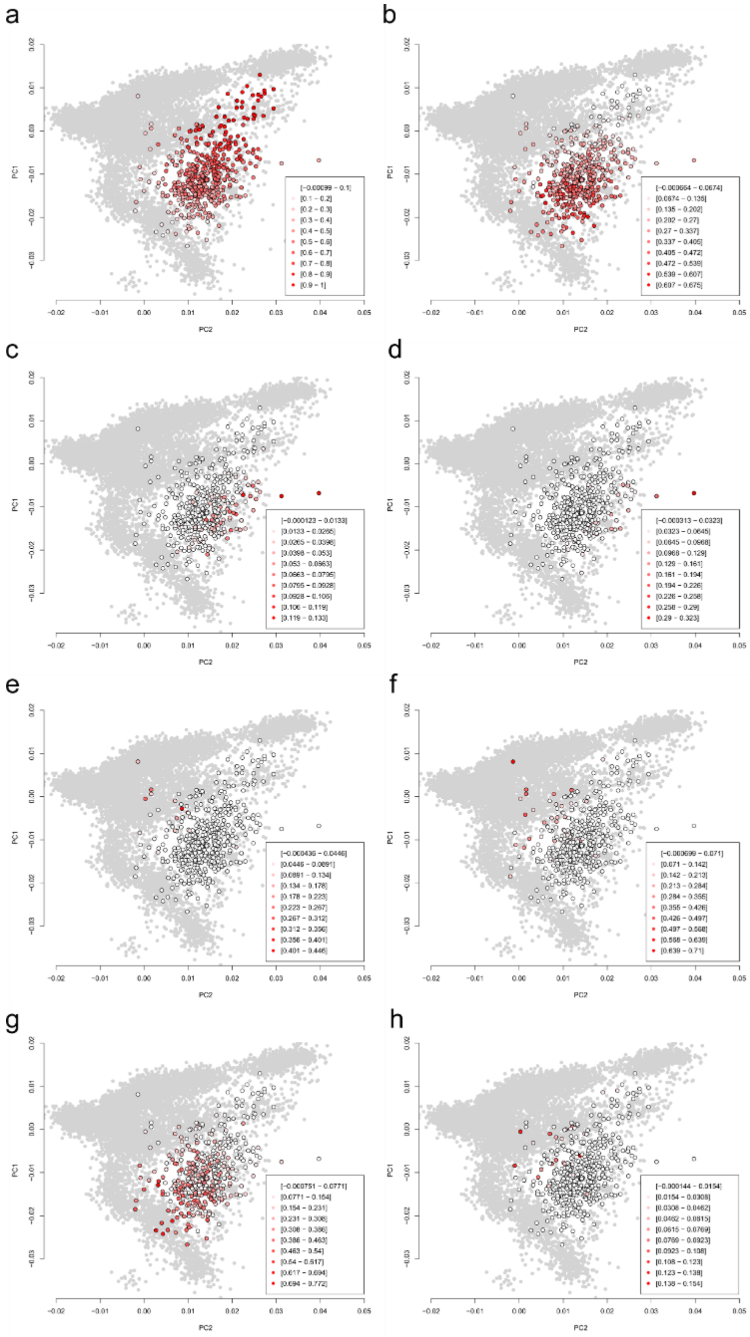

Supplementary Fig. 12. Comparison of PCA coordinates and supervised ADMIXTURE estimates. a) For BAL. b) For WAS. c) For EAS. d) For NAS. e) For CNE. f) For NOR. g) For CWE. h) For WBI.

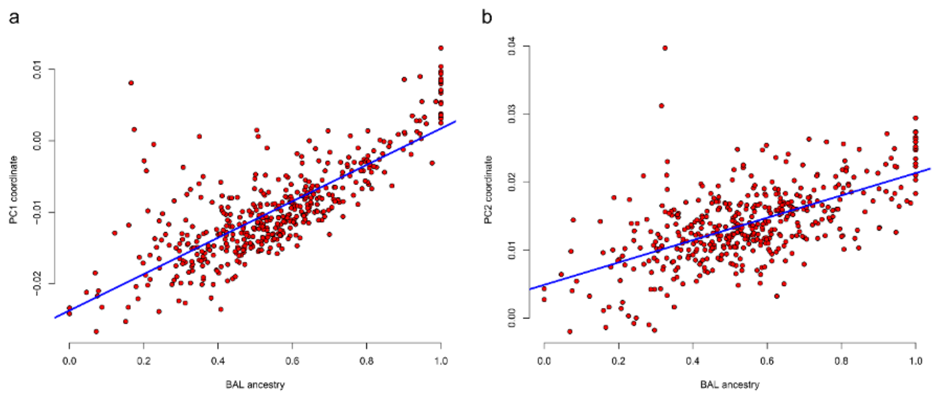

Supplementary Fig. 13. Correlation test between PC coordinates and ancestry. a) Correlation between PC1 coordinates and BAL ancestry. b) Correlation between PC2 coordinates and BAL ancestry.

On average, the Mödling individuals carry 54% BAL-related ancestry, indicating that the majority of their ancestry originated from the Northeast of Europe. As second and third largest components, we inferred WAS with 27% and CWE with 15%, respectively. Northern European ancestry (here the sum of WBI, CNE, NOR and FIN) accounted for less than 3% of the total ancestry. Components from Asia (SAS, NAS, and EAS) similarly represented a minor fraction of the Mödling ancestry (1.3%). Thus, we suggest that the majority of the Mödling gene pool was formed as the admixture between a north-eastern European or Baltic (BAL), a southeastern European source (CWE), and a West Asian source (WAS). The admixture of southern European ancestry with intruding ancestry from West Asia during Imperial Roman times is documented for Italy[105] and Serbia[106], evidencing that ~60% and ~75%, respectively, of the local ancestry was replaced in south-eastern Europe by ancestry from the eastern Mediterranean during that time. A similar process is thinkable for the Mödling population (Western Asian ancestry entering during the Roman period), which was subsequently followed up by a second admixture event introducing northeastern European ancestry, and potentially Y chromosome haplogroup R1a, a haplogroup commonly found in north-eastern Europe[107]. This second gene flow event might be associated with the spread of the Slavic languages and impacted Europe on the continental scale, as evidenced by the arrival of north-east European ancestry in Poland[108], Russia[109] and the southern Balkans[106,110].

d. Contrasting patterns in the Leobersdorf and Mödling cemeteries

i. Ancestry difference

Overall, we observed substantial differences between Leobersdorf and Mödling, including their genetic ancestry, the mixing pattern between their ancestral populations, pedigree pattern, social structure, and possible phenotypes. First of all, the majority of individuals in Leobersdorf derive from East-Asian-related ancestry (Extended Data Figure 1, Supplementary Table 2), and the rest of these sampled individuals derive their ancestry from recent admixture with Szólád-related ancestry (Extended Data Fig. 1, Supplementary Table 2). The genetic ancestry of Mödling individuals is different from Leobersdorf in three aspects: 1) it derives from a prevalently European gene pool; 2) the average genetic distances measured by Fst among individuals within a community of the same archaeological site is smaller, compared to Leobersdorf (Supplementary Fig. 14); 3) Mödling presents a mixing pattern among multiple European ancestries in a deeper time scale (Supplementary Fig. 10). We also examined the possible phenotypic differences that are

typical in different populations, for instance, typical phenotypes in Europeans or Xianbei_IA in Asians, considering the ancestry difference in Leobersdorf and Mödling populations. Based on the pedigree we built in the two sites, we made inferences on the difference in terms of their social structure back then.

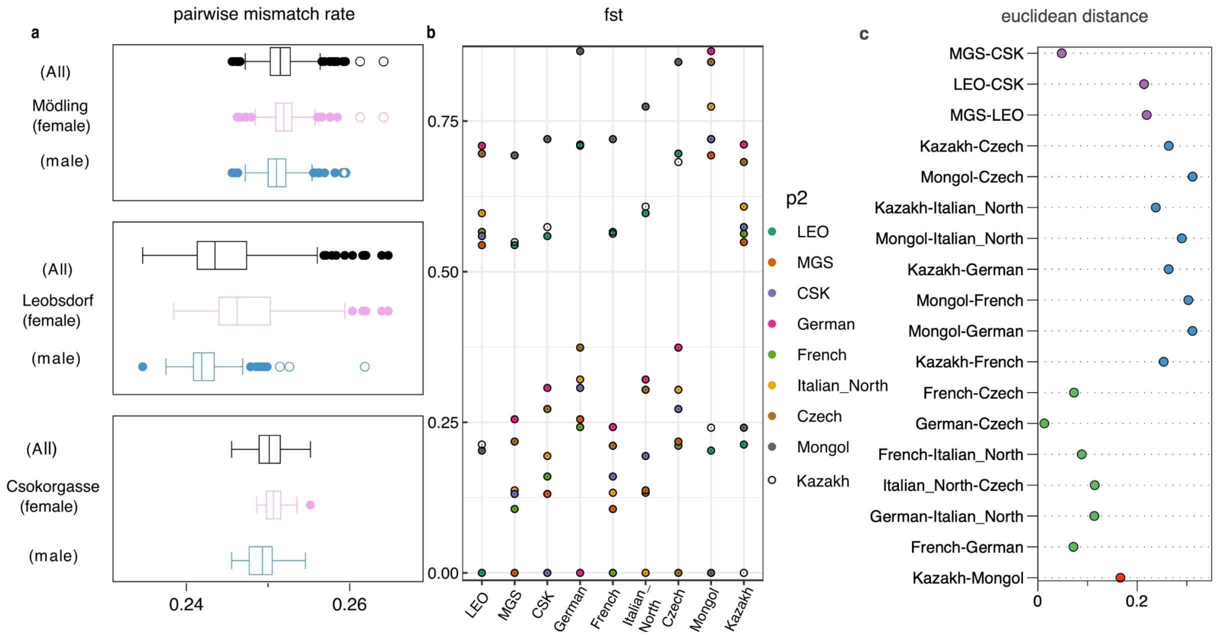

Supplementary Fig. 14. Average genetic relatedness and distances within and between Leobersdorf, Mödling and Csokorgasse. a. Pairwise mismatch rate in each archaeological site. For pairwise mismatch rate (PMMR) calculation**,** we carried out this per-individual calculation based on the mismatch rate between the target individual and everybody else in the subgroup. Here we plot the average relatedness score for three subgroups: all females in Leobersdorf, all males in Leobersdorf, all individuals in Leobersdorf. The smaller the statistic is, the more biologically related the individuals are. The males show closer relatedness to each other than females in Leobersdorf. **b. Fixation index (Fst)**. For Fst calculation, we employed smartpca software with "fstonly" option in the parameter file for assessing the average allele frequency differentiation between LEO, MGS, CSK and the differentiation between modern Europeans (Czech, Italian_North, French, German) and modern Asians (Mongol, Kazakh). **c) Euclidean distance**. We estimated the euclidean distance between LEO, MGS, CSK, and between modern Europeans (Czech, Italian_North, French, German) and Asians (Mongol, Kazakh) on the Eurasian PCA shown in Fig. 2.

ii. Phenotypic marker difference

Given the striking difference in ancestries between Leobersdorf and Mödling, we examined phenotype-informative SNPs, especially those that are contrasting between Europeans and Asians. In particular, we calculated allele frequency at five loci that are associated with shovel-shaped incisor (EDAR), lactase persistence (LCT), alcohol metabolism (ADH1B), and skin pigmentation (OCA2 and SLC2A5), see Supplementary Fig 15.

The shovel-shaped incisor is a typical phenotype among East Asians with the associated high allele frequency in the modern East Asian populations[111]. Interestingly, we found the allele frequency of EDAR in Leobersdorf is above 60% while the allele frequency in Mödling is nearly zero, corroborating the East-Asian-dominant ancestry in Leobersdorf and European-dominant ancestry in Mödling. It should be noted that there are

individuals with relatively high East Asians ancestry among Leobersdorf individuals that do not have this genetic marker. That suggests that while this marker strongly correlates with this ancestry, it is not restricted to it. The range of the expression of this feature at teeth is also very variable, supplementing this result[112].

An allele in the gene ADH1B is associated with adverse reactions to alcohol drinking in East Asians, namely with facial blushing after drinking alcohol, so-called “Asian flush”[113]. While such a phenomenon could also be expected to be among other ‘typical Asian phenotypes’, we found that the allele frequency of ADH1B in Leobersdorf and Mödling is rather equally low (dashed horizontal green line, as shown in Supplementary Fig. 15). The allele of rs1229984 is totally absent in present-day western Eurasia, but is at various frequency levels in different parts of Asia[114]. It has been estimated that the origin of East Asian populations coincides with the time of origin and expansion of agriculture in southern East Asia[115], which might be part of the reason for the low allele frequency in Leobersdorf.

Similarly, for the typical phenotypes in Europeans, such as lactase persistence and skin pigmentation gene, the allele frequency in modern-day Europeans is relatively high, but virtually absent in East Asians. As expected, we found that Mödling has higher allele frequency of lactase persistence (LCT) than Leobersdorf, correlated well with the European-dominant ancestry in Mödling. Despite the allele frequency of LCT being higher in Mödling than in Leobersdorf, the frequency level in Mödling community is still under the allele frequency among present-day central Europeans (blue dashed lines in Supplementary Fig. 15); instead, it is close to the average allele frequency levels in modern southern European populations (26.1%)[116].

As for the European skin pigmentation gene (SLC24A5), we found that the allele frequency in MNCödling is nearly fixed while it also reaches 50% in Leobersdorf, which is unexpected when compared to the zero frequency in present-day East Asian (Supplementary Fig. 15). For the European blue eye color gene (HERC2/OCA2)[117], we found the responsible allele frequency in Mödling reaches over 50%, while in contrast such frequency is maintained at low level in Leobersdorf (Supplementary Fig. 15). Particularly, the allele frequency of the East Asian skin pigmentation gene (OCA2) is similarly low at Leobersdorf and Mödling, in contrast to the medium-to-high allele frequency in present-day East Asians[118]. Such phenomena triggered further thoughts on how the skin pigmentation phenotype was genetically regulated in various populations and how the gene-phenotype correlation has evolved and changed over time in different parts of the world[119,120].

However, we caution that the allele frequency calculated here within the site could be biased, due to the sampling gap remaining in the region and the high biological relatedness within the site. Given that the majority of the sampled individuals are biologically related, it is explainable that they present rather consistent biological phenotypic features. However, whether they represent the genetic diversity of populations settled in the region back then requires further sampling to fill the gene pool.

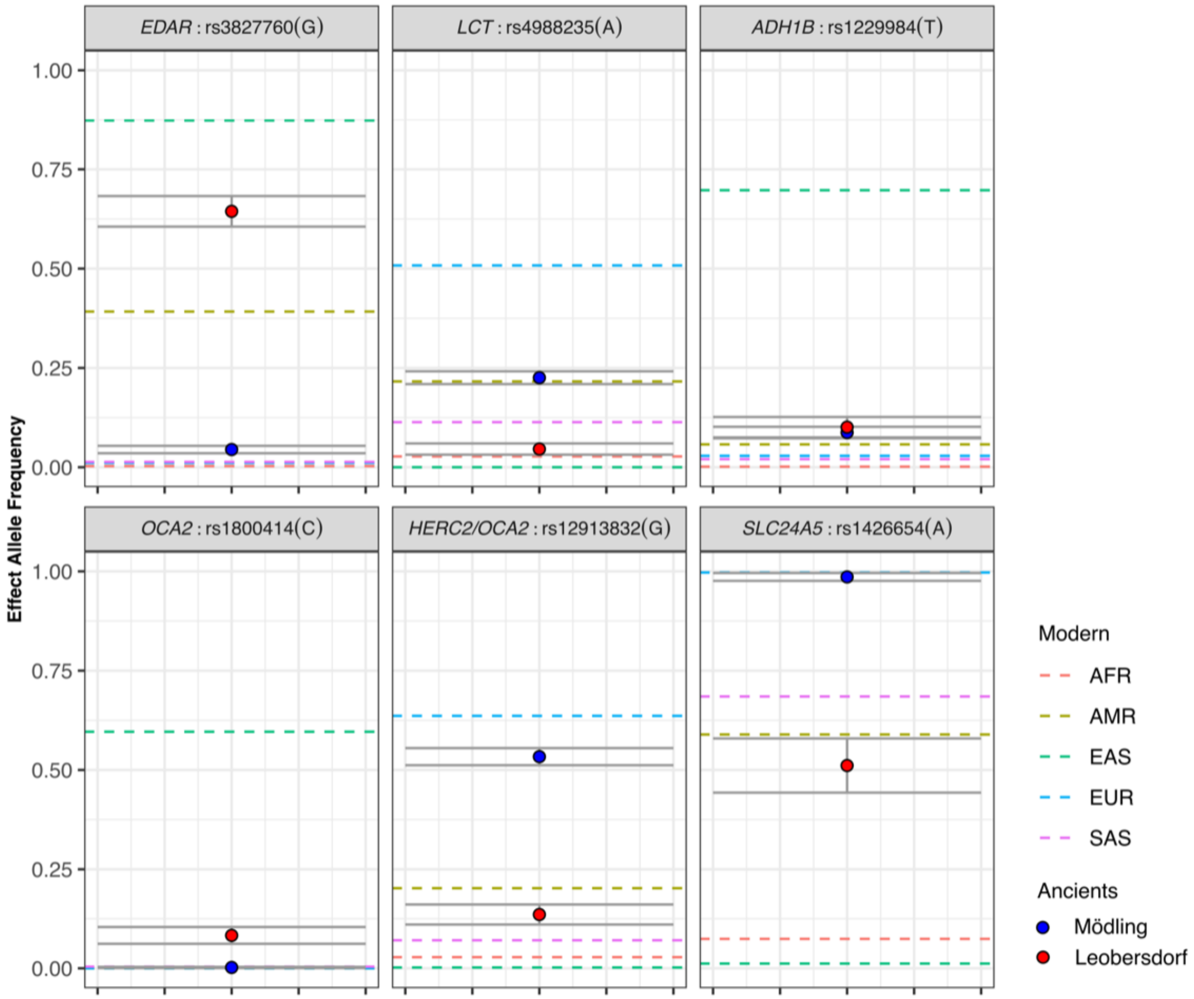

Supplementary Fig. 15. **Allele frequencies of phenotype-informative markers in Leobersdorf and Mödling.** The color-filled dots represent allele frequency calculated from the total number of sampled individuals in the site. The horizontal dashed lines represent allele frequencies calculated from present-day modern populations in 1000 Genomes. Population abbreviations: AFR, African. AMR, American. EAS, East Asian. EUR, European. SAS, South Asian.

iii. Uniparental marker difference

We inferred Y-chromosome haplogroups with two different methodological approaches and compared the results. In the first approach, the Y-chromosomal variants were called from the bam files from samples whose genetic sex was estimated to be "male" or "unassigned" with Samtools v1.9's[121] mpileup and PileupCaller (https://github.com/stschiff/sequenceTools) using the mode "--majorityCall"; Y-chromosome haplogroup assignment was performed using the software yHaplo (https://github.com/23andMe/yhaplo), using the ISOGG panel v.11.349 as a reference (https://isogg.org/tree/; date of access 02 February 2023). In the second approach, Y-chromosome haplogroups were also defined using Y-Lineage-Tracker's subcommand "classify"[122], using as a reference panel the ISOGG Y-haplogroup tree v.15.73 (https://isogg.org/tree/); in this case the input files were genotypes from each individual, estimated using the "allelePresence" method from the ATLAS's (https://bitbucket.org/wegmannlab/atlas/)[123] call tool, accounting for post-mortem damage (PMD) patterns and base score recalibration patterns, estimated respectively with ATLAS's tools "PMD" and

“recal”. For every genetically male individual, close relatives were checked to logically improve the Y-haplogroup calls, as we expect a complete identity of the Y-chromosome of father and male offspring.

The results from the two methodologies were then compared, keeping in account the differences between the two reference panels and the close relative’s estimated Y-chromosome haplogroup. In case the two methodologies’ yielded deeply diverging results (i.e. to the first two ISOGG alphanumeric classification symbols) or were discordant with the estimated reciprocal genetic relatedness between individuals, the haplogroup assessment was further investigated using the software pathPhynder[124] with default options, using as reference the “BigTree'' Y-chromosome data set and the reference phylogenetic tree for sample placement provided via GitHub with the software and as input files the bam files filtered for phred mapping quality > 30. In case of concordance among the results or a deeper call from Y-Lineage-Tracker , the results from Y-LineageTracker (column “Key Haplogroup”) were considered reliable, given the more stringent estimation of the genotypes and the updated ISOGG Y-chromosome phylogenetic tree version. In order to produce the Y-chromosome haplogroup plots in Extended Data Fig. 7 all the haplogroups nomenclature were pruned to the first three characters; haplogroups with less than 3 characters of the ISOGG notation were excluded from the plots. Complete Y chromosome haplogroups can be found in Supplementary Table 1.

Mitochondrial DNA haplogroups were estimated with HaploGrep v2.51[125] using the individual consensus mitochondrial genome produced by Schmutzi[126]. For every individual, close relatives were checked to improve the haplogroup calls, adopting diverging ones only if the original Haplogrep quality was lower than 0.8 and the close-relative one was higher than 0.9. Results of these processes are reported in Supplementary Table 1. In order to produce the mitochondrial DNA haplogroup plots in Extended Data Fig. 7, all the haplogroups nomenclature were pruned to the first three characters; haplogroups with less than 3 characters were excluded from the plots.

As shown in the pedigree (Fig 2, Extended Data Fig. 4), we found that the Leobersdorf site presents a rather patrilocal pattern based on Y haplogroup distribution (Extended Data Fig. 7), where Y haplogroup C2a (C2a1a1b1a2a, C-F9721) dominates the site, with only two individual exceptions (O2a2b1a2a1a1a1, R1a1a1b). This haplogroup is prevalent among modern and ancient central Asians[127]. The only individual with European type haplogroup R1a is unrelated to anyone else in the site (Supplementary Table 1, 2). In contrast, the mitochondrial lineages present much more diversity (Extended Data Fig. 7), encompassing haplogroups prevalent in Europe (e.g. H, J, V, X, T etc.) and Asia (e.g.C, D, U, B, K, T, F etc.). The presence of typical European mitochondrial haplogroups in females is consistent with the female-driven European gene influx and higher European ancestry in females than in males on average in Leobersdorf (Extended Data Figure 1).

Contrastingly, the Mödling site harbors diverse Y haplogroups and mitochondrial haplogroups. The majority of Y haplogroups in Mödling, such as R1a, I2a, T1a, G2a, E1b and R1b, are typical to Europe. Among these, when looking into the most defined sub-haplogroups (Supplementary Table 1, column “Y-Haplogroup final”), we observe lineages that are typically frequent in a diverse range of Western Eurasian regions according to modern DNA and aDNA data from literature, similarly to what observed in Amorim et al. in the sites of Collegno and Szólád[2,97,105,128–132].

We only observe a small minority of Y haplogroups that are mainly observed in Asia: one individual (MGS015) carries a C2a Y-haplogroup (C2a1a1b1a2a, C-F9721), while MGS283’s Y-chromosome haplogroup

was assigned to an N1a sub-haplogroup more frequently observed in Asia (N1a1a1a1a3a, N-F4205) and in previously reported Avar-period cemeteries[5,133,134] (Extended Data Fig. 7).

Although to a smaller degree with respect to the Y-chromosome haplogroups, the mitochondrial haplogroups in Mödling also encompass more varieties as compared to the mitochondrial haplogroups in Leobersdorf, which also corresponds to the higher number of matrilineal lines in Mödling than in Leobersdorf.

e. Potential sex bias in the gene flow into Mödling

To investigate possible sex bias in the gene flow from north-eastern Europe into the Mödling, we compared f_4-statistics[135] based on autosomal loci with equivalent analyses based only on X chromosome loci[131]. For the f_4-statistics, we used qpDstat (v755) from ADMIXTOOLS v3.023 with activated f_4-mode. Significant deviation from zero can be interpreted as rejection of the tree population typology ((Outgroup, X);(Pop1, Pop2)). Under the assumption that no gene flow occurred between Pop1 and Pop2 and the Outgroup, a positive *F*-statistic suggests affinity between X and Pop2, whilst a negative value indicates affinity between X and Pop1. Standard errors were calculated with the default block jackknife 5 cM in size.

We compared two different setups of f_4-statistics for each Mödling individual X to minimize potential bias caused by uneven sampling, missing data, or batch effects. We selected present-day sources based on admixture f_3-statistics based on population pairs that produced the lowest significant *Z* values.

1) A "present-day" setup - f_4(FIN, TSI; X, CHB)- where we used only present-day populations for Pop1 and Pop2. As proxies TSI and FIN from the 1000 Genome project[95] were chosen to represent southern and north-eastern European ancestry.
2) An "ancient" setup - f_4(Mbuti, X; Szólád_south, Slovenia_EIA/Lithuania_BA/MLE_6thCE_north) - where we used only ancient populations for Pop1 and Pop 2. Here, Szólád_south is the proxy for local ancestry, and Slovenia_EIA is the proxy for southeastern ancestry. Lithuania_BA/MLE_6thCE_north represents the proxy for north-eastern European ancestry.

We note that the f_4-statistics based on X chromosomal have wide standard errors in both setups due to the smaller number of loci used, yet f_4 estimates for the autosomes and X chromosome are in both setups. The same is true for f_4 estimates between the "present-day" and the "ancient" setup, both on the autosomes and the X chromosome (Supplementary Fig. 16, 17). In general, both setups provide tentative evidence that introgression of north-eastern European ancestry into the Mödling population was not or only to a small-extent sex-biased.

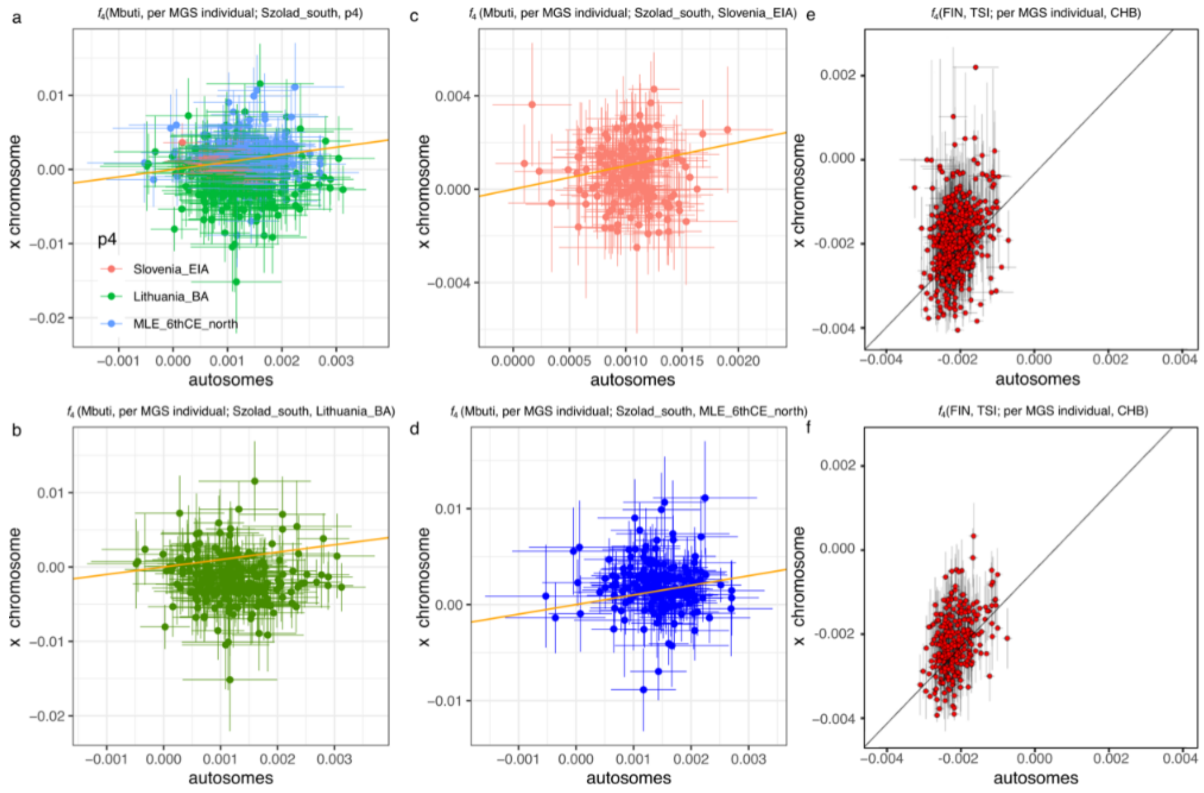

Supplementary Fig. 16. Test sex bias with f_4 statistics. a) f_4(Mbuti, per MGS individual; Szolad_south, p4) for Mödling individuals based on autosomal (x-axis) and on X chromosome data (y-axis), where p4 are Slovenia_EIA/Lithuania_BA/MLE_6thCE_north. b) c) d) are individual plot for each p4 as shown in a). e) f_4-statistics of the form f_4(FIN, TSI; X, CHB) for all 496 Mödling individuals based on autosomal (x-axis) and on X chromosome loci (y-axis). f) same as e) but only for individuals with more than 10,000 overlapping SNPs.

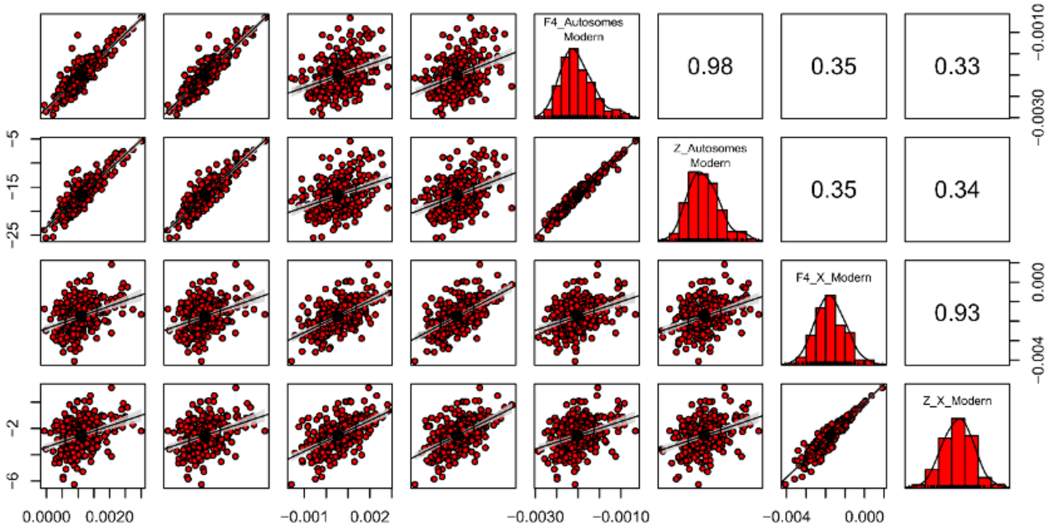

Supplementary Fig. 17. Correlogram illustrating pairwise correlations between f_4-statistics of the "present-day" setup on the autosomes and X chromosome. Pearson's r values for each pairwise correlation test are indicated in the upper triangle of the matrix. Error bands represent ± 2 standard errors.

f. Genetic similarity between Mödling and Csokorgasse

Different from the evident genetic distinction between Mödling and Leobersdorf, sampled individuals from Csokorgasse cluster tightly together with Mödling on the Eurasian PCA (Extended Data Fig. 1). Employing the same ancestry modeling strategy as used for Mödling, we are able to successfully model 70 out of 83 Csokorgasse individuals as a mixture of three distal ancestries: i) a southeastern-European-related source (Slovenia_IA/Croatia_IA as proxy); ii) a western-Asian-related source (Anatolia_IA/Anatolia_Hellenstic as proxy); and iii) a northeastern-European-related source (Lithuanian_BA/Pohansko as proxy)/a northeastern-Asian-related source (Xianbei_IA as proxy), this is similar for MGS where the high-resolution PCA points more likely the former (Extended Data Fig. 1). The three-way combination of SE Europe, W Asia and NE Europe explains the ancestry decomposition of 51 Csokorgasse individuals and 301 Mödling individuals from qpAdm (Supplementary Fig. 18), suggesting a similar genetic profile between Mödling and Csokorgasse with the major ancestry deriving from SE Europe.

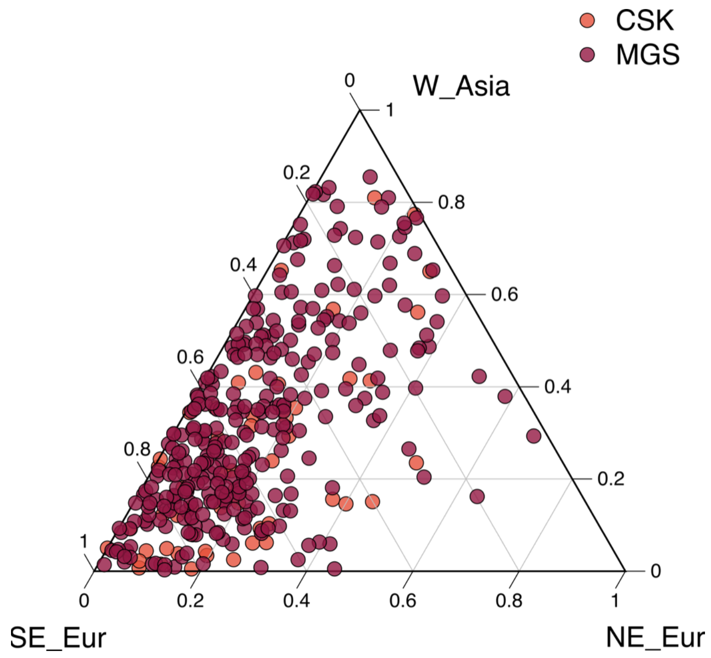

Supplementary Fig. 18. Comparison of Mödling and Csokorgase gene pool in a triangular ancestry setup. W_Asia for western Asian source (Anatolia_IA or Anatolia_Hellenistic), SE_Eur for southeastern European source (Croatia_EIA or Slovenia_EIA) and NE_Eur for northeastern European source (Lithuania_BA or Pohansko). Here, we plot the 3-way qpAdm ancestry model (p-value > 0.05) for 51 individuals in Csokorgasse (CSK), 301 individuals in Mödling (MGS). The raw numbers of each ancestral proportion are reported in Supplementary Table 2.

7. Integrating anthropology, genetics, archaeology and history

a. Anthropological characteristics of the pedigrees

i. The configuration of the pedigrees: age at death, sex, sex ratios, and generations for Leobersdorf and Mödling

The pedigrees constructed for Leobersdorf and Mödling in the present study can here be compared where appropriate to the results of a recently-published article about the cemetery of Rákóczifalva, situated at the Tisza River in eastern Hungary[5]. It allows putting the similarities and differences of the two sites in perspective. The individuals who were present (filled circles and squares), inferred (empty circles and

squares), and also those distantly related and unrelated, were counted for each site (see Fig. 2-3, Extended Data Fig. 4, 5, 6).

The configuration of the pedigrees of Leobersdorf and Mödling is similar in terms of the proportion of present, inferred, not related males as well as females, and also males and females distantly related (Supplementary Fig. 19). One big difference is that there are only four small loose pedigrees in Leobersdorf, one consisting of two infans I brothers, one consisting of a mother and child pair, one consisting of two second degree related females, and one comprising a male and a female in a second degree relation. There is also a very small number of unrelated individuals, consisting mainly of females whilst there are 15 small pedigrees in Mödling observed, including reproductive unions with up to three children, as well as parent-child, siblings and second degree related individuals.

The unrelated group in Mödling is composed of mainly young to young-middle adult females (more than 60 % of this group), a few older females and a smaller group of males of mixed ages (see Section 9). Although in Rákóczifalva similar results were found, present males were proportionally more frequent and thus inferred males less frequent than in the Vienna basin. However, more than double the number of females than males is inferred in the pedigree (Supplementary Fig. 19). The nine small pedigrees in Rákóczifalva consist of small reproductive unions with offspring, but also of some sibling pairs.

Another striking observation is that more than half of the group (58 %) distantly related in Leobersdorf, which is largely made up of women, died when they were young or young-middle adults. In contrast in Mödling, the sex ratio of the distantly related group is rather balanced, and also the age groups are more evenly distributed. Mödling in general shows a much more even age distribution in the pedigree, small pedigree and distantly related groups.

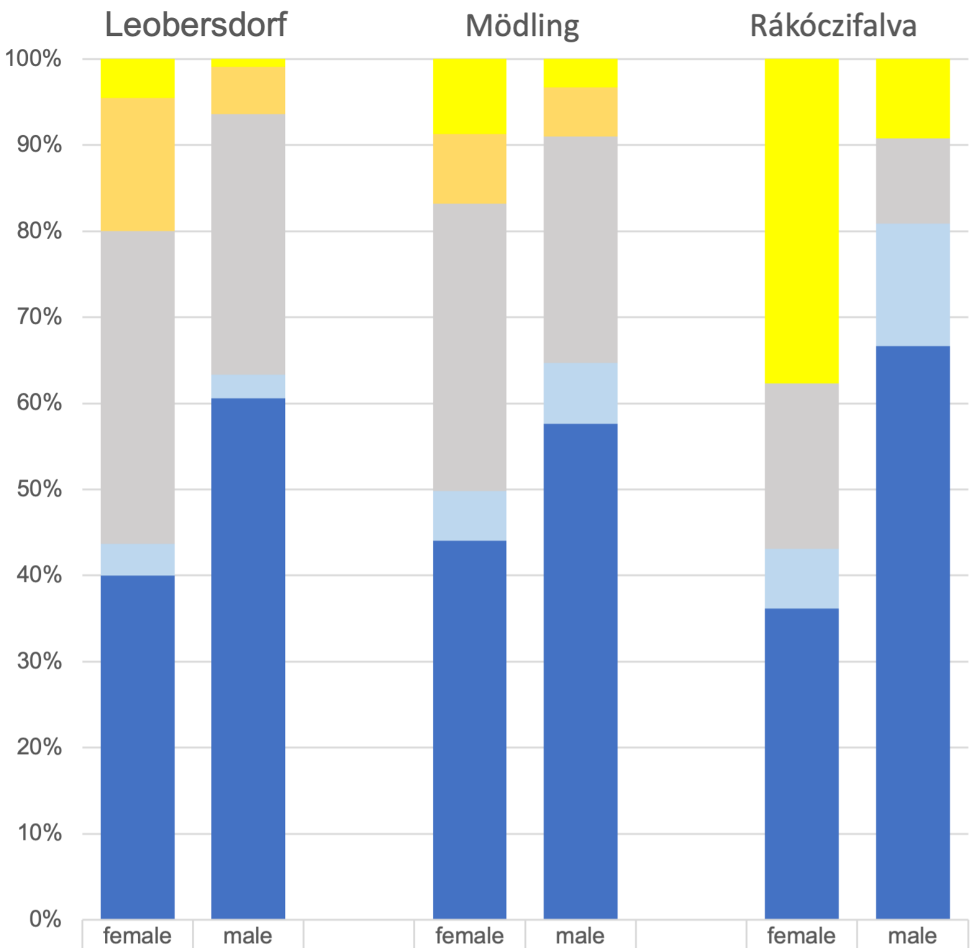

	Leobersdorf female	Leobersdorf male		Mödling female	Mödling male		Rákóczifalva female	Rákóczifalva male
n unrelated	5	1		27	12		49	13
n distantly related	17	6		25	21			
n inferred in pedigree	40	33		103	97		25	14
n small pedigree	4	3		18	26		9	20
n present in pedigree	44	66		136	212		47	94

Supplementary Fig. 19. Comparison of the distribution of male and female individuals present, small pedigrees, inferred, distantly related, and unrelated in Leobersdorf and Mödling, and male and female individuals present, small pedigrees, inferred, and unrelated for Rákóczifalva. Additionally to this in Mödling there are unsexed individuals (neither anthropologically nor genetically sexed): one in the distantly related, ten in the inferred in the pedigree and eight in the present in the pedigree group. In Leobersdorf there is one additional unsexed individual in the present in the pedigree, one in the small pedigree and one in the distantly related group.

ii. Age at death

In both Vienna Basin sites, Leobersdorf and Mödling, around 40% of individuals present in the pedigree (from now on called only "present") died under the age of 18 years (Supplementary Fig. 21). By contrast, in the Rákóczifalva pedigree, there is a very low percentage of children who died up to the juvenile age (c. 20 %) compared to the adults (80 %)[5]. In particular, the proportion of infans I is very low (c. 8 %) compared to

Leobersdorf (17 %) and Mödling (28 %) (Supplementary Fig. 20). When testing only the infans I against all other individuals in the two sites, there is a significantly lower proportion (χ^2 p=0.001) of children up to 6 years in Leobersdorf than in Mödling. In addition, there are considerably more juvenile individuals in the Leobersdorf pedigree than in Mödling and Rákóczifalva. A low proportion of neonates was observed in all three sites but the absence of skeletons of children who died under 1 year of age is a common observation in ancient and historic cemeteries[136]. This difference is probably due to customs regulating the burial of young children, due to differing conditions for the preservation of children's skeletons and/or to children´s graves being more shallow than those of adults (as shown for Mödling and Leobersdorf in Supplementary Fig. 20), and/or cultural practices. Most plausible explanation is that this results from a combination of these factors.

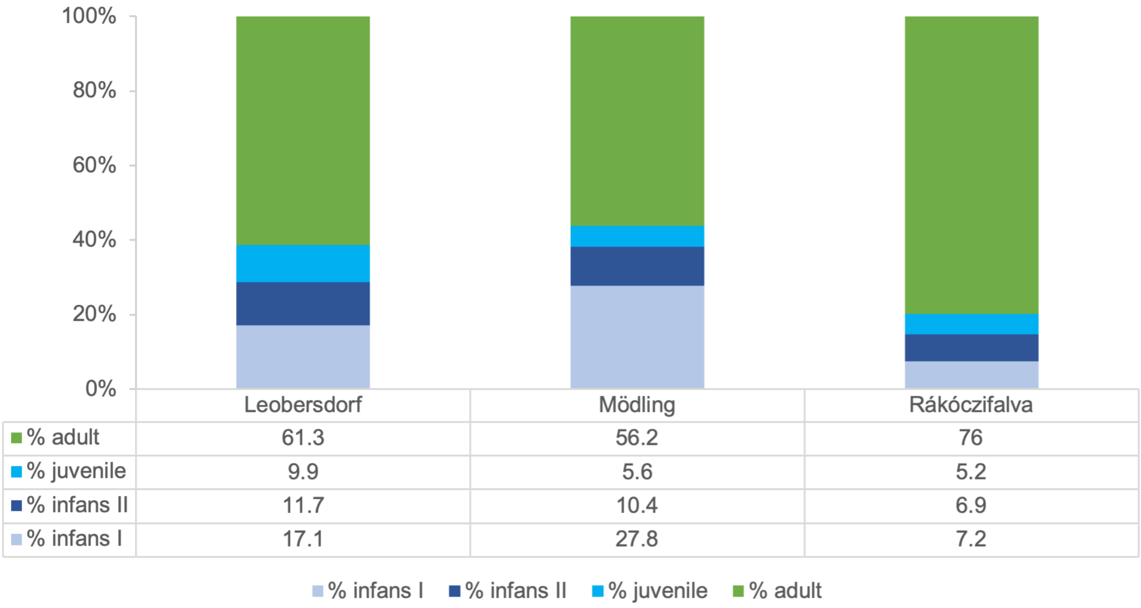

	Leobersdorf	Mödling	Rákóczifalva
% adult	61.3	56.2	76
% juvenile	9.9	5.6	5.2
% infans II	11.7	10.4	6.9
% infans I	17.1	27.8	7.2

Supplementary Fig. 20. Proportion of age groups in the Leobersdorf, Mödling and Rákóczifalva pedigree. Mödling has a significantly higher number of infans I than Leobersdorf, and Rákóczifalva shows the lowest number of infans I individuals.

Regarding the number and proportion of age groups in the pedigrees, small pedigrees, distantly related and unrelated groups of the Leobersdorf and Mödling cemeteries, the latter show a more even age distribution (Supplementary Fig. 21). Noteworthy is the high proportion of young to young-middle adults in the distantly related group in Leobersdorf and in the unrelated group in Mödling (see next chapter).

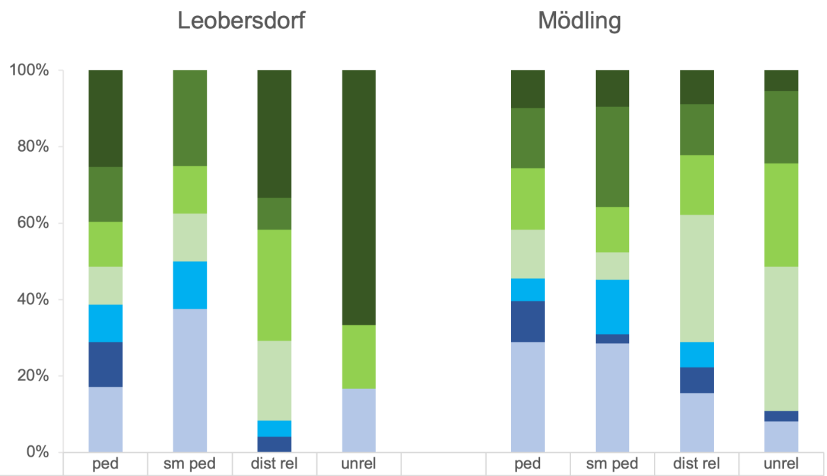

	ped	sm ped	dist rel	unrel		ped	sm ped	dist rel	unrel
n old adult	28	0	8	4		34	4	4	2
n middle adult	16	2	2	0		54	11	6	7
n young-middle adult	13	1	7	1		55	5	7	10
n young adult	11	1	5	0		44	3	15	14
n juvenile	11	1	1	0		20	6	3	0
n infans II	13	0	1	0		37	1	3	1
n infans I	19	3	0	1		99	12	7	3

Supplementary Fig. 21. Proportion of age groups for male and female individuals, in the pedigrees, small pedigrees, distantly related and not related. All age groups are present in the main pedigrees of Leobersdorf and Mödling cemeteries while this is not always the case for the other categories, especially in Leobersdorf. Abbreviations: 'ped' for pedigree; 'sm ped' for small pedigree, 'dist rel' for distant related pedigress, 'unrel' for 'related'.

iii. Sex distribution based on genetic data

From the mean values of age at death we retrieved the following distribution of sex per age groups in Leobersdorf and Mödling (Extended Data Fig. 8).

Within the Leobersdorf pedigree the proportion of infans I girls and boys is about the same, whereas only bones of one neonate were commingled in an adult burial. In contrast, there are relatively more infans I boys than girls in Mödling, including 13 neonates.

There is a significantly (χ^2 p=0.003) higher proportion of juvenile females in the pedigrees in Leobersdorf (15.9 %) than in Mödling (2.9 %). In contrast, the proportion for juvenile males is similar in both sites (Leobersdorf 6.1 %, Mödling 7.5 %). In Leobersdorf, 30.3 % of males died aged 50 years or more compared to Mödling (10.9 %). In sum, there is a much higher proportion of females who died as juveniles, as well as of males who died in old age in Leobersdorf than in Mödling.

iv. Male to female ratios in the Vienna basin pedigrees considering all other groups

If the analyzed cemeteries would represent stable populations, we would expect the ratio of males to females (nM/nF) to be roughly 1:1. However, both in Leobersdorf and in Mödling significantly more males than females are present in the pedigree, which is reflected in the male-to-female ratios of 1:0.67 (χ^2

p=0.0002) and 1:0.66 (χ^2 p=0.0001) respectively (Supplementary Table 8). The sex ratio of the individuals inferred in the pedigree is also comparable between the sites.

Contrary, there are significantly more females in the unrelated groups in Mödling (χ^2 p=0.0006), and significantly more females in the distantly related groups in Leobersdorf (χ^2 p=0.001) as well as in Mödling (χ^2 p=0.002). Despite the small number of individuals in the unrelated group in Leobersdorf, five of six of those are of female sex, but of older age (see Supplementary Table 8). Most of them died during reproductive age, possibly suggesting female mobility (see Section 7d).

Comparing the number of males and females present in the main pedigrees of the Vienna Basin sites with Rákóczifalva, the proportion of males is always higher than that of females, with 77% being the highest in Rákóczifalva. (Supplementary Fig. 22)

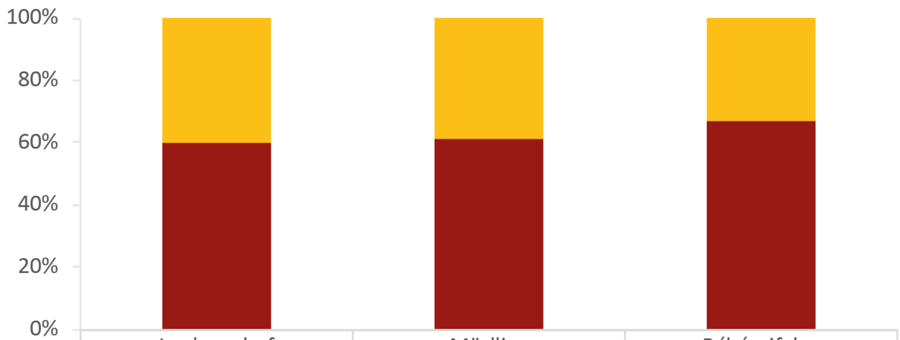

	Leobersdorf	Mödling	Rákóczifalva
n females	44	136	47
n males	66	212	94

Supplementary Fig. 22. Proportional relationship between males and females present in the pedigrees of Leobersdorf, Mödling and Rákóczifalva. The number of present males clearly surpasses the female number in all three sites.

v. General features of the generations as depicted by the pedigrees

Analyzing the number and distribution of individuals present and inferred in the large and small pedigrees clearly shows that the Vienna Basin cemeteries were used for not more than six and seven generations respectively. Comparing the distributions of sex and age at death between the generations allows insights into paleodemography, social structure, and the foundation and abandonment of each cemetery.

1. Age at death distribution between the generations

Although in both Vienna Basin sites the number of individuals buried increases from generation 1 to 4, it grows more intensely and for one generation longer in Mödling (see Fig. 4b, Supplementary Fig. 23). In both sites the largest number of adults died in generation 4; in the following generations, there is a decrease in individuals, and especially adults buried. In both sites, generation 5 seems to represent what is expected of a more stable demographic profile of an ancient population with a high infans I mortality. In fact, children who died as infans I represent about one third of generation 5 in both sites. It is striking that generation 6 in Mödling consists of more than half of infans I and even two thirds when adding infans II. This is not the case in Leobersdorf, where the proportions in generation 6 are relatively similar to generation 5.

Even though there is a large overlap between the generations in terms of age groups, urging for caution in its interpretation, the patterns observed across time in the pedigrees shed light onto the development of the populations of Leobersdorf and Mödling and their burial practices. Of the generations 1 to 3, curiously, almost no individuals died as children - either the children who arrived with the adults were not buried on the site, were not found or survived to adulthood, or, alternatively, mainly adults arrived as founding group at the site (at least first and second generations may have arrived together). During generation 4 there seems to have occurred an intense population growth so that generation 5 roughly represents the expected paleodemographic profile of a stable population, with a high infant mortality (infans I and II). Thereafter, these sites seem to have been abandoned, faster in Leobersdorf than Mödling, since in generation 6 many more children than adults were buried. The last generation in both sites is represented only by subadults. That can only mean that those from the generation who lived longer were not buried at the site any more, because they moved elsewhere or died and were not properly buried.

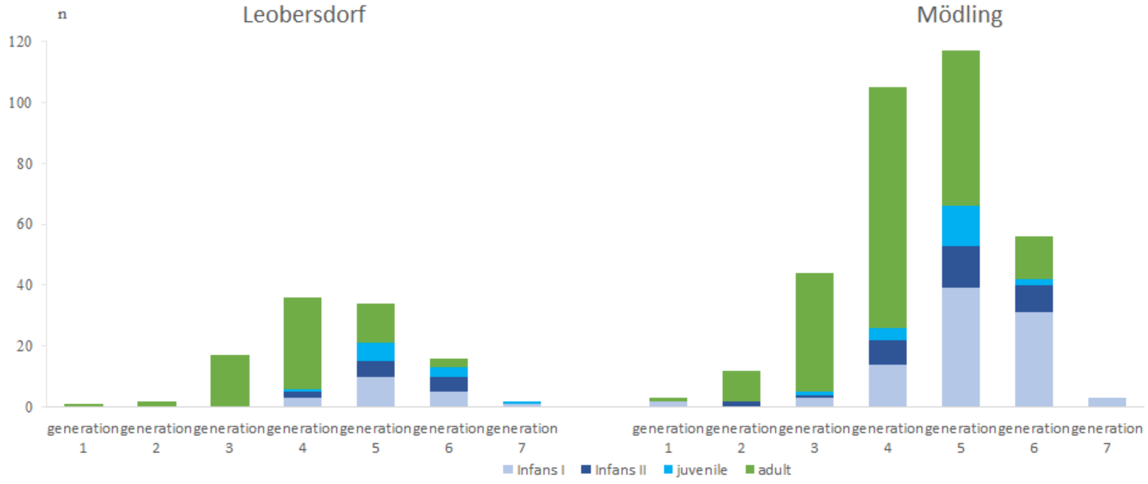

Supplementary Fig. 23. Distribution of individuals present in the pedigrees according to generations 1 to 7 (see pedigrees) divided into age classes (infans I and II, juveniles and adults) in Leobersdorf and Mödling.

2. Sex distribution among adults and subadults across the generations

A more detailed paleodemographic picture is visible when looking at the relationship between sex ratios, age groups and generations.

When comparing the sex distribution pattern between Leobersdorf and Mödling, the most striking result is the lack of females in many age groups and throughout the generations in Mödling (see Fig. 4b). With the exception of generation 4 there is also a lack of females visible in Leobersdorf, especially in the infans II and old adult age groups.

In the first two generations of the Leobersdorf main pedigree the numbers of individuals are too small, precluding further interpretations. The only more reliable interpretations possible for the Leobersdorf main pedigree regarding the sex and age distribution across generations are that: a) in generations 3 to 5, considerably more old adult males than old females (died older than 50 years) are present and that b) there are mostly male children buried on site who died below age 14 in generations 5 and 6 (amongst infans II, there are almost exclusively males, Supplementary Table 8, Extended Data Fig. 7).

If generation 5 is herein considered a stable population, one would expect similar numbers of boys and girls buried in the site. However, in Leobersdorf, there are twice as many boys (n=14) than girls (n=7) below 18y, confirming a reduced number of girls. On the other hand, in Mödling the first two generations are also represented by few individuals, not allowing stringent interpretations. In the generations 3 to 6 there are considerably more males in the juvenile to middle adult groups. Furthermore, in generations 5 and 6 there are also more boys than girls buried on site and died as infans I.

One or more of the following explanations may apply to those missing girls: female infanticide, less care for girls, girls buried elsewhere or even girls abandoning the site. An explanation for the higher number of buried boys could be due to a higher biological risk of boys to die early, or a better care and/or status of the boys in comparison to the girls, when dead.

These patterns in Leobersdorf and Mödling could suggest the following interpretations:
a) different burial treatments according to sex, as less females are buried in the sites;
b) a higher tendency of boys than girls to be buried on each of the cemeteries;
c) the lack of the youngest children could be a consequence of preservation problems and/or a burial practice of shallow graves for the children died;
d) the overall lower number of individuals in generations 1 and 2 could be due to the practice of scattered graves for some of the individuals of the founding group, so that they were not found - it may be that there was no fixed settlement yet, but a more mobile pastoral way of life, so that only part of the group was buried on what should later become the main cemetery of the community; and/or finally
e) the trend that adults are underrepresented in the last generations, females seem to be underrepresented in earlier generations than males (females in the 5th and males in the 6th generation). This suggests abandonment of adults in the last generations of both cemeteries.
f) migrations.

b. Reproductive strategies in Leobersdorf and Mödling in comparison to Rákóczifalva

i. Patrilinearity, matrilinearity and bilinearity

Analysing the pedigrees of the three sites Leobersdorf, Mödling and Rákóczifalva reveals a different picture of the distribution of generation sequences of patrilinearity, matrilinearity and bilinearity (for examples see Supplementary Fig. 24). The highest number of five continuous patrilinear lines applies to both Vienna basin sites, whereas in Mödling the highest percentage of matrilinear lines was visible.

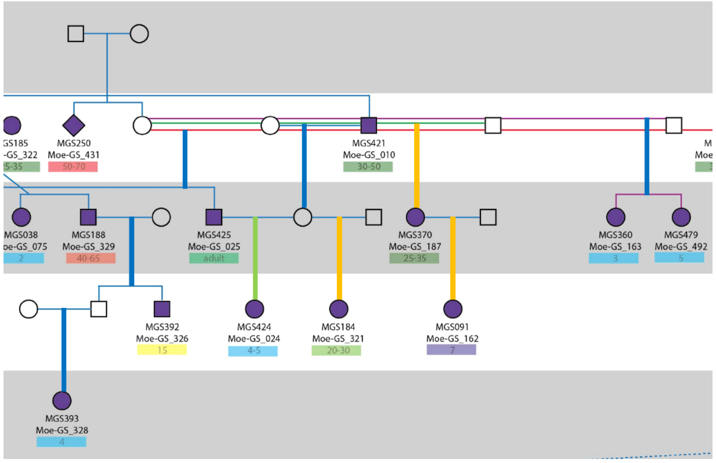

Supplementary Fig. 24. Example for matrilinear (yellow lines), patrilinear (blue lines) and bilinear (green lines) generation sequence in Mödling. Coloured vertical lines indicate the type of linearity.

The pedigrees of all three sites, Leobersdorf, Mödling and Rákóczifalva show that patrilinearity accounts for at least c. than 70% of the reproductive unions (Fig. 4c, Supplementary Fig. 25). Leobersdorf shows a higher proportion of patrilinearity than Mödling (LEO: c. 88%; MGS c. 70%). Regarding the matrilinear lines, the highest proportion of matrilinear reproductive unions (14.4 %) can be found in Mödling, the lowest in Rákóczifalva (2.0 %).

When testing linearity, in Rákóczifalva the proportion of patrilinearity is significantly higher than in Mödling (χ^2 p=0.00004), accounting for as much as 98% of the reproductive unions, but not compared to Leobersdorf. When regarding the number of matrilinear lineages in the two Vienna basin sites, no significant difference occurred. However, when merging the matrilinear and bilinear lineages, and testing them together against the patrilinear lineages, there is a significant difference between Leobersdorf and Mödling (χ^2 p=0.009), and also between Rákóczifalva and Mödling (χ^2 p=0.00004), but not between Rákóczifalva and Leobersdorf (χ^2 p=0502).

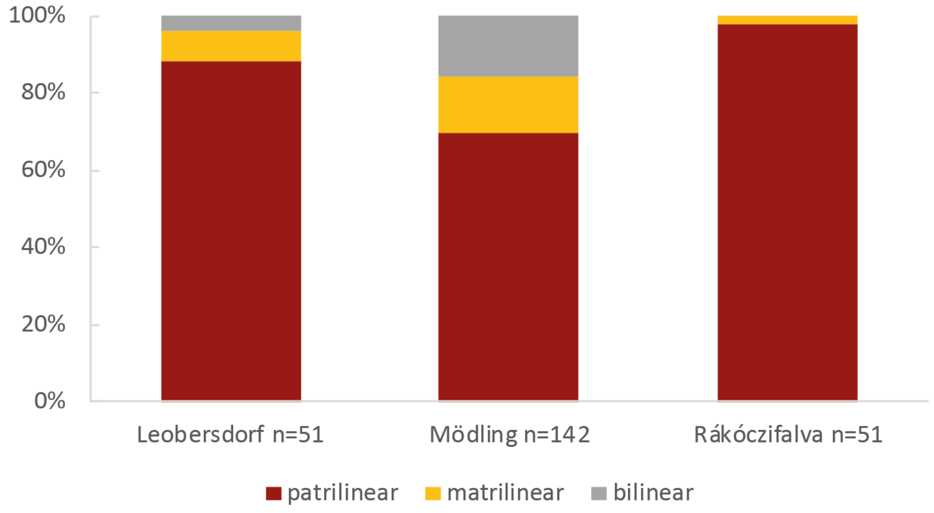

Supplementary Fig. 25. Proportion of patrilinearity, matrilinearity and bilinearity in Leobersdorf, Mödling and Rákóczifalva. Mödling shows significantly more matrilinearity and bilinearity than Leobersdorf and Rákóczifalva, and the latter shows significantly more patrilinearity than both other cemeteries.

Of course, it needs to be stressed that the genetic pedigrees provide no clues to the cultural meaning of patrilinearity or matrilinearity, and can only visualize the shape of biological lineages. The archaeological evidence and the anthropological age categories can provide evidence for the social significance of the genetic links (see section 3.8). It is clear that almost all lineages in Leobersdorf were patrilocal, because most mothers had no ancestors on site. Endogamy within the community was almost completely avoided. This was even more marked in Rákóczifalva, and a bit less so in Mödling. The latter may be due to the fact that the Mödling population was much larger, and it was somewhat easier to find a partner on-site while still avoiding consanguinity. There is no genetic evidence for consanguinity in any of the three sites, and incest in a broad sense was strictly avoided. This allows the assumption that reproduction was regulated by an efficient set of norms and cultural practices, which in turn shaped biological relatedness as we can detect it in the pedigrees. Exogamy also furthered the transmission of cultural features through the exchange of

females between communities in the region and beyond it. In the cases of Leobersdorf and Mödling, it also perpetuated a specific type of ancestry.

ii. Single and multiple reproductive unions

In both Vienna Basin sites c. 60% of the reproductive relations were single, where adults had children with only one person of the other sex (LEO:56.8 %; MGS:59.4 %), while in Rákóczifalva this percentage is considerably lower (41.6 %). In contrast, Rákóczifalva had a significantly (χ^2 p=0.001) higher rate of multiple reproductive unions (including those individuals that are single reproductive persons in multiple reproductive unions, c. 58.4 %), while in the Vienna basin sites this is c. 40 % (LEO 43.2 % , MGS 40.6, Supplementary Fig. 26).

There is no significant difference in the proportion of single- to multi-reproductive unions when comparing between Leobersdorf and Mödling (χ^2 p=0.361), and also between Leobersdorf and Rákóczifalva. In contrast, when testing between Rákóczifalva and Mödling, there are significantly more multiple reproductive unions in Rákóczifalva than in Mödling (χ^2 p=0.006). Interestingly, in Rákóczifalva, in the multiple reproductive unions there is more than one third (34.7 %) are single reproductive persons in multiple reproductive unions, while this percentage is only about 25 % (LEO 24.3 %, MGS 26.5 %) in the Vienna basin sites.

The ratio of single versus multiple reproductive relations is c. Leobersdorf=3:1; Mödling=4:1; Rákóczifalva= 1.5:1 (Fig. 4d).

Both polygyny and levirate unions of widows are well-attested in steppe peoples of the period[137–139]. In the Avar Empire, only the polygamy of the khagan is repeatedly mentioned, but it is not unlikely that it was also practiced within the Avar core group[6]. Obviously, both forms of multiple reproductive partnerships were still practiced close to the Avar core area in the 8th century, as the evidence from Rákóczifalva suggests. For Leobersdorf, the relatively low rate of multiple reproductive unions indicates that this population, in spite of its Eastern Central Asian ancestry, did not continue the Central Asian customs of reproduction on the same level as the community at Rákóczifalva[5]. In this respect, the difference between Leobersdorf and the Avar core area was bigger than with the population of European ancestry in Mödling. As shown below, the tendency towards multiple reproductive unions decreased further in the course of the 8th century.

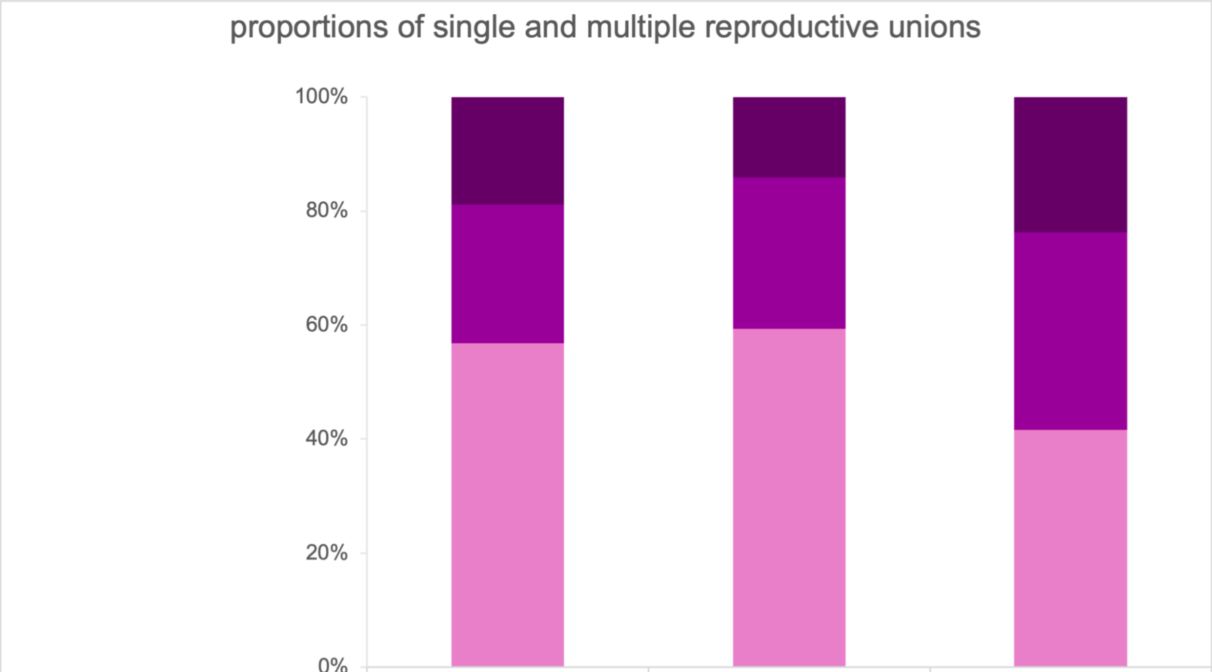

	Leobersdorf	Mödling	Rákóczifalva
% multiple reproductive unions	14	31	24
% single reproductive persons in multiple reproductive unions	18	58	35
% single reproductive unions	42	130	42

Supplementary Fig. 26. Proportions of single and multiple reproductive unions per cemetery, as well as single reproductive persons in multiple reproductive unions. Rákóczifalva shows significantly more multiple reproductive unions, where either a male or a female had offspring with more than one person. The multiple reproductive unions were counted based on the individuals in the multiple reproductive unions, single reproductive unions are based on partnerships.

iii. Are there more males than females in multi-reproductive unions?

Although the proportion of multi-reproductive male persons in Leobersdorf (23.8 %) is more than twice as high as among females (9.5 %), this difference is not statistically significant (Fisher exact χ^2 p=0.1593). Mödling shows similar proportions of multi-reproductive male (12.8 %) and female persons (12.1 %). In contrast, in Rákóczifalva the proportion of multi-reproductive male persons (48.4 %) compared to females (22.0 %) is twice as high, but not significantly different (χ^2 p=0.099). This means that in Leobersdorf and Rákóczifalva about twice as many males than females are multi-reproductive persons, while in Mödling this proportion is similar.

The only significant difference regarding the males is that Mödling shows a significantly lower proportion of multi-reproductive union males (12.8 %) compared to Rákóczifalva (48.4 %, χ^2 p=0.0008), but not compared to Leobersdorf (23.8 %). There is no significant difference in this between Leobersdorf and Rákóczifalva (χ^2 p=0.129).

Among women in Leobersdorf and Mödling the proportion of multi-reproductive persons is similar with around 9-10 % in both (LEO 9.5 %, MDG 12.1 %), whereas it is twice as high (22.0 %, although the difference is not significant compared to the Vienna basin sites) in Rákóczifalva (χ^2 p=0.586).

iv. Is the proportion of multi reproductive unions stable throughout the generations in Leobersdorf and Mödling?

Throughout the generations 3 to 5 (those with the higher numbers of individuals) similar proportions of single and multi-reproductive unions could be found in Leobersdorf and Mödling (Supplementary Fig. 27). In both cemeteries there seems to be a trend of decrease from earlier to more recent generations. However, even at the brink of the abandonment of the sites, after five generations, the practice is maintained, which suggests that this was basically a stable cultural trait.

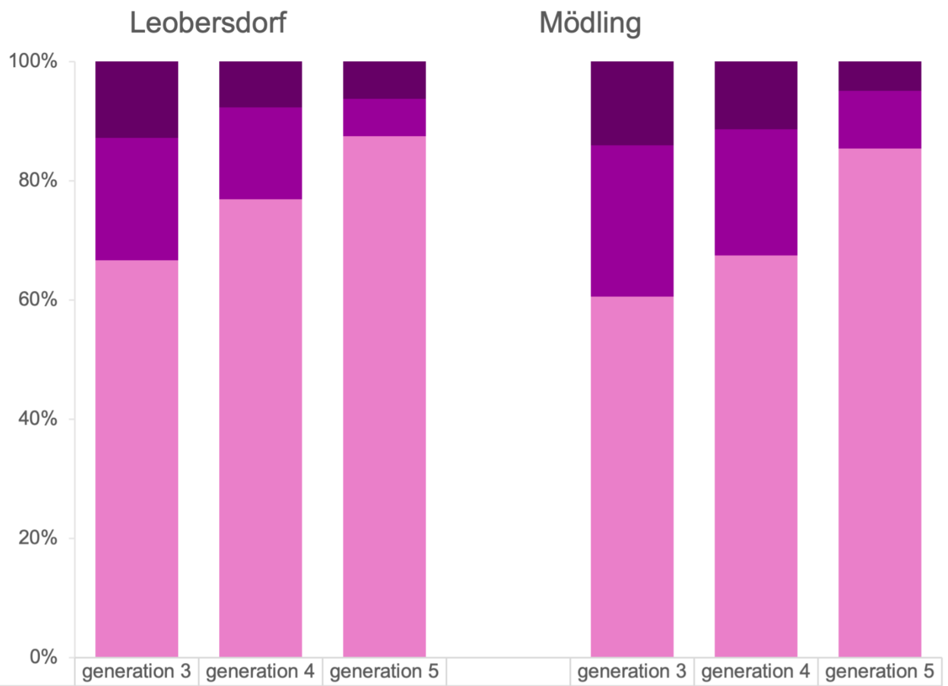

	Leobersdorf generation 3	Leobersdorf generation 4	Leobersdorf generation 5		Mödling generation 3	Mödling generation 4	Mödling generation 5
% multi repro union	5	3	1		10	14	4
% single in multi repro union	8	6	1		18	26	8
% single repro union	26	30	14		43	83	70

Supplementary Fig. 27. Proportion of single reproductive unions, single persons in multiple reproductive unions and multi-reproductive unions across generations 3 to 5 in Leobersdorf and Mödling. Abbreviations: 'multi repro union' for multiple reproductive union; 'single in multi repro union' for single reproductive persons in multiple reproductive union; 'single repro union' for single reproductive union.

v. Special cases of multiple reproductive unions: levirates and sororates

The proportion of levirates respectively sororates visible in the pedigrees is 4.3 % in Leobersdorf (3 levirates and 14 multi-reproductive unions in 56 reproductive relations), 3.6 % in Mödling (5 levirates and two sororates, and 31 multi-reproductive unions in 161 reproductive relations), and c. 5.6 % in Rákóczifalva (5 levirates and 24 multiple reproductive unions in 66 reproductive unions) (Supplementary Table 7). There is no statistically significant difference in terms of levirates between the sites (LEO:MDG Fisher exact χ^2 p=0.371, LEO:RAKO Fisher exact χ^2 p=1.000, MOE:RAKO Fisher exact χ^2 p=0.533). Sororates were only

observed in Mödling. In the Vienna Basin levirates involved brothers of the deceased, half-brothers, a female with her stepson; and quite unusually, a grandson with the partner of his grandfather. The levirate practice is thus present in all three sites, and it does not seem to be restricted to East Central Asian ancestry, although it was not a standard practice, but took on many different forms.

vi. Are the numbers of children per sex and age group comparable between the Vienna basin sites?

In Leobersdorf there are 60 females present/inferred on site in the main pedigree with 118 identified biological children. In Mödling 152 females with a total number of 361 children were identified in the pedigrees. The average number of children per mother in Leobersdorf is 2.0 and in Mödling 2.4 children. However, these numbers do not necessarily represent the true reproductive outcome per female. On the basis of extant Indigenous populations, one would expect about 5 children per female[140].

Most of the present and inferred males and females in the main pedigree of the Vienna Basin died at 17 years or older and had 1-2 children buried in the cemeteries (69.6 %). A proportion of 22.5 % of present and inferred males and females in both sites had 3-4 children. In Mödling, the percentage of males and females with five or more children is slightly higher for females (9.2 % for females and 7.7 % for males) than in Leobersdorf (5.2 % for females and 7.7 % for males). The maximal number of children per individual is 5 for Leobersdorf and 10 for Mödling.

The fact that males in Mödling had more children than females could be explained by the higher proportion of buried males and the more frequent engagement of males in multiple reproductive unions. Of course, for males the reproductive outcome is not as age-dependent as for females.

c. Indicators of regional burial rites, social status and gender

i. Orientations of graves in the cemeteries

Archaeologically significant factors, apart from the number of burials, are location of the site (on a slope [Mödling-An der Goldenen Stiege], in a plain [Leobersdorf-Ziegelei Polsterer], etc.) and positioning of graves (in rows or groups, orientation, etc). In all three cemeteries the graves are arranged in more or less orderly rows. In the Middle Ages, it was a common ritual and/or religious practice to place the body of the deceased in a west-east oriented burial pit, with the head resting in the west and facing east. There are generally four different explanations for the choice of this orientation and the deviations from it [141]: It may be a general (random) eastward orientation; it may follow the ground plans of buildings (churches, if present); it may be an astronomical orientation according to the sun or other celestial bodies; or geographical orientation facing a specific place or point in the landscape. Sometimes regional aspects may also play a role[142,143].

There are apparent differences in the choice of grave orientation between all three sites. The frequency of the two dominant orientations W-E differs significantly between them (LEO:MOE, MOE:CSK χ^2 p=0.000, LEO:CSK χ^2 p=0.013) and WNW-ESE between Leobersdorf and Mödling, and Möding and Wien-Csokorgasse

(LEO:MOE χ^2 p=0.000, MOE:CSK χ^2 p=0.000) but not between Leobersdorf and Wien-Csokorgasse (LEO:CSK χ^2 p=0.306). The burials in Mödling followed the W-E orientation in a higher frequency than those in the cemeteries of Leobersdorf and Wien-Csokorgasse, where WNW-ESE prevails (Supplementary Fig. 28). Only a minority is oriented NW-SE and WSW-ENE, and few exceptions are oriented NNW-SSE, SSW-NNE and SW-NE. The much larger cemeteries of Mödling and Wien-Csokorgasse show a smaller range in orientation than the one of Leobersdorf (Supplementary Fig. 29).

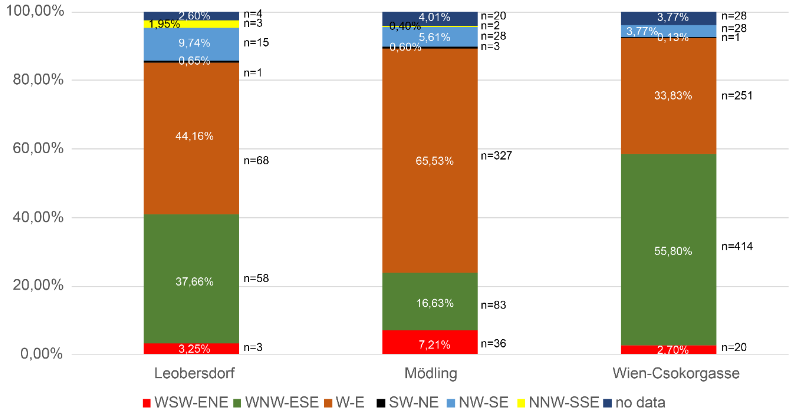

Supplementary Fig. 28. Main orientations of the graves in the cemeteries of Leobersdorf, Mödling and Wien-Csokorgasse. Here we show the cardinal points of grave orientation using abbreviations (N, North; S, South; E, East; W, West).

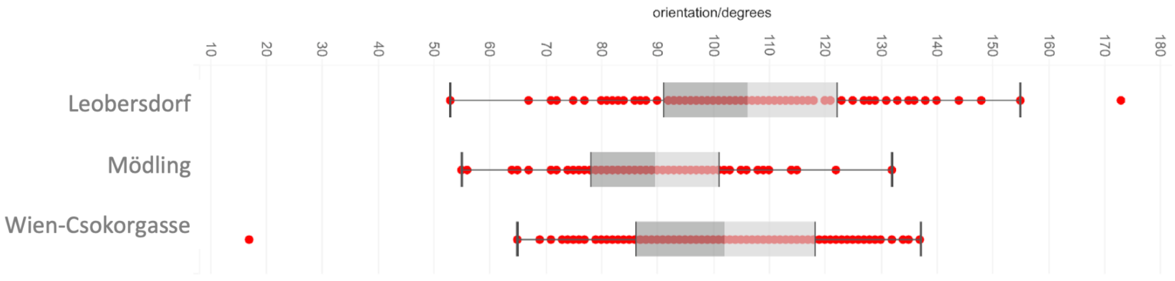

Supplementary Fig. 29. Box plot of orientations (in degree) in the cemeteries of Leobersdorf, Mödling and Wien-Csokorgasse.

ii. Grave volume

How much effort was devoted to the burial provides information about the position of the deceased in society - how they were perceived by their closest relatives, the surrounding community and even beyond? One significant method to assess their esteem in the community is to calculate the volume of the excavated soil for the grave pits. The largest volumes fall on the double and multiple burials, which, as will be explained in more detail below, rarely occur in the cemeteries. On average, the single graves in Mödling are in general significantly more voluminous than those in Leobersdorf (t-test p=0.000). This also applies to biological sex. The difference regarding the grave volumes between males and females between the sites is significant (males t-test p=0.025, females t-test p=0.000): in Mödling both sexes have higher grave volumes. On the other hand, within the cemeteries, the grave volumes do not differ significantly between the sexes (LEO

t-test p=0.223, MOE t-test p=0.085)However, the grave volumes also differ between the age groups (Supplementary Fig. 30). Accordingly, there is almost no overlap in the majority of grave volumes under and over 14 years of age. In Leobersdorf, on the contrary, a clearer area of overlap regarding this can be seen, especially among the male individuals under and over 14 years of age; but there are only few female graves.

Differences can also be shown in relation to the sub-pedigrees. The majority of graves, excluding double, multiple and reused grave pits, of sub-pedigrees 1 in Leobersdorf were dug more elaborately than those of the other lines (Supplementary Fig. 31). Above all, one can see the differences in the graves of the males, but they are also recognizable in those of females and boys in sub-pedigree 1_1. Differences, albeit much smaller, can also be observed in Mödling, depending on the sub-pedigree. For females, the grave volumes in sub-pedigrees 1_1 and 1_5_2 are above average, for men in sub-pedigrees 1_0, 1_4 and 1_8. For some sub-pedigrees, however, the small number of graves must be taken into account (Supplementary Fig. 32).

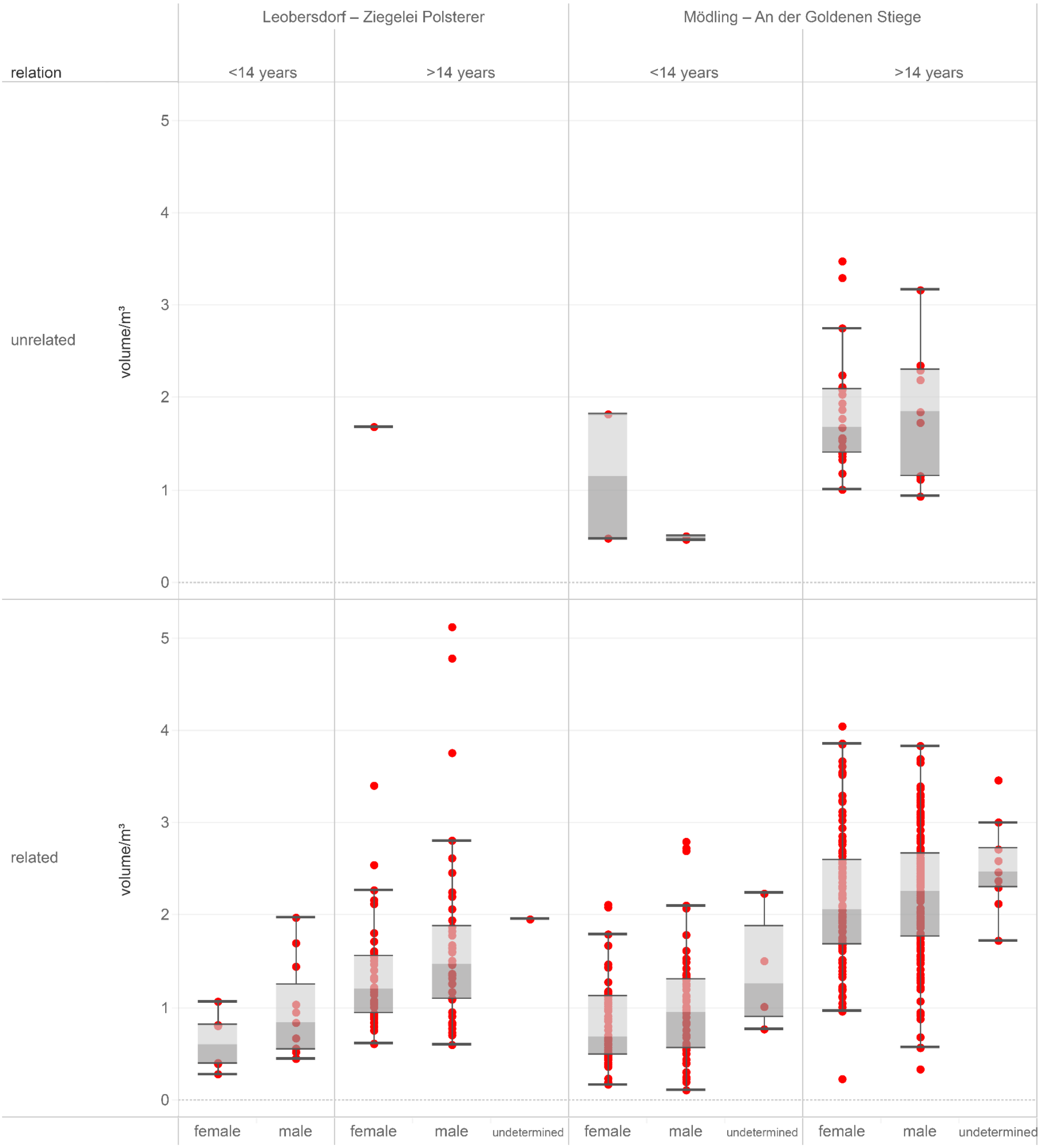

Supplementary Fig. 30. Box plot of grave volumes excluding multiple, double and reused grave pits grouped by sites, sex and age groups

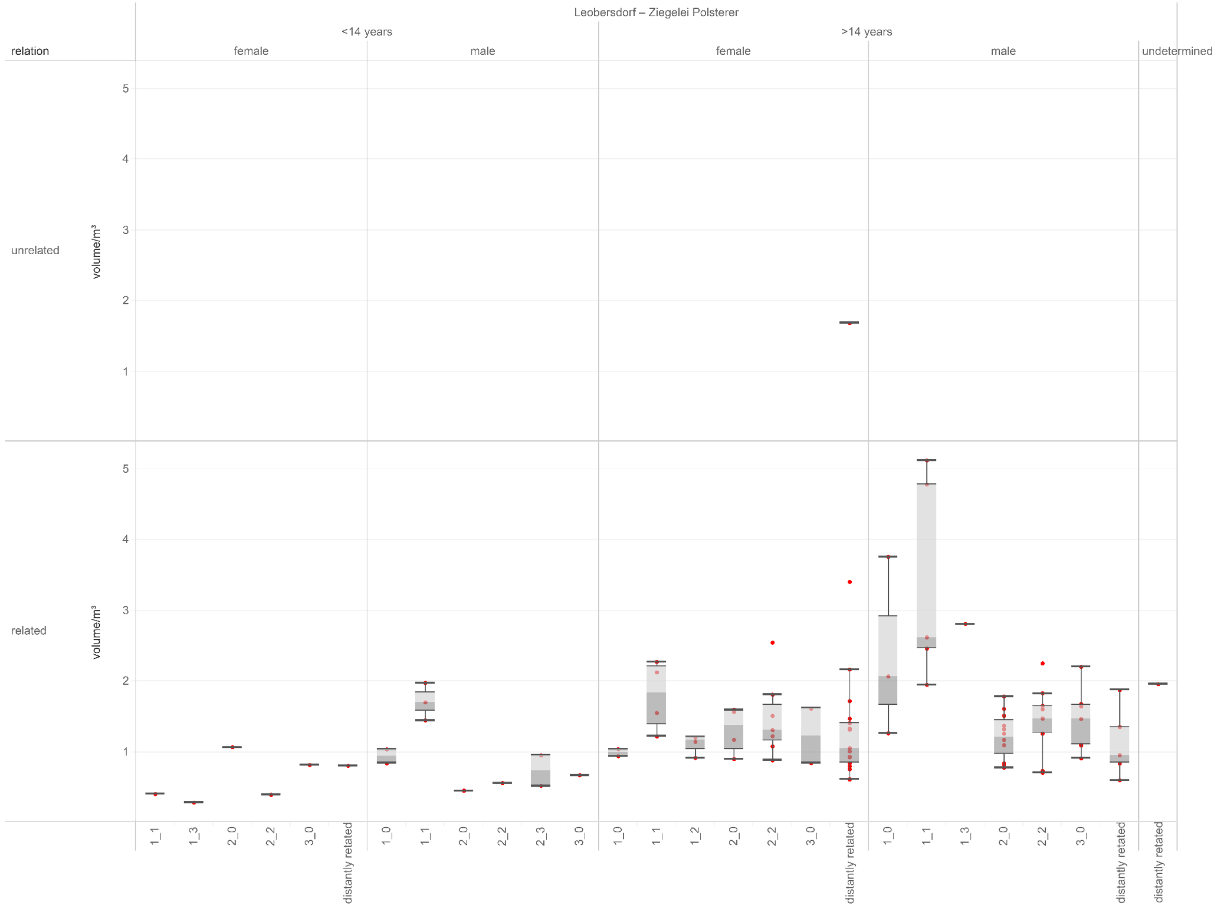

Supplementary Fig. 31. Box plot of grave volumes (m3) excluding multiple, double and reused grave pits in Leobersdorf grouped by sex age groups and pedigrees.

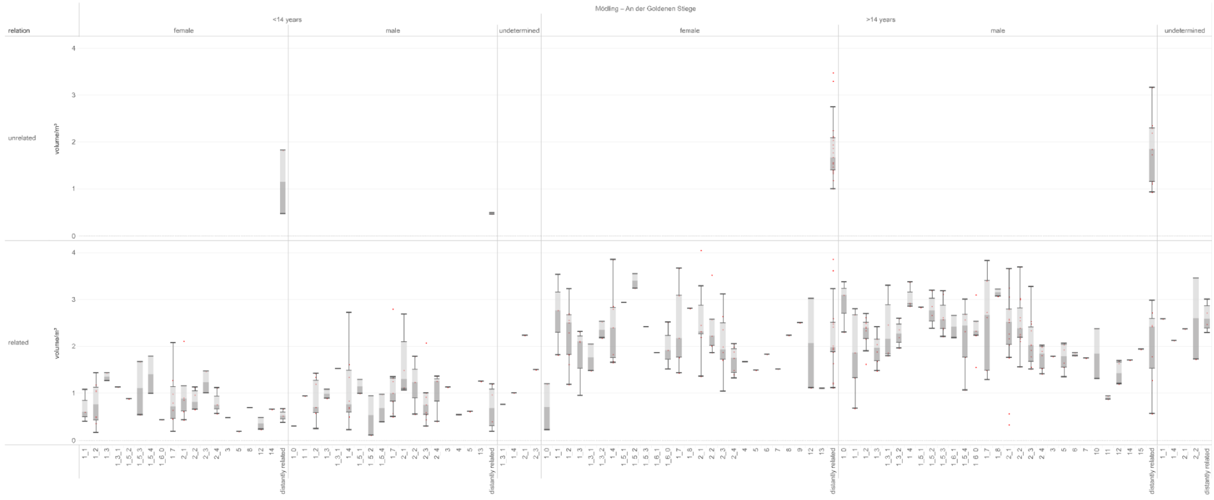

Supplementary Fig. 32. Box plot of grave volumes (m3) including multiple, double and reused grave pits in Mödling grouped by sex age groups and pedigrees.

iii. Single, double and multiple burials and reuse of burial pits

In Mödling there are a larger number of grave overlaps, and the grave pits tend to be closer to each other than in Wien-Csokorgasse and Leobersdorf. Burial rites seem to have been shared to a certain extent, as the majority of individuals were buried in separate burial pits at all three cemeteries. Burials of two or more individuals next to each other, where it is assumed that the inhumations took place at the same time, occur less frequently. In some cases, a burial was added to an already existing burial pit at a later time (Supplementary Fig. 33). In each of these cases, the primary burial was more or less disturbed. Complete animals were buried together with human individuals in Mödling-An der Goldenen Stiege and Wien-Csokorgasse – dogs in both cemeteries, horses and dogs together only in Wien-Csokorgasse.

In Leobersdorf, the number of multiple graves is significantly higher than in Mödling (χ^2 p=0.018), while this is not true for the reused graves (Supplementary Fig. 33).

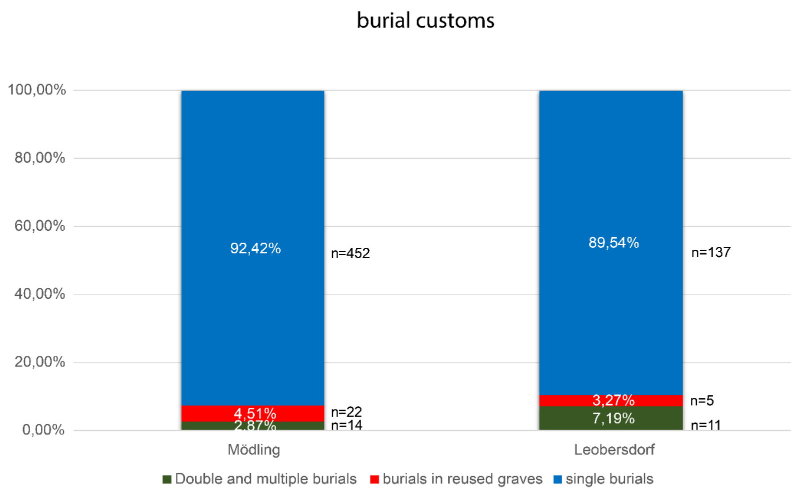

Supplementary Fig. 33. Use of grave pits for burials as a percentage of the total

iv. Individuals in multiple burials and multiple-use graves

The description of contemporary multiple burial pits can be found in the chapter on Archaeology above.

In Leobersdorf there are 11 multiple graves (from which three are disturbed, 67, 104 and 145) out of a total number of 153 graves.

Among them, 8 are double and 3 are triple burials. In one case only one individual could be sampled (144). Almost all of the genetically analyzed multiple burials contain individuals closely related including 2 couples (35A+B, 21A+B with a child 21 C), one parent-child and second degree related child (82) and 4 parent-child burials (21, 67, 93, 119). Only grave 114 is a double burial with not closely related adults in different pedigrees, an old adult male and a young-middle adult female. In contrast to all the other double burials, the bodies are oppositely oriented (the female shows a diverging orientation). Seven among the 11 children from multiple burials died as infans I. There is no sexual preference in parent-child burials, however if siblings are buried together, they are brothers who died as infans I or II (103, 145).

In total, there are four cases of one parent and child buried together in Leobersdorf (Leo-ZP 82, 93, 104, 119); one parent and two children (Leo-ZP 67); three pairs of couples (Leo-ZP 21, 35, 79); three pairs of siblings (Leo-ZP 103, 145, 134); and both parents buried with their child of undetermined sex (Leo-ZP 21).

In addition, Leobersdorf has six reused graves 53, 79, 99, 105, 122, 134 (not sampled 105a, 122b).

Two of the reused graves (79 and 134), located in close proximity to each other in Leobersdorf, contained probably originally double burials. 79b/C and 79b/D are the grandparents of the two boys 134c and d, buried as infans I and II in pedigree 2.

It is noteworthy that five of the six individuals from the group of unrelated individuals in Leobersdorf were found in reused graves: Two potential secondary burials (79a, old adult male and 122b, old adult female); 99b belonged to a disturbed skeleton of an old adult female under grave 99a (from which the bones are missing) and 105c and d, an old adult female and an infans I girl came from the disturbed bones in this grave. In the unrelated group, only grave 37 was a regular burial of a middle adult female.

On the other hand, in Mödling there are 14 multiple graves out of a total number of 488 graves. Among them one is a triple burial (31) and 13 are double burials (159, 167, 256, 257, 265, 315, 317, 382, 406, 490, 505, 507, 529; one was disturbed, grave 490 and in grave 167 only one individual could be sampled, grave 505 included a secondary burial). Nine out of 13 analyzed multiple graves contain individuals closely related: zero couples and six parent-child, one grandparent-child burial and two brothers. 11 of the 12 children from multiple burials died as infans I and with three exceptions (31C, 257 A and B) all of them were boys. There is no preference of one sex in parent-child burials, but if siblings are buried together, they are brothers (one juvenile and one young adult pair). In total, there are six parent-child (Moe-GST 31, 265, 406, 490, 505, 529), one grandparent-child burial (Moe-GST 317) and two burials with brothers (Moe-GST 315, 382).

There are 14 reused graves in Mödling (24+23, 231+239, 233+232, 291+290, 197+180, 258+263, 352+345, 404+403, 417+408, 450+452+459, 227 had only few remains), and three with four individuals (81, 363+365+366+367 and 389). Almost half of them are in the group unrelated or distantly related.

In eight burials, during the anthropological investigation additional skeletal remains were discovered: 36, 44, 55, 119, 168, 201, 364, 518 (not sampled 55 II, 518 II), the majority of them are assigned to the pedigrees. In one disturbed grave with four closely related individuals two parent-child relationships were assigned (respectively 389 a and b, and 389 c and d). Grave 58 contains at least three neonates and one infans I, representing separate burials on the top of a Hallstatt pit (three of them were boys). In grave 81 there were three superimposed burials (81A the lowest and 81C on the top), and the fourth one (81_IV) was discovered during the anthropological investigation, but the assignment of the skeletal remains to individual numbers is not clear. This grave was also found on top of a Hallstatt pit. In addition, some of the bones show brown-black traces of exposure to fire.

The main differences regarding multiple burials between the sites thus are that multiple burials are more frequent in Leobersdorf than in Mödling. In both sites multiple burials contain mainly close relatives, frequently infants, but there are no couples buried in Mödling. In case this is not only a result of bias, the choice, tradition or necessity leading to multiple burials was higher in Leobersdorf than in Mödling. It also strikes that in Leobersdorf the children buried in multiple burials are of either sex, whereas in Mödling the children buried in multiple graves are preferentially boys. Finally, in both sites siblings buried together always

are brothers, in Leobersdorf boys who died as children and in Mödling brothers who died as infants, juveniles or young adults. That only male siblings are buried together in both sites suggests once more the sex-influenced treatment of the dead.

In Leobersdorf it is remarkable that five of the six individuals from the group of unrelated individuals were found in multiple graves, while in Mödling the majority of individuals from reused graves or individuals identified during anthropological investigations could be genetically assigned to pedigrees.

v. Kinship-oriented cemetery development

The development of the cemeteries of Leobersdorf and Mödling is strongly influenced by biological kinship: the initial generation was buried in small groups or at a large place in between them. Up to the third generation the burial pattern is maintained with relatively large distances between burials. Only from the fourth generation onwards does the distance between the graves decrease continuously. At the same time, it was attempted to bury close relatives next to each other. Thus, groups of relatives can be observed in the cemetery. While in Leobersdorf no grave disturbances due to grave overlapping can be detected, in Mödling these can be found in 37 cases. In only 4.2% of the relatives in the pedigree the grave pit was disturbed by overlaps. In the case of individuals that were relatives but not in the main pedigree, the figure was 14%. The highest percentage was among the unrelated individuals in the cemetery with about 22% overlapping. It is obvious that better grave care also depended on the relatives present at the site.

The precondition for this burial practice was the marking of the graves. At least the position of the previous generation must have been known to the community. Since the cemetery was used in all areas at least until the last generation, the area must have been maintained and kept free from strong vegetation. To ensure this, the cemetery areas had to be kept free either by members of society or by herd animals (sheeps, goats, cattles).

vi. Grave goods

As it was common in the whole Carpathian Basin in the period from the 7th to the 9th century, the majority of the deceased were provided with garments, jewelry and offerings. Only just under 10% of the individuals had no grave goods at all in the cemeteries of Leobersdorf and Mödling (Supplementary Fig. 34). Half of these individuals were children under the age of 14 years. No sex differences were observed in either age group (Supplementary Fig. 35). The small number of burials without grave goods allow us to observe that unrelated individuals and individuals which are related but not in the pedigree were treated in the same way as the individuals within a pedigree and were buried with garments, jewelry and offerings.

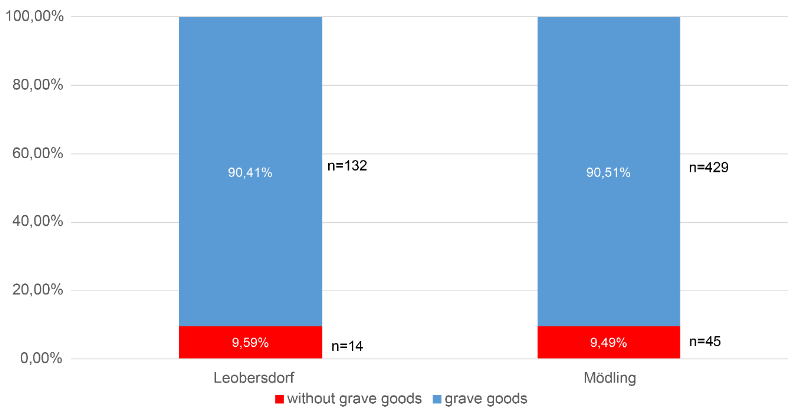

Supplementary Fig. 34. Percentage of graves with and without grave goods in Leobersdorf and Mödling

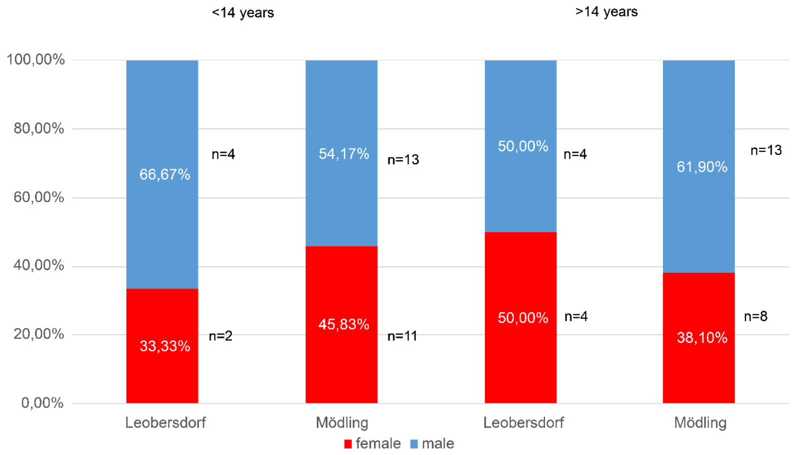

Supplementary Fig. 35. Individuals without grave goods grouped by age and sex as a percentage of total number in Leobersdorf and Mödling.

vii. Differences in the use of grave goods

1. Ceramic vessels

Pottery vessels regularly occur in both male and female graves in the cemeteries of Wien-Csokorgasse and Mödling, but only rarely in Leobersdorf. About 60% of the individuals in Mödling had vessels in their graves, while in Leobersdorf only 15% to about 6% had vessels, depending on sex. However, the low percentage of pottery vessels is also attested in other preceding and contemporary eastern Austrian burial sites such as Zwölfaxing II, Frohsdorf (27.7%) or Podersdorf am See[144–146]. They are also rare in several studied sites in the Danube-Tisza Interfluve, the Upper-Tisza and the Middle-Tisza region. In Hajdúnánás, for example, not a single vessel is found in the cemetery; in Rákóczifalva and Kunpeszér only around 9% of the burials have vessels, while in Kunszállás, they amount to 28% of the graves[5]. Similarly, ceramic vessels are not typical in contemporaneous burial grounds of the Turkic period in the Altai, Tuva or Mongolia. When they do occur, everyday ceramics are used for the burials and no ceramics are produced specifically[147]. However, we do

occasionally find vessels made of wood or leather[147]. In Mödling there are no differences between males and females in the incidence of ceramic vessels, in Leobersdorf females were given vessels significantly more frequently than males (χ^2 p=0.00001, Extended Data Fig. 7). In Leobersdorf, the age of the deceased also plays an important role. Under the age of 14, male and female individuals were given vessels in the grave with about the same frequency. From the age of 14, however, females received significantly (χ^2 p=0.00001) more.

Based on these data, the amount of pottery in the graves is one of the most striking differences between the two sites). We have no results as to what these vessels contained, but most likely these were food offerings for the journey to the netherworld. Archaeobotanical investigations of Avar age vessels from Hungary have shown that bread or porridge was mainly deposited in vessels in child burials; even flowers have been found[148]. Evidence of certain liquids such as milk is not yet available. At first glance, the difference between both sites might be associated with observations that nomad populations generally use less pottery than sedentary ones (other types of containers are more convenient in a mobile lifestyle). However, the population of Leobersdorf, at least after the first two generations, seems to have led the same kind of sedentary life as the one of Mödling. In any case, it is important to emphasize that the custom of placing vessels in the grave depended on the local community as a whole and not on their individual ancestry. Thus, individuals within the cemeteries of Mödling and Leobersdorf with completely different ancestries (Asian or European) may both have vessels in their graves or no vessels at all. Thus, the cultural preferences of the respective community appears to be more important than the individual genetic ancestry.

2. Necklaces

Necklaces consisting mainly of glass beads were also worn by women and men, although they seem to have played a somewhat smaller role than earrings. Around 49% of the females in Leobersdorf and 61% of the females in Mödling wore necklaces. The proportion of female individuals over 14 years of age with necklaces is about 43% in Leobersdorf and 59% in Mödling, slightly more necklaces were found with girls under 14 years of age in Mödling (66%) and an even greater number in Leobersdorf (81%). Here, necklaces seem to have been commonly added to children’s graves (Supplementary Fig. 36). In Mödling, about 17% of all males wore beaded necklaces, with around 25.6% of boys under 14 having one and 13.8% in adulthood. In Leobersdorf, the necklaces hardly play a role among men. Only two adult males were given beaded necklaces in their graves (3.64%) (Supplementary Fig. 37).

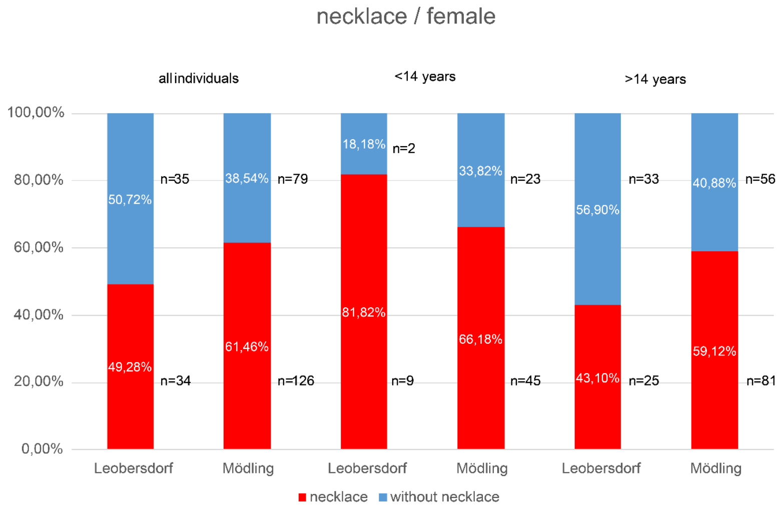

Supplementary Fig. 36. Necklaces in female burials as a percentage of a total of each age group

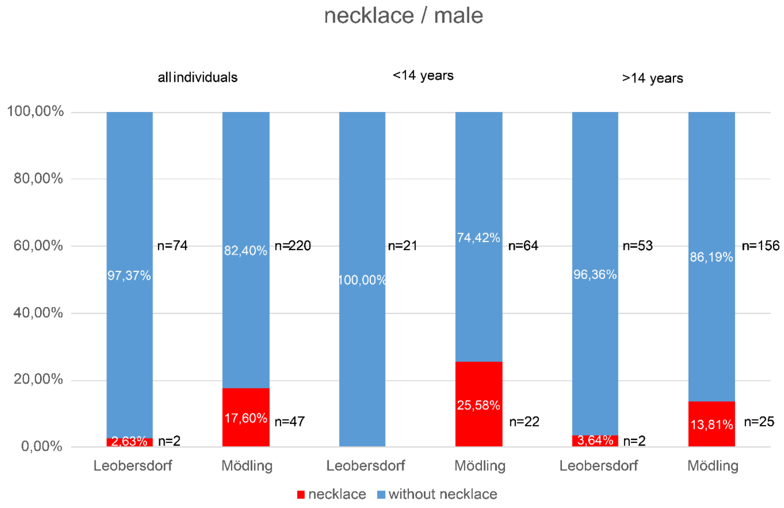

Supplementary Fig. 37. Necklaces in male burials as a percentage of a total of each age group

In general, it can be seen that glass beads are far more common in Mödling than in Leobersdorf (Supplementary Fig. 38).

A low proportion of bead necklaces, like in Leobersdorf, or their absence in men's graves can also be observed for the Avar-period cemeteries of Zwölfaxing II and Podersdorf am See[145], and also occur in many of the Middle-Tisza-Region cemeteries[149]. The cemeteries of Sommerein, Münchendorf and Zwölfaxing I also show quite a high proportion of bead necklaces, but the percentage in male graves is very low[150–152]. In the genetically studied cemeteries of the Danube-Tisza-Interfluve-Region and Trans-Tisza-Region, bead necklaces are also characteristic for women's graves and are only rarely found in men's graves[5]. As far as both the proportion of beads and the distribution in terms of sex and age groups are concerned, the cemeteries of Wien-Csokorgasse and Zillingtal are comparable to Mödling[153].

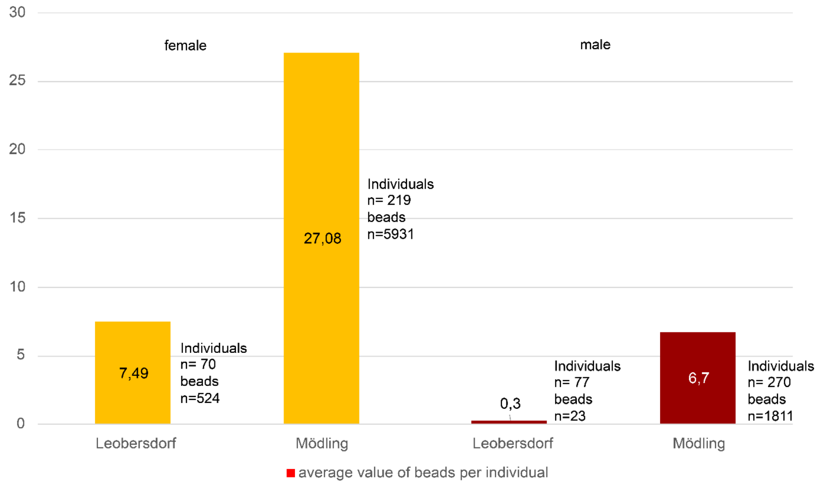

Supplementary Fig. 38. Average number of beads per individual in Leobersdorf and Mödling

viii. Prestige and social status

1. Belt buckles

Prestige objects and status symbols are only accessible to a relatively small group of people. Without written or iconographic sources, however, it is difficult in many cases to identify such items among the existing grave artifacts and to determine their original function. In this context, the belt plays an important role. As we can observe from the grave findings, up to two belts served to hold and close the outer and/or the inner garments of both men and women. Usually only the belt buckles are still preserved from these belts. Gender and age seem to be decisive factors in the distribution of belts in graves. In both cemeteries, more than 50% of the males had belt buckles in the graves, whereas only around 30% of the females had at least one. Grouped by age and gender, a more distinct picture emerges, which reflects the habits at which time of their lives subadults were equipped with certain items of daily use: In Leobersdorf, 85% of the males under the age of 14 had a belt buckle , above the age of 14 only 43.9%, which is significant (Fisher exact χ^2 p=0.002, Supplementary Fig. 39)). In Mödling, the trend is the opposite - 32.9% of the males under the age of 14 had belt buckles, while from the age of 14 the figures rise to 76.8% (χ^2 p=0.000, Supplementary Fig. 39). For females the percentages are more similar in both cemeteries but differ greatly from the males: In Leobersdorf, females younger than 14 years of age received no belt buckles at all, 15.5% of them had one in Mödling (Supplementary Fig. 40). Of the females older than 14 years, 34.5% had belt buckles in their graves in Leobersdorf and more than 50% in Mödling (significant at χ^2 p=0.032, Supplementary Fig. 40).

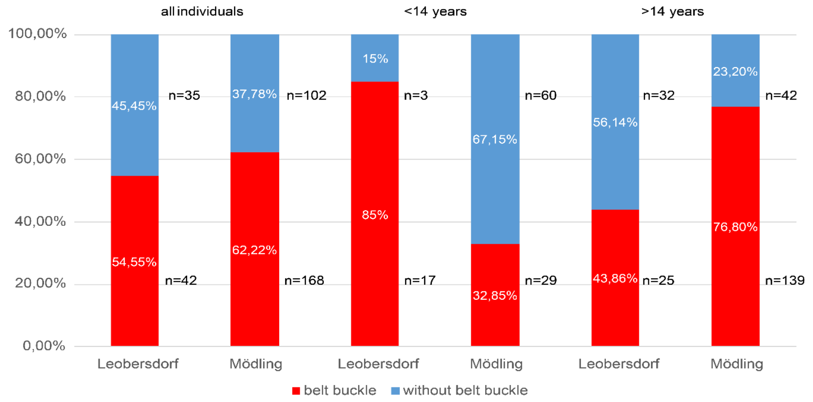

Supplementary Fig. 39. Belt buckles in male burials as a percentage of a total of each age group in Leobersdorf and Mödling.

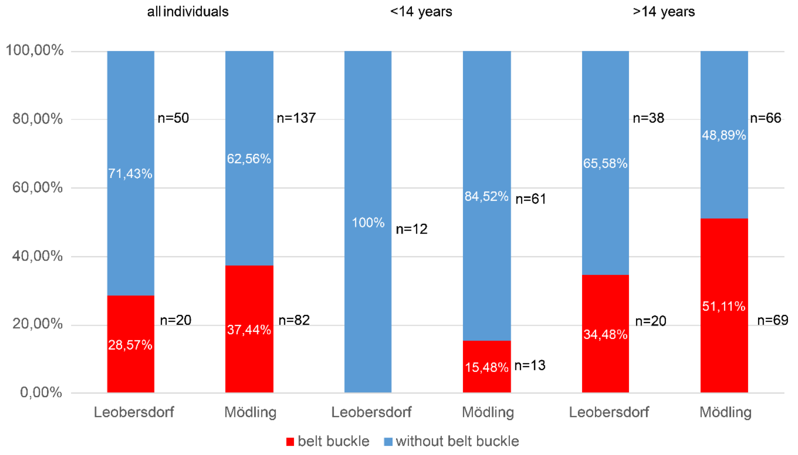

Supplementary Fig. 40. Belt buckles in female burials as a percentage of a total of each age group in Leobersdorf and Mödling.

2. Belt sets

Apart from the simple belts closed only with a belt buckle, so-called multi-part belt sets made from silver or copper sheet metals or cast in copper alloy stand out. Iconographic representations and written sources indicate that these belts were symbols of status and rank as well as symbols of official dignity[6,154]. Since we lack concrete evidence to interpret these belts in the Carpathian Basin of the 7th-9th century AD, a more general interpretation as a status symbol is probably the most appropriate.

In Leobersdorf and Mödling genetic sex determination seems to indicate that these belt sets were reserved for men (Extended Data Fig. 4). 29.9 % of the men in Leobersdorf were buried with such belts or parts of such belts, which is significantly more (χ^2 p=0.013) than in Mödling with a percentage of only 17%. Again, a separation by age groups provides cultural insights. 35.1% of the individuals over 14 years of age were buried with multi-part belts in Leobersdorf, whereas only 23.8% received them in Mödling, but statistically not significant. The proportion of children with these belt sets is low in both places, but higher in

Leobersdorf with 15% compared to 3.4% in Mödling, but not statistically significant (χ^2 p=0.074). Comparing the frequencies of belt sets in the age groups within both sites, significantly more males aged >14 years were buried with one than those below 14 years in Mödling (χ^2 p=0.00001). The lower proportion of simple belt buckles among individuals over 14 years of age in Leobersdorf could be explained by the higher proportion of multi-part belt sets in this age group (Extended Fig. 2).

Expressions of elevated status in the representation of the deceased at their burial, then, were slightly more frequent in Leobersdorf than in Mödling. The difference in the number of sub-adults buried with belt fittings indicates that this form of representation was not necessarily by merit or office, but by descent. This is also confirmed by the uneven distribution of belt sets between different pedigrees or parts of pedigrees. That does not necessarily mean, however, that the Leobersdorf population generally enjoyed higher status than the Mödling one. It may also be that social competition, which led to higher investment in displays of status at the funeral, made itself felt a bit more in the smaller settlement of Leobersdorf. In quality, the top level belt sets at both sites does not indicate any overall status difference between them. The iconography on the belt fittings shows that most of those from the Vienna Basin came from a local workshop circle[155]. In both sites, the surfaces of the fittings on selected belt sets were refined with gold in some cases (Leo-ZP_069; Leo-ZP_071) or tin plating (Moe-GST_325; Moe-GST_326; Moe-GST_358; Moe-GST_440; Moe-GST_521).

With regard to the pedigree lines, the wearers of the belt sets in Leobersdorf differ from each other. Overall, there are more bearers of a belt set in the sub-pedigree 1 than in the other sub-pedigree. Half of the males in sub-pedigree 1_1 (50%) and 66.7% in sub-pedigree 1_3 wore one; 36.4% in sup-pedigree 2_2, while only 20 to 25% in the other sub-pedigrees did. Only in line 1 are there pairs of brothers (Leo-ZP_021A and Leo-ZP_069 or Leo-ZP_036 and Leo-ZP_071) who had belt sets or parts of belt sets in the graves. Brother pairs Leo-ZP_021A and Leo-ZP_069 in particular are remarkable for the fact that the round belt fittings of Leo-ZP_021A were copied from the set of Leo-ZP_069. In addition, three children were buried with belt sets, too.

Comparable differences can also be found in Mödling: The pedigree lines 1_5_2 and 1_6 contain a comparatively large number of belt set wearers. Three pairs of brothers (Moe-GST_093 and Moe-GST_242, Moe-GST_110 and Moe-GST_135 or Moe-GST_139 and Moe-GST_100) with belt sets can be found in Mödling, as well as a total of six belt set bearers whose sons also wore such garments (Moe-GST_518 and Moe-GST_529A, Moe-GST_035 and Moe-GST_276 or Moe-GST_279 and Moe-GST_283). Particularly noteworthy is the complex relatedness constellation of Moe-GST_242 with brother Moe-GST_93, his son Moe-GST_440 as well as his sons Moe-GST_140 and Moe-GST_382A, all of whom were buried with belt sets and belonged to the two pedigree lines 1_5_2 and 1_6.

3. Close combat weapons (sword, sabre, seax, axe, lance)

Close combat weapons (such as sword, sabre, seax, axe and lance) are only rarely included in the graves of males above the age of 14 years. In Leobersdorf and Mödling less than 10% had such a weapon. With around 16% the percentage in Wien-Csokorgasse is significantly higher compared to the other sites (χ^2 p=0.0007 CSK:Vienna basin), while no significant (χ^2 Fisher exact p=1.0) difference was observed between Leobersdorf and Mödling. Often the bearers of these weapons are interpreted as elite warriors. Researchers have suggested that swords in Eastern Central Asian societies can be interpreted as insignia of power[156]. According to differing archaeological interpretations, the weapons in early medieval graves in Europe simply

represent a personal armament, or they show the wealth of the deceased whose family can afford to bury a precious metal object, or they underline his legal status as a free warrior[157]. In the former territories of the Roman Empire, weapons were no longer furnished by the state as in the Roman army, but had to be provided by the soldiers. “Some weaponry elements such as swords were routinely given as gifts to establish fidelity bonds between warriors and their leaders. Weapons thereby became unique symbols of specific social links”[158]. Whether this observation made for post-Roman Italy is also applicable for the Avar realm is debatable. Also, we should not take it for granted that the most renowned warriors were endowed with the most weapons in their graves. In Anglo-Saxon England, boys sometimes had swords in the graves rather than adults[159]. It remains plausible that weapons in graves constitute traces of a circulation of symbolic objects[160], which marked off the sphere of a military community in death. Combat weapons are frequent in graves of the early and middle Avar period, in line with the considerable number of military activities reported in the written sources until 626 CE, and the mostly internal struggles attested after that in the course of the 7th century[161,162].

Obviously, that was different in the northwestern periphery of the Avar realm in the 8th century. In the graves of Leoberdorf and Mödling very few close combat weapons were found, which is striking (Supplementary Fig. 41). In Leobersdorf, only two single-edged swords were excavated. Both bearers (Leo-ZP_071 and Leo-ZP_035B) are from the third generation, their parents and grandparents were not buried at the site. Apart from the swords, two axes are attested in Leobersdorf, one next to Leo-ZP_071 (father) and the other one to his son Leo-ZP_081. This is the only instance of a generational sequence in the deposition of weapons in Leobersdorf. In Mödling, one sword was given to an adult in the penultimate generation (grave Moe-GST_350), the other, of which only a parry bar was found, to a boy under 6 years of age (Moe-GST_374), who, moreover, does not belong to the main pedigree (related in small pedigree 5). In contrast to swords, axes occur more frequently from the third generation onwards. However, no generational sequence practice of depositing axes can be observed in Mödling. Only one pair of brothers can be mentioned, to both of whom axes were given (MOE-GST_382A & 382B). Further close combat weapons found in Mödling are one lance and three seaxes. Usually, only one weapon per individual was deposited in both cemeteries, with the exception of MOE-GST_350 where a sword was found together with a lance. In Wien-Csokorgasse a combination of axe with sword or sabre can be observed (graves 257 and 523). Nevertheless, almost twice as many close combat weapons were deposited in the graves of the Wien-Csokorgasse cemetery than in the other sites. It is remarkable that in the Vienna-Csokorgasse cemetery there are relatively many axes in the graves, even as compared to the Carpathian Basin as a whole[163]. The axes could have been used as tools, too. But in combination with other weapons and the lack of other tools in graves make it likely that they were used as battle axes.

Due to the small number of weapons in all three sites, it is difficult to make general statements about close-combat weapons. The slight difference between Wien-Csokorgasse and the other two sites may be explained by its location at the main route along the Danube, where a higher military presence is more likely than in the fairly sheltered areas of the Leobersdorf and Mödling sites (Supplementary Fig. 41). But basically, none of the three cemeteries correspond to the often more warlike burials of the earlier Avar period, and to the image of a warrior society that is often associated with steppe peoples such as the Avars. Rather, the evidence confirms the picture of a rather peaceful period in 8th-century eastern central Europe that we also get from the written sources[6]. It also clearly contradicts any assumption that late-Avar men of Asian extraction would be in any way more militarized than those of European ancestry. On the contrary: Among the Europeans in Wien-Csokorgasse, relatively more burials emphasized the warrior masculinity of the

deceased than either in Mödling or in Leobersdorf. It is also interesting to compare all three of the Vienna Basin cemeteries to the roughly contemporary cemetery at Kunszállás in the Danube-Tisza interfluve[5]. Weapon burials were more frequent there, in the heartland of the Avar empire, than in the border region south of Vienna. There is no evidence that any of the three sites there would represent a garrison settled here to control and protect the border of the Avar settlement area, which ended at the Vienna Woods, against Franks or Slavs.

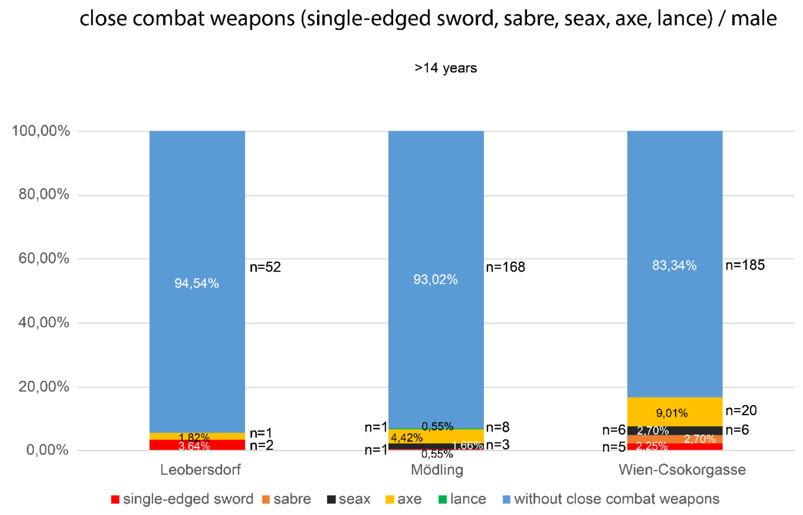

Supplementary Fig. 41. Close combat weapons in the burials of males >14 years from the cemeteries of Mödling, Leobersdorf an Wien-Csokorgasse as a percentage of a total number of all individuals

4. Evidences of injuries

The scarce anthropological evidence of lesions or trauma corresponds to the picture of a largely peaceful community in which military activities and violence played no big role, on the pragmatic and on the symbolic level. In both Leobersdorf and Mödling, the proportion of injured individuals is very low and restricted to healed or healing conditions, suggesting a rather peaceful way of life[164,165]. The same applies for the Csokorgasse[42]. However, there is a striking exception: In Mödling three cases of decapitation (all males) were already described in a previous publication. Two of them were buried in supine position, with their heads in anatomical location, while the third one was found in prone position but also with his head in the anatomically correct place[166]. All three are in the main pedigree 1 in different generations and different parts of the cemetery. During the new analyses carried out for the present study a fourth beheaded male individual was found. Due to a grave disturbance, the original location of his head can no longer be determined. Although violence is usually more prevalent among males, apart from these four cases, no perimortal, that is fatal, injuries were visible on either males or females.

5. Coat clasps

It is more difficult to find appropriate objects that can be interpreted as status symbols for women than for men. Usually, rare high-quality articles are interpreted as prestige objects[167]. That applies to the fine coat clasps, made of copper-alloy sheet metal with glass inlays. A singular coat clasp from MOE_144, representing two warriors drawing the bow, was refined with a gilded surface. Just two percent of the female individuals

in Leobersdorf and Mödling had such coat clasps (Extended Data Fig. 2). While in Mödling the coat clasps were found exclusively among females over 14 years of age, the only coat clasp from Leobersdorf was on a girl under 14 years of age (Extended Data Fig. 2). Three of the Mödling females with coat clasps were young adults who cannot be linked to a partner and children, most likely, recently married. Only one middle-adult female had a partner (not found on site) and two children. Strikingly, most of these graves are located near males who wore belt sets. In Leobersdorf, this is also true for the girl who lay next to a boy with a belt set. A proximity of women and children with coat clasps to men with belt sets could also be observed in the cemetery of Nuštar (HR)[168]. It is, however, significant that status representation at the funeral only rarely involved putting conspicuous jewelry in female graves, and in the rare cases where that happened, it mostly involved women without attested children.

ix. Gender

1. Earrings

Earrings were an integral part of attire in both cemeteries. They were mainly made of a copper alloy, but silver alloy earrings were also found in both cemeteries. In isolated cases we can prove the use of gilding (LEO_009; LEO_028; MOE_034) and tin plating (LEO_096; MOE_251) or gold alloy (MOE_035). In addition, some earrings have glass pendants in the form of beads. From a technological point of view, there are more types of earrings in Mödling than in Leobersdorf, and they were worn by men and women, with some differentiations. Over 60% of females were buried with earrings. The younger females tend to have less than the older ones. About 30% of the females under the age of 14 in Leobersdorf and 42% of them in Mödling had earrings. Over 14 years of age, the percentage of females wearing earrings increases significantly about 66 % in Leobersdorf (χ^2 Fisher exact p=0.0045) and over 80% in Mödling (χ^2 p=0.00001) (Supplementary Fig. 42). Among the male individuals, between 20% and 30% had earrings in both cemeteries. In Mödling, the proportion of males buried with earrings does not change markedly between boys under 14 and older males, whereas in Leobersdorf only 5% of the boys under the age of 14 had an earring – so men seem to have received an earring when they were over 14 years old, whereas those in Mödling presumably received one already at the beginning of their childhood (Supplementary Fig. 43).

Differences can also be seen in Leobersdorf with regard to the distribution of earrings among the males in the pedigrees. Thus 29.4% of the males in the entire sub pedigree 2 had earrings, 16% in sub pedigree 3 and only 4.2% (one individual) in sub pedigree 1. There only the male Leo-ZP_21_A could be assigned at least one earring due to the location in the grave. Due to the multiple burial together with his partner and the common child, an assignment of the earring to his partner would be possible. Thus the males of pedigree 1 by the absence of earrings would differ significantly from those of the other pedigrees where earrings are more common.

Comparable proportions of men with earrings are also found in the Middle-Tisza region, as a recent study shows[149].

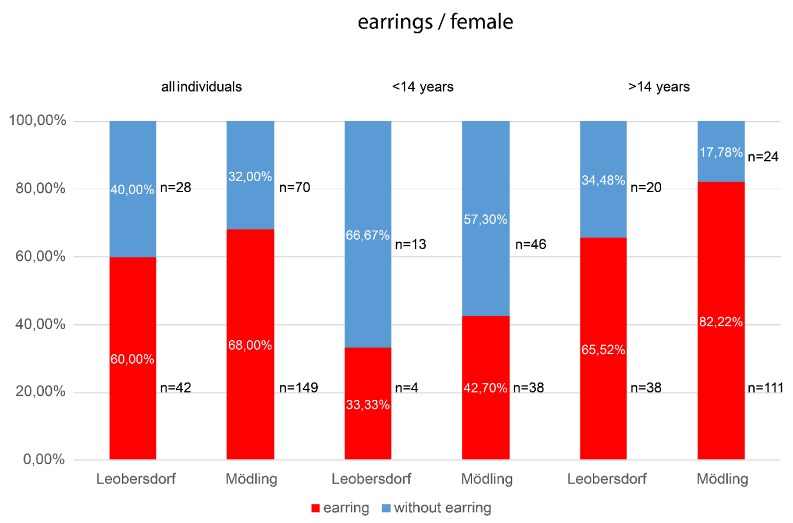

Supplementary Fig. 42. Earrings in female burials as a percentage of a total of each age group

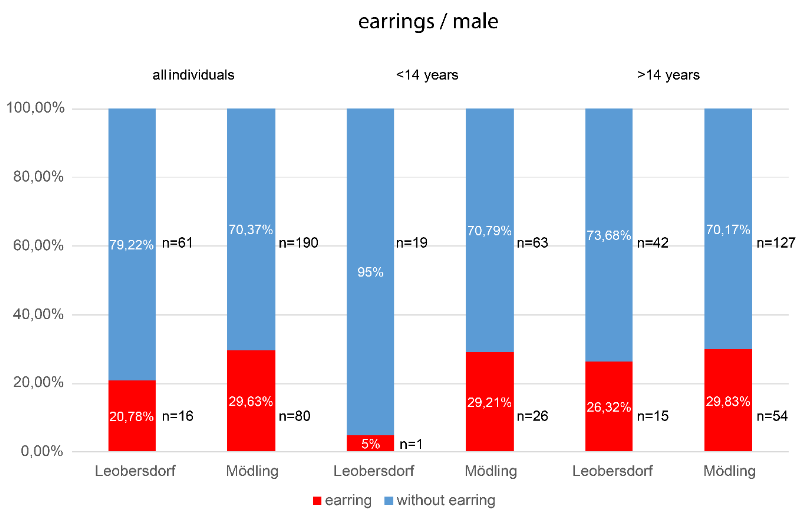

Supplementary Fig. 43. Earrings in male burials as a percentage of a total of each age group

2. Spindle whorls

Spindle whorls were found in female, but also in male graves. More than half of the females in Leobersdorf and half of the females in Mödling had a spindle whorl, the proportion of females over 14 years of age is over 60% in Leobersdorf and almost 70% in Mödling. This is because only 19% of the girls in Mödling were given a spindle whorl, whereas the proportion in Leobersdorf was twice as high at over 40%. The proportion of females in Mödling having spindle whorls between the two age groups is significantly different (χ^2 p=0.00001, less in the infants). While no spindle whorls were given to men in Leobersdorf, there were nine with a whorl in Mödling (Supplementary Fig. 44).

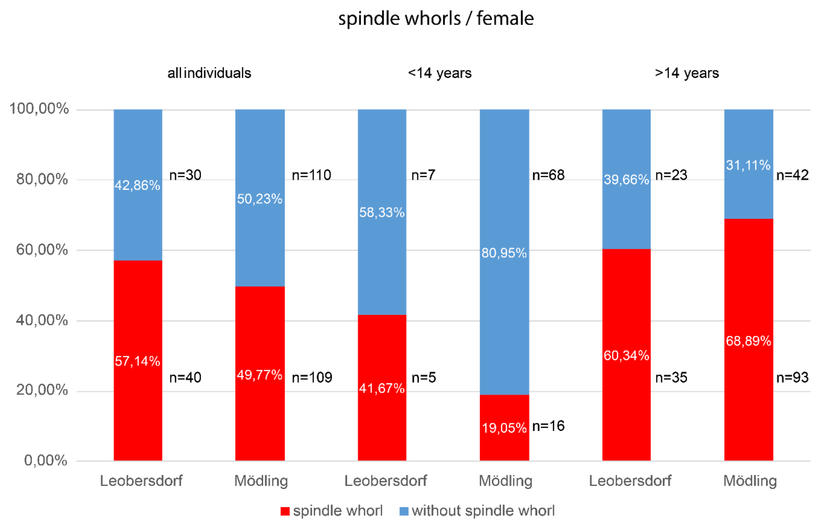

Supplementary Fig. 44. Spindle whorls in female burials as a percentage of a total of each age group

3. Combinations of earrings, necklaces and spindle whorls

In addition to looking at the earrings, necklaces and spindle whorls individually, it is worthwhile to consider the combination of all the three items together. Only 27.8 % of the females in Leobersdorf had such an item combination, as compared to more than twice as many females in Mödling (62.9 %). The mean age of the deceased was taken into account, for which three age groups between 0-18, 19-40 and 41+ were chosen. On the basis of these age groups, the deposition and use of the item combination from childhood to the old adult age becomes more apparent. In combination, the items occur less frequently than individually. While in Leobersdorf the proportion among females having these three items decreases constantly from 0-18 to 41+ from about 39 % to about 6 %, in Mödling it increases from 24 % to about 47 % in the adult age groups. Older Mödling females were equipped in the same way as those in the middle age group (Supplementary Fig. 45). Among men, none has the combination of earring, necklace and spindle whorl in his grave in Leobersdorf, but four in Mödling have. Due to the poor state of preservation of the skeletons, it was not possible to determine the sexes anthropologically. However, genetic analysis determined their biological sex as male. The youngest of the males was 14-15 years old (Moe-GST_017) the other one between 20 and 30 years (Moe-GST_266). None of these males had a partner or children, both are linked to the pedigree 1 through parents or siblings, respectively. These two examples show that even if anthropological or archaeological sex assignments agree, they do not necessarily reflect the actual biological sex. More importantly, the evidence for different gender expressions has been found in early medieval societies[169]. Their location in the cemetery also suggests that they are at least part of the burial community. In comparison to Leobersdorf, however, differences in gender expressions are not visible in all communities, at least in this form.

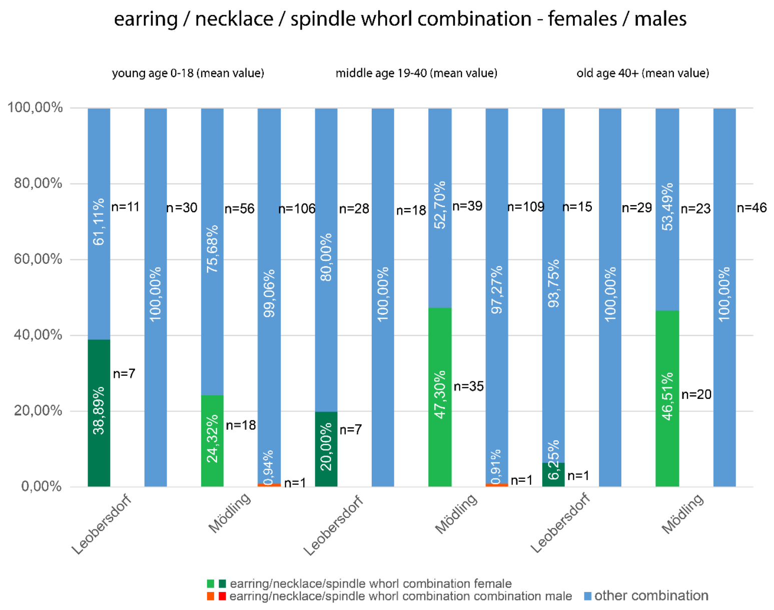

Supplementary Fig. 45. Item combination (earring / necklace / spindle whorl) as a percentage of a total of each age group

4. Females – Item combinations

As the last graph has already shown, the combination of different items in a grave reveals gender and social identities. Based on the frequency of the Leobersdorf and Mödling grave goods, different item combinations were determined. For female burials both cemeteries share the combination of animal offerings, earrings, necklaces and textile working tools in general. In Mödling, an additional significant combination consists of belt buckles, knives and ceramic vessels. Only relatively few individuals were equipped with a combination of so many different objects. In Leobersdorf, it can be seen that in the two age groups of 0-18 and 19-40, respectively, the combination remains the same with about 22 % of the females. It drops sharply by almost three quarters to about 6 % from the age of 41 plus. In Mödling, a completely different trend can be observed, here the combination increases almost threefold with each age group (Supplementary Fig. 46). Among females in Mödling it can also be observed that in the age group 18-40 only three out of ten had one child and one had three children. In the following age group 41+, only one of the 13 females did not have a child. Seven of the females had more than three children and three even had two partners. In Mödling, females with several children and a higher age were valued at least as much as younger females in this society. This was not the case in Leobersdorf, where older females were hardly given the same gifts as younger females.

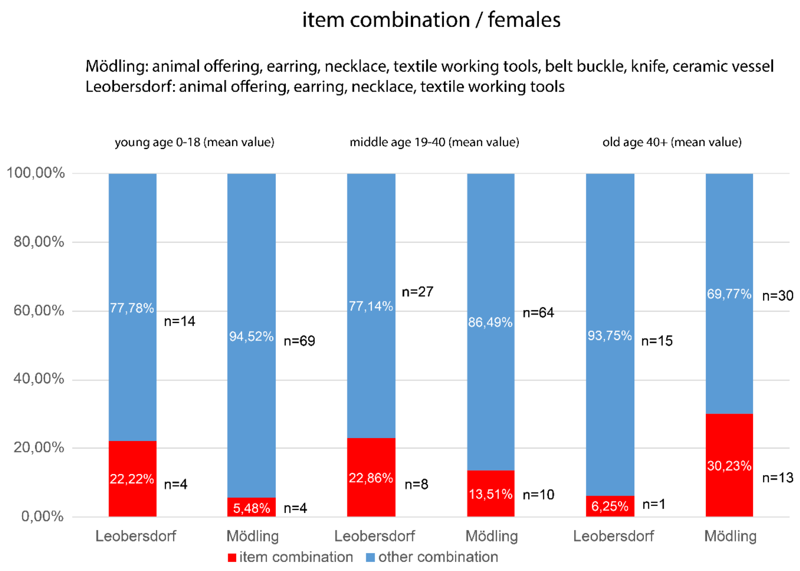

Supplementary Fig. 46. Item combination female individuals as a percentage of a total of each age group

5. Males – Item combinations

Similar to the grave goods for women, two different combinations were also chosen for the men, based on the frequency of occurrence of the items. In both cemeteries, it is the combination of animal offerings, belt buckles, belt sets and long-range weapons in general, in Mödling ceramic vessels are added as well. Different trends can be observed among men, both in contrast to women and in comparison between the two sites. In Leobersdorf, the combination of grave goods increases strongly from the age group 0-18 to the age group 19-40 and then decreases strongly again in men over 40. In comparison, at the age of 0-18, the females already had the same level as in the following age group. In Mödling, the combination also decreases strongly from the first to the second age group, but then remains at approximately the same level in the third age group. Here, a difference to the females can be noted, who show a significant increase in the item combination in the age group 41+.

With regard to the combinations of different items, differences can be seen in Leobersdorf and Mödling. This applies above all to the age groups in which grave goods and jewelry are present. Some grave goods are already given in childhood, while in the other cemetery they are only relevant from adulthood onwards. It can also be seen that especially in Mödling there is no drop in the number of grave goods in the 40+ age group, whereas this is the case in Leobersdorf. Among the women in Mödling, there is even an increase in the number of individuals who are buried with a combination of grave goods in this age group (Supplementary Fig. 47).

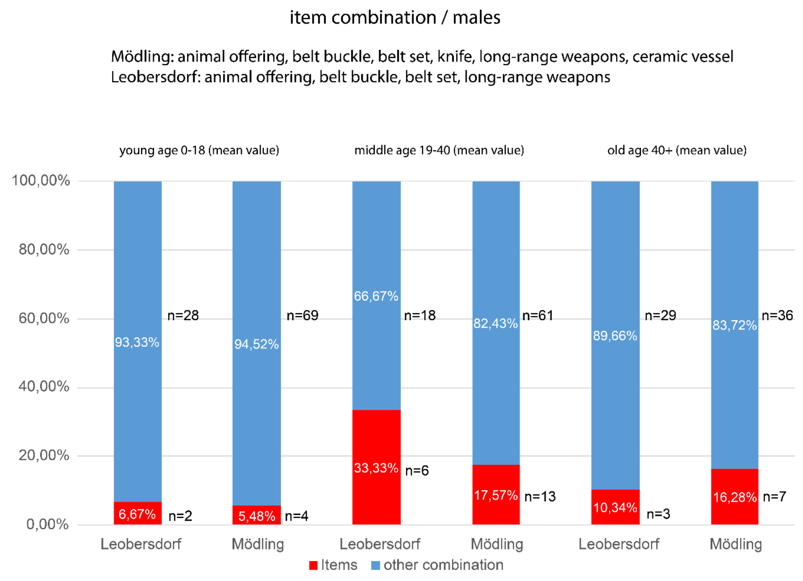

Supplementary Fig. 47. Item combination male individuals as a percentage of a total of each age group

6. Age related associations grouped by sex

d. Long distance connections

i. Female mobility

There are several types of evidence pointing to high adult female mobility in Leobersdorf and Mödling.

1. Pedigrees and linearity – mothers and non-mothers

As the pedigrees demonstrate, at least two thirds of the lineages are patrilinear compared to matri- or bilinearity in reproductive unions in Leobersdorf and Mödling (see Supplementary Table 8).

The majority of the female partners engaging in reproduction come from outside these communities, at least most of them do not have any parents buried on site, and thus represent female mobility (present in pedigree: 17 of 19 in Leo and 45 of 58 in Mödling). The ratio of mothers present compared to those inferred in the main pedigrees is slightly higher in Mödling (LEO 1:2.1, MDG 1:1.6). Among the non-mothers, in Leobersdorf and Mödling the majority died as juvenile and young adult (LEO 12 of 15, MGS 15 of 22). These juvenile and young adult single females could represent females who died before reproducing and leaving the site.

2. Distantly related – largely young adult females

The number of adult females distantly related is considerably higher in Leobersdorf than in Mödling compared to the number of adult males. Apart from that, in Leobersdorf 14 of the 18 females distantly related died during reproductive age (between 18 and 45 years), and left no offspring in the cemetery. This contrasts with the six males distantly related, who mainly died in older age. In Mödling, also 16 of 19 females distantly related died during reproductive age. The 12 distantly related adult males died in all ages.

3. Unrelated – largely females

As for the distantly related individuals, in Leobersdorf and in Mödling the group of the unrelated also consists nearly exclusively of adult females. In Leobersdorf, this group is very small, and five of six died in adult age (three old adults). In contrast in Mödling, 22 of 25 of the unrelated died in reproductive age. Most of the males unrelated to anybody else are evenly distributed over the age groups.

What both sites have in common, then, is that females present in the pedigrees are under-represented. This suggests that a large proportion of females (those unrelated and those only distantly related) came from outside these communities and potentially engaged in relationships. Either they left no offspring in the cemeteries because they did not reproduce at all, died before reproduction, or because their reproductive partners and/or offspring emigrated and/or were buried elsewhere. One indicator for this hypothesis that they died before having offspring is the burial of Mödling-An der Goldenen Stiege grave Moe-GS_144 (MGS153). This young female with extraordinary grave goods was buried close to a group of high status males but had no offspring buried in the cemetery, but potentially a social affiliation to this group. In the same group there are two other examples of unrelated, respectively distantly related young females buried close to these males (Moe-GS_118, Moe-GS_167).

4. Mobile sisters on site

In Leobersdorf, the present sisters without parents consist of the sisters, Leo-ZP_61 (red pedigree, young adult, single reproductive union, one child), and Leo-ZP_097 (old adult, single reproductive union, one child). Other sister pairs are Leo-ZP_003 (red pedigree, old adult, single reproductive union, one child) and Leo-ZP_079b/C (old adult, single reproductive union, two children) and Leo-ZP_086 (blue pedigree, middle adult, single reproductive union, two children) and Leo-ZP_149 (young adult, no children). Interestingly, all of them occur in generation 4 and there they make up half of the present females (6 of 12) in the pedigree. There is no female with an inferred sister buried in the site.

In Mödling, there is only one pair of of sisters present without parents buried in the site (Moe_GS_019: adult, single reproductive union, one child) with sister Moe-GS_390: young adult, no children), and three females who have inferred sisters (Moe-GS_284: old adult, multiple reproductive union, 2 children, Moe-GS_273: old adult, inferred sister in relationship, and Moe-GS_463: young adult, no children).

5. Widows in the Vienna basin

Widows are here considered as those females present in the cemetery who were in reproductive unions with present males, and who seemed to have survived their partners for more than 20 years. In Leobersdorf, no potential widows could be detected, as there are only very few couples present, and those are of similar age.

In Mödling in contrast, at least four potential widows were detected, three of them in the third, one in the fifth generation. Two females were part of a levirate, Moe-GS_143 (old adult) and Moe-GS_120 (old adult), and had children with one male (Moe-GS_093, young-middle adult), whereby Moe-GS_120 also reproduced with the brother (Moe-GS_242, middle adult) of this male. However, this higher number of widows in Mödling is probably associated with the greater number of individuals buried in this site compared to Leobersdorf.

8. IBD Network analysis

a. General pattern

We performed a network analysis on the IBD on the matrix of pairwise IBD connections between all the currently available Avar-period individuals, including the newly sequenced individuals from LEO, MGS and CSK and previously published Avar period sites located in the DTI (KUP, KFJ, DTI_elite) and TT (RK, HNJ) regions in the Carpathian Basin[4,5]. The "DTI_elite" group includes most elite Avar period solitary burials ever found in the nearby area in the DTI region: Kunbábony, Petőfiszállás, Budapest-Csepel-Kavicsbánya and Kecskemét-Sallai út published in Gnecchi-Ruscone et al. 2022[4] which are pooled together for IBD analyses.

For this analysis we considered only shared IBD segments longer than 12cM to focus on the more recent shared ancestry patterns and if a pair of individuals had segments < 16cM we only included them if they had more than one segment in common to exclude residual 1% - 2% false positive rate of the 8-12 cM range[77]. We used Cytoscape v3.9.1[170] to plot the networks of pairwise IBD relations and network statistics were calculated with the R igraph package [171] and custom scripts from publication[5]. The entire network consists of 731 individuals (nodes) and 9234 IBD connections (edges) between them, to visualize the strength of the connectivity between individuals we used the length of the longest IBD segment (max_IBD) shared between a pair of individuals (Supplementary Fig. 48).

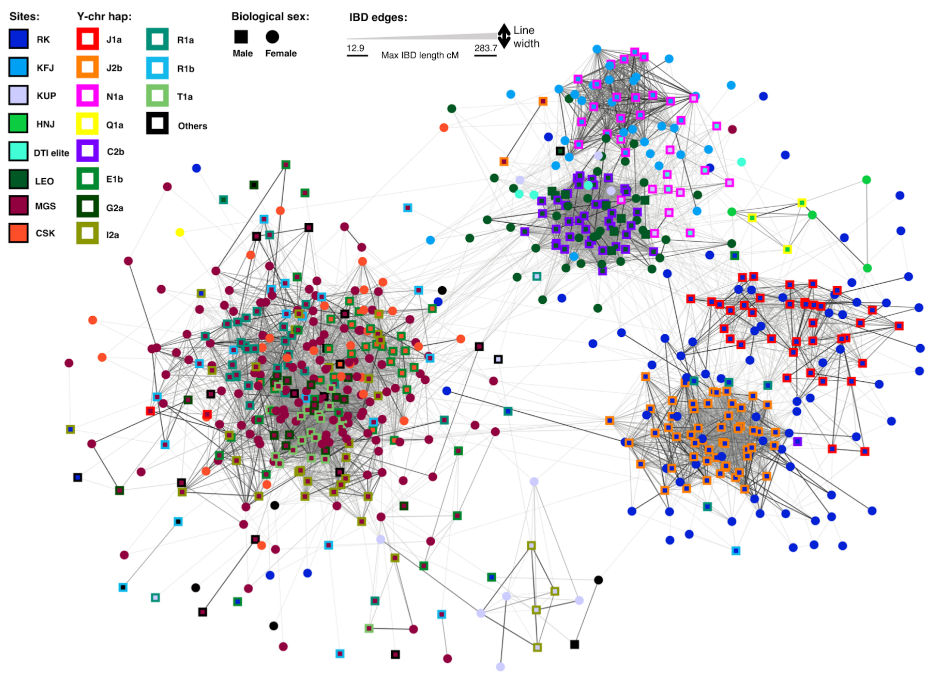

Supplementary Fig. 48. Network analysis of the haplotype-IBD sharing between the Avar period sites with Y haplogroup included shown in bolder colors. Each node is an ancient individual, squares are male and circles female individuals. Node's internal color corresponds to the site, while the border of squares corresponds to the males' Y-haplogroups. The strength of the IBD connection is expressed by the longest IBD tract found that pair of individuals. The length distribution is shown as color and width gradient of the

connecting lines, thinner and lighter gray to thicker and darker gray line corresponds to low to high max_IBD length.

The most evident feature in the IBD network are the main clusters of close genetic relatives found at each of the largest sites considered including the new LEO, MGS and CSK sites. Mostly these clusters tightly correspond to a single patriline of related individuals which in turn usually corresponds to the site unit but there are notable exceptions. Despite being from the same site, the two patrilines of RK site form distinct clusters, and males with N1a haplogroups (KFJ, KUP, DTI_elite) cluster together despite being from different sites[5]. Instead in the MGS site different Y haplogroups (6 main ones with > 10 male individuals) are represented and they do not form separate noticeable clusters in the IBD network. On the contrary the MGS, together with CSK, form an extended macro cluster, reflecting the less strict patrilineal descent system observed in the MGS pedigree(s) with respect to LEO and the other DTI and TT sites. Another main feature of the network is that LEO shares more IBD with the DTI sites of KFJ / KUP / DTI_elite clusters than with the geographically neighboring MGS or CSK sites despite the presence of a few relatively close relatives between MGS and LEO (see Section 4). CSK site in contrast to all other sites does not form its own cluster but it is firmly set in the MGS cluster. That could be due to the low number of sampled skeletons and also the size of this whole community (745 graves) of which only 80 were sampled. Nonetheless, the firm connections between CSK and MGS are apparent from almost all individuals except for Y haplogroup G,H,K.

b. Intra- and interregional patterns of connectivity

To explore the between-site connections in more detail we calculated the average amount of IBD sharing between groups, normalized by the total number of possible individual pairs to account for sample size difference between the groups (Supplementary Fig. 49). We included in the previously described group "DTI_elite", and also another only partially sampled DTI elite cemetery Albertirsa (ALBA)0[4]. We separated individuals from KUP into KUP_EA (EA, Early Avar period) and KUP_LA (LA, Late Avar period) based on the definition of Early and Late Avar period described in Gnecchi-Ruscone et al. 2024[5].

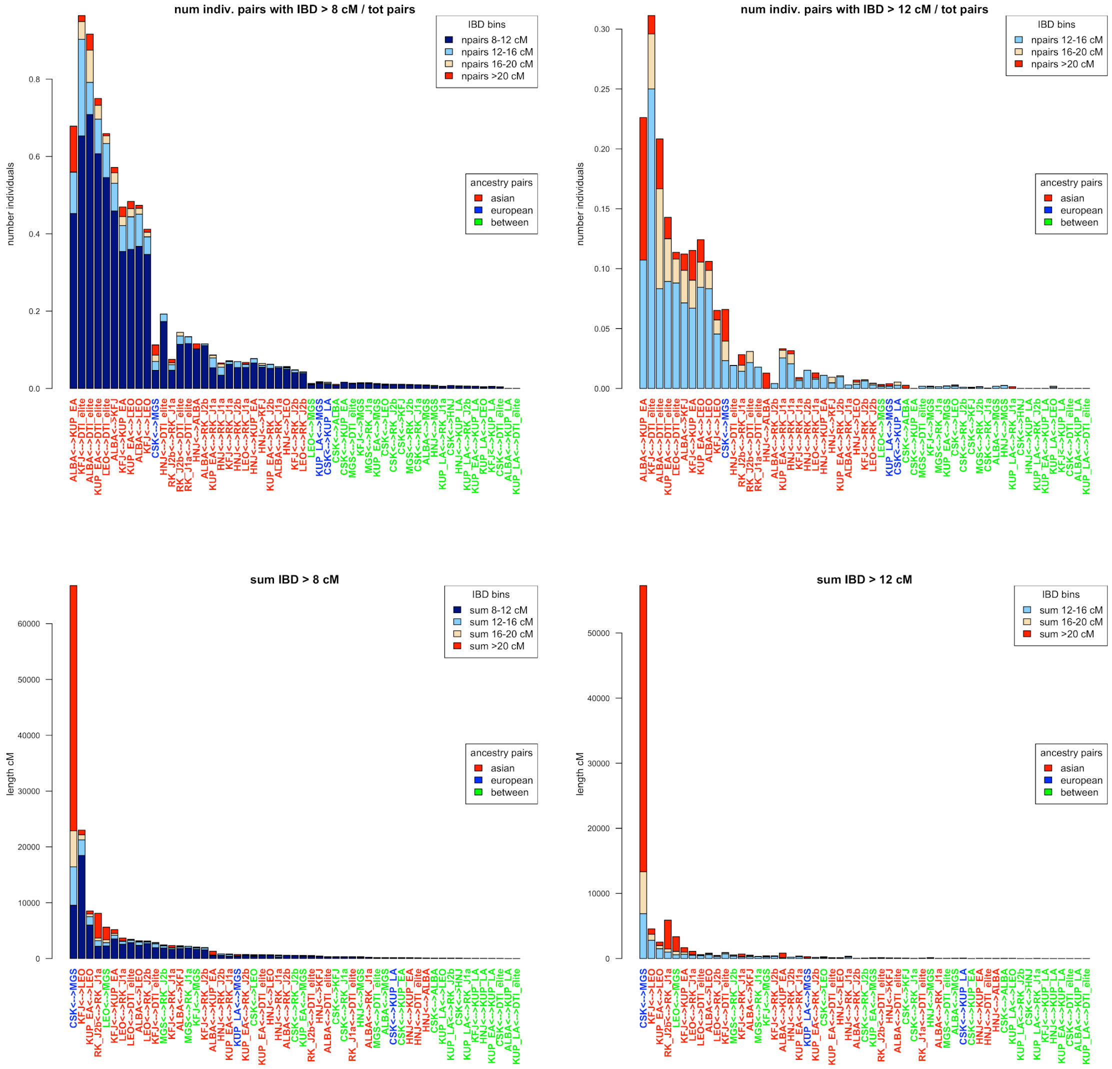

Supplementary Fig. 49. Summary of IBD sharing between the different Avar period sites considered. Top, the number of individual pairs that share IBDs normalized by the total number of possible pairs between the two sites. On the left IBDs are grouped by all the 4 IBD lengths classes and on the right are only plotted the longest IBDs (>12cM) corresponding to the ones visualized in the network graph (Supplementary Fig. 20). **Bottom,** total sum of IBDs shared between two sites, not normalized by sample sizes. The three colors on the X axis correspond to pairs of sites with predominantly Asian ancestry individuals, or European ancestry individuals and between them.

Based on IBD-sharing patterns, we summarised the following observations. First, of all the sites with predominantly East Asian or admixed ancestry individuals (KFJ, KUP_EA, RK, LEO, HNJ, DTI_elite, ALBA) share more IBDs between them (marked in red color as shown in Supplementary Fig. 49) than they share with the sites with mostly European ancestry individuals (MGS, CSK and KUP_LA) (marked in green color as shown in Supplementary Fig. 49). These latter groups of sites share less IBDs among them than the Asian sites but they still share more than they share with the "Asian" sites. Even within the same site, the late Avar period site of KUP (KUP_LA) composed of females of mostly European ancestry individuals shares more IBD with MGS than with the early KUP site or any of the other closer DTI or TT sites. Second, the cluster of sites formed by LEO and the other DTI sites share considerably more IBDs between them than any other sites, especially IBDs in the shorter categories of 8-12 cM or 12-16 cM. LEO and all these DTI sites are also the ones

with the highest amounts of female East Asian ancestry. Lastly, the sites with "intermediate" admixture proportions (HNJ and RK, divided into the two patrilines RK_J1a and RK_J2b) from the TT share less IBDs than DTI and LEO but still present a high amount of IBD sharing between them and with the other DTI and LEO sites. When analyzing the total length of IBD shared between sites, not normalized by sample sizes (Supplementary Fig. 49) it is possible to appreciate the extent of close genetic relatives between specific sites. As confirmed by the network plot and KIN analyses, MGS and CSK have many close relatives between them, and therefore result as extreme outliers in this plot compared to all the rest. The two main patrilines of RK (RK_J1a and RK_J2b) as well as LEO and MGS also share a considerable amount of longer IBDs between each other, reflecting the close relatives found between them (Supplementary Fig. 49)[5].

In conclusion, even if there are cases of direct admixture events within pedigrees and closely related individuals between sites with different ancestry composition, when normalized by the sample sizes, the patterns of IBD sharing are highly correlated to the genomic ancestry composition. Or in other terms, there are clusters of IBD sharing that correlates with clusters of sites with similar genomic composition.

c. Male and female connectivity

To explore the patterns of connectivity between males and females, as the pedigrees are exclusively or strongly patrilineal, we calculated unweighted and weighted network statistics. The IBD network is undirected (as IBD sharing is bi-directional), so we considered undirected statistics: as an unweighted measure we estimated the degree centrality (*k*) defined as the number of connections each node has and for the weighted we calculated the strength (*w*) defined as the sum of the weights (max_IBD) of the links each node has. When sex is considered as a node attribute, the degree and the strength distributions are significantly different between males and females (Supplementary Fig. 50).

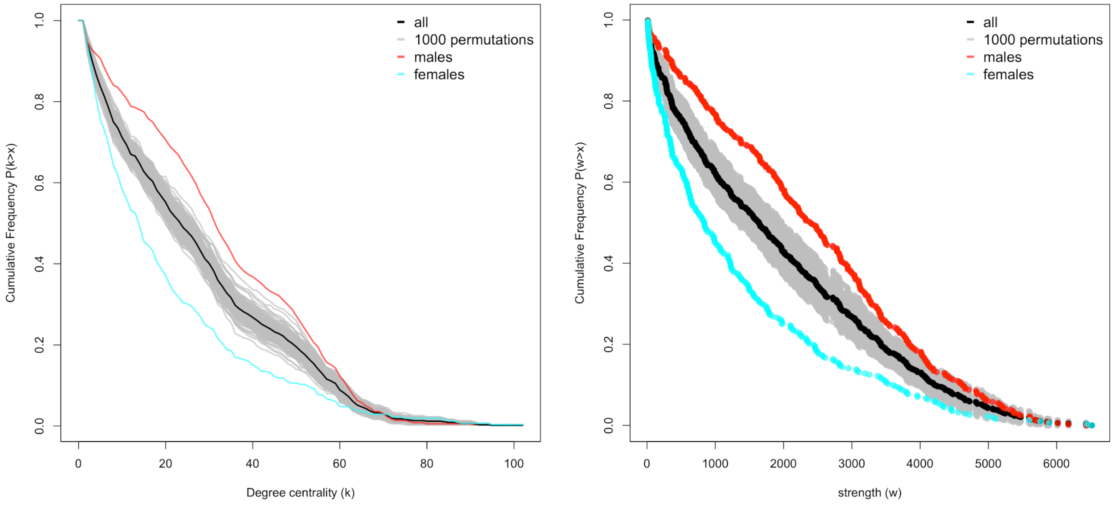

Supplementary Fig. 50. The cumulative density function of the degree centrality (k) and the strength (w) distributions of the network of IBD links > 12 cM in Supplementary Fig. 49.

Analyzing separately the sites with predominantly East Asian or admixed ancestry individuals (KFJ, KUP_EA, RK, LEO, HNJ) and the ones with mostly European ancestry individuals (MGS, CSK and KUP_LA) revealed that this pattern is driven by the former group of sites as the distinction between the male and female

distribution is even more pronounced for the Asian sites, while for the European sites the distributions are not significantly different, falling within the range of random variability (Supplementary Fig. 51).

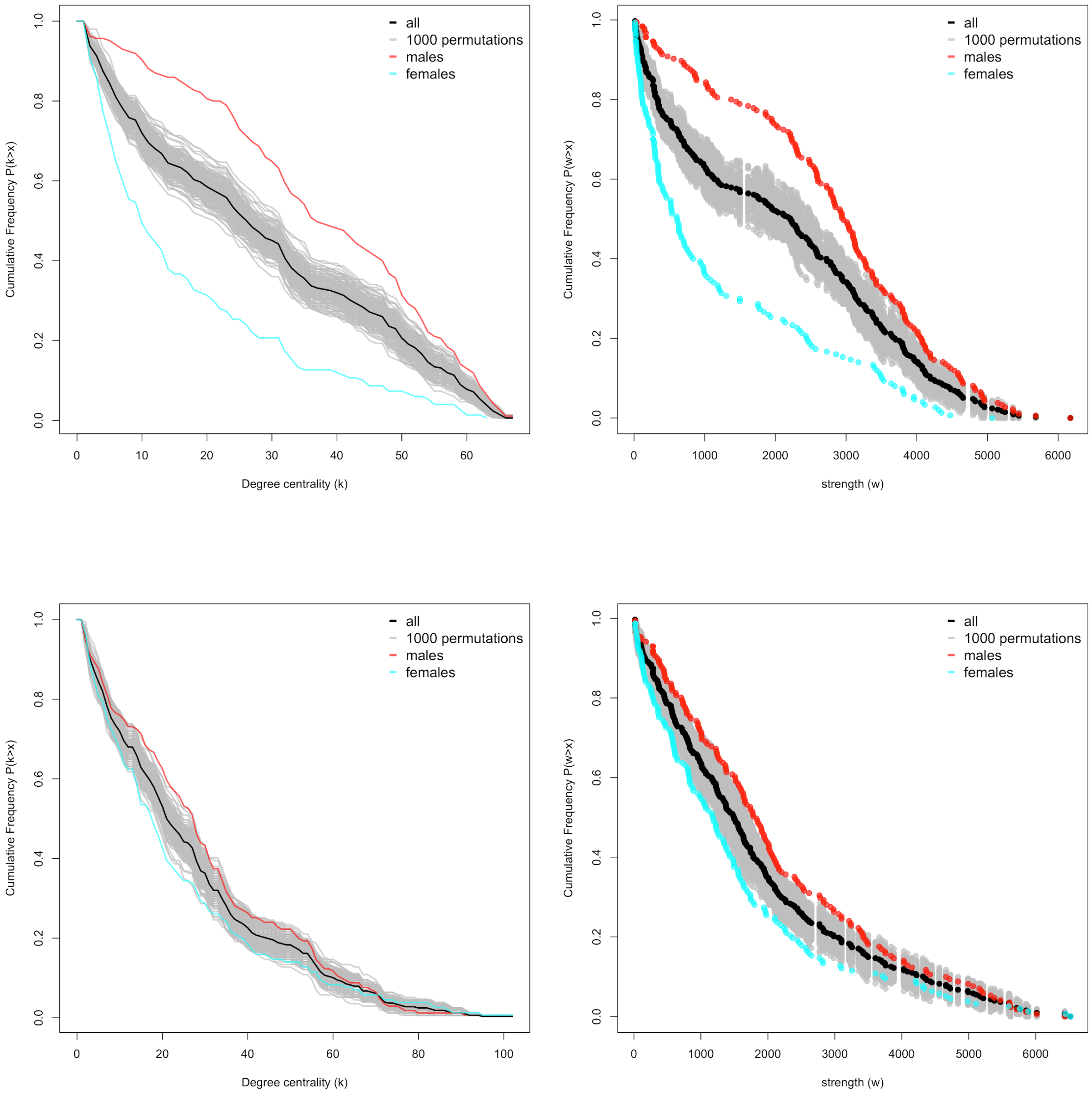

Supplementary Fig. 51. The cumulative density function of the degree centrality (k) and the strength (w) distributions of: top, predominantly East Asian or admixed ancestry individuals (KFJ, KUP_EA, RK, LEO, HNJ); bottom, predominantly European ancestry individuals (MGS, CSK and KUP_LA).

The degree centrality of a node can be split into within-module (k_W) and between-module links (k_B). We considered the archaeological site as a module and calculated the k_B/k ratio, representing the ratio of between-sites connections over the total connections, which can range between 0 and 1, with 0 indicating that the individual has IBDs only with other individuals from the same site and 1 indicating that a related individual has only IBDs with individuals from another site other than its own. If we estimate this distribution separately for males and females we obtain statistically significantly different distributions (Supplementary

Fig. 52 left; t = -5.7848, df = 564.31, mean males = 0.12, mean females = 0.24; p-value = $1.2*10^{-8}$ based on Welch's t-test).

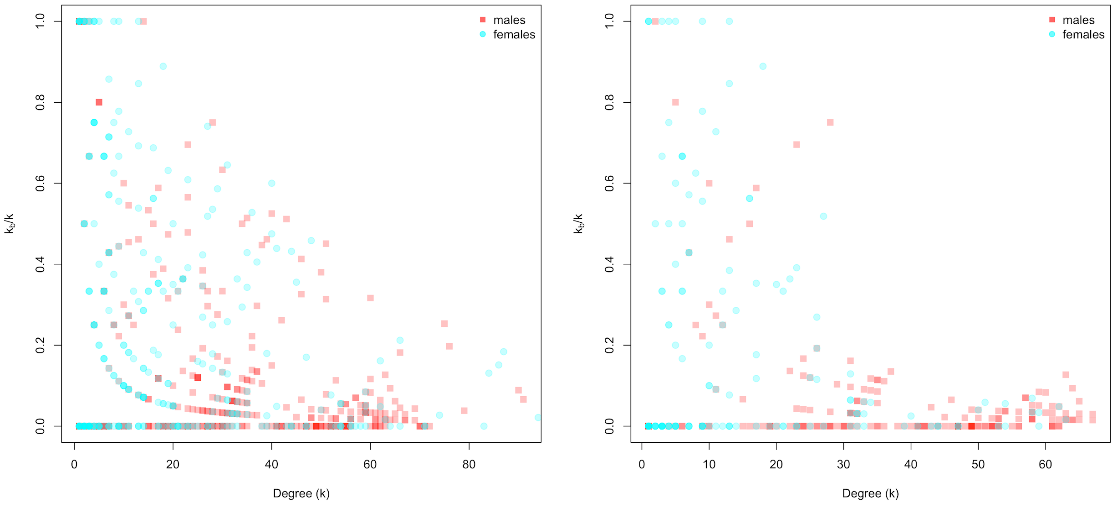

Supplementary Fig. 52 - The total degree centrality (*k*) is plotted vs the ratio of *k* calculated between-site edges and total *k* (k_B/k). Considering the whole network of IBD links > 12 cM (left) and only between the Asian sites (right).

The significance holds when considering only the sites with predominantly Asian ancestry (Supplementary Fig. 52 right; t = -5.0023, df = 209.57 mean males = 0.06, mean females = 0.2; p-value = $1.9*10^{-6}$ from Welch's t-test) and even if only the European sites are considered (t = -3.9878, df = 271.8, mean males = 0.12, mean females = 0.23; p-value = $8.5*10^{-5}$ Welch's t-test).

In conclusion, despite the overall significanly higher connectivity of males with respect to females (Supplementary Fig. 50, 51), when considering only the connectivity between sites, the females are the ones showing the higher connectivity (Supplementary Fig. 52).

d. Regression on IBD Networks

To identify variables corresponding with a significant increase in genetic relatedness, we applied Exponential Random Graph Models (ERGMs) and Generalised Exponential Random Graph Models (GERGMs) in the context of an IBD network. However, unlike for standard linear regression methods, we must consider the correlation inherent in connected graphs when modeling the effect of variables.

In general, a network is defined by a set of nodes denoted X_i = 1, …, n, and a set of edges where Y_{ij} is the edge between nodes X_i and Y_j.

For all ERGM analyses[172,173], we used the *ergm* package within the *Statnet* suite of packages (https://github.com/statnet/ergm). For GERGM analyses, we used the *GERM* package[174].

i. ERGMs

ERGMs allow researchers to ask whether nodes in a network are more likely to be connected based on if they have an individual attribute (nodefactor), if they share the same attribute (nodematch) or if they have some combination of a variable (nodemix).

In the case of the individual-based network, where each node is an ancient individual, we defined an edge to be

$$Y_{ij} = \{0,\ if\ individuals\ i\ and\ j\ are\ unrelated;\ x,\ if\ individuals\ i\ and\ j\ are\ related\}$$

Specifically, we define two individuals to be related if they share at least two IBD blocks of length longer than 12cM, and at least one IBD block of length longer than 16cM.

We began by fitting all additive models, using the *nodefactor*, *nodematch* and *nodefactor* variable representations. For all models, we calculated the Akaike Information Criterion (AIC), and retained the model with minimum AIC. We found that the model which fit best included nodematch on site, indicating that individuals who were buried at the same site are clearly more likely to be related. Importantly, by including this variable we are able to remove the correlation of site and the remaining variables of interest.

We found that nodemix on sex was significant, and that pairs that included two genetically female individuals were the least likely to be related, that a pair that included a genetically male and a genetically female individual are 1.35-fold more likely to be related ($p<10^{-4}$), and that pairs that included two genetically male individuals are even more likely to be genetically related, by a 2.54-fold increase ($p<10^{-4}$).

Next, we found nodemix on status symbols was significant. We saw that if one individual in a pair was buried carrying a status symbol, then this increased the probability of relatedness by 1.27-fold ($p<10^{-4}$). If both carried a status symbol, then this was a 2.55-fold increase ($p<10^{-4}$). Note that the *ergm* package only reports significance to the resolution of 10^{-4}.

ii. GERGMs

GERGMs, like EGRMs, model the effect of variables on connections between nodes in a network. However, where ERGMs model the probability of two nodes being connected, GERGMs model the non-binary edge weight between two nodes. Due to this increased level of complexity, GERGMs can so far only be applied to relatively small networks, and hence we could not apply these to the individual networks.

Like for ERGMs, we fit the full GERGM model and use AIC to identify the best fitting model, and we use measures of hysteresis to assess model degeneracy. We also measure the improvement in mean squared error (MSE) for edge weights when assessing if the GERM improves the fit compared to a simple ERGM. In cases where we identified a significant predictor for the GERGM model, there appeared to be no degeneracy and MSE was improved by >97%.

In the case of sites, we define the edge weight to be the mean number of IBD blocks of length 8-12, 12-16, 16-20 and >20 cM, and perform four different network models. These four models allow us to investigate the patterns of relatedness from distant relatedness (8-12 cM) to close relatedness (>20 cM).

We included the types of ancestry at the sites (Eastern or European) and the geographic location (latitude and longitude). We found that no model required the latitude or longitude, indicating that the distance between sites was not a significant predictor of increased genetic relatedness between sites (Extended Data Fig. 8, Supplementary Fig. 53).

In the case of distant relatedness (8-12 cM and 12-16 cM), the type of ancestry was not a significant predictor of relatedness. However, for closer relatedness measures (16-10 cM and >20 cM), we observed that nodemix for ancestry was a significant predictor (Extended Data Fig. 5, Supplementary Fig. 53). Specifically, if two sites both had Eastern ancestry, then there was a significant increase (between 2-6%) in the expected average number of IBD blocks ($p=1.16 \times 10^{-7}$ and $p=2.24 \times 10^{-8}$) respectively.

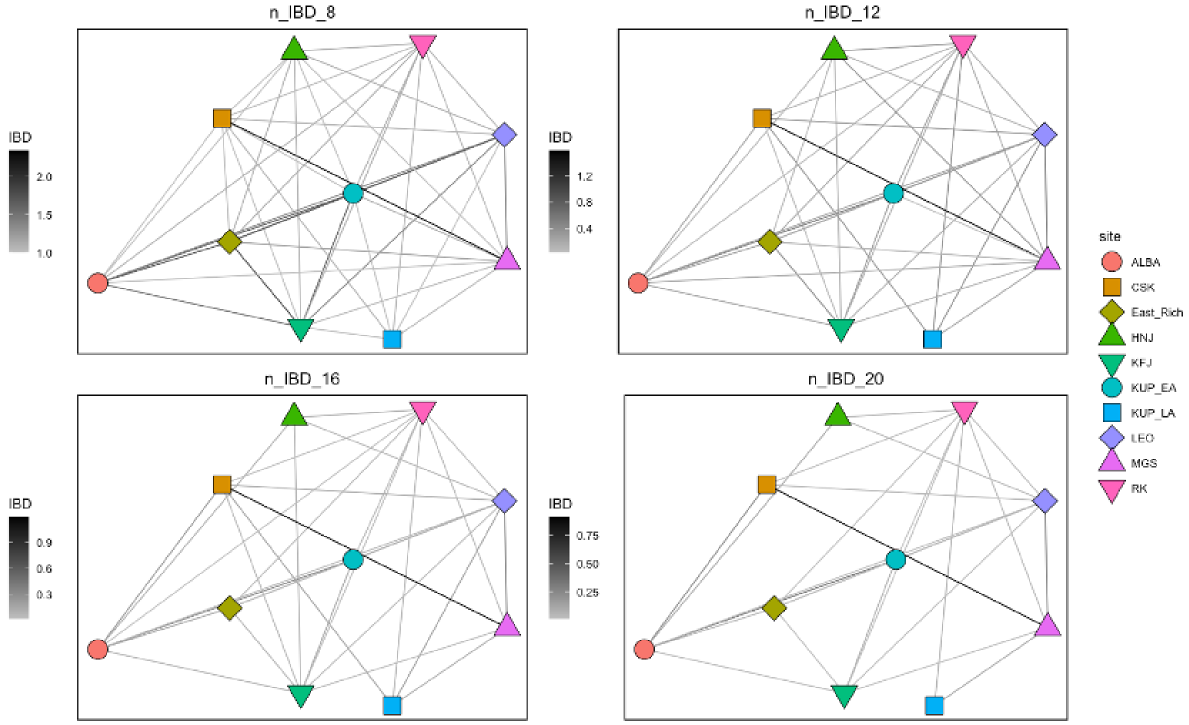

Supplementary Fig. 53. Site-based IBD networks with edges coloured by mean IBD block values. Node shape and size indicate the sites. In order to explore different levels of relatedness, panels are separated by the mean number of IBD blocks of size >8cm (top-left), >12cM (top-right), >16cM (bottom-left) and >20cM (bottom-right).

9. References

1. Mathieson, I. & Scally, A. What is ancestry? *PLoS Genet.* **16**, e1008624 (2020).

2. Amorim, C. E. G. *et al.* Understanding 6th-century barbarian social organization and migration through paleogenomics. *Nat. Commun.* **9**, 3547 (2018).

3. Pohl, W. Introduction ? Strategies of Identification: A Methodological Profile. in *Strategies of Identification* vol. 13 1–64 (Brepols Publishers, 2013).

4. Gnecchi-Ruscone, G. A. *et al.* Ancient genomes reveal origin and rapid trans-Eurasian migration of 7th century Avar elites. *Cell* **185**, 1402–1413.e21 (2022).
5. Gnecchi-Ruscone, G. A. *et al.* Network of large pedigrees reveals social practices of Avar communities. *Nature* **629**, 376–383 (2024).
6. Pohl, W. *The Avars: A Steppe Empire in Central Europe, 567–822*. (Cornell University Press, 2018).
7. Vyas, D. N. *et al.* Fine-scale sampling uncovers the complexity of migrations in 5th-6th century Pannonia. *Curr. Biol.* **33**, 3951–3961.e11 (2023).
8. Barford, P. M. *The Early Slavs: Culture and Society in Early Medieval Eastern Europe*. (Cornell University Press, 2001).
9. Dzino, D. Becoming Slav, Becoming Croat: Identity Transformations in Post-Roman and Early Medieval Dalmatia. in *Becoming Slav, Becoming Croat* (Brill, 2010).
10. Curta, F. *The Making of the Slavs: History and Archaeology of the Lower Danube Region, c.500–700*. (Cambridge University Press, 2001).
11. Kaizer, J., Wild, E. M., Stadler, P., Teschler-Nicola, M. & Steier, P. Update on the Absolute Chronology of the Migration period in Central Europe (375–568 AD): new data from Maria Ponsee, Lower Austria. *Radiocarbon* **61**, 1653–1662 (2019).
12. Wolfram, H. How Many Peoples Are (in) a People? in *Visions of Community in the Post-Roman World* (eds. Pohl, W., Gantner, C. & R. Payne, F.) 101–108 (Routledge, 2012).
13. Brather, S. Die Projektion des Nationalstaats in die Frühgeschichte. Ethnische Interpretationen in der Archäologie. in *Inventing the Past in North Central Europe* (eds. Hardt, M., Lübke, C. & Schorkowitz, D.) 18–42 (The National Perception of Early Medieval History and Archaeology, Frankfurt a. Main, 2003).
14. Pohl, W. What can archaeogenetics contribute to historical research on social identity and relatedness? A medievalist's view. in *Kinship, Sex, and Biological Relatedness. The contribution of archaeogenetics to the understanding of social and biological relations, 15* (eds. Meller, H., Krause, J., Haak, W. & Risch, R.) 51–60 (Mitteldeutscher Archäologentag, Halle/Saale: Landesmuseum für Vorgeschichte, 2023).
15. Pohl, W. Pippin and the Avars. in *Spes Italiae* 99–109 (Brepols Publishers, 2023).

16. Hummer, H. Kinship and inheritance in early medieval Europe. in *Visions of Medieval History in North America and Europe* 151–167 (Brepols Publishers, Turnhout, Belgium, 2022).
17. Sahlins, M. *What Kinship Is-And Is Not*. (University of Chicago Press, 2013).
18. Castro, E. V. de. Chapter 10 THE GIFT AND THE GIVEN: THREE NANO-ESSAYS ON KINSHIP AND MAGIC. in *Kinship and Beyond* 237–268 (Berghahn Books, 2022).
19. Bamford, S., Bamford, S. C. & Leach, J. *Kinship and Beyond: The Genealogical Model Reconsidered*. (Berghahn Books, 2012).
20. Sneath, D. *The Headless State*. (Columbia University Press, 2007).
21. Saliari, K., Tobias, B. & Draganits, E. Animal Husbandry During Late Antiquity: Archaeozoological Analysis and Regional Comparison of the 4th to 6th Century AD Small Rural Settlement in Podersdorf am See (Burgenland, Austria). *Environmental Archaeology. The Journal of Human Palaeoecology* 1–22 (2022).
22. Geary, P. J. *The Myth of Nations: The Medieval Origins of Europe*. (Princeton University Press, 2002).
23. Daim, F. *Das Awarische Gräberfeld von Leobersdorf, NÖ*. (Verlag d. Österr. Akad. d. Wiss, 1987).
24. Eibner, C. & Matzner, K. Mödling, BH Mödling. *Fundber. Österreich* 148–149 (1971).
25. Matzner, K. Mödling, BH Mödling. *Fundber. Österreich* 212–217 (1971).
26. Matzner, K. & Schwammenhöfer, H. Mödling, Gem. Mödling, BH Mödling. *Fundber. Österreich* **12**, 127–129 (1974).
27. Schwammenhöfer, H. & Schwammenhöfer, L. Mödling, Gem. Mödling, BH Mödling. *Fundberichte aus Österreich* **10**, 102–127 (1972).
28. Schwammenhöfer, H. & Schwammenhöfer, L. Mödling, Gem. Mödling, BH Mödling. *Fundberichte aus Österreich* **11**, 121–130 (1973).
29. Matzner, K. *Der Awarenfriedhof in Mödling: 'An der goldenen Stiege'*. (Katalog der Sonderausstellung im Museum der Stadt Mödling, 1977).
30. Daim, F. *Die Awaren in Niederösterreich*. (Verlag Niederösterr. Pressehaus, 1976).
31. Stadler, P. Das langobardische Gräberfeld von Mödling, Niederösterreich. *Archaeologia Austriaca Wien* **63**, 31–47 (1979).

32. Moßler, G. Zwei neue Fundorte frühgeschichtlicher Gräber in Niederösterreich. *Österreichische Zeitschrift für Kunst und Denkmalpflege* **12**, 108–110 (1958).
33. Streinz, L. Wien 11–Csokorgasse. *Fundberichte aus Österreich* **17**, 393 (1978).
34. Bühler, B. & Kirchengast, S. Horse-riding as a habitual activity among the early medieval Avar population of the cemetery of Csokorgasse (Vienna): Sex and chronological differences. *Int. J. Osteoarchaeol.* **32**, 821–831 (2022).
35. Streinz, L. & Daim, F. Zur Belegungschronologie der Nekropole von Wien 11–Csokorgasse. in *Quasi liber et pictura. Die Tierknochenfunde aus dem Gräberfeld an der Wiener Csokorgasse. Eine anthrozoologische Studie zu den awarischen Bestattungssitten.* (ed. Baron, H.) vol. 143 615–626 (Monographien des Römisch-Germanischen Zentralmuseums, Mainz, 2018).
36. Streinz, L. Wien 11–Csokorgasse. *Fundberichte aus Österreich* **16**, 475–531. (1977).
37. Huber, E. H. Awaren in Simmering. *Archäologie Österreichs* **9**, 41–43 (1998).
38. Huber, E. H. Neu entdeckte Awarengräber in Wien, Simmering. *Fundort Wien* **1**, 117–143 (1998).
39. Huber, E. H. Wien 11-Csokorgasse. *Fundberichte aus Österreich* **36**, 899–902 (1998).
40. Benedix, J. & Greussing, I. Die nachantiken Grabfunde Wiens und ihr Verhältnis zu den Überresten Vindobonas unter berücksichtigung neuer Radiokarbondaten. in *Von Vindobona zu Wienna. Archäologisch-historische Untersuchungen zu den Anfängen Wiens* (ed. Felgenhauer-Schmiedt, S.) 175–219. (Wien, 2019).
41. Grefen-Peters, S. *Das Awarische Gräberfeld von Leobersdorf, NÖ*. (Verlag der Österreichischen Akademie der Wissenschaften, Wien, 1987).
42. Großschmidt, K. Paläopathologische Untersuchungen an den menschlichen Skeletten des awarenzeitlichen Gräberfeldes Csokorgasse in Wien-Simmering: Schmelzhypoplasien, Cribrosierungen und Harris' sche Linien als Streßindikatoren. (University of Vienna, 1990).
43. Herold, M. Sex differences in mortality in Lower Austria and Vienna in the Early Medieval Period: An investigation and evaluation of possible contributing factors. (University of Vienna, 2008).
44. Orfanou, E., Himmel, M., Aron, F. & Haak, W. Minimally-invasive sampling of pars petrosa (os

temporale) for ancient DNA extraction v2. *protocols.io* dx.doi.org/10.17504/protocols.io.bqd8ms9w (2020) doi:10.17504/protocols.io.bqd8ms9w.

45. Sirak, K. A. *et al.* A minimally-invasive method for sampling human petrous bones from the cranial base for ancient DNA analysis. *Biotechniques* **62**, 283–289 (2017).
46. Neumann, G. U., Valtuena, A. A., Yates, J. A. F., Stahl, R. & Bramdt, G. Tooth Sampling from the inner pulp chamber for ancient DNA Extraction v2. *protocols.io* dx.doi.org/10.17504/protocols.io.bqebmtan (2020) doi:10.17504/protocols.io.bqebmtan.
47. Dabney, J. *et al.* Complete mitochondrial genome sequence of a Middle Pleistocene cave bear reconstructed from ultrashort DNA fragments. *Proc. Natl. Acad. Sci. U. S. A.* **110**, 15758–15763 (2013).
48. Rohland, N., Glocke, I., Aximu-Petri, A. & Meyer, M. Extraction of highly degraded DNA from ancient bones, teeth and sediments for high-throughput sequencing. *Nat. Protoc.* **13**, 2447–2461 (2018).
49. Gansauge, M.-T. *et al.* Single-stranded DNA library preparation from highly degraded DNA using T4 DNA ligase. *Nucleic Acids Res.* **45**, e79 (2017).
50. Gansauge, M.-T., Aximu-Petri, A., Nagel, S. & Meyer, M. Manual and automated preparation of single-stranded DNA libraries for the sequencing of DNA from ancient biological remains and other sources of highly degraded DNA. *Nat. Protoc.* **15**, 2279–2300 (2020).
51. DeAngelis, M. M., Wang, D. G. & Hawkins, T. L. Solid-phase reversible immobilization for the isolation of PCR products. *Nucleic Acids Res.* **23**, 4742–4743 (1995).
52. Fu, Q. *et al.* DNA analysis of an early modern human from Tianyuan Cave, China. *Proc. Natl. Acad. Sci. U. S. A.* **110**, 2223–2227 (2013).
53. Haak, W. *et al.* Massive migration from the steppe was a source for Indo-European languages in Europe. *Nature* **522**, 207–211 (2015).
54. Fu, Q. *et al.* An early modern human from Romania with a recent Neanderthal ancestor. *Nature* **524**, 216–219 (2015).
55. Brown, T. A., Nelson, D. E., Vogel, J. S. & Southon, J. R. Improved Collagen Extraction by Modified Longin Method. *Radiocarbon* **30**, 171–177 (1988).

56. DeNiro, M. J. Postmortem preservation and alteration of in vivo bone collagen isotope ratios in relation to palaeodietary reconstruction. *Nature* **317**, 806–809 (1985).
57. Ramsey, C. B. Bayesian analysis of radiocarbon dates. *Radiocarbon* (2009).
58. Reimer, P. J. *et al.* The IntCal20 Northern hemisphere radiocarbon age calibration curve (0–55 cal kBP). *Radiocarbon* **62**, 725–757 (2020).
59. Marhold, F.-J. Anthropologische Untersuchung der Skelette des awarenzeitlichen Gräberfeldes in Mödling 'Goldene Stiege': Dissertation. (Universität Wien, Wien, 1977).
60. Acsádi, G. & Nemeskéri, J. *History of Human Life Span and Mortality*. (Akadémiai Kiadó, Budapest, 1970).
61. Bass, W. M. *Human Osteology : A Laboratory and Field Manual of the Human Skeleton*. (Columbia, Mo: Missouri Archaeological Society., Columbia, Missouri, 1971).
62. Lovejoy, C. O., Meindl, R. S., Pryzbeck, T. R. & Mensforth, R. P. Chronological metamorphosis of the auricular surface of the ilium: a new method for the determination of adult skeletal age at death. *Am. J. Phys. Anthropol.* **68**, 15–28 (1985).
63. Ferembach, D., Schwidetzky, I. & Stloukal, M. Empfehlungen für die Alters-und Geschlechtsdiagnose am Skelett. (Recommandations pour le diagnostic de l'âge et du sexe sur les squelettes). *Homo* **30**, 1–32 (1979).
64. Brůžek, J. A method for visual determination of sex, using the human hip bone. *Am. J. Phys. Anthropol.* **117**, 157–168 (2002).
65. Buikstra, J. E. & Ubelaker, D. H. Standards for data collection from human skeletal remains. *Fayetteville: Arkansas Archaeological Survey* **44**, 18 (1994).
66. Fazekas, I. G. & Kósa, F. *Forensic Fetal Osteology*. (Akadémiai Kiadó, 1978).
67. Stloukal, M. & Hanáková, H. Die länge der Längsknochen altslawischer Bevölkerungen unter besonderer Berücksichtigung von Wachstumsfragen. *Homo* **29**, 53–69 (1978).
68. Ubelaker, D. H. *Human Skeletal Remains: Excavation, Analysis, Interpretation*. (Aldine, Chicago: , 1978).
69. Cunningham, C., Scheuer, L. & Black, S. *Developmental Juvenile Osteology*. (Elsevier Academic, London,

2016).

70. Brooks, S. & Suchey, J. M. Skeletal age determination based on the os pubis: A comparison of the Acsádi-Nemeskéri and Suchey-Brooks methods. *J. Hum. Evol.* **5**, 227–238 (1990).

71. Brothwell, D. R. *Digging Up Bones: The Excavation, Treatment and Study of Human Skeletal Remains*. (The British Museum (Natural History), London, 1965).

72. Szilvassy, J. Altersdiagnose am Skelett. in *Anthropologie : Handbuch der vergleichenden Biologie des Menschen* (eds. Knussmann, R. & Martin, R.) 421–443 (Gustav Fischer Verlag, Stuttgart , 1988).

73. Szilvassy, J. Eine Methode zur Altersbestimmung mit Hilfe der sternalen Gelenksflächen der Schlüsselbeine. *Mitteilungen der Anthropologischen Gesellschaft Wien* **108**, 166–168 (1978).

74. Boldsen, J. L., Milner, G. R., Konigsberg, L. W. & Wood, J. W. Transition analysis: a new method for estimating age from skeletons. in *Paleodemography* (eds. Hoppa, R. D. & Vaupel, J. W.) 73–106 (Cambridge University Press, Cambridge, 2002).

75. Getz, S. M. The use of transition analysis in skeletal age estimation. *WIREs Forensic Sci* **2**, (2020).

76. Popli, D., Peyrégne, S. & Peter, B. M. KIN: a method to infer relatedness from low-coverage ancient DNA. *Genome Biol.* **24**, 10 (2023).

77. Ringbauer, H. *et al.* Accurate detection of identity-by-descent segments in human ancient DNA. *Nat. Genet.* **56**, 143–151 (2024).

78. Ringbauer, H., Novembre, J. & Steinrücken, M. Parental relatedness through time revealed by runs of homozygosity in ancient DNA. *Nat. Commun.* **12**, 5425 (2021).

79. Ning, C. *et al.* Ancient genomes from northern China suggest links between subsistence changes and human migration. *Nat. Commun.* **11**, 2700 (2020).

80. Damgaard, P. de B. *et al.* 137 ancient human genomes from across the Eurasian steppes. *Nature* **522**, 207 (2018).

81. Goldberg, A., Günther, T., Rosenberg, N. A. & Jakobsson, M. Ancient X chromosomes reveal contrasting sex bias in Neolithic and Bronze Age Eurasian migrations. *Proc. Natl. Acad. Sci. U. S. A.* **114**, 2657–2662 (2017).

82. Skoglund, P. *et al.* Genomic insights into the peopling of the Southwest Pacific. *Nature* **538**, 510–513 (2016).

83. Fenner, J. N. Cross-cultural estimation of the human generation interval for use in genetics-based population divergence studies. *Am. J. Phys. Anthropol.* **128**, 415–423 (2005).

84. Chintalapati, M., Patterson, N. & Moorjani, P. The spatiotemporal patterns of major human admixture events during the European Holocene. *Elife* **11**, (2022).

85. Schulz, I. *et al.* Ancient genomes from Moravia evidence demographic spread of Slavic-associated groups. In review (2025 (forthcoming)). PRJEB39997.

86. Lauermann, E., Adler, H., Bemmann, J. & Schmauder, M. Die Langobardenforschung im norddanubischen Niederösterreich und im Tullnerfeld. *Kulturwandel in Mitteleuropa* (2008).

87. Gretzinger, J. *et al.* The Anglo-Saxon migration and the formation of the early English gene pool. *Nature* **610**, 112–119 (2022).

88. Leslie, S. *et al.* The fine-scale genetic structure of the British population. *Nature* **519**, 309–314 (2015).

89. International Multiple Sclerosis Genetics Consortium *et al.* Genetic risk and a primary role for cell-mediated immune mechanisms in multiple sclerosis. *Nature* **476**, 214–219 (2011).

90. Genetic Analysis of Psoriasis Consortium & the Wellcome Trust Case Control Consortium 2 *et al.* A genome-wide association study identifies new psoriasis susceptibility loci and an interaction between HLA-C and ERAP1. *Nat. Genet.* **42**, 985–990 (2010).

91. Urnikyte, A. *et al.* Patterns of genetic structure and adaptive positive selection in the Lithuanian population from high-density SNP data. *Sci. Rep.* **9**, 9163 (2019).

92. Tamm, E. *et al.* Genome-wide analysis of Corsican population reveals a close affinity with Northern and Central Italy. *Sci. Rep.* **9**, 13581 (2019).

93. Mallick, S. *et al.* The Simons Genome Diversity Project: 300 genomes from 142 diverse populations. *Nature* **538**, 201–206 (2016).

94. Pagani, L. *et al.* Genomic analyses inform on migration events during the peopling of Eurasia. *Nature* **538**, 238–242 (2016).

95. International HapMap 3 Consortium *et al.* Integrating common and rare genetic variation in diverse human populations. *Nature* **467**, 52–58 (2010).

96. Sudmant, P. H. *et al.* An integrated map of structural variation in 2,504 human genomes. *Nature* **526**, 75–81 (2015).

97. Kushniarevich, A. *et al.* Genetic Heritage of the Balto-Slavic Speaking Populations: A Synthesis of Autosomal, Mitochondrial and Y-Chromosomal Data. *PLoS One* **10**, e0135820 (2015).

98. Kovacevic, L. *et al.* Standing at the gateway to Europe--the genetic structure of Western balkan populations based on autosomal and haploid markers. *PLoS One* **9**, e105090 (2014).

99. Genome of the Netherlands Consortium. Whole-genome sequence variation, population structure and demographic history of the Dutch population. *Nat. Genet.* **46**, 818–825 (2014).

100. Behar, D. M. *et al.* No evidence from genome-wide data of a Khazar origin for the Ashkenazi Jews. *Hum. Biol.* **85**, 859–900 (2013).

101. Yunusbayev, B. *et al.* The Caucasus as an asymmetric semipermeable barrier to ancient human migrations. *Mol. Biol. Evol.* **29**, 359–365 (2012).

102. Behar, D. M. *et al.* The genome-wide structure of the Jewish people. *Nature* **466**, 238–242 (2010).

103. Patterson, N., Price, A. L. & Reich, D. Population structure and eigenanalysis. *PLoS Genet.* **2**, e190 (2006).

104. Alexander, D. H., Novembre, J. & Lange, K. Fast model-based estimation of ancestry in unrelated individuals. *Genome Res.* **19**, 1655–1664 (2009).

105. Antonio, M. L. *et al.* Ancient Rome: A genetic crossroads of Europe and the Mediterranean. *Science* **366**, 708–714 (2019).

106. Olalde, I. *et al.* Cosmopolitanism at the Roman Danubian Frontier, Slavic Migrations, and the Genomic Formation of Modern Balkan Peoples. *bioRxiv* 2021.08.30.458211 (2021) doi:10.1101/2021.08.30.458211.

107. Underhill, P. A. *et al.* The phylogenetic and geographic structure of Y-chromosome haplogroup R1a. *Eur. J. Hum. Genet.* **23**, 124–131 (2015).

108. Stolarek, I. *et al.* Genetic history of East-Central Europe in the first millennium CE. *Genome Biol.* **24**, 1–20 (2023).

109. Peltola, S. *et al.* Genetic admixture and language shift in the medieval Volga-Oka interfluve. *Curr. Biol.* **33**, 174–182.e10 (2023).

110. Lazaridis, I. *et al.* The genetic history of the Southern Arc: A bridge between West Asia and Europe. *Science* **377**, eabm4247 (2022).

111. Scott, G. R. & Turner, C. G. *The Anthropology of Modern Human Teeth: Dental Morphology and Its Variation in Recent Human Populations. Cam- Bridge*. (Cambridge University Press, New York, 1997).

112. Richard Scott, G. & Turner, C. G. *The Anthropology of Modern Human Teeth: Dental Morphology and Its Variation in Recent Human Populations*. (Cambridge University Press, 2000).

113. Brooks, P. J., Enoch, M.-A., Goldman, D., Li, T.-K. & Yokoyama, A. The alcohol flushing response: an unrecognized risk factor for esophageal cancer from alcohol consumption. *PLoS Med.* **6**, e50 (2009).

114. Borinskaya, S. *et al.* Distribution of the alcohol dehydrogenase ADH1B*47His allele in Eurasia. *Am. J. Hum. Genet.* **84**, 89–92; author reply 92–4 (2009).

115. Peng, Y. *et al.* The ADH1B Arg47His polymorphism in east Asian populations and expansion of rice domestication in history. *BMC Evol. Biol.* **10**, 15 (2010).

116. Evershed, R. P. *et al.* Dairying, diseases and the evolution of lactase persistence in Europe. *Nature* **608**, 336–345 (2022).

117. Andersen, J. D. *et al.* Importance of nonsynonymous OCA2 variants in human eye color prediction. *Mol. Genet. Genom. Med.* **4**, 420–430 (2016).

118. Murray, N., Norton, H. L. & Parra, E. J. Distribution of two OCA2 polymorphisms associated with pigmentation in East-Asian populations. *Hum Genome Var* **2**, 15058 (2015).

119. Martin, A. R. *et al.* An Unexpectedly Complex Architecture for Skin Pigmentation in Africans. *Cell* **171**, 1340–1353.e14 (2017).

120. Ju, D. & Mathieson, I. The evolution of skin pigmentation-associated variation in West Eurasia. *Proc. Natl. Acad. Sci. U. S. A.* **118**, e2009227118 (2021).

121. Li, H. & Durbin, R. Fast and accurate short read alignment with Burrows–Wheeler transform. *Bioinformatics* **25**, 1754–1760 (2009).

122. Chen, H., Lu, Y., Lu, D. & Xu, S. Y-LineageTracker: a high-throughput analysis framework for Y-chromosomal next-generation sequencing data. *BMC Bioinformatics* **22**, 114 (2021).

123. Link, V. *et al.* ATLAS: Analysis Tools for Low-depth and Ancient Samples. *bioRxiv* 105346 (2017) doi:10.1101/105346.

124. Martiniano, R., De Sanctis, B., Hallast, P. & Durbin, R. Placing ancient DNA sequences into reference phylogenies. *Mol. Biol. Evol.* **39**, msac017 (2022).

125. Weissensteiner, H. *et al.* HaploGrep 2: mitochondrial haplogroup classification in the era of high-throughput sequencing. *Nucleic Acids Res.* **44**, W58–63 (2016).

126. Renaud, G., Slon, V., Duggan, A. T. & Kelso, J. Schmutzi: estimation of contamination and endogenous mitochondrial consensus calling for ancient DNA. *Genome Biol.* **16**, 224 (2015).

127. Liu, B.-L. *et al.* Paternal origin of Tungusic-speaking populations: Insights from the updated phylogenetic tree of Y-chromosome haplogroup C2a-M86. *Am. J. Hum. Biol.* **33**, e23462 (2021).

128. Semino, O. *et al.* The genetic legacy of Paleolithic Homo sapiens sapiens in extant Europeans: a Y chromosome perspective. *Science* **290**, 1155–1159 (2000).

129. Olalde, I. *et al.* A genetic history of the Balkans from Roman frontier to Slavic migrations. *Cell* **186**, 5472–5485.e9 (2023).

130. Margaryan, A. *et al.* Population genomics of the Viking world. *Nature* **585**, 390–396 (2020).

131. Rodríguez-Varela, R. *et al.* The genetic history of Scandinavia from the Roman Iron Age to the present. *Cell* **186**, 32–46.e19 (2023).

132. Rootsi, S. *et al.* Phylogeography of Y-chromosome haplogroup I reveals distinct domains of prehistoric gene flow in europe. *Am. J. Hum. Genet.* **75**, 128–137 (2004).

133. Maróti, Z. *et al.* Whole genome analysis sheds light on the genetic origin of Huns, Avars and conquering Hungarians. *bioRxiv* 2022.01.19.476915 (2022) doi:10.1101/2022.01.19.476915.

134. Neparáczki, E. *et al.* Y-chromosome haplogroups from Hun, Avar and conquering Hungarian period

nomadic people of the Carpathian Basin. *Sci. Rep.* **9**, 16569 (2019).

135. Patterson, N. *et al.* Ancient admixture in human history. *Genetics* **192**, 1065–1093 (2012).

136. Lewis, M. E. *The Bioarchaeology of Children: Perspectives from Biological and Forensic Anthropology*. (Cambridge University Press, 2007).

137. Holmgren, J. Observations on marriage and inheritances practices in early mongol and yüan society, with particular reference to the levirate. *J. South Asian Nat. Hist.* **20**, 127–192 (1986).

138. Krader, L. Principles and Structures in the Organization of the Asiatic Steppe-Pastoralists. *Southwest. J. Anthropol.* **11**, 67–92 (1955).

139. Broadbridge, A. F. *Women and the Making of the Mongol Empire*. (Cambridge University Press, 2018).

140. Sellen, D. W. & Mace, R. Fertility and Mode of Subsistence: A Phylogenetic Analysis. *Curr. Anthropol.* **38**, 878–889 (1997).

141. Štular, B. *Grave Orientation In The Middle Ages: A Case Study from Bled Island*. (Založba ZRC, 2022).

142. Harrington, S., Brookes, S., Semple, S. & Millard, A. Theatres of Closure: Process and Performance in Inhumation Burial Rites in Early Medieval Britain. *Cambridge Archaeological Journal* **30**, 389–412 (2020).

143. Brookes, S., Harrington, S., Walther, L., Semple, S. & Gowland, B. Demography beyond typology: revisualising death and data. in *New Narratives for the First Millennium AD? : Alte und neue Perspektiven der archäologischen Forschung zum 1. Jahrtausend n. Chr.* (eds. Ludowici, B. & Pöppelmann, H.) 245–264 (Wendeburg: Uwe Krebs, Wendeburg, 2022).

144. Tobias, B., Wiltschke-Schrotta, K. & Binder, M. Das langobardenzeitliche Gräberfeld von Wien-Mariahilfer Gürtel. Mit einem Beitrag zur künstlichen Schädeldeformation im westlichen Karpatenbecken. *Jahrb. RGZM* **57**, 279–337 (2010).

145. Müller, S. *Das Awarische Gräberfeld in Zwölfaxing, Burstyn-Kaserne*. (NÖ Institut für Landeskunde, St. Pölten, 2015).

146. Scharrer-Liška, G. Das awarenzeitliche Gräberfeld von Frohsdorf, Niederösterreich – Ein Zwischenbericht. in *Die Leitha – Facetten einer Landschaft.* (eds. Doneus, M. & Griebl, M.) 119–129

(Österreichische Gesellschaft für Ur- und Frühgeschichte c/o Institut für Urgeschichte und Historische Archäologie, Wien, 2015).

147. Кубарев, Г. В. *Культура древних тюрок Алтая (по материалам погребальных памятников) (The Culture of the Ancient Turks of the Altai (on the Basis of the Burials)*. 62–72, 171–172 (Rossijskaja Akad. Nauk, Sibirskoe Otd., Inst. Archeologii i Ėtnografii, Novosibirsk, 2005).

148. Lőrinczy, G. A szegvár-oromdűlői kora avar kori temető (The Avar-period populations of Szegvár-Oromdűlő and the Trans-Tisza Region). in *A szegvár-oromdűlői temető és a Tiszántúl kora avar időszaka. (The Szegvár-Oromdűlő Cemetery and the Avar Period in the Trans-Tisza Region)* (eds. Lőrinczy, G. & Türk, B. M. &.) 11–404 (Budapest, Szeged, Szentes, 2022).

149. Horváth Á. M. Female gender markers in graves of men in the Avar Period of the Middle Tisza region. *Communicationes Archaeologicae Hungariae* **2021**, 41–67 (2023).

150. Lippert, A. *Das awarenzeitliche Gräberfeld von Zwölfaxing in Niederösterreich*. (Berger, 1969).

151. Daim, F. *Das awarische Gräberfeld von Sommerein am Leithagebirge*. (Verlag der Österreichischen Akademie der Wissenschaften, 1984).

152. Bachner, M. Das awarische Gräberfeld von Münchendorf, Niederösterreich. Berichte des Symposions der Kommission für Frühmittelalterforschung. in *Die Bayern und ihre Nachbarn.* 69–122 (1985).

153. Pleyer, A. Die Perlen des awarischen Gräberfeldes von Zillingtal. (University Vienna, 2005).

154. Albrecht, S. Warum tragen wir einen Gürtel? Der Gürtel der Byzantiner - Symbolik und Funktion. In Byzanz - Das Römerreich im Mittelalter 1. in *Welt der Ideen, Welt der Dinge* (eds. Daim, F. & Drauschke, J.) 79–95 (Monographien des Römisch-Germanischen Zentralmuseums 84, Mainz, 2010).

155. Fancsalszky G. *Állat- és emberábrázolás a késő avar kori öntött bronz övvereteken*. (Martin Opitz Kiadó, Budapest, 2007).

156. Göckenjan, H. Bogen, Pfeil und Köcher in der Herrschafts- und Rechtssymbolik der eurasischen Steppenvölker. *Acta Orientalia Academiae Scientiarum Hungaricae* **58**, 59–76 (2005).

157. Csiky, G. *Avar-Age Polearms and Edged Weapons: Classification, Typology, Chronology and Technology*. (BRILL, Leiden, Boston, 2015).

158. Barbiera, I. Remembering the Warriors: Weapon Burials and Tombstones between Antiquity and the Early Middle Ages in Northern Italy. in *Post-Roman Transitions* (eds. Pohl, W. & Heydemann, G.) vol. 14 407–435 (Brepols Publishers, 2013).

159. Härke, H. 'Warrior Graves'?: The Background of the Anglo-Saxon Weapon Burial Rite. **126**, 22–43 (1990).

160. Weiner, A. B. *Inalienable Possessions: The Paradox of Keeping-While Giving*. (University of California Press, Berkeley and Los Angeles, 1992).

161. Szentpéteri, J. Kígyómotívum a griffes-indás népesség hagyatékában—Das Schlangenmotiv in der Hinterlassenschaft des Greifen-Ranken-Ethnikums. *Herman Ottó Múz. évkv.* (1993).

162. Szentpéteri, J. Archäologische Studien zur Schicht der Waffenträger des Awarentums im Karpatenbecken II. *Acta Archaeologica Academiae Scientiarum Hungaricae* vol. 46 231–306 (1994).

163. Szücsi, F. Avar kori balták, bárdok, szekercék és fokosok. Baltafélék a 6–8. századi Kárpát-medencében–Awarenzeitliche Streitäxte und Beile. Äxte im 6–8 …. *Alba Regia* **42**, 113–186 (2014).

164. Wagner, A. Few and well-healed? Injuries of the long bones of Avar period skeletons from the cemetery of Mödling - An der Goldenen Stiege". (University of Vienna, 2023). doi:10.25365/THESIS.73649.

165. Mühlburger, M. Peaceful Avars? Preprint at https://doi.org/10.25365/THESIS.73121 (2023).

166. Wiltschke-Schrotta, K. & Stadler, P. Beheading in Avar times (630-800 A.D.). *Acta Medica Lituanica* **12**, 58–64 (2005).

167. Perémi, S. Á. Avar kori mellboglárok a Balatonudvari-Fövenyes temetőből. in *Vitézek, mi lehet ez széles föld felett szebb dolog a végeknél? Tanulmánykötet Gere László tiszteletére 60. születésnapja alkalmából,* (eds. Velladics, M. & Vizi, M.) (Martin Opitz Kiadó, Budapest, 2022).

168. Early fashion influencers or just ordinary women? in *Avari i Slaveni dvije strane pojasnog jezičca - Avari na sjeveru i jugu kaganata. (Avars and Slavs. Two sides of a belt strap end - Avars on the north and the south of the Khaganate* (eds. Papeša, A. R. & Dugonjić, A.) 88–105 (Zagreb, 2022).

169. Barbiera, I. Cross Dressing and Gender Transgression in the Middle Ages. Introduction. in *Il genere nella ricerca storica. Atti del 6° Congresso della Società italiana* (eds. Chemotti, S. & La Rocca, M. C.) 117–127

(Il Poligrafo, Padova, 2015).

170. Shannon, P. *et al.* Cytoscape: a software environment for integrated models of biomolecular interaction networks. *Genome Res.* **13**, 2498–2504 (2003).

171. Csardi, G. & Nepusz, T. The igraph software package for complex network research. *InterJournal, complex systems* **1695**, 1–9 (2006).

172. Krivitsky, P. N., Hunter, D. R., Morris, M. & Klumb, C. Ergm 4: New features for analyzing exponential-family random graph models. *J. Stat. Softw.* **105**, (2023).

173. Hunter, D. R., Handcock, M. S., Butts, C. T., Goodreau, S. M. & Morris, M. ergm: A Package to Fit, Simulate and Diagnose Exponential-Family Models for Networks. *J. Stat. Softw.* **24**, nihpa54860 (2008).

174. Wilson, J. D., Denny, M. J., Bhamidi, S., Cranmer, S. J. & Desmarais, B. A. Stochastic weighted graphs: Flexible model specification and simulation. *Soc. Networks* **49**, 37–47 (2017).
