# Supplementary material for: Ancient DNA reveals reproductive barrier despite shared Avar-period culture
Source: Nature. 2025 Jan 15;638(8052):1007–14. doi: 10.1038/s41586-024-08418-5 (PMC11864967; doi:10.1038/s41586-024-08418-5)
Supplement: Supplementary file 3 — Supplementary Tables 1–8 and a Table guide. [file 41586_2024_8418_MOESM3_ESM.zip › Table-guides.pdf]

Supplementary Tables Legends

Supplementary Table 1. Meta information of newly reported data from Leobersdorf, Mödling and Csokorgasse. A. Summary statistic for each sequencing library. B. List of low data quality individuals in Leobersdorf, Mödling and Csokorgasse.

Supplementary Table 2. Ancestry decomposition from qpAdm estimates. A. Ancestry modeling results for each individual in Leobersdorf. B. Ancestry modeling results for each individual in Mödling. C. Ancestry modeling results for each individual in Csokorgasse.

Supplementary Table 3. IBD sharing between and within sites and individuals newly reported in this study. A. IBD sharing within Leobersdorf. B. IBD sharing within Mödling. C. IBD sharing within Csokorgasse. D. IBD sharing across sites.

Supplementary Table 4. Relatedness estimates from KIN, BREADR in Leobersdorf, Mödling and Csokorgasse.

Supplementary Table 5. Pairs of genetically identical individuals. A. List of possible twins in Leobersdorf and Mödling. B. List of genetically identical individuals resulting from commingled skeletons.

Supplementary Table 6. The mean and median values of ROH distributions in Leobersdorf, Mödling and Csokorgasse. The p-value from distribution similarity test by two sample Kolmogorov-Sminov test using ks.test function in R. The p-values lower than 0.05 suggests the two distributions are statistically significantly different.

Supplementary Table 7. Pairs of potential levirate and sororate unions in Leobersdorf and Mödling.

Supplementary Table 8. Number of individuals counted for anthropological questions in Leobersdorf, Mödling and Rákóczifalva.
